# Retroposed Elements as Archives for the Evolutionary History of Placental Mammals

**DOI:** 10.1371/journal.pbio.0040091

**Published:** 2006-03-14

**Authors:** Jan Ole Kriegs, Gennady Churakov, Martin Kiefmann, Ursula Jordan, Jürgen Brosius, Jürgen Schmitz

**Affiliations:** **1**Institute of Experimental Pathology, Center for Molecular Biology of Inflammation, University of Münster, Münster, Germany; Massey UniversityNew Zealand

## Abstract

Reconstruction of the placental mammalian (eutherian) evolutionary tree has undergone diverse revisions, and numerous aspects remain hotly debated. Initial hierarchical divisions based on morphology contained many misgroupings due to features that evolved independently by similar selection processes. Molecular analyses corrected many of these misgroupings and the superordinal hierarchy of placental mammals was recently assembled into four clades. However, long or rapid evolutionary periods, as well as directional mutation pressure, can produce molecular homoplasies, similar characteristics lacking common ancestors. Retroposed elements, by contrast, integrate randomly into genomes with negligible probabilities of the same element integrating independently into orthologous positions in different species. Thus, presence/absence analyses of these elements are a superior strategy for molecular systematics. By computationally scanning more than 160,000 chromosomal loci and judiciously selecting from only phylogenetically informative retroposons for experimental high-throughput PCR applications, we recovered 28 clear, independent monophyly markers that conclusively verify the earliest divergences in placental mammalian evolution. Using tests that take into account ancestral polymorphisms, multiple long interspersed elements and long terminal repeat element insertions provide highly significant evidence for the monophyletic clades Boreotheria (synonymous with Boreoeutheria), Supraprimates (synonymous with Euarchontoglires), and Laurasiatheria. More importantly, two retropositions provide new support for a prior scenario of early mammalian evolution that places the basal placental divergence between Xenarthra and Epitheria, the latter comprising all remaining placentals. Due to its virtually homoplasy-free nature, the analysis of retroposon presence/absence patterns avoids the pitfalls of other molecular methodologies and provides a rapid, unequivocal means for revealing the evolutionary history of organisms.

## Abstract 2

The authors identified and sequenced retroposons in mammalian genomes. The presence and absence of these retroposons provided evolutionary markers from which the authors reconstructed the phylogenetic history of placental mammals.

## Introduction

The recent “large-scale” compilations of available sequence information to reconstruct the mammalian phylogenetic tree categorized the placental mammals into four superordinal clades or lineages [ 1, 2], a categorization that has been confirmed by other studies as well [ 3, 4]: (I) Afrotheria, a diverse group mainly distributed in Africa; (II) Xenarthra, a southern North American- and South American-distributed group; (III) Supraprimates [ 1, 5] (synonymous with Euarchontoglires [ 2, 6]), a superordinal clade assembled from molecular genetic results, combining the Glires clade (Rodentia and Lagomorpha) with that of the Euarchonta (Scandentia, Dermoptera, and Primates); and (IV) Laurasiatheria, a group compiled from molecular data including cetartiodactyls (Cetacea and even-toed ungulates), perissodactyls (odd-toed ungulates), carnivores, pangolins, bats, and eulipotyphlan insectivors [ 1, 2, 6– 10].

While most studies recover the taxon Boreotheria [ 1] (synonymous with Boreoeutheria [ 11], a name that has been suggested because early fossils of this group have been found in the Northern Hemisphere), comprising the sister taxa Laurasiatheria and Supraprimates, questions about the first divergence in the placental mammalian tree remain [ 4, 12]. Xenarthra and Epitheria (all remaining placentals [ 13]), or Atlantogenata (Afrotheria and Xenarthra), as sister taxon to all other placentals [ 4], are possible hypotheses for early placental evolution. As a third hypothesis, the recent large-scale compilations [ 1, 2, 7, 8] suggest an out-of-Africa scenario with basal Afrotheria and a monophyletic clade Exafricomammalia (Boreotheria and Xenarthra) [ 4].

However, there are some important issues that must be taken into consideration when using sequence data alone to answer these questions. For example, Bayesian branch-support values as used by Murphy et al. [ 2] should not be interpreted as probabilities that a tree-topology is correct and are known to overestimate the degree of clade support [ 14]. Species sampling and missing data have strong impacts on sequence analyses [ 1, 12, 15, 16]. Furthermore, combining nuclear and mitochondrial sequences may lead to artificial branchings, because the nucleotide composition plasticity of some mammalian mitochondrial genomes may interfere with phylogenetic reconstructions. The erroneous clustering of the colugo within primates by Murphy et al. [ 7] is one such example [ 17, 18].

Rare genomic changes, such as indels, can be used as an independent evaluation of phylogenetic relationships, and they have been successfully used as temporal landmarks of evolution [ 10, 19– 23]. Retroposed elements provide an exceptionally informative source of rare genomic changes. They are a virtually ambiguity-free approximation of evolutionary history [ 24, 25]. The nearly homoplasy-free character and innate complexity of retroposed elements in mammalian species, coupled with their high abundance, enables phylogenetic reconstructions based on a variety of alternative markers. For example, retropositions provided conclusive evidence for the position of whales (Cetacea) within Cetartiodactyla [ 26], the monophyly of Afrotheria [ 27], hominoid relationships [ 28], and the topology of the primate strepsirrhine tree [ 29]. The coincidence of perfectly orthologous insertions of retroposons belonging to the same subtype, showing shared diagnostic mutations compared with the known consensus sequence, and in some cases exactly the same truncations, is extremely unlikely. The only significant limitation of this method is that nodes difficult to resolve by sequence data (short branches) are also rarely supported by presence/absence patterns of retroposed elements [ 30].

To overcome this limitation, we have developed several strategies to search for and recover phylogenetically informative retroposons in the current genomic data (i.e., completed genomes for a few species and large fragments of several others). The “presence” of given retroposed elements in related taxa implies their orthologous integration, a derived condition acquired via a common ancestry, while the “absence” of particular elements indicates the plesiomorphic condition prior to integration in more distant taxa. The use of presence/absence analyses to reconstruct the systematic biology of mammals depends on the availability of retroposed elements that were actively integrating before the divergence of a particular species. Since long interspersed elements (LINE1) and long terminal repeat (LTR) elements were active at the critical time points of mammalian divergences [ 31], we focused our investigations on these retroposons.

Precise excision [ 32], hotspots of insertions [ 33, 34], and incomplete lineage sorting [ 28] of retroposed elements are thought to be extremely rare events in mammalian evolution. Thus, there is a very low probability of insertion homoplasy. Nevertheless, we performed a statistical test for all five investigated nodes [ 1] and revealed significant support for all branches except for the Epitheria divergence.

## Results/Discussion

**Figure 1:**
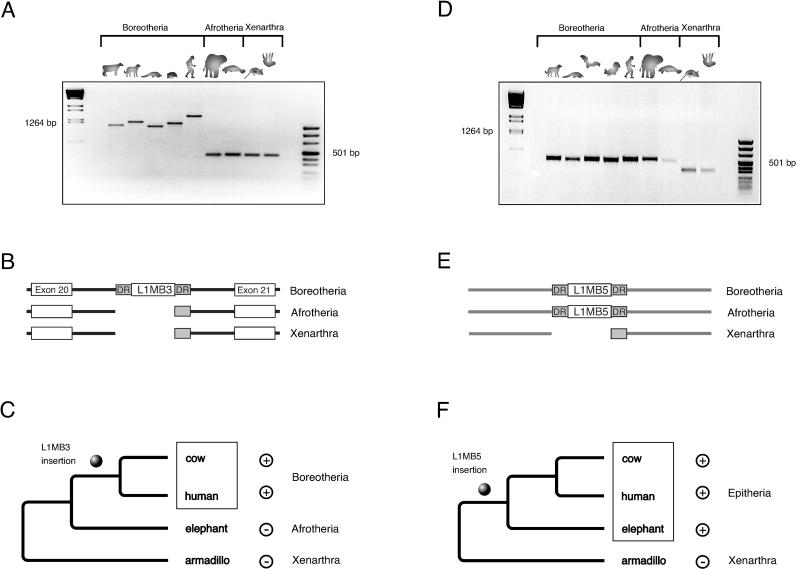
Two Examples of Presence/Absence Analyses (A) Genomic PCR fragments. The L1MB3 element is present in all boreotherian species. The element is located between exon 20 and 21 of the human *AP4E1* gene on human Chromosome 15 (q21.2). Small-size variations are due to random indels. The larger fragment for human is due to an additional insertion of an *Alu* Sx element. Smaller fragments in afrotherians and xenarthrans indicate the absence situation prior to insertion of the element (plesiomorph condition). (B) A schematic representation of the presence/absence loci of various taxa after sequence determination. Direct repeats and the unoccupied target sites are shaded gray. (C) A phylogenetic interpretation of the presence/absence pattern. The L1MB3 element is present (+) in representatives of boreotherians and absent (−) in afrotherians and xenarthrans. The ball indicates the integration time of the L1MB3 element prior to the common ancestor of all recent boreotherians, but after this lineage separated from other placentals. The relative time of this integration is represented by node 3 in Figure 2; ten other integrations confirm the boreotherian hypothesis. (D) Genomic PCR fragments. The L1MB5 element, in addition to its presence in all boreotherian species is also found in the afrotherian species. The smaller fragments in xenarthrans indicate its absence in these species. Its integration site corresponds to the human locus on Chromosome 15 (q23). (E) A schematic representation of the presence/absence loci of various taxa after sequence determination. Direct repeats and the unoccupied target sites are shaded gray. (F) The L1MB5 element is present (+) in representatives of boreotherians and afrotherians, grouping them in the clade Epitheria, and is absent (−) in xenarthrans. The ball indicates the integration time of the L1MB5 element prior to the common ancestor of all Epitheria, but after this lineage separated from other placentals. This integration time is the same as node 2 in Figure 2, and we have so far recovered one additional retroposon integration to support the Epitheria hypothesis. DR, direct repeats.

We scanned approximately 4.4 gigabases of human, dog, and mouse genomic sequences with RepeatMasker looking for the presence or absence of retroposed elements surrounded by highly conserved sequence regions (more than 75% similarity in pair-wise comparisons of different mammals). Primers for high-throughput PCR were designed from 237 of these loci and presence/absence-informative fragments were amplified from the genomes of representatives of all four placental superorders. When the amplified PCR products demonstrated evident fragment size shifts, indicating presence of a retroposed element in one and absence in another taxon ( Figure 1) in orthologous loci clearly evidenced by sequence comparisons, we extended the taxon sampling for both amplification and sequence analyses. Selected for further characterization were 28 such presence/absence patterns. The remaining loci were not phylogenetically informative for the early mammalian divergences, either because the retroposon was present in only one or in all species, or because it was not amplifiable in critical taxa.

**Figure 2:**
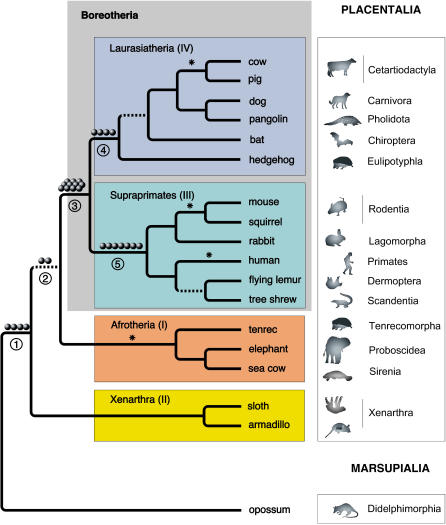
Positions of Retroposed Elements as Landmarks of Evolution on the Bayesian-Based Placental Evolutionary Tree from Murphy et al. [ 2] The resultant tree is consistent with previous studies [ 1, 2, 4, 5, 7, 8, 10, 38, 39] in most aspects. Note that the positions of afrotherians and xenarthras have been reversed, based on the presence of two retroposon insertions at node 2. Gray balls represent single insertion events. Supported splitting points are labeled with Arabic numerals. Superordinal clades, in the order shown, were established by Waddell et al. [ 6] and supported by several major studies [ 1, 2, 7, 8], and are labeled with Roman numerals. The taxa shown represent only those from which we sampled LINEs and LTRs. Dotted lines indicate nodes in need of further confirmation. Asterisks represent retroposon evidence from the literature for monophyly of Afrotheria [ 27], Primates [ 18], Rodentia [ 45], and Cetartiodactyla [ 26].

All 28 presence/absence patterns were verified by complete sequence analyses in all investigated taxa. This enabled us to establish clear orthology and to compare identical retroposons in different species. As most of the analyzed elements are 5′-truncated forms of the original retroposon, the shared point of truncation in all species harboring the element is evidence that the respective insertions are identical by descent rather than conversion. Together these features make it highly unlikely that our markers represent independent insertional events such as those common to retropositional hotspots [ 33]. Remarkably, despite the extensive sequence drift that can occur during 80–100 million years of random mutation, in several cases we could, after very careful sequence alignment, still recognize short direct repeats flanking the retroposed elements, as well as the unoccupied singular target sites of species that diverged before the transposition occurred.

**Figure 3:**
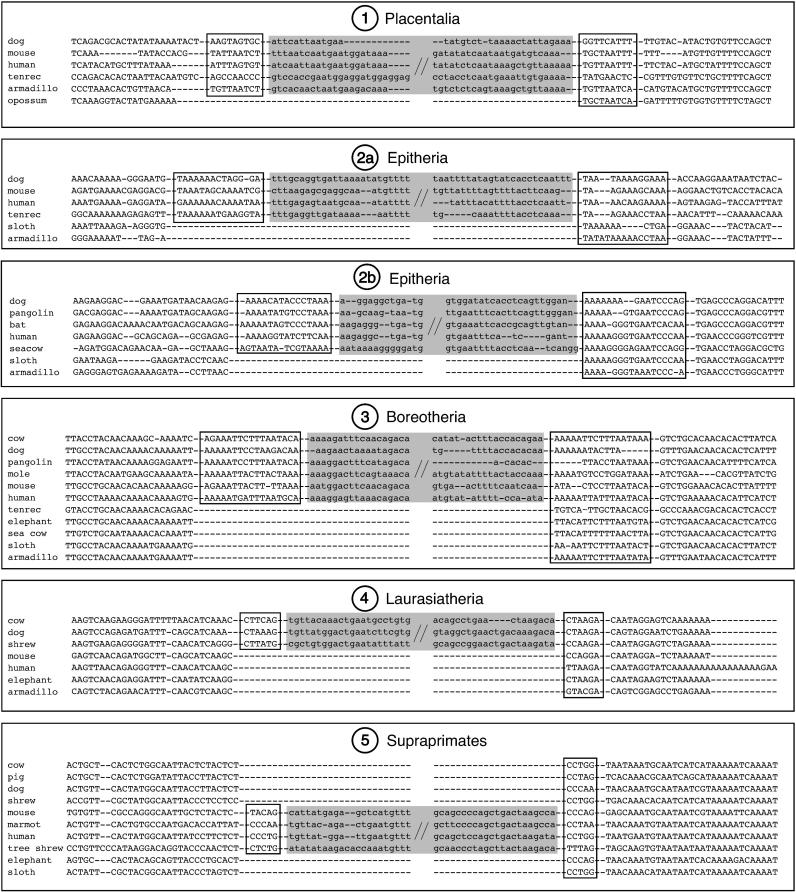
Representative Alignments of the Presence/Absence Regions Indicating Support for the Five Investigated Evolutionary Divergences Potential direct repeats are boxed. The 5′ and 3′ ends of the retroposon insertions are partially shown in lower case letters on a gray background. Node designations corresponding to Figure 2 and the names of the supported monophyletic groups are given above the inserted elements.

Using the Bayesian tree from Murphy et al. [ 2] as a framework, we evaluated the evolutionary relatedness of representatives of the major placental mammalian taxonomic orders by examining the presence/absence patterns of all 28 retroposon markers. All markers represent independent insertions and are distributed throughout the genome ( Figure S1). The results of this analysis provide evidence to substantiate several superordinal divergences in the placental mammalian evolutionary tree and suggest new support for xenarthrans as the basal branch ( Figures 2 and 3).

(1) Four L1 elements (L1MB4a, L1MB7, and 2X L1MB8) were present at their respective, orthologous loci in all species tested except the opossum. As there is general agreement on the monophyly of placentals [ 13], it can be defined as a clear prior hypothesis and all competing hypotheses can be rejected ( *p* = 0.0123; [4 0 0] [ 1]). Moreover, these four unambiguous presence/absence patterns demonstrate the effectiveness of using retroposons as phylogenetic markers, even when the evolutionary divergence occurred more than 100 million years ago, long enough for high-sequence divergences and/or large deletions between both taxa to have occurred.

(2) Two insertions of an L1 (L1MB5) element were detected that unite the Boreotheria and Afrotheria to the exclusion of Xenarthra, suggesting that the latter constitute the most basal branch of the placental mammalian tree, thus inverting the basal branching proposed by Murphy et al. [ 2, 7]. Assuming a clear prior hypothesis for Epitheria [ 13], there is only a small chance ( *p* = 0.111; [2 0 0] [ 1]) of these occurring due to ancestral polymorphism. However, since there are actually three formulated hypotheses [ 1, 2, 4, 13] and obvious ambiguity about this part of the tree, Epitheria might not serve as a clear prior hypothesis, thus possibly decreasing the significance of the data ( *p* = 0.333). Note that due to the small amount of genomic sequences available for both xenarthrans and afrotherians, genomic searches starting predominantly from human sequence information are biased for Epitheria and Exafricomammalia. However, the lack of any evidence in support of Exafricomammalia is therefore surprising and cannot be due to the same bias. An additional argument against a cluster of Afrotheria and Xenarthra is that in our high-throughput PCR amplifications we found no secondary integrations merging those two taxa. Secondary integrations are additional random insertions of transposed elements and their recovery is therefore independent of any search strategy based on pre-selected potential informative phylogenetic markers (see also Schmitz et al. [ 35]).

Interestingly, morphologists have long proposed an Epitheria hypothesis in which Xenarthra are the sister group to all other placentals [ 36]. In contrast, by Bayesian tree reconstruction, Afrotheria have been reported to constitute the earliest divergence of placentals [ 2]. Nevertheless, although the splitting interval at the early placental divergence may have been too short to allow fixation of many diagnostic retroposon integrations we were able to find markers supporting the Epitheria hypothesis by scanning nearly 11 million elephant and 10 million armadillo trace sequences, each for L1MB5 insertions. Since, in contrast to the other investigated divergences, we have identified only two such insertions so far, the implication of this data will surely stimulate further searches and investigations and a reconsideration of the early evolution of mammals, and therewith revitalize the classical, morphologically-based Epitheria hypothesis.

(3) We found 11 L1 (7X L1MB3, 2X L1MB4, and 2X L1MB5) elements that were present in all Supraprimates and Laurasiatheria and absent in Afrotheria and Xenarthra. The species of these two superordinal clades comprise the Boreotheria. Taking this as the only clear prior hypothesis [ 1, 2, 6– 8], there is little chance of this data occurring under any other tree ( *p* < 0.0001; [11 0 0] [ 1]), and all alternative hypotheses of the placental tree can be clearly rejected. In contrast to the strong mitochondrial signal for boreotherian paraphyly [ 37], which contradicts other mitochondrial studies [ 1, 4, 5, 38, 39], our retroposon data validate results drawn from predominantly nuclear sequences [ 1, 2, 7, 8].

(4) Four retroposed elements (L1MA9, 2X MLT1A0, MER34) were present in all Laurasiatheria and clearly support the monophyly of this superordinal clade ( *p* = 0.0123; [4 0 0] [ 1]). Some extensive mitochondrial data analyses consistently place the hedgehog close to the root of the placentals [ 37], while others argue against this [ 4, 5, 40– 42]. The basal divergence can now be firmly excluded by the presence of these four insertions as well as the Boreotherian markers ( Figure 2, node 3).

(5) Orthologous transposed elements (L1MA9, L1MC1, 3X MLT1A0, MLT1A, MER93B) are present at seven different loci in Supraprimates that are absent in other mammals. The concept of a superordinal clade Supraprimates is challenged by some molecular studies based on mitochondrial sequences that, for example, place myomorph rodents as basal placentals [ 37, 43]. Even certain nuclear sequence datasets support such contrasting evolutionary scenarios [ 16]. On the other hand, our data confirm several prior scenarios [ 1, 2, 6– 8, 15, 21, 22, 42]. The newly introduced concept of Supraprimates is now clearly supported by our retroposon analysis ( *p* < 0.004; [7 0 0] [ 1]).

Recently, Bashir et al. [ 44] published a purely computational method for reconstructing the phylogenetic relationships between mammals by automatically scanning for the presence and/or absence of transposed elements in mammalian sequences. However, this use of pure bioinformatics is fraught with pitfalls. The available sequence information is often not reliable, sequence drift makes identifying orthologous insertions extremely difficult, and full sequences are available for only a limited number of species. Extreme care must be taken to conclusively verify that supposedly homoplasmic insertions belong to the same class of transposons and are integrated at orthologous positions. For high-quality, reliable phylogenetic inferences it is essential to individually characterize the nature of each insertion as well as its integration site, a process not amenable to high-throughput computational searches and incomplete species sampling.

On the other hand, the combining of molecular biological methodologies with those of bioinformatics in the analysis of retroposed elements provides a reliable, homoplasy-free reconstruction of phylogenetic trees. In this study, we have unambiguously substantiated the monophyly of the placental, boreotherian, supraprimates, and laurasiatherian mammalian clades with multiple pieces of independent evidence from retroposon presence/absence data. Furthermore, by screening nearly 21 million genomic trace sequences we found two retropositions that lend support to the Epitheria hypothesis [ 13]. Interestingly, this is an area where sequence-based tree analyses have tended to support other trees, but at least some authors have remained skeptical of the ability of automatic tree-building procedures to infer the root of the mammalian tree when all data are known to violate the underlying model of sequence evolution [ 1, 4, 5, 10, 12].

While this report tests the validity of the placental evolutionary tree, the method we present provides a statistically valid, unequivocal means of substantiating all tree reconstructions, and thus affords morphologists, palaeontologists, and molecular evolutionists alike, solid unequivocal platforms for future investigations of mammalian evolution.

## Materials and Methods

### Taxon sampling

We analyzed DNA samples and/or sequences from the following mammalian species, representing all placental orders ( Table S1).

Infraclass Placentalia, Order Xenarthra: Dasypus novemcinctus (nine-banded armadillo) and Choloepus hoffmanni (Hoffmann's two-fingered sloth); Order Proboscidea: Loxodonta africana (African savanna elephant); Order Sirenia: Trichechus manatus (Caribbean manatee); Order Tenrecomorpha: Echinops telfairi (small Madagascar hedgehog, tenrec); Order Scandentia: Tupaia belangeri (northern tree shrew); Order Dermoptera: Cynocephalus variegatus (Malayan flying lemur); Order Primates: Homo sapiens (human), Pan troglodytes (chimpanzee), and Macaca mulatta (rhesus monkey); Order Lagomorpha: Oryctolagus cuniculus (rabbit); Order Rodentia: Mus musculus (house mouse), Rattus norvegicus (Norway rat), Cavia porcellus (Guinea pig), Marmota marmota (European marmot), and Sciurus vulgaris (Eurasian red squirrel); Order Eulipothyphla: Erinaceus europaeus (western European hedgehog), *Talpa europaea* (European mole), and Sorex araneus (European shrew); Order Chiroptera: Rhinolophus hipposideros (lesser horseshoe bat), Rhinolophus ferrumequinum (greater horseshoe bat), Carollia perspicillata (Seba's short-tailed bat), Myotis daubentonii (Daubenton's bat), Myotis lucifugus (little brown bat), *Plecotus austriacus* (gray big-eared bat), and Pipistrellus pipistrellus (common pipistrelle); Order Pholidota: Manis javanica (Malayan pangolin); Order Carnivora: Canis familiaris (dog) and Felis catus (cat); Order Cetartiodactyla: *Sus scrofa domestica* (domestic pig) and Bos taurus (cow). As an outgroup, we used the opossum, Infraclass Marsupialia, Order Didelphimorphia: *Didelphis virginiana* (North American opossum).

### Computational strategies

To find phylogenetically informative loci featuring presence/absence patterns of retroposed elements, we developed several different in silico strategies; a flow-chart outlining these can be found in Figure S2.

### Strategy I

For testing potential sister taxon relationships of human-mouse or human-dog, we downloaded whole genome, pair-wise alignments of these species from the University of California Santa Cruz Server (UCSC) ( http://hgdownload.cse.ucsc.edu/downloads.html; 2.1 and 1.7 gigabases, respectively) and transformed them into FASTA format with our own computer algorithm. As a reference point, we scanned the human sequence with the local version of RepeatMasker (A. F. A. Smit, R. Hubley, and P. Green, http://www.repeatmasker.org) for the presence of retroposed elements, which were then aligned to sequences of other species. Recovered were 120,000 candidate loci with either LINE1 or LTR insertions. From these, another computer algorithm identified loci suitable for further study based on the following criteria: (1) Flanking regions of shared transposed elements were free of other transposed elements, (2) The sizes of the transposed elements were smaller than 1 kilobase (kb) to facilitate routine PCR amplification, and (3) A maximal sequence divergence of 25% was allowed for clear identification of shared retroposed elements. These constraints reduced the number of potential phylogenetic-informative loci to 2,100, which were further examined by eye in Genome Browser ( http://mgc.ucsc.edu/cgi-bin/hgBlat) for the presence and/or absence of retroposed elements and conserved flanking regions in the various representative species. For designing PCR primers, 100 loci were selected.

### Strategy II

We downloaded all the available 186,500 human intronic sequences (547 megabases) from the UCSC Server ( http://genome.ucsc.edu/cgi-bin/hgTables). After excluding duplicated sequences and introns larger than 1 kb we searched for the presence of retroposed elements (RepeatMasker). Introns with primate-specific elements and/or low complexity repeats were excluded. The remaining 514 loci were analyzed for the presence of conserved flanks (UCSC Server, http://genome.ucsc.edu/cgi-bin/hgBlat) and 71 loci were chosen to generate PCR primers.

By screening intronic sequences for presence/absence markers comprising trace sequences of Xenarthra and Afrotheria, we found one marker (L1MB5) supporting xenarthrans basal to all other placentals.

### Strategy III

Approximately 93 megabases of draft sequences from elephant ( Loxodonta africana VMRC15), nine-banded armadillo ( Dasypus novemcinctus VMRC5), and two bat genomes ( Rhinolophus ferrumequinum VMRC7 and *Carollia perspicillata* clones) ( http://www.ncbi.nlm.nih.gov/entrez/query.fcgi?db=nucleotide) were downloaded and searched for retroposed element insertions according to Strategy I, conditions 1 and 2. A total of 206 elephant, 5,632 armadillo, and 1,027 bat loci contained potentially informative retroposed elements, the sequences of which were used in BLAT searches (UCSC). We found 12 elephant, 40 armadillo, and 11 bat loci with flanks conserved in either human or dog, which were then used to design conserved PCR primers.

### Strategy IV

To find additional support for the basal placental divergence we scanned all available elephant trace sequences (≍ 11 million) for L1MB5 elements. Presence/absence of L1MB5 markers at 21,000 loci were analyzed by eye using the UCSC Server. One additional Epitheria marker was found. To test for potential conflicting markers (homoplasy), we analyzed the available ≍ 10 million trace sequences of the armadillo for presence of L1MB5 at about 24,000 loci and presence/absence in other species. There was no evidence to support afrotherians at the base of the placental tree. Searching about 2 million available European shrew *(Sorex araneus)* traces by this strategy we found 1,750 LINE1- or LTR-containing loci, within which were two additional markers confirming Laurasiatheria monophyly.

Thus, we attempted to amplify each of 237 different loci in at least one representative of the four mammalian superorders using a high-throughput PCR approach. In all the respective, investigated taxa, 28 were informative and were chosen for an expanded taxon sampling ( Table S1). The distribution of informative presence/absence markers was verified in other species by complementary sequence information retrieved from trace data available at the National Center for Biotechnology Information ( http://www.ncbi.nlm.nih.gov/BLAST/mmtrace.shtml).

### PCR amplification and sequencing

Special strategies were used for the presence/absence analyses in representatives of the superordinal clades of mammals. We designed PCR primers located in DNA regions highly conserved between human and chicken or/and dog ( Table S2). PCR reactions were performed using Phusion DNA Polymerase (New England BioLabs, Beverly, Massachusetts, United States). The first high-throughput PCR was carried out in a 96-well plate format, amplifying the sloth, nine-banded armadillo, elephant, squirrel, shrew, mole, and pangolin genomes. PCR was performed for 30 s at 98 °C followed by 35 cycles of 10 s at 98 °C, 30 s at 55 °C, and 30 s at 72 °C. Following gel-electrophoreses, those markers in which fragment size shifts indicated the presence or the absence of the embedded transposed elements, were amplified in the expanded species sampling ( Figure 1). All investigated PCR fragments were sequenced directly or purified on agarose gels, ligated into the pDrive Cloning Vector (Qiagen, Hilden, Germany) and electroporated into TOP10 cells (Invitrogen, Groningen, The Netherlands). Sequencing was performed using the Ampli Taq FS Big Dye Terminator Kit (PE Biosystems, Foster City, California, United States) and standard M13 forward and reverse primers ( Table S2).

### Statistical analyses

Statistical analysis of our data to test the validity of clade hypotheses at various nodes of the phylogenetic tree and for rejecting alternative hypotheses were carried out according to the method of Waddell et al. [ 1]. Assuming there is only one clear prior hypothesis at any given node, a minimum of three integration sites are required for a significance level of *p* < 0.04.

## Supporting Information

Dataset S1 Aligned Sequences of the 28 Phylogenetic Markers in FASTA Format (579 KB DOC).

Figure S1 Schematic Human Chromosomal Map including the Positions of Presence/Absence Markers (A) The various chromosomal locations indicate the independent integration of the 28 markers investigated. The different colors for markers refer to the clades shown in Figure 2.
(B) Presence (+) and absence (−) of all markers in the various mammalian clades. The numbers in column 1 correspond to the divergences shown in Figure 2, and lower case letters indicate the specific markers. The retroposon designations are taken from the RepeatMasker outfile and correspond to human sequences. Chr, human chromosomal location; O, outgroup (opossum). Roman numbers in columns 4–7 correspond to clades in Figure 2.
(1.8 MB JPG).

Figure S2 Computational Strategies (1.4 MB JPG).

Table S1 Detailed Presence/Absence Patterns (148 KB DOC).

Table S2 Oligonucleotides Used for PCR Amplifications (29 KB DOC).

### Accession Numbers

The GenBank ( http://www.ncbi.nlm.nih.gov/Genbank) accession numbers for the sequences discussed in this paper are DQ198489–DQ198536, DQ205239–DQ205242, DQ304437–DQ304442, and DQ317408.

## Abbreviations

kb: kilobase
LINE1: long interspersed element
LTR: long terminal repeat
