# Supplementary material for: Retroposed Elements as Archives for the Evolutionary History of Placental Mammals
Source: PLoS Biol. 2006 Mar 14;4(4):e91. doi: 10.1371/journal.pbio.0040091 (PMC1395351; doi:10.1371/journal.pbio.0040091)
Supplement: Supplementary file 1 [file pbio.0040091.sd001.doc]

## Dataset S1

# Aligned sequences of the 28 phylogenetic markers in fasta format.

# Marker 1a:

>L1MB7

--------------------------------------------------

---------------------------------------atccatagaga

cagaaagtagattagtggttgccaggggctggggggaggggggaatgggg

agtgactgctaatgggtacggggtttctttttg-gggtgatgaaaatgtt

ctgaaattagatagtgg-tgatggttgcacaac-tctgtgaatatactaa

aaa-ccactgaattgtacactttaaaagggtgaattttatggtatgtga-

attatatctc---aataaagctgttatttt-aaaaa--------------

--------------------------------------------------

--------------------------------------------------

--------------------------------------------------

------------------------

>dog

AAAACTGTCCATTTCCTTTTGCCGATCCTTTTGTCTGCTCT-GTTTAAGA

AGTTAAAACACAAG---CTTTCA-AAACACAAGGCC---atccagggaca

---aaaatactc---tggttgtcaaaggctagtggaagggagaatag-gc

aatgacagctaatgagcaaagggtttcttttta-aggtgatgaaaatgtt

ctggaattagtg---------ctgttatacaac-tagatgaatatact-a

aaagccactgaattacgcactttaaaaggatgaaaacatggtacatga--

aatatgtcttaaaa-------gaag-caaaatacccacaacatcata---

------------CAC--TAAAGAAAGTATC---AATAG--------TAAA

TCAGAACA--TGAAAG-----TAGTGA--CAACCTTCAAAGAC-AATCTG

CCAAGT--TTACCTGAATGATTGCTTTCAAGTTATCTACTCCTTCATCAA

GGCCCAACTCCTTCCTGCTTATTT

>bat4b

AAAATTGTCCATTTCCTTTTGCCGATCCTTTTGTCTGTTCT-GTTTGAGA

-GTTAAAACACAAG---CTTTCA-AAACACAAGACT---attgaaagaga

cagaag-taca-tagtggtggg--aa-------ggaagaatggggag-tg

actgactgctaatgggtaaagagattcttttta-gggtgatgaaa-tgtt

ctggaattagtg-----ctgatggttgtacaac-ttgatgaatatatt-a

aaaaccattgagttgtacacttcaaaaaggtgaa--tatgatatatg--a

attaggtcttaaaagaaaaaaaaaaacattacacatatac-tccaaaa--

------------TAGCTGTAG----GAATC---AG-AACAAGAACATAAA

---GAAA---TGGCAATGG--CAGCA----AACCTTCACAGAC-AG-CTG

CCAAGT--TTACCTGAATGAGTGCTTTCAAATTAT------CTTCATCAA

GTCCTAACTCCTTCCTGCTCATTT

>mouse

AAAACTGTCCATTTCCTTTTGCCGATCCTTTTGTCTGCTCT-GTTTGAGA

AGTTAAAATAT-AG---CTTTCAATATG-CAACACT----------gaga

ca-aag-catt-tcatcattaggaggggttggagtaaagcaccc-----c

agtgactaacaatggctac-gggcttcctctgt-ggtggtgacagctg--

-tacagcttggagcggg-----ggtggcaattc-tctgacttgtacattc

tcaaa--------------ccccaaaaagatgc--tataaattatgtgta

agtgtaccttaaaggaaaaacaaag-caaaaacaaaaaaa-tcctgaaat

a-----------CATTCCAAA--------------CAGT--GTACAAATA

ACTG------TGAGAGGGG-CCTGTGAGGGG-------------AGCCTG

CCACCT--TTACCTGGATGAGTGCTTTCAAGTTATCCACTCCTTCTTCCA

GTCCCAGCTCCTTTCTGCTCAACT

>rabbit

AGAATTGTCCATTTCCTTTTGCCGATCCTTTTGTCCGTTGCTGTTGGAGA

AGTTAAAACACAAG---CTTTCAAAAT-CCAGCAAA---atcaacagaca

cagaaaatact-tagtggttaccaggagctgaaggaaggggagagtgg-g

agggactgctaacaag-aatggatttcttcatg-gagtggtaaaaatatt

ctagga-taagacagtggtgacggctgtagaac-ttagtgactactata-

aaaactactgaa-tgtacactataaaaacaaacagctgcta-tatgag--

-tcatgtctt----gaa----gaaaa-aaaatacc---------------

------------CACCACACCACA--TATA---TGCTCCACAGAAAATAA

AG--CCAA--TTGGAATCAGAACATGAAAGTGGT------GAC-AGCCTC

CCAAGT--TTACCTGAATGAGTGCTTTCAAGTTATCCACTCCTTCATCAA

GCCCCAGTTCCTTCCTGCTCACTT

>rhesusmonkey

AAAATTGTCCATTTCCTTTTGCCGATCCTTTTGTCTGCTCT-GTTTGAGA

AGTTAAAACACAAG---CTTTCA------CAACATT---atccatagaca

--gaaggtact-tagtggatgccaggggctgga--------aaagtg--g

agtaactactaatgggta-ggagttt--ttttg-gaatggtgaaaatgtc

ctacaa-ttgg----tgttaataattgtaaaac-tttgtaaataca-tta

aaaaccaccaaactgtacactttaaaaggacaaac-ggatggtatgcga-

cttatgtcttaaaagaa----gaaaacaaaatacaa--------------

------------CATTCCAAAGAAAATATT---AGCAATAG------GAA

TCAGATCA--TTAAAG-----ATGTGG---CAAC----------AAACTG

CCAAGT--TTACCTGAATGGCTGCCTTCAGGCTATCCACGCCTTCATCAA

GCCCCAACTCCTTTCTGCTCATTT

>human

AAAATTGTCCATTTCCTTTTGCCGATCCTTTTGTCTGCTCT-GTTTGAGA

AGTTAAAACACAAG---CTTTCA------CAACATT---atccatagaca

--gaaagtacc-tagtggttgccaggggctgga--------aaagtg--g

aataactactaatgggtatggagttt--ttttg-gaatggtgaaaatgtc

ctacaa-ttgg----tgttaataattgtaaaac-tttgtaaatacactt-

aaaaccaccaaattgtacactttaaaaggacaaat-agaaggtatgcg--

gttatgtcttaaaagaa----gaaaacaaaatacaa--------------

------------CATTCCAAAGAAAATATT---AGCAGT--------AAA

TCAGATCA--TTAAAG-----ATGTGG---CAAC----------AAACTG

CCAAGT--TTACCTGAATGGCTGCCTTCAGGCTATCCACGCCTTCATCAA

GCCCCAACTCCTTTCTGCTCATTT

>chimpanzee

AAAATTGTCCATTTCCTTTTGCCGATCCTTTTGTCTGCTCT-GTTTGAGA

AGTTAAAACACAAG---CTTTCA------CAACATT---atccatagaca

--gaaagtacc-tagtggttgccaggggctgga--------aaagtg--g

agtaactactaatgggtatggagttt--ttttg-gaatggtgaaaatgtc

ctacaa-ttgg----tgttaataattgtaaaac-tttgtaaatacattt-

aaaaccaccaaattgtacactttaaaaggacaaat-agaaggtatgcg--

gttatgtcttaaaagaa----gaaaacaaaatacaa--------------

------------CATTCCAAAGAAAATATT---AGCAGT--------AAA

TCAGATCA--TTAAAG-----ATGTGG---CAAC----------AAACTG

CTAAGT--TTACCTGAATGGCTGCCTTCAGGCTATCCACGCCTTCATCAA

GCCCCAACTCCTTTCTGCTCATTT

>seacow

AAAATTGTCCATTTCCTTTTGCCGATCCTTCTGTCTGCTCT-GTTGGGAA

AGTTAAAACACAAG---CTTTT-AAAA--CAGTACT---atccatagaa-

------------tggtggccaggc---gctggggtgaggagacagtgggg

agtgactgctaatgggtacagggtttctttttg-gggtgatgaaactttt

ccagaattagtgg-----ttatgattgtacaac-tttgtgaatatc-g-a

aaatccaatgaatagtacaatttaaaagggtgaa-ttaggatatgtga--

-attggtcttaa-------aaaaaaacaaagaa--------tccacaaca

ctatacata---taCTCCAAATAAAATAGG---AGTAAT------GGGAA

TCAGAACA--TGAAAA-----AAGTGG-----------------TGGC--

--------------------------------------------------

------------------------

>elephant

AAAATTGTCCATTTCCTTTTGCCGATCCTTTTGTCTGCTCT-GTTTGGAA

AGTTAAAGCACAAG---GTTTCA-AAA--CAGCACT---atccacaa---

-------------agtggtggccaggggctggaatgaggggatcatgggg

agtgactggtaatgggtacagggtttctttttg--ggtaatgagaatgtt

ccagaattagtgg-----tgatggttgtacaac-tttgtgaatttatg-a

aaatccaatgaatagtacactttaaaagg-tgaatatatagtatgtga--

-actgatcttataaaaaaaaggaaaaaaaaaaaacaaacaatccacaacg

ctacactta---tACTCCAAAGAAAACAGG---AGCAAT------GGGAA

TCAGAACA--TGAAGG-----AGCTGGCGGCAGCCTTCACAGACAACACG

CCAAGT--TTACCTGGATGGCCACTTTCAAATTATCCACCCCTTCACTGA

GCCCCAACTCCTTCCTGCTTCTTT

>sloth

AAAACTGTCTATTTCCTTTTGTCGATCCTTTTGTCTGCTCT-GTTTGAGA

AATTAAAACACAAG---CTTTCA-AAACACAACATT---gtccataaaga

caaaaagtacattagtggttaccagggcctaagggagggg-agaatgg-a

aatgattgctaatgggt-cagggtttcttctggggggcgatgaaactatt

ctggaattagtag-----tgattcacttc--------gtgaatacact-a

aaaaccactgaattatacactttaaaaggatgaatatatgatac-tga~~

attatatcttaaaaaattaaaacaaaaaga-tacccataacaccatacag

gtaca-------CACTCCAAAGAAAATATT---AGCAAC------AAGAA

TCAGAAAA--TGAAAG--------TGGCAGCAACCTTCAAAGACAACATG

GTAAGA--TCACCTGAATTGCTGCTTTCAAATTATCCATCCCTTCATCAA

GTCCCAACTCCTTCCTGCTGGTTT

>armadillo

AAAACTGTCCATTTCCTTTTGCCGATCCTTTTGTCTGCTCT-GTTTGAGA

AATTAAAACATAAG---CTT-CA-AAACACAACACT---gcccataaaga

cataaagtacattagtggttgccaggggctgaggggaggggagattggaa

agtgactgctaatgggtacagggtttcttttgc-gggccttgaaaatatt

ctggagttagtaa-----tgatggttgtaaaacttttgtggatataatta

aaaaccactgaattatatacatctaaagtatgaatatgtgatatgtga~~

attatatcttaaaaaaaa----------ga-aacccataacgttatatgt

a-----------TACTCCAAAGAATATATT---AGCAAT-----AGGAAA

TTAGAACA--TGAAAG-----AAATGGTGGCAACCTTCATaGACAACATG

CCAAGA--TTACCTGAATGGCTGCTTTCAAGTTATCCATCCCTTCATCAA

GGCCCAACTCCTTCATGCTCATTT

>opossum

AAAACTGTCCATTTCTTTTTCCCGATCCTTTTGTCTGCTCT-GTTGAAAG

AATCAAAGCAACAG------------------------------------

--------------------------------------------------

--------------------------------------------------

--------------------------------------------------

--------------------------------------------------

--------------------------------------------------

-------------ACCCCTAAGAAAAGAAC---TGGGGGGAAGGGGGAGC

TCCCAGCTCTTGGAACCCACAAGACCCA--CAACACCT------AGTATG

CCAAGTGCTCACCTGTATGAGTGCTTTTAGGTCACCTGATCCCTCCCCCA

GACCCAGCTCCTTTCTGGTCTTTT

Marker 1b:

>L1ME4a

--------------------------------------------------

--------------------------------------------------

--------ctggaaggaaatacaccaaaa---tgttaacagtggttatct

ctgggtggtgggattatgggtgattt--ttattttcttttttctttgtat

tttctgtattttccaaattttctacaatgaacatgtattacttttataat

cag----aaaaaaaa-----------------------------------

--------------------------------------------------

--------------------------------------------------

--------------------------------------------------

--------------------------------------------------

--------------------------------------------------

--------------

>cow

TTCCAAGAATATTTGACTACTCTCCTTTAGATCCAACACAAGATTTCAAC

ACACAGATGTAAGTGACCTG----CTGTAGTTTT----------------

---------taaga--aaagtttatctgaaatagtgctcagagtggttat

ctctgggttgcagaattaaaaacgattctttgctttactgtct-------

---gtattgtttaaaagtattccaaaataagcatgtgttgcttttgtaaa

aaggaag--aaatta---GTAAAATGAT---AAAGTACTGAGAA---TTT

GAAAA--------------TGGAATCAG-TATCAAGCAGAATCA------

---AGTAAGTTATTTGTGAGTAATTGAGATACAGTGTTTCACTAAAAGCA

GCTGTAGTTACTGAAATTTGTAGATAATTTTGGCA--TCTCTGAGGGGAA

GCAACAA-----TGACCTTTTTGTCTCTTT--------------------

---CT--CCCTCCTGTCCAGGACTAAGTTAGGACATGGCCTTCTCTCAGG

CCAGTATTCAAAAC

>dog

TTCCAAGAATATTTGACTACTCTCCTTTAGATCCAACACAGGACTTCAAC

ACACAGATGTAAGTG-CTGG---TAAAAAATTTT----------------

---------taagg--aaaatgtccagt-aaatgttcataatggttattg

ctgggttgcagaattaaaggtacatctcttttcttttttgttttctgtat

t-----------agaagtgttctaaaaagagcaggtgttacttttagaag

aag------ataata---GTTCTTTAT----AAAGTACTGAGGGTAGTTT

GAAA---------------TGGCATCAGTTATCAGGAAGAATCA------

---AATAAGTTATTTTTGAGTAATCAAGATAAAATGTTCCACC-------

----------TAAAAGCATTCATAGATGATGTTAATGTCTCTATGGGGAA

ACAACAA-----TGACCTTTTTGTTT--GTCTTT----------------

---CT--CCCTCCTGTCCAGGACTAAGTTAGGACATGGCCTTCTCTCAGG

CCAGTATTCAAAAC

>mouse

TCCCAAGGATATTTGACTACTCACCGTTAGATCCAACGCAGGACTTCAAC

ACACAAATGTAAGTGCCCAG---TGCTGTGTTTT----------------

-----------------aagtttctggtggggtgtttgcattggttactg

ctgagttg--------aaggcaattcttttttcttctttattttctgtat

tttt----------aagtgttctagaatgggtgtgtgttacctttataag

aagagattaataa-a---GTTAT--------AAAGTACTGAGGAAACTTT

GACA--------------GTGGAGTCAG-AAT------------------

---ACCAAGCTGTTTGTGAGGAATCCGGATAGAGA--------------G

CAGCCCCGC-TACAAGAACTCATGGGTCATCTTCACATCTCTTTGG-GAA

ACAGCAG-----TGGCCTCTTTATT---GTTTGT----------------

---CT--CTCTCCTGGCTAGGACTAAGTTGGGACATGGCCTCCTCTCTGG

CCAGTACTCGAAGC

>rat

TCCCAAGGATATTTGACTACTCACCATTAGATCCAACGCAGGACTTCAAC

ACACAGATGTAAGTGCCCAG---TGCTGTGTTTT----------------

-----------------aagtttctggtggggtgtttgcattggttactg

ctgagttg--------aaggcaattcttttttcttctttattttctgtat

tttt----------aagtgttctagaatgggtgtgtgttacctttataag

aag------ataa-a---GTTAT--------TAAATTCTGAGGAAACTTT

GAAA--------------GTGAAGTCAG-AAT------------------

---ACCAAGCCATTTGTGAGGAATCCGGATAGAGA--------------G

GAGCCCCGC-TACAAGAACTCATGGGTCATCTTCACATCTCTGTGG-GAA

AGAGCGG-----TGGCCTCTTTATT---GTCTGT----------------

---CT--CTCTCCTGCCTAGGACTAAGTTGGGACATGGCCTCCTCTCTGG

CCAGTACTCGAAGC

>rhesusmonkey

TTCCCAGAATATTTGACTACTCGCCTTTAGATCCAACACAAGATTTCAAC

ACACAGATGTAAGTG-GCAG---AATTTTTTTTTTTTTTTT---------

--------ttacga--aaagcatccaataaaatgttcatggtggttatct

ctgggttgcaaaattaaaggcaattc---------------tttatgttt

tttttttttttt-aaagtgttccaaaatgagaatgtgttacttttataag

aag------ataa-a---GTTATCTAC----AAAATACTGAAGAAAATTT

GAAA--------------CTGGTATCAG-TGTGAAGCAGAATCAAA----

-TAAATAAGTTATTTGTGTGTAATCCACATCAAATGCTCCACTTAAC-AG

CAGCTGTACTTATTGATATTCATAGATAAATGTAATATTGCTATGGAAAA

ACAACAA-----TGACCTTTTTGTTTTTGTTT------------------

---CT--CTGTCCTGGCCAGGACTAAGTTAGGACATGGCCTTCTGTCAGG

CCAGTATTCAAAGC

>human

TTCCCAGAATATTTGACTACTCGCCTTTAGATCCAACACAAGATTTCAAC

ACACAGATGTAAGTG-CCAG---ATTTGTATTTTTTTTTTT---------

--------ctagaa--aaagcatccaataaaatgttcatagtggatatct

ctgggttgcaaaattaaaggcaattc---------------ttcatgtat

ttttaa------aaaagtgttccaaaatgagaatgtgttacttttataag

aag------ataa-a---GTTATCTAC----AAAGGACTGAAGAAAATTT

GAAA--------------CTGGTATCAG-TATGAAGCAGAATCAAA----

-TAAATAAGTTATTTGTGTGTAACGCAGATCAAATGCTCCACCTAAC-AG

CAGTTGTACTTATTGATATTCATAGATAAATTTAATATTACTATGGAAAA

ACAACAA-----TGACCTTTTTGTTTCTGTTT------------------

---CT--CTGTCCTGGCCAGGACTAAGTTAGGACATGGCCTTCTCTCAGG

CCAGTATTCAAAGC

>chimpanzee

TTCCCAGAATATTTGACTACTCGCCTTTAGATCCAACACAAGATTTCAAC

ACACAGATGTAAGTG-CCAG---ATTTGTATTTTTTTTTTT---------

--------ctagaa--aaagcatccaataaaatgttcatagtggttatct

ctgggttgcaaaattaaaggcaattc---------------ttcatgtat

ttttaa------aaaagtgttccaaaatgagaatgtgttacttttataag

aag------ataa-a---GTTATCTAC----AAAGGACTGAAGAAAATTT

GAAA--------------CTGGTATCAG-TATGAAGCAGAATCAAA----

-TAAATAAGTTATTTGTGTGTAACGCAGATCAAATGCTCCACCTAAC-AG

CAGTTGTACTTATTGATATTCATAGATAAATTTAATATTACTATGGAAAA

ACAACAA-----TGACCTTTTTGTTTTTGTTT------------------

---CT--CTGTCCTGGCCAGGACTAAGTTAGGACATGGCCTTCTCTCAGG

CCAGTATTCAAAGC

>elephant

TTCCAAGAATATATGACTACTCTCCTTTAGATCCAACGCAGGATTTCAAC

ACACAGATGTAAGTG-CCAG---ATGGTTTTTTTTTTTTTTT--------

---------ttttt--aaagaatccactaaagcattcatagtaattatct

ctggtttgcagaattaaagacaattc------------tcttttctgtgt

tttttg------aaaagttttctaaaatgagcatgtgttac-tttaacaa

gaagaaattaataaa---GTTATCTTC----AAAGTGCTGAGGAAAATTT

GAAA--------------AATTT--CAG-AATCAAATAAATGAA------

---ACCA-GTTATTTGTGAGTCATCGAGATAAA-TGTTCTACCTAAA-AG

CAGCTGTACTTACTGAAATTCATAAACAATGCTAACATCTCTAAGGAAAA

AAAAAAAAACAATGACCTGTTTGTTT--GTTT------------------

---CT--CCCTGCTGTCCAGGACTAAGTTAGGACACGGCCTTCTCTCAGG

CCAGTATTCAAAAC

>armadillo

TTCCGAGAATATTTGACTACTCTCCTTTAGACCCGACACAAGATTTCAAC

ACACAGATGTAAGTG-CCGT---CTTGCACTGTATTTTTGTT---atgtt

ttatatttcttttt-ttaaaaaaa--ggattcagggaaatgttcatagtg

gttaattgcagaattaaaggcaattc------------tctttttttctt

taatctt-----aaaactcttataaac-aattatatgttaacttttacaa

gaggaacttttaaaa---ATTATTTTT----AAAGTACAAAGAGAAATTT

GAAA--------------AAGAAATCAAGTGTCAAACAGAATCAAATAAG

TGAAAGAAGTTATTTGTGAATAACTGGAATAGAATGTTCTATCTAAA-AG

GAGCTGTACTTATTGAAATTCATAGGTAGTTTTAATATCGCTAAGGGAAA

ACAACAA-----TGACCTTTTTGTTTTTGTT-------------------

---CT-TCCCTCCTGCCCAGGACTAAGTTAGGACATGGCCTTCTCTCAGG

CCAGTATTCAAAAC

>opossum

-----AGAATATTTGACTACTCTCCTCTAGACCCAACACAGGATTTCAAC

ACACAGATGTAAGTG-CCAG------------------------------

--------------------------------------------------

--------------------------------------------------

--------------------------------------------------

------------------GGCTCCATT----GGGAGGGGAGAAAAGTATT

GAGTAGAACTGGGAAAATTCCCTAGCAA-TCAGAAATAAATGACC-----

-AAAATAAACTTTTGTCATGACTCTTGAGACTTA--------GATAATGG

GACAGCCATTTTTCTGGCTAAAATGGCAAATGTAGTGGCAATTTGGGGAG

AGGA--------TGACAACTTGGAAGTTCATTGTTTTGTTTATTTGTTTG

TTTCTCTGCTTTATGCC-AGGGCTAAGTTAGGACATGGCCTTCTCTCAGG

CCAATATTCTAAAC

Marker 1c:

>L1MB8

--------------------------------------------aaagtg

gaaacaacccaaatgtccatcaactgatgaatggataaataaaatgtggc

atatccatacaatggaatatta----ttc-agc--aataaaaaggaatga

agtactgatacatgct-acaacatggatgaaccttg-aaaacattac--g

ctaagtgaaagaag-ccagtcacaaaaga--ccacatattgtatgattcc

atttatatgaaatgtccagaataggcaaatccatagagacagaaagtaga

ttagtggttgcctggggctg------------------------------

--------------------------------------------------

--------------------------------------------------

--------------------------------------------------

--------------------------------------------------

--------------------------------------------------

--------------------------------------------------

--------------------------------------------------

------gggggaagggggaatggggagtgactgctaatgggt--------

--------------------------------------------------

--------------------------------------------------

--------------------------------------------------

--------------------------------------------------

---------------------------------acg-gggtttcttttgg

gggtgatgaaaatgttctaaaa-tgattgtggtgatggttgcacaactct

gtgaatatactaaaaaccattgaattgtacac----tttaaa--------

---tgggtgaattgtatggtatgtgaattatatctcaataaagctattaa

aaaa----------------------------------------------

--------------------------------------------------

--------------------------------------------------

--------------------------------------------------

------------------------------------

>dog

TTGTCTGTGGGATGGACCAGCTTCC---AAGAGTACTAGCG---aggggg

ggaa-aacccaaatgtccatcagcagatgagaggcaaaagctaacatggc

gtgaccttgtgtgttggggggtac---tcctcagttataaaagggaatga

agttctgattcaggct-ataacatggatcatggttcttgaaggcatg---

--aagt-aaagaag-tcagacacaaataa--ctacatatgatatgattct

acttaagggaaacatccagaacagggaaa-ttatagagacagaagacaga

-------ttgcttagggctg------------------------------

--------------------------------------------------

--------------------------------------------------

--------------------------------------------------

--------------------------------------------------

--------------------------------------------------

--------------------------------------------------

--------------------------------------------------

------tggggatagggaatgggggggtgatggc----------------

--------------------------------------------------

--------------------------------------------------

--------------------------------------------------

--------------------------------------------------

---------------------------------aca-gggtttcttttgg

aactgataaaaccattctaaa-tttgactgtcgttgt-----acaattct

gcgaatatgctaaaaaccaacaaa-tctgcat----tttaaa--------

----tgagtgaatggtatag-tgtgaaaatta-tctcagtaaggctgtta

aa---------------------GGAAGAGTGCTGGGGAG---AGGCAGA

GGCAGACAAGAGTTCGCGTGAATGGA-GGTCAGGCTG-GA-GAGG----T

CTGGGGAGGGGGA----------GGCCCTCTGCCTTCATGA-CACCTACC

ACCTCCATGACGTGCGCGCTCCACTTGG-ATAAAAGCTGCAGGC-CCCGC

AGGGCCAGGTCGAAGAGCTCACGGTACTCCTCGTCT

>mouse

TTGTCTGTGGGGTGAACCAGCTTCC---AAGAGTACTGGAG---ggagaa

gaaac-agccaa-cacccatcaactga-ggagtggtaaacaaagcagtct

agcaaca-ccagggagca--ctaatgggatgt-ggggtaaacatactgac

tcgtgcaggatgggg-----------ctggaccctgaaaacacaat----

-----gtaagaaactccagacacaaaacaccacacgagctctatgagtct

atatatggtgaatctctaaaactgggatgc--atagagtcagaataccga

------------------ag------------------------------

--------------------------------------------------

--------------------------------------------------

--------------------------------------------------

--------------------------------------------------

--------------------------------------------------

--------------------------------------------------

--------------------------------------------------

------gct-aaac-gggcagggtgacagatgtgggcttttggt-tgtga

gcctagccttaacggctgagtcatctctccagccccagatgtgggttca-

------tttatgagatttacttttaatattatttatg--------tttct

atatgtctgtggggaagtgcacgtgagtgtgggtgtcaca----------

-gaagccagacgcaaactatctactgtgctggggttacaagaggttgtga

gccaatt--aatgttggtgctgggtccc-----aca-tgtgatatttttg

ttgtttaagtgatg----aaactatt-----------------aatttaa

ggtcatatacttctgta-aat---------ata----ttaaatgcaggtt

tcagttggg------taag--ta-tggtatatggattttta----tgttg

aaaga--------------------CAGAGTGCCAACTAG---AGGCAGA

TGCCCA----------------TGGA-TGCCTTGTGAGT--CAGG----C

CAGTGTGA---------------GGCCTGGCACC-TCAGGA-CACTTACC

ACCTCCATGACATGGGCACTCCACTTGG-ATAGAAGCTGCAGGC-CTCGC

AGGGCCAGGTCAAAAAGCTCCCGGTACTCTTCATCC

>rat

TTGTCTGTGGGGTGAACCAGCTTCC---AAGAGTACTGGGG---agagaa

gcaac-agcc--actcccatcaactga-ggaaccgtaaacaaaacggtct

agcaata-ccagggaacacgctaacgggatgtgggggtgaacataaacag

aataccaaag----------------------------------------

--------------------------------------------------

--------------------------------------------------

--------------------------------------------------

--------------------------------------------------

--------------------------------------------------

--------------------------------------------------

--------------------------------------------------

--------------------------------------------------

--------------------------------------------------

--------------------------------------------------

------gctgaatcaaggcagggtgatggatgtgggttttt---------

-----------------------------------------------taa

aaatattttttaaggcttacttttattgttgtttatgtgtctatatgtct

gtctgtctgtggggatgtgcccatgagtgtgggtgtcacagaagccagac

cgaagccggaggtgtacaatctgctgtgctgggcttacaagaggttggga

accaaca--aaaattggtgctgggtcccag---acgtagtttttgtttgt

ctgttgaa-tgatg----aaaatatt---------------cttaactga

ggtcataccattctgta-aat---------acc---ctcag-tgtacgtt

tcagttaggtgagt-agtg-----tggtatatagattttca----tgttg

aaaga--------------------CAGAATTCCAACGAG---AGGCGGA

CGTCCA----------------TGGA-TGCCTTGTGAAT--CAGG----T

CAGCGTGG---------------GGCCTGCCACC-TCAGGA-CACTCACC

ACCTCCATGACGTGGGCACTCCACTTGG-ATAGCAGCTGCAGGC-CCCGC

AAGGCCAGGTCAAAAAGCTCCCGGTACTCTTCATCG

>rhesusmonkey

TTGTCTGTGGGATGAACCAGCTTCC---AAGAGTACTGGGG---agagtg

gaaacaaaccaaatgtccatcaactgatgaacggacaaacagaatgtgga

ggatccatgcaatggaatatta-----ttcagctga-aaaaaaggaataa

agtactaattcatgct-acaacat-gaaaaaccttgaaagtatgcaa---

--aa-taaaagaag-cctgacacaaaaag--ccacatatcctatgatttt

atttatgtgaaacatccagaatagggaaacctatagaggtagaaagcaga

-------ttgctcagggcta------------------------------

--------------------------------------------------

--------------------------------------------------

--------------------------------------------------

--------------------------------------------------

--------------------------------------------------

--------------------------------------------------

--------------------------------------------------

------gggagatgggaggggatataac----------------------

--------------------------------------------------

--------------------------------------------------

--------------------------------------------------

--------------------------------------------------

---------------------------------aca-gggcttctttttg

aaatgatgaaaatgttctaaatttgactgtggctat------acaactct

gcggatatactaaaaaccgacgaa-ctta-cac---tttaaa--------

----tgggtaaatggtacggcatgtgaattataactccataaatctgtta

aaaa---------------------GACAGCGCTAGGGGG---AGATGGA

GG--------------------CAGA-TTCCAAAGAAAGA-GAGG----T

CAGTGTGA---------------GGCCCTCTACC-CGGAGA-CACCTACC

ACCTCCATGACGTGGGCGCTCCACTTGG-ATAGAAGCTGCAGAC-CCCGC

AGGGCTAAGTCAAAGAGCTCGCGATACTCCTCGTCT

>human

TTGTCTGTGGGATGAACCAGCTTCC---AAGAGTACTGGGG---agagtg

ggaacaaaccaaacgtccatcaactgatgaacggacaaacagaatgtggt

ggatccatgcaatggaatatta-----ttcagctgg-aaaaaaggaataa

agtactgattcatgct-acaacat-gaaaaaccttgtaagtatgcaa---

--aag-aaaagaag-cctgatacaaaaga--ccacatatcatatgattct

atttatgtgaaacatccggaatagggaaacctatagaggtagaaagcaga

-------ttgctcagggcta------------------------------

--------------------------------------------------

--------------------------------------------------

--------------------------------------------------

--------------------------------------------------

--------------------------------------------------

--------------------------------------------------

--------------------------------------------------

------gggagatgggaggggagataac----------------------

--------------------------------------------------

--------------------------------------------------

--------------------------------------------------

--------------------------------------------------

---------------------------------aca-gggtttctttttg

aagtcaagaaaatgttctaaatttgactgtggctat------acaactct

gcggatatactaaaaaccaatgaa-ctta-tac---tttaaa--------

----tgggtaaatcgtatggtacgtgaattgtaactccgtaaatctgtta

aaaa---------------------GAGAGTGCTAGGGGG---AGATGGA

GG--------------------CAGA-TTCCAAGGAAAGA-GAGG----T

CAGTGTGA---------------GGCCCTCTACC-CAGAGA-CACCTACC

ACCTCCATGACGTGGGCGCTCCACTTGG-ATAGAAGCTGCAGAC-CCCGC

AGGGCTAGGTCGAAGAGCTCGCGATACTCCTCGTCT

>chimpanzee

TTGTCTGTGGGATGAACCAGCTTCC---AAGAGTACTGGGG---agagtg

ggaacaaaccaaacgtccatcaactgatgaacggacaaacagaatgtggt

ggatccatgcaatggaatatta-----ttcagctgg-aaaaaaggaataa

agtactgattcatgct-acaacat-gaaaaaccttgaaagtatgcaa---

--aag-aaaagaag-cctgatataaaaga--ccacatatcatatgattct

atttatgtgaaacatccagaatagggaaacctatagaggtagaaagcaga

-------ttgctcagggcta------------------------------

--------------------------------------------------

--------------------------------------------------

--------------------------------------------------

--------------------------------------------------

--------------------------------------------------

--------------------------------------------------

--------------------------------------------------

------gggagatgggaggggagataac----------------------

--------------------------------------------------

--------------------------------------------------

--------------------------------------------------

--------------------------------------------------

---------------------------------aca-gggtttctttttg

aagtgatgaaaatgttctaaa-tttgactgtggctat-----acaactct

gcggatatactaaaaaccaatgaa-ctta-cac---tttaaa--------

----tgggtaaatggtatggcacgtgaactgtaactccgtaaatctgtta

aaaa---------------------GAGAGTGCTAGGGGG---AGATGGA

GG--------------------CAGA-TTCCAAGGAAAGA-GAGG----T

CAGTGTGA---------------GGCCCTCTATC-CAGAGA-CACCTACC

ACCTCCATGACGTGGGCGCTCCACTTGG-ATAGAAGCTGCAGAC-CCCGC

AGGGCTAAGTCGAAGAGCTCGCGATACTCCTCGTCT

>tenrec

TTGTCAGTGGGGTGAACCAGCTTCC---AGGAGTACTAGGG---ggagta

gaaacaacccacctgtccatccaccgatgaagagataacccaaatgtggt

acatgcatacaaaggaacc--------tgaagcactgaccaatgtgtgtt

acaattagaa--tgc--acctcaaaaaacattagtctaact-aaaataag

ccagttac-aaaga-ccacagac-tatg-ttatccaagtctatgaaagat

caggagtaggaaatc-gatgagacag------atag----------t-ga

tcagtgg-tgc-taggtcta-ggaggagccttggggataccatcagtaca

gtgctcaactgctaacccaaatgccaatggtttaatccaccagtggctcc

atggatgaaaagacctagcaatctgggaccataaagcttacagcctagga

aaccctacaggagagttctacttggccaaaagcagaccaaagagaaaccc

ctgccttcgagtcgattctgactcacagcgaccatacgacagagcagaat

cactctgtagggcacccaaggctgtaagtcttcatggaatgattgtctca

tctttctctcactgtgtgacgggtgggttcagactgcttacctggccgtg

agcagcccagagcctaacaactacgctgttgactcgatggcacaccacaa

ggccc-gggggagaggacgtaggaga-tggtagtgaatggg---------

--------------------------------------------------

--------------------------------------------------

--------------------------------------------------

--------------------------------------------------

-------------------------------caata-aagattc--ttgg

t--aacag-aaatgttctaaaattgactgtggtgatagttgtataactct

gtg----------aagatactgaa-ttgcacac---ttcaaa--------

----tgggtgaagggtatggctcagt---tgtatggttaaaaataaataa

acacgca-------------------------GTGGGGAG---CGATGGC

G---------------------TGAA-AACC-AGG----CACAGCGGGCT

CAGTGTGGAGGGGA---------------------CGAAGA-TACCCACC

ACCTCCATGACATGGGCGCTCCACTTGGTACAACAGCTGCAGGCTCTCGC

AGGGCCAGGTCGAGGAGCTCGCGGTACTTCTCGTCA

>armadillo

TTGTCTGTGGGGTGAACCAGCTTCC---AAGAGTACTAGAA---agcacg

gaaacaacccaaatgtccgtcaactgatg-aa---tggctagaaggtggt

gtagccctacaatgg--cataa-----cgttcagccataaaaaaaagaaa

caagttgtgattcatgcacaacttggatgaaccttgacagcattac---g

ccaagggaaaaaag-cccgtcacaaaaga----cca----cctg--ttat

atttatataaaatatccagaacaggcaaatctatatgaacagaaaataga

ttcgaggttgtctaggactg------------------------------

--------------------------------------------------

--------------------------------------------------

--------------------------------------------------

--------------------------------------------------

--------------------------------------------------

--------------------------------------------------

--------------------------------------------------

------cagaggacgggaggtagaagggtgataggtaaagggt-------

--------------------------------------------------

--------------------------------------------------

--------------------------------------------------

--------------------------------------------------

---------------------------------atg-gaacttctttgga

a-gtggtgaaattgttctaaaa-tggactgaggtgctagttgcacaactc

agttaatataataaaaccactaaa-ttgcacac---tttaaa--------

----tgggtgaa-----ctgtatgtgagttatatctcagtaaattttttt

ttttt---------tttaa---GAAAAGAACACTGGT------------C

AAAGGCAATGGCAGAAA-GAGTCCACGTGAACAGGGGC---CAGTG--TG

GAGGGGGGCAGCAAGA--------GCCCCTCAC-CACGGGG-CACCTACC

ACCTCCATGACGTGGGCACTCCACTTGG-ATAGAAGCTGCAGGC-CTCGC

AAGGCCAGGTCGAAGAGTTCGCGGTACTCCTCGTCT

>sloth

TTGTCTGTGGGATGAACCAGCTTCC---AAGAGTACTGGGA---ataata

gaaacaatgcaaatgtccaccagctgatg-ga---tggataagatgtgtt

ctatccatacagtggtacat-------tcttcagccataaaaaagaataa

aatactgattcatgc--acaacatgggtgaaccttgaaaacattacacta

--aagg-aaagaag-ctagtcacaaaaga--ctacatagtaaatgattcc

atttatatgaaatatccagaataggcaaatctatagggacagcaaataga

ttagtggttgcttaggactg------------------------------

--------------------------------------------------

--------------------------------------------------

--------------------------------------------------

--------------------------------------------------

--------------------------------------------------

--------------------------------------------------

--------------------------------------------------

------cagaggattgagggaaaggagggtgatagctaaagcgt------

--------------------------------------------------

--------------------------------------------------

--------------------------------------------------

--------------------------------------------------

---------------------------------aca-aatcttctttcaa

aagtgatgaaaatgttctaaaa-ctgactgtggtgatggttgcacaactc

aattgatataataaaatcactgaa-ttacacac---tttaat--------

------ggagaattgtactgtatgtgagttctagctcaataaatcttttt

gttgttgctgtttgtttaa------GAGCGTACT---GAG---CAGAGGC

AGG-------------------CCGA-GTCCACGTG-----CAGGGGGTT

GGGGGGG-CAGG----GAGCTGGGT-CCTTCACCCTTGGGG-CACCTACC

ACCTCCATGACGTGGGCACTCCACTTGG-ATAAAAGCTGCAGGC------

------------------------------------

>opossum

TTGTCAGTGGGATGAACCAGCTTCC-------------------------

--------------------------------------------------

--------------------------------------------------

--------------------------------------------------

--------------------------------------------------

--------------------------------------------------

--------------------------------------------------

--------------------------------------------------

--------------------------------------------------

--------------------------------------------------

--------------------------------------------------

--------------------------------------------------

--------------------------------------------------

--------------------------------------------------

--------------------------------------------------

--------------------------------------------------

--------------------------------------------------

--------------------------------------------------

--------------------------------------------------

--------------------------------------------------

--------------------------------------------------

--------------------------------------------------

--------------------------------------------------

-------------------------AGGAGTACTGGGGAG---AAGGAGA

CCAAAGA----------TGAATGGCAGACTGCTTTTGTAGACAGGA-CAG

GAGGGGGACACTGTTT-GCCTGGGG--CTGGAG--TTGCGGTCACTTACC

ACCTCCATGACATGGGCACTCCACTTGG-ATAGCAGCTGGAGCC-CTCGC

AAAGCCAGATCGAAGAGTTCCCTGTACTCTTCATCG

Marker 1d:

>L1MB8

--------------------------------------------------

--------------------------------------------------

--------------------------------------------------

-----------------------------------atcaactgatgaatg

gataaa---------taaaatgtggcatatccatacaa-tggaatattat

tcagcaataaaaaggaatgaagtactgatacatgctacaacatggatgaa

ccttgaaaacattacgctaagtgaaagaagcca----gtcacaaaagacc

acatattgtatgattccatttatatgaaatgtccagaataggcaaatcca

tagagacagaaagtagattagtggttgcctggggctggggggaaggggga

a----------tggggagtgactgctaatgggtacggggtttcttttggg

ggtgatgaaaatgttctaaaat-gattgtggtgatggttgcacaactctg

tg----------------------------------------aatata--

c-taaaaa---------ccattgaattg----------------------

--------------------------------------------------

-----------------------------------tacactttaaatggg

tgaattgtatggtatg-tgaattatatctcaataaagctattaaaaa---

--------------------------------------------------

--------------------------------------------------

--------------

>cow

CACCCACTCACTGAATATCAGGAATGTCCACTTTGGGGATGCCGCTGAGT

ACACCTTTGTGGCTGGAAAAGCTACCTCGACAGCCACTCTTTATGTGGAA

GGTAT-GGTTTCATAAATGTCATATCCATAAAAAG------AAATGTCAA

ACACACTACATAAAAA-------TGTTAGTGC---gtcagctaatgaatg

aa-------------taaataaggaatatcta-tgcaa-tgaaatattac

ttagtaataataaggaag----tactgataaatattacaacatgaatgaa

ccttaaccttataccaagtgaaagaag---ctagttataaagaag----c

acatattatatgaatccagttacaggaagagttcagaatgtgcaaatcaa

tagagacatagatttgt-----gcctaagtgtcc---gggaagagcagtg

cacggggtc--tggaaggttatgactaagagatacgtggtttcttttggg

agtaatgagtatgccttgaaattgattatggtgatggctgtacaattct-

-----------------------------------------aaata---t

actgaaag---------cccctgaccta----------------------

--------------------------------------------------

-------------------------------------tgctttcagtggg

tgaattgtatagtatg-t-aatcatatcttggcaaagctgttagaaa---

GGTTAATTC---TTTTAC-ATGCTATCCTGCAGCTCGTCATATAGAATTT

AGGAAGCACATTAAGGACATTAAGGTTCTCGAGAAGAAGCGAGCCATGTT

TGAATGTGAAGTTT

>dog

TACGCACTCACTGAACATCAGGAACGTTCACTTTGGGGATGCAGCTGAGT

ACACCTTTGTGGCTGGAAAAGCTACATCAACAGCCACCCTTTATGTGGAA

GGTAT-GGTTTCACAAATGTCTTATCCATAAAAAT------AAATGTCAG

ACGCACTATATAAAATACT----AAGTAGTGC---attcattaatgaa--

---------------taaaatgtggtgtat--gtttaa-tgaaatattg-

ttagcaataaaaagcaag----tgctgata-atgccacaacgtggatgaa

ccttaaaaactttatgctaagtgaaagaagccagtcccaaaggac----c

acatatggtatgattccatttataggaaatgtc-ggaatacacaaatctg

tagagatgtaga-ttgg----ggcctaaatctg---aggggagaccataa

gagg---gatctggagggtggtggctaaggggtggatgg--tctttcggg

agtaatgagaatattctaaccttgattgaggtgatggttagagggtgatg

gctaaggggtgtgtggtctttcgggggtagtgcacaactctgccta---c

actgaaagg---------ccctgaattg----------------------

--------------------------------------------------

-----------------------------------agtgctttgcatggg

tggattatgcactctg-tgagt--tatgtct-taaaactattagaaa---

GGTTCATTT---TTGTAC-ATACTGTGTTCCAGCTCGTCATATAGAATTT

AGGAAACACATTAAGGACATTAAGGTTCTTGAGAAGAAGCGTGCCATGTT

TGAATGTGAAGTTT

>bat4b

CACCCACTCCCTGAACATCAGGAACGTTCACTTTGGAGATGCTGCTGATT

ACACCTTTGTGGCCGGAAAGGCTACTTCAACAGCGACCCTTTATGTGGAA

GGTAT-GGCTTCATAGGTGTCCTGTCCATAAAAAT-------AATGTCAA

ACACACTGTAGAAAATGTT--------AGTGC---ataatttggtgaatg

gataat---------tgaaatgtggtatatccatgcta-tgaaatattat

ccagcaataaaaaggaac----tactggtacatgctacagcatgaatgaa

ccctgaacactttatgctgcatgaaa-aggccagtcaccaaggat----t

aaattttgtatgatgtcatttacatgaaatcttcaggatacacacatcta

tagagatataaa-ttgg------cctacgtctg---aggagaaacagtaa

tagg---agtgtgaggggttatggataagaggctcatagtttctttctag

ggtaactagaatgttctaaaattgattgtggtgatggttgcacaactctg

------------------------------------------aatg---t

acttaaag---------ccaatgaatca----------------------

--------------------------------------------------

-----------------------------------tatactgaaa-tggg

tgaattttatagtatg-tgagttatgtcttcataaagctatgaga-----

GGTTAATTT----TATAC-ATGCTGTCTTCTAGCTCGTCATATAGCATTT

CCTAAACACATTAGGGACATTAAGGTGCTCGAGAGCATGCGAGCCATGTT

TGAATGTGAGGTTT

>rat

GACACACTCTCTGAACATCAGGAATGTCCACTTCGGAGATGCTGCGGATT

ACACTTTTGTGGCTGGGAAAGCCACATCGACGGCCACCCTTTATGTGGAA

GGTAT-GAATTCCTAGATGT---AGCCGTAGGGGGTA----TGAAGTCGA

A-----TATACCACGTGAAG---TATTAATCT---ttcgattaatgaatg

gataaa---------taaagtgaggtatgtccatccaatttgggtggtaa

ttggaaattaaaaggaat----taccagcacat-----------------

-------------atgagaaatgatagaggccagccacagagcaa----c

acatgctgtatgagtccatttatatgaagtggccctgatacacaactcta

tcgagacataga----tcattagcttctgtcta---gcaagaggtcatgg

gaagt--ggtcttgcatatgatgcctaagaactgtctgatattttctggg

aaaaattaaatggttttaa----aattatggtgatgcttatataat----

-------------------------------------------atata-c

catgaaag---------ccattaaattaaacattaaatcagaactgttta

accttttggcttctctgggctacactggcttggcctaggtgtccacggta

catcacatgtattaaccacatgaagacatgtacatgtttatttaaaagga

taaatcaaatagcatg-tgacttatatatcaataaagatgtcaat-----

TGGTAATTT---TCT-AT-GTTCTGTTTTCCAGCTCGTCATATAGAATTT

AGGAAACACATTAAGGACATTAAGGTTCTAGAGAAGAAACGAGCCATGTT

TGAATGTGAAGTTT

>mouse

CACGCACTCGCTGAACATCAGGAATGTTCACTTTGGAGATGCTGCTGATT

ATACTTTTGTGGCTGGAAAAGCCACATCGACGGCCACCCTTTATGTGGAA

GGTAT-GATTTCCTGGATGT---ATGCACAGGGGGAA----GAAAGTCAA

A-----TATACCACG--------TATTAATCT---tttaatcaatgaatg

gataaa---------taaagtgtggtgtgtccctccaagttgggtggtaa

ctggaaataaaaaggagt----tactagcacatgctatgatatgggtagc

tgctgaaaatattatgagaaatgaaagaagccagccacagagcaa----c

aaacactgtaggagtccatttatatgaaatggccctgatacacaagtcta

ttgagacgtaga----gcattagcttcagtcta---gcaagaggtaatag

gaaat--gttctcgcatatggtggctaagagctatctgagcttttctgag

aaaaatgaaatggttttaa----aattatggtgatgcttatatgataagc

----------------------------------------atgatatcac

catggaag---------ccattaagttaaacaccaaatcagga-tgtcta

accttttggtttc-------------------------------------

--------------------------------------------------

-----tata--gaatg-tgactgatatatcaataatgatgtcaaa-----

TGCTAATTT---TTT----ATGTTGTTTTCCAGCTCGTCATATAGAATTT

AGGAAACACATTAAGGATATTAAGGTTCTGGAGAAGAAACGAGCCATGTT

TGAATGTGAAGTTT

>rhesusmonkey

CACACACTCCCTGAACATCAGGAATGTTCACTTTGGGGATGCTGCTGACT

ACACCTTTGTGGCTGGAAAAGCTACATCAACAGCCACACTTTATGTGGAA

GGTAT-GGTTTCATAAATATTAGATCCATAGAAAA-------AATGTCAC

ACATGCTTTGTAAA---------ATTTAGTGT---atcaattaatgaatg

gataaa---------taaaatgtgggatagccatacaatgggaaagcaat

cagcaacaaaaaggaagcac--tacgatttactacaaaatggatgaacat

taaaaactttatgctaagtgaaagaagccagagaccatacaaagatcaca

tattgcatgattccatttatatgaaatgtccaagatacacaaatctatag

agatgtagattggtggttgcctaggtctac-----aagggaga-caatag

aaaa---gtccttg-gggcaatggctaaggggtacataatttcttcttgg

ggaa-tgagaatgttctaa----aattatggtgatgtctgcacaatgatg

------------------------------------------aata----

catggaaa--------ctcattgaattg----------------------

--------------------------------------------------

-----------------------------------tgtactttaaatggg

tggattgtatagtatg-tgaattatatctcaataaagctgttaaaaa---

TGTTAATTT----TCTAC-ATGCTATTTTCCAGCTCGTCATATAGAATTT

AGGAAACACATTAAGGACATTAAGGTTCTGGAGAAGAAGCGAGCCATGTT

TGAATGTGAAGTTT

>human

CACACACTCACTGAACATCAGGAATGTTCACTTTGGGGATGCTGCTGACT

ACACCTTTGTGGCTGGAAAAGCAACATCAACAGCCACACTTTATGTGGAA

GGTAT-GGTTTCATACATATTAGATCCATAGAAAA-------AATGTCAT

ACATGCTTTATAAA---------ATTTAGTGT---atcaattaatgaatg

gataaa---------taaaatgtgggatagccatacga-tgggatatcat

tcagcaataaaaaggaag----cactacgatatgctacaacatggatgaa

cctttaaaactttatgctaagtgaaagaagccagagaccatacaaagacc

acatattgtatgattccatttatatgaaatgtccaagatacacaaatcta

tagagatgtaga-ttggtggttgcttaggtctac--aggggagacaatag

aaaa---gtccttg-gggtgatggctaagggttacatgatttcttcttgg

ggaa-tgagaatgttctaa----aattatggtgatggctacacaatgatg

------------------------------------------aata----

catggaaat--------tcattgaattg----------------------

--------------------------------------------------

-----------------------------------tatactttaaattgg

tggattgtacagtatg-tgaattatatctcaataaagctgttaaaaa---

TGTTAATTT---TTCTAC-ATGCTATTTTCCAGCTCGTCATATAGAATTT

AGGAAACACATTAAGGACATTAAGGTACTGGAGAAGAAGCGAGCCATGTT

TGAATGTGAAGTTT

>chimpanzee

CACACACTCACTGAACATCAGGAATGTTCACTTTGGGGATGCTGCTGACT

ACACCTTTGTGGCTGGAAAAGCAACATCAACAGCCACACTTTATGTGGAA

GGTAT-GGTTTCATACATATTAGATCCATAGAAAA-------AATGTCAT

ACATGCTTTATAAA---------ATTTAGTGT---atcaattaatgaatg

gataaa---------taaaatgtgggatagccatacaa-tgggatatcat

tcagcaataaaaaggaag----cactacgatatgctacaacatggatgaa

cctttaaaactttatgctaagtgaaagaagccagagaccatacaaagacc

acatattgtatgattccatttatatgaaatgtccaagatacacaaatcta

tagagatgtaga-ttggtggttgcttaggtctac--aggggagacaatag

aaaa---gtccttg-gggtgatggctaagggttacatgatttcttcttgg

ggaa-tgagaatgttctaa----aattatggtgatggctacacaatgatg

------------------------------------------aata----

catggaaat--------tcattgaattg----------------------

--------------------------------------------------

-----------------------------------tatactttaaattgg

tggattgtatagtatg-tgaattatatctcaataaagctgttaaaaa---

TGTTAATTT---TTCTAC-ATGCTATTTTCCAGCTCGTCATATAGAATTT

AGGAAACACATTAAGGACATTAAGGTACTGGAGAAGAAGCGAGCCATGTT

TGAATGTGAAGTTT

>treeshrew

CACACACTCACTCAACATCAGAAATGTTCACTTTGGGGATGCTGCTGACT

ACACCTTTGTAGCTGGAAAAGCTACTTCAACAGCCACCCTCTATGTGGAA

GGTAT-GATTTCATAAAGGTCATATGCATAGAAAAA------AATGTCAG

GTATACTCTATAAA---------ATGCCAGTAC--atcgtctgacaaatg

gataaa---------caaaatgtgggatattcacacag-tgggatattat

ttatcaataaaaaggaag----tactgatatagttaacatcaagggtgaa

ctctgagaactttatgctggaagaaagaagccaggcacaaagacacatca

tatgcttc---------atttataggga-tgtcagaacagacaatttgtg

gggacatatc---ttggtggttgtctaagtct----gggggaagatcatg

ggaagactctagggtctgatggctgagagtgcatagtgtcttttt--ggg

gaaagttaggatgttttaaattttattgtggtgatggttgcac-------

-------------------------------------------aac----

tctggatataccgaaagtcattgaattgtgt-------------------

--------------------------------------------------

--------------------------------------catttaaatgag

tgaattgtataatc---tgaattatatatcaattaagccattaaaa----

TGTAAATTT---TTTCAC-ATGCTGTTTTTCAGCTCGCCATATAGAATTT

AGGAAACACATTAAAGACATAAAAGTTCTTGAGAAGAAGCGAGCCATGTT

TGAATGTGAAGTGT

>tenrec

CACACACTCCCTGAACATCCGGAATGTGCATTTCGGTGATGCTGCTGACT

ACACGTTTGTGGCTGGGAAAGCCACATCGACAGCCACCCTCCACGTGGAA

GGTACGGGGTCCCACTT-GTCATAGCCATAA-------------AGCCAG

ACACACTAATTACAATGTC----AGCCAACCC---gtccaccgaatggag

gatggaggagaggtaaaacagggtgagacacccattcaatgggata---t

ttcacagc---aaggacac--gaaatgaaggatggatacaggtgaccatt

gggtgaata-ttatgttgaa--taaagaagccaattaagaaagac--cac

atagcggt-----ctctttatatgaaatgtctggaatagaccaaagacta

gtgcttgcgaggaccc------------------agggtaggaacgtgag

-------gctaggctgtgatggggagaagggctgtgagagctccttgggg

ggccgtgagaatgttctaaaattgattgtgctgatggttttttaaccctg

------------------------------------------agta----

catggaaag--------ccatcga-ttg----------------------

--------------------------------------------------

------------------------------------tatatttaaatggc

tgcattgtgtggaatc-agactcctacctcaatgaaattgtgaaaa----

TATGAACTC---CGTTTGTGTTCTGCTTTTCAGCTCGCCATATAGAATTT

AGGAAACACATCAAGGACATTAAGGTTCTGGAGAAGAAGCGAGCGATGTT

TGAATGTGAAGTTT

>armadillo

CACACATTCACTGAACATCAGGTAATGTCATTTTGGGGATGCTGCTGACT

ATAC-CTTGTGGCTGGAAAAGCTACATCGACAGCCAC-TGTTATGTGGAA

GGTAT-GGTTTCATA--CGTCAAATCCATAAAAT---------AAGCCCT

AAACACTGTTAACA---------TGTTAATCT---gtcacaactaatgaa

gacaaa---------taaaatgttgtatacctgtacaaacagaatattat

ttgccaataa-aaagaaat--gaaaaatgca-tgccacaacatggatgaa

ccttgaaaacattttgctaagtgaaataaaccagtcacaaaggac-----

---------------attcatatgaagtgtccaagacaggcaaatccata

aagac--agaaagtagattagaccttgcctagagc--tggggagagatgg

gggctggaactgggaagacgatggctaagggatatggagttactttgtgg

ggtcatgagaatggtcttaacttgattgtggtgctggttgcacaattttc

------------------------------------------gcta----

ttctgaaag--------ccactgaactg----------------------

--------------------------------------------------

-----------------------------------tgtgctttaagcgga

tgaattgtatggtgccctgaattgtctctcagtaaagctgttaaaa----

TGTTAATCA---CATGTACATGCTGTTTTCCAGCTCGTCATATAGAATTT

AGGAAGCACATTAAAGACATTAAGGTTCTTGAGAAGAAACGAGCCATGTT

TGAATGTGAAGTTT

>opossum

CACACACTCCCTGAACATAAGGAATGTCCATTTCGGGGATGCGGCAGAGT

ACACTTTTGTTGCTGGAAAAGCCACATCAACAGCTACCCTTTATGTGGAA

GGTAA-TCTTTCACAGTTGGGATATTTGAAAACATCCTCATCTCTTTCAA

AGGTACTATGAAAAA-----------------------------------

--------------------------------------------------

--------------------------------------------------

--------------------------------------------------

--------------------------------------------------

--------------------------------------------------

--------------------------------------------------

--------------------------------------------------

--------------------------------------------------

--------------------------------------------------

--------------------------------------------------

--------------------------------------------------

--------------------------------------------------

TGCTAATCA---GATTTTTGTGGTGTTTTCTAGCTCGTCACATTGAATTT

CGGAAACATATTAAGGATATTAAGGTTCTTGAGAAGAAAAGAGCGATGTT

TGAATGTGAAATTT

Marker 2a:

>L1MB5

--------------------------------------------------

------------------------------------------------tt

tggg-gtgatgaaa--atgttctggaactagatagaggtggt-ggttgca

caacattgtgaatgtactaaatgccactgaattgtwcactt-------ta

aaatggttaatttt-----------atgttatgtgaatttcacctcaatt

----twaaaaa---------------------------------------

--------------------------------------------------

--------------------------------------------------

--------------------------------------------------

--------------------------------------------------

--------------------------------------

>dog

AGTAGCATTATTTTGTGGTGCTGCAGCATTCCCCTCTAAATACATCTGCA

AAACCAAACAAAAA-GGGAATG---TAAAAAA-----CTAGG-GA---tt

tgcaggtgattaaaatatgttttagaactacaaagaagtgatgattg-ca

caacattgtgaatgtagtacatgccaccaaatcacatgctt-------ta

aaatgat-------------------taattttatagtatcacctcaatt

t---TAAT--AAAAGGAAA-------ACCAAGGAAATAATCTAC--TTAC

ATACAT-TCTG---------------------------------------

---------------------------------------GAGTAACCTTG

CCTGAGGGCAAAGAAATGGACTCTGTCTCTGTTTTCTCACGTTGCC----

------------------TTTCATCTAAA---GTCGTATTTGGCACTTC-

----AGGAAGAGGAGCATTTGTCCC-----CATGGTGA

>mole

AGTAGCATTATTTTGTGGTGCTGCAGCATTACCTTCTAAATACATCTAC-

--------CAAAAAAAAAAAAG---TAAAAAAAAAAaCTGAAGAA---ct

tgagggtgatgaaaa--tgttttagaattacatacaggtgattatag-ta

caatgttgtgaatatagtaaatgccactgaattgtgtt----------ta

aaatgat----------------taattttatgttatttgaattccatct

c---TGTTTAAAAAATAGAAAAA---ACTAAAGAAACAACCTA---TTGT

ATATAC-TCTG---------------------------------------

---------------------------------------GAGTGACCTTG

--TC-CAAGAAGTGTATGCAATTTATTTTTTTTTTCACA--TTCCC----

------------------TTCCATGTAAATTTATGGTATATAGCATTTC-

----TGAAAGAAGGGCAATTGTCCC-----CATGGTTA

>mouse

AGTGGCGTTATTTTGTGGTGCAGCAGCATTCCCTTCAAAATACATCTAC-

-----AGATGAAAACGAGGACG---TAAATAG-----CAAAATCG---ct

taagagcgaggcaa--atgttttaaaactaagtagcactaatgattgtta

ctgtattgggagtataatgactaccactgaattttccattt-------ta

aaggag----------------tacatgttattttagttttacttcaag-

----TA---AGAAAGCAAA-------AGGAACTGTCACCTACACACACAC

ACACACACATG----------------AGAG-GAGAAAGAGAGAGAGAGA

GAGAGAGAGAGAGAGAGAGAGAGAGAGAGAGAGAGAAGAGAGAGA--TTG

CCCCAGAACAAAGAAATGGATCCTATTTGTGTTTTCTCCTGTTCTC----

--------------------GCATCTAAA-TTACAGTATGTGACATTTC-

----TGGAAGCAGGGTATGCATTCC-----CATGGTAA

>rat

AGTGGCATTATTTTGTGGTGCAGCAGCGTTCCCCTCTAAATACATCTAC-

-----AGATGAAAAAGAGGATG---TAAATAG-----CAAAATCG---ct

taagagtgaggc-a--atgttttaaaactaagtggccctaatgatcgtta

ccgtattgggaatataataacta-cactgaattttccattt-------ta

aagtag----------------cacacgttattttagttttacttcaag-

----TA--AGAAAGCAGA--------AGAAACT--CACCTACACACACAC

ACACAC-ACACACACACACACACACACAGAGAGAGAGAGAGAGAGAGAGA

GAGAGA---------------------------------GAGAGAGAGAG

CCTCAGAACAAAGAAATGAACCCTATTTGTGTTTTCTCCTATTCCT----

-------------------CGAATCTAAA-TTACAGTATGTGACAGTTC-

----TGGAAGCAGGGTACACATTCC-----CATGGTAA

>rhesusmonkey

AGTAGCATTATTTTGCGGTGCGGCAGCATTCCCCTCTAAATACATCTAGG

AAGAAAAATGAAAAGAG-GATA---GAAAAAAA----CAAAATAA---tt

tgagggtaatacaa--atattttaaaatgaaatagaggtaag-agtggta

caacacattgaaagtagtaaatgccactgaattgtatactt-------ta

gagtggtcaattttgtgttatttaca---tatttacattttacctcaatt

----taaaaCAACAAGAAAA------AGTAGGAG-TACCATTTAT----T

ACAGATTTCTG---------------------------------------

---------------------------------------GAGTGACCCTG

CCTAAGGGCAAATAAATGGACCCTGTTTGTGTTTTCTCTCTGTCTTTTGT

TTT-----------------GCATCTACATATACTGTGTGTTGCACTTA-

-----GGAAGCAAGACATTTGTCCC-----TATGGTTA

>human

AGTAGCGTTATTTTGCGGCACGGCAGCATTCCCCTCTAAATACATCTAGG

AAGAAAAATGAAAA-GAGGATA---GAAAAAA-----CAAAATAA---tt

tgagagtaatgcaa--atattttaaaatgaaatagaggtaac-agtggta

caacatattgaatgtagtaaatgccactgtaaatgccactgaattgtata

aagtggtcaattttgtgttatttaca---tatttacattttacctcaatt

----TAA--AACAAGAAAA-------AGTAAGAG-TACCATTTAT----T

ACAGACTTCTG---------------------------------------

---------------------------------------GAGTGACCCTG

CCTGAGGGCAAAGAAATGGACCCTGTTTGTGTTTTCTCTCTCTCTCTCTC

TTTTTTTTTTTTTTTTTTTTGCATCTACATTTACTGTATGTTGCACTTA-

-----GGAAGCAGGACATTCGTCCC-----TATGGTTA

>chimpanzee

AGTAGTGTTATTTTGCGGCACGGCAGCATTCCCCTCTAAATACATCTAGG

AAGAAAAATGAAAA-GAGGATA---GAAAAAA-----CAAAATAA---tt

tgagagtaatgcaa--atattttaaaatgaaatagaggtaac-agtggta

caacatattgaatgtagtaaatgccactgtaaatgccactgaattgtata

gagtggtcaattttgtgttatttaca---tatttacattttacctcaatt

----TAAAACAACAAGAAAA------AGTAAGAG-TACCATTTAT----T

ACAGACTTCTG---------------------------------------

---------------------------------------GAGTGACCCTG

CCTGAGGGCAAAGAAATGGACCCTGTTTGTGTTTTCTCTCTCTCTCTCTC

----TCTTTTTTTTTTTTTTGCATCTACATTTACTGTATGTTGCACTTA-

-----GGAAGCAGGACATTCGTCCC-----TATGGTTA

>elephant

AGTAGCATTATTTTGGGGTGCTGCAGCATTCCCCTCTAAATACATCT---

----GGGGAAAAAAAAAGAGTG---TAAAAAA-----CTAAGGAA---tt

tgaaggtgatgaaa--aaaatttgaaag----------------------

-----------------tagatgccactgaattgtgcactt-------ta

aaatgattaatgttatgtt----acgtg-------aatttcacc------

--------------------------------------------------

--------------------------------------------------

---------------------------------------GTG--ACCTTG

TACAAGGGCAAAGAGGTGGACTATACTTGTGTTTTCTCATATGGTTCC--

------------------TTACATCTAAGTTTATTGTATGTAGCACTTC-

----TGGAAGTAGGTCACGTGTCCT-----GGTGGTA-

>tenrec

TGTGGCATTATTTTGGGGTGCTGCAGCATTCCCCTCTAAATACATCTAC-

--AGGGGCAAAAAAAGAGAGTT---TAAAAAA-----TGAAGGTA---tt

tgaggttgataaaa---aattttgaaacgagatattat------------

---------------------------tgaattcaacattt-------ta

aaataatta-ttttatg------acatg-----caaattttacctcaaa-

----TA---AGAAACCTAA-------AACATTT--CAAAAACAAA--CAA

AATAAACCCCCAAACAACTCCACTCAA-----------------------

---------------------------------------GGTGGTCATCA

TATAATTATAAAGTGGTGAACCATATATATGTTTTCTCAGATATTTTTT-

--------------------GCATCTAAATTTATTGAATAGATTATTTT-

----TTCAAGCAGGGAACTTGTCCC-----TGTGGTTA

>sloth

AGTAGCGTTATTTTGTGGTGCTGCAGCATTCCCTTCTAAATACATCTGGG

GGTGGAAATTAAAGA-AGGGTG----------------------------

--------------------------------------------------

--------------------------------------------------

--------------------------------------------------

----TAAAAAA----CTGA-------GGAAAC---TACTACAT-------

---ACATTCTG---------------------------------------

---------------------------------------GAGTGA-TTTG

CCTGAGGGCAGAGAGATGGACCACGTTTGTGTTTTCTCATGCTTCCT---

-------------------TGTATCTAAATTTACTGTGTGTAGCACTTGT

CCCTTGGAAGCAGGGCACTTGTCGC-----CATGGTTA

>armadillo

AGTAGCGTTATTTTGTGGTACTGCAGCATTCCCTTCTAAATACATCT---

--GGGGGGAAAAAT---TAG-A----------------------------

--------------------------------------------------

--------------------------------------------------

--------------------------------------------------

----TATATAAAAACCTAA-------GGAAAC---TACTATTT-------

---GCAATCTG---------------------------------------

---------------------------------------GAGCGAC-TTT

CCTGAGGGCAGAGGGATGGACCATATTCGTGTTTTCTCAAGTTTCCCT--

-------------------TTTATCTAAATTTACTGTATGTAGCACTTGT

CCCTTGGAAGCAGGACACTTGTCCTGTCACCATGGTTA

Marker 2b:

>L1MB5

--------------------------------------------------

--------------------------------------------------

--------------------------------------------------

--------------------------------------------------

--------------------------------------------------

--------------------------------------------------

--------------------------------------------------

--------------------------------------------------

--------------------------------------------------

--------------------------------------------------

--------------------------------------------------

--------------------------------------------------

--------------------------------------------------

--------------------------------------------------

--------------------------------------------------

--------------------------------------------------

--------------------------------------------------

--------------------------------------------------

-------------------------aatggggagtgactgcttaatgggt

acggggtttccttttggggtgatg---aaaatgttctggaactagata-g

aggtggtg----gttg-cacaacattgtgaatgtactaaatgccactgaa

-ttgtscactttaaaatggttaattttatgttat-gtgaatttcacctc-

-aatttsaaaaa--------------------------------------

--------------------------------------------------

--------------------------------------------------

------

>cow

-GATTACCTGCTCATCCAG-CCCTGGTGTAATTAAAAATGGCCAGCTTGG

GGCTCTGGTTTCCCCAGCCCTTGCAAAGGAAACTGGGTTTTATCTT-ATG

AAGCTTCAGGTTGGATCCAGGGGTTGACTTGGAGTCATAGGTGATCAGAG

GGACATCAGGATATTATCAAG-CACAGGAAATAAACTCCCAAGAAGAACA

AAATGATAGCAAGAGAAAATAAATCCTGAgggaaggatgagggaagggat

gatcaaggagtttgggatcacaggtacatgctgctatatttaaagtagat

gaccaacgaagtccgactgtatagcacagggaagtctgctcagtgttaag

tgcagcctggatgggaaggagtttagaagagaaaggatacgtgtacatgc

atggctgagtcactatgctgtgcccctgaaactatcacaacattgttaat

tgactatTGTTTCtgtggcttagttgctaagttgtgtccaactctttgcc

accccatggactgtagccgtccaggtttttctgtccatgggatttcccag

gcaagaatactggagtgggttgctgtttctttttccaggggatcttcccg

acttagggattgaacctgcatctcctgcttggcaggaggattctatacTC

CAATATAAAATaagtgaagttgctcagtttcgtccgactctttgtgagcc

catggactgtagcccactaggctcctccatcctttggattctcaaggcaa

gaatactggagtgggttgccgtttccttctccaggggatcttcccgaccc

agggatcaaaccccggtctcccgcattgcaggcagatgctttaacctctg

agccaccagggaagccCAA--TATAAAATA--AGAAGTAA-TAAAAAGAA

----AAAATATACCCTTAA------aagagggggctgtgtaatgggtaca

-gg--ctttt-------------------cccattttag-----------

-----------aactt-gctaagtgtgtgaacacactaaatgccactgaa

-gtgttcactttataatggttacttttatgttac-ctgacttttcacctc

---gtggaaaaaag----------------------------AAAGAGTG

AATCCCAA---TGAGCCCAGAACGTTTACATTTGAGAAGCTCCA------

--ATGTCCAACTCAGGCTTTGGCTCAGAGAAAATGCTTAATAAATGTTTA

TTGAAA

>dog

TGATTACCTGCTCATCCAG-CTCTGGCGTCATTAGACATGGCAGGCTCAG

GGCTCTGGTCTCCCCAGCGCTGGCAAAGGAAAGTGGATCCCAACTTGGAG

AAGCTTGAGGTCGGATCCTGGGTTTGGCTTGGCATCACCGGTGATCGGAG

GGCCATCGGGTCATTATCAAA-CCCAGGAAATCAGC--------------

--------------------------------------------------

--------------------------------------------------

--------------------------------------------------

--------------------------------------------------

--------------------------------------------------

--------------------------------------------------

--------------------------------------------------

--------------------------------------------------

--------------------------------------------------

--------------------------------------------------

--------------------------------------------------

--------------------------------------------------

--------------------------------------------------

----------------TCTG-AAGAAGGAC---GAAATGATAACAA-GAG

----AAAACATACCCTAAA------a--ggaggctga-tg-ttagtggct

acagcctttt------------------aatagtttcgg-ac-gtggtag

gggtgtc----ggtt--cacaatattctgaatgcactaagtgccactcaa

-ttgttctctttcaaatggttaattctgtgttat-gtggatatcacctca

gttggaaaaaaaaaaaaa------------------------AAAAAAAG

AATCCCAG---TGAGCCCAGGACATTTACATTTGAGAGACTCTA------

--ATGCCTAACTCAGGCTTTGGCTCAGAGTAAATGCTTAATAAATGTTTA

TTGAAA

>pangolin

CGGTTACCTGCGCACCCAC-CCCTGGGGTGATTAAAAATGGCAGGCCCAG

GGTTCTGGTTTCTCCTGTGCTGGCAAAGGAGACTGGAGTTCCACTT-ATG

AAGCTTGAGGTTGAAGCCAAGGGTTGGTTTGGAGTCACAGGTGATCCAAG

TGACATTAGGATAACAGCAGG-CCCAAGAAGTCAAC--------------

--------------------------------------------------

--------------------------------------------------

--------------------------------------------------

--------------------------------------------------

--------------------------------------------------

--------------------------------------------------

--------------------------------------------------

--------------------------------------------------

--------------------------------------------------

--------------------------------------------------

--------------------------------------------------

--------------------------------------------------

--------------------------------------------------

----------------TCCT-GACGAGGAC---AAAATGATAGCAA-GAG

----AAAATATGTCCTAAA------aa-gcaag-taa-tgtttaatgggt

acaagtttttc-------------------aagtttt-gaaatttggtag

aggtatt----ggtt--catgatattgtgaatgcactaaatgccacggag

-ttgttccctttaaaatggttaa-----ttttatgttgaatttcacttca

gttgggaaaa--------------------------------AAAAAGTG

AATCCCAG---TGAGCCCAGGACGTTTACATTTGAGAGGCTCTA------

--ATGCCTATCTCGGGCTTTGGTTCAGAGTAAATGCTTAATAAATGTTTA

TTGAAA

>bat1

CGATTACCTGCTCACCCAG-CCCTGGTGTAATTAAAAACGGCAGGCTCAG

GGCTCCGGTTTCCCCAGTTCTGGCAAAGGAGCCTGGGTTTCAGCTT-ATG

AAGCTGGAAGTTGGATGCAGGGGTTGGCTGGGAGTCACAGGACGTCGCAG

GCACATTAGGATCTTACCAAA-CCCAGGAAAGCTAC--------------

--------------------------------------------------

--------------------------------------------------

--------------------------------------------------

--------------------------------------------------

--------------------------------------------------

--------------------------------------------------

--------------------------------------------------

--------------------------------------------------

--------------------------------------------------

--------------------------------------------------

--------------------------------------------------

--------------------------------------------------

--------------------------------------------------

----------------TCCG-GAGAAGGACAAAACAATGACAGCAA-GAG

---AAAAATAGTCCCTAAA------aagaggg--tga-tgtataaagggt

acaggcttttc-------------------atgttctgga-agccgacag

agctgttg----gttcgcaacatcgtgtgcatgcactaaatgccactgaa

tttgtttgctttaaaatggttaattttatgttag-gtgaaattcaccgca

gttgtaaaa---------------------------------AAAGGGTG

AATCACAA---TGAGCCCAGGACGTTTACATTTGAGAGGCTCTA------

--ATGCCTAACTCAGGCTTTTGCTCAGAGTAAATGCTTAATAAATGTTTA

TTGAAA

>bat6

CGATTACCTGCTCATGCAG-CTCTGGTGTAA-TAAAAATGGCAGGCTCAG

GTTCCTGGTTTCCCCAGCACTGGCTAAGGAAACTGGGTTTCATCTT-GTG

AAGCTGGAGCTTGGG-CCAGGAGTTGGCTTGGAGTCCTAGGAGATCAAAG

GGACACTAGGATATTATCAAG-CTCAGGATATCAAT--------------

--------------------------------------------------

--------------------------------------------------

--------------------------------------------------

--------------------------------------------------

--------------------------------------------------

--------------------------------------------------

--------------------------------------------------

--------------------------------------------------

--------------------------------------------------

--------------------------------------------------

--------------------------------------------------

--------------------------------------------------

--------------------------------------------------

----------------TCCC-GACAAGGAC---AAAATGATAGCAAGAGA

-----AAATCTACCCTTAGA--------ggagggtga-catttaatgggt

acaggctttt-------------------tgagttttggaacttgaca-g

aggtatt-----ggtt-cacaatgttgtgaatgcactaaacgccactgaa

-ttgtttgcttaaaaatggttaattttatgttag-gtgaatttcaactc-

-agttggaaaa-------------------------------AAAGGGTG

AATCTCAA---TGAGCCTAGGACATTTACATTTGAGAAACTCTA------

--ATGCCTAACTCAGGCTTTGGCTCAGAGTAAATGCTTAATAAATGTTTA

TTGAAA

>shrew

CGACTACCTGCTTCTCCAG-CCCTGTAGTGATTTCAAAGGGCAGGCTCGG

GGCGCCCGTATCCCCGCAGTGGGCA-------------------------

-------------------GGGCAAACAGGGGAGCCTGAGTCAGATCTGG

GGACTGACATAGGACTGAAAG-CACGTTAGGGTGCT--------------

--------------------------------------------------

--------------------------------------------------

--------------------------------------------------

--------------------------------------------------

--------------------------------------------------

--------------------------------------------------

--------------------------------------------------

--------------------------------------------------

--------------------------------------------------

--------------------------------------------------

--------------------------------------------------

--------------------------------------------------

--------------------------------------------------

----------------TAGG-AGTCT--GG--GAAACCAAGGACAAGGCT

-------ATTGTCCAAGAA----aacagagcc--tgc------aaagggt

gatg-cttccc---tggggacagagaggtatg----gggccagcagtccc

agaccct-----gccacggccac-tggccactgtatctgctgctct----

-ttgttctgctctaaagggttaatttcatcttag-gtgaatttcacctc-

-agtggggagaaaaggaggga---------------------GAAGCCTG

AGGAGGAG----GAG---GGGAAGTTTACATTTGAGAGGCTCTG------

--ATGCCTAACT-GGGCTTTGGCTCAGAGTAAATGCTTAATAAATGTTTA

TTGAAA

>squirrel

CGATTACCTGCTCATCCAGCCCCTGGTGTAATTAAAAACGGCAGGCTCGG

CGCTCTGGCTTCCCCGGCGCTGGCAAAGGAGTCTGGGTTTCAACTT-GTG

AAGCTCAAGGATGGAGCCAGGGATTGGCTTGGCGTCCCGGGTGACCCGGG

GGTCATCAGGATGTTATCAAG-CCCCTGGGTTCAAA--------------

--------------------------------------------------

--------------------------------------------------

--------------------------------------------------

--------------------------------------------------

--------------------------------------------------

--------------------------------------------------

--------------------------------------------------

--------------------------------------------------

--------------------------------------------------

--------------------------------------------------

--------------------------------------------------

--------------------------------------------------

--------------------------------------------------

----------------G-CCTGGGAAGGAC---CAAAGA-GAGCTTGGAA

-----AAAGTTTCCTAAAAA------gaaggtgatgg---ttaac-ag-t

gca-ggcttttcttcgggatga-----tgttggttgt-gaacacagtga-

cggccc----------------------------------tgtcacagag

-ttgttcaatttagaatggttaattttat------gtaactttcacctt-

-aaaacaataccaacaaaaacaacaacaaaaacccaa-----GAAGCGTG

ACTCCCAG---TGAACTCTGGGCATTTGCATCTGGGAGGCCCAA------

--AAGCCTAACACAGGGTT-------------------------------

------

>rhesusmonkey

--------------------------------------------------

--------------------------------------------------

--------------------------------------------------

--------------------------------------------------

--------------------------------------------------

--------------------------------------------------

--------------------------------------------------

--------------------------------------------------

--------------------------------------------------

--------------------------------------------------

--------------------------------------------------

--------------------------------------------------

--------------------------------------------------

--------------------------------------------------

--------------------------------------------------

--------------------------------------------------

--------------------------------------------------

-----------------------GAAGGAC---ACAGCA-GAGCGA-GAG

----AAAAGGTACCTCAAA-------agaggc--tgg-tgttcagtgggt

acgggcttttcttttggggtgaggaaaaaaatgttttagaac-ttgtgag

aggtgttg----gttg-tgcaacatcgtgaatggattaaatgccaccgaa

-tcgttcactttaaaatggttaattttatgttgt-gtgaatttca--tc-

------gaaaaaaaataa------------------------AAAGGGTG

AATCCCAA---TGAACCCAGGTCGTTTACATTTCAGAAGCTCTA------

--ATGCCTAACTCAGGGTTTGGCTCAGAGTAAATGCTTAATAAATGTTTA

TTGAAA

>human

CGATTACCTGCTCACCCAG-CCCTGATGTAATTAAAAATGGCAGGCTAGG

GGCTCTGTTTCCCCCGGCTCTGGCAGAGAAACCTGGGTTTCGACTT-GTG

AAGCTTGAGGTTGGATGTGGGAATTGGCTTGGAGTCATAGGCGATGAGAG

GGACATTAGGATATTATGAAG-CCCGTGAACTCAAC--------------

--------------------------------------------------

--------------------------------------------------

--------------------------------------------------

--------------------------------------------------

--------------------------------------------------

--------------------------------------------------

--------------------------------------------------

--------------------------------------------------

--------------------------------------------------

--------------------------------------------------

--------------------------------------------------

--------------------------------------------------

--------------------------------------------------

----------------TCCT-GAGAAGGAC--GCAGCAGA--GCGA-GAG

----AAAAGGTATCTTCAA------aagaggc--tga-tgttcagtgggt

acgggcttttcttttgggatgaggaaaaaaatgttttagaa-cttgtgag

aggtgttg----gttgtgcagcatc-gtgaatggactaaatgccaaagaa

-tcgttcactttaaaatggttaattttatgttgt-gtgaatttca--tc-

---gaaaaaaaaaaaataa-----------------------AAAGGGTG

AATCCCAA---TGAACCCGGGTCGTTTACATTTCAGAAGCTCTA------

--ATGCCTAACTCAGGGTTTGGCTCAGAGTAAATGCTTAATAAATGTTTA

TTGAAA

>chimpanzee

CGATTACCTGCTCATCCAG-CCCTGATGTAATTAAAAATGGCAGGCTAGG

GGCTCTGTTTCCCCCGGCTCTGGCAGAGAAACCTGGGTTTCGACTT-GTG

AAGCTTGAGGTTGGATGTGGGAATTGGCTTGGAGTCGTAGGCGATGAGAG

GGACATTAGGA--TTATCAAG-CCCGTGAGCTCAAC--------------

--------------------------------------------------

--------------------------------------------------

--------------------------------------------------

--------------------------------------------------

--------------------------------------------------

--------------------------------------------------

--------------------------------------------------

--------------------------------------------------

--------------------------------------------------

--------------------------------------------------

--------------------------------------------------

--------------------------------------------------

--------------------------------------------------

----------------T-CCTGAGAAGGAC---ACAGCA-GAGCGA-GAG

----AAAAGGTACCTTCAAA------agaggc--tga-tgttcagtgggt

acgggcttttcttttgggatgaggaaaaaaatgttttagaac-ttgtgag

aggtgttg----gttg-tgcagcatcgtgaatggactaaatgccaaagaa

-tcgttcactttaaaatggttaattttatgttgc-gtgaatttca--tc-

------gaaaaaaaataa------------------------AAAGGGTG

AATCCCAA---TGAACCTGGGTCGTTTACATTTCAGAAGCTCTA------

--ATGCCTAACTCAGGGTTTGGCTCAGAGTAAATGCTTAATAAATGTTTA

TTGAAA

>elephant

CCATTACCTGCTCATCCAGCCCCTGATGTAATTAAAAATGGCAGGCTTGG

GGCTTAGGTTTCCCTAGGGCTGGCAGAGAAGTCTGGGTTTCAGCTT-GTG

AAGCTTGAGTTTGGATCCAGGGGTTGGCTTGGAGTCATAGGTGACCAGTG

GGACACTGGAATATTATCAAG-TCCAGGAAATCCCC--------------

--------------------------------------------------

--------------------------------------------------

--------------------------------------------------

--------------------------------------------------

--------------------------------------------------

--------------------------------------------------

--------------------------------------------------

--------------------------------------------------

--------------------------------------------------

--------------------------------------------------

--------------------------------------------------

--------------------------------------------------

--------------------------------------------------

----------------TAGA-TGG----AC---AGAACAAGAGCTA-AAG

----AGTAA-CACCTTAAA----aacacaaaagagggatgcttaatggct

agggggtttcctttccgggtgatggg---aatgtttg-gagc-tggatcg

cggtgaca----gttg-tacaacacggtgaatgtactaaatgtcactcaa

-ctgttcactttaaaatggttaattttacgttat-gtgaattttagaatt

ttaacttaattaaaaagagaaa--------------------AAGGGGCG

AATCCCAA---TGAATCCAGGACGTTTACATTTGAGAGGCTCTATTTTTT

TAATGCCTAACTCAGGGTTTGGCTCACAGTAAATGCTTAATAAATGTTTA

TTGAAA

>seacow

CGATTACCTGCTCATCCAG-CCCTGATGTAATTAAAAATGGCAGGCTTGG

GGCTTTGGTTTCCCTAGCGCTGGCAAAGAAGCCTGGGTTTCAGCCT-GTG

AAGCTTGAGGTTGGATCCAGGGGTTGGCTTGGAGTCATAGGTGACCAGAG

GGACATTGGAATATTATCAAG-TCCACGAAATCCCC--------------

--------------------------------------------------

--------------------------------------------------

--------------------------------------------------

--------------------------------------------------

--------------------------------------------------

--------------------------------------------------

--------------------------------------------------

--------------------------------------------------

--------------------------------------------------

--------------------------------------------------

--------------------------------------------------

--------------------------------------------------

--------------------------------------------------

----------------T-----AGATGGACAGAACAA-GA--GCTAAAGA

----GTAATA-TCGTAAAA------aataaaagggggatgcttaatgggt

gtggggtttccttttggggtgacggg---aatgtttg-gaa-ctggatag

aggcaatg----gttg--caa-------gacacta-tgaatgtcacttaa

-ctcttccctttagaatggttaattgtatgttat-gtgaattttacctca

a--tcaaaaaaaggaa--------------------------AAGGGGAG

AATCCAGG---TGAACCTAGGACGCTGACTTTTGAGAGGCTCTA------

--ACGCCTCACTCAGGGTTTGGTTCAGAGTGAATGCTTAATAAATGTCTA

TTGAAA

>sloth

CGATTACCTGCTCATCCAG-CCCTGATGTAATTAAAAATGGCAGGCTAGG

GGCTCTGGTTTTTACTGTGCTGGCAAAGGATGCTGGGTTTCAACTT-GTG

AAGCTTGAGGTTGGATCCAGAGGTTGGCTTGGACTCC-CCATCATCAGAG

GGATGTTAGAATATTATCAAG-CCCATGCAATCAAC--------------

--------------------------------------------------

--------------------------------------------------

--------------------------------------------------

--------------------------------------------------

--------------------------------------------------

--------------------------------------------------

--------------------------------------------------

--------------------------------------------------

--------------------------------------------------

--------------------------------------------------

--------------------------------------------------

--------------------------------------------------

--------------------------------------------------

----------------TCCA-GAATAAGA------GAAGA-TACCTCAAC

--------------------------------------------------

--------------------------------------------------

--------------------------------------------------

--------------------------------------------------

----------------------------------------AAAAAGGGTG

AATCCCAA---TGAACCTAGGACATTTACATTTGAGAGGCTGTA------

--ATGCCTCACTCAGGGTTTGGCTCAGAGTAAACGCTTAATAAATGTTTA

TTGAAA

>armadillo

TGATTACCTGCTTAGCCAGCCCCTGGTGTAATTAAAGATGGCCGGCTCGG

GGCTTTGGTTTCCAGGGTGCTGGCAGAGGAGCCTGCGTTTCGACTT-GTG

AAGCTAGAGGTTGGATCCAGGGATTGGCTAGGAGTCCTAGGTGATCAGAG

GGG-ATTGGAATACAATCAAG-CCCATGAAATCAAC--------------

--------------------------------------------------

--------------------------------------------------

--------------------------------------------------

--------------------------------------------------

--------------------------------------------------

--------------------------------------------------

--------------------------------------------------

--------------------------------------------------

--------------------------------------------------

--------------------------------------------------

--------------------------------------------------

--------------------------------------------------

--------------------------------------------------

----------------TCCA-GAGGGAGTGAGAAAAGATA---CCTTAAC

--------------------------------------------------

--------------------------------------------------

--------------------------------------------------

--------------------------------------------------

-----------------------------------------AAAAGGGTA

AATCCCA----TGAACCCTGGGCATTTACATTGGAGAGACTGTA------

--ATGCCTCACTCAGGCTTTGGCTCAGAGTAAATGCTTAATAAATGTTTA

TTGAAA

Marker 3a:

>L1MB5

--------------------------------------------------

--------------------------------------------------

--------------------------------------------------

--gtactaaatgccactgaattgtwcactttaaaatggttaattttatgt

tatgtgaatttcacctcaattt---waaaaa-------------------

--------------------------------------------------

--------------------------------------------------

--------------------------------------------------

--------------------------------------------------

--------------------------------------------------

--------------------------------------------------

--------------------------------------------------

--------------------------------------------------

--------------------------------------------------

--------------------------------------------------

--------------------------------------------------

--------------------------------------------------

--------------------------------------------------

--------------------------------------------------

--------------------------------------------------

--------------------------------------------------

--------------------------------------------------

-------------------------------

>dog

GTGGCAGCACC----------AGGCCTGGCAGGGGGAAGGGGACTTCATT

-CGTCATTATCTACTGTTGCTGT-------CATTACTGACTCTACCCTTA

GCTCCCCTCTGAGTGCAAACAAGTGGGGATTGGGGGTCA-----------

--gtactaaatgccactgaattgtttacattaagatagttaattttatgc

tatatgaatttcacctcaattt---TTAAAAAAAGGAGGAG---GAGGGC

TAGGGACCATCATTATTT--------------------------------

--------------------------------------------------

--------------------------------------------------

--------------------------------------------------

---TCTGCCTACAGGATCGTAAAGGCATCTCATTGAGAGTGCAGC-TAGT

TA-GGCCTGAATTGCTCCTAAAAGCT-ACCCCC----CATCAGCCTTC--

--------TGAACCTCTTCTAGGGGTCAGCTGTGT---------------

---TCTCTTTCCCAGAAGAGAGAG-----CCTGGCTCCCTTGTGCCCTGG

CTTTGTGGAGGGGAGGAGAGAGTTGGAAGAGA-GTAGGG---------CA

TTGGGCTCCTGGCT------GCCCCCATCAAAGGGACACAGGGACGTCTT

GGATA---TCTGTCCTTTCTCTGGGCTGCTGTGAGATCCCTGACAGCTGT

GCTGTGTGGTGGCTTTCCAGG--TCCTTTCTAGGTGTCCTCTGCCACCTT

TCTAGGT---GACACCTCTGCCAAGCAGCAGTCAGCCTGTCTGCTGGACT

GAGAGACACGCCCCCCTCACCCCCCCCACCCCCAGATGGGAAACTGATGT

TCAGCACAACCAGGGATGTGCCCAGGATGATGT---TAATGTCTTAAGGC

AGCCTCAGCCCTGCTGGCCCTGTGGGTGTCACTGGCTTTCTGGATGTGCC

AGAAGCATGGTGTGTATGGGGTGCTTCTGAAATCAGAGCTTCTTT-CACC

ACACCAGGGGAAGCTCCTATTTTAAGTTTCC

>pangolin

ATGGCAGCTCCTTGGAGCACCAGGCCTGGCAGGGGCAAAGGGACT-----

-CGTCATTTT---CTGCTGCTGTTGT----CATTAGTGACTCTACCCTCA

ACTCTACTGAGTATGAAAG--AGTGGGGAAA--GGGTCA-----------

--gtgctaaatgccaatgaattgcgcacattcaaatggttaattttttgt

tatgtgaatttcacttaaattt---TTTAAAAAAGGAAGGG---CAGGGC

TAGGGGCCACCACTACTT--------------------------------

--------------------------------------------------

--------------------------------------------------

--------------------------------------------------

---TCTGCCTAGAGGACGCTCATCTCATCTCACTGGGTGTGCACTTCAGC

C--------------------------ATCACCCC--CATCAGGCTTC--

-----------TGCATTTCTGGGG-TCAGCTGTTT---------------

---CCCCTTTCACAGAGGGCAGAG-----CCTGGCTTCCCTAGGCACTGG

CATCATGCAAGGGAGGAGAGAGTTTGGAGGAGAGTCAGGA--------CG

TTGGGCTCTGGCCC------------ATCGAG-GGACACAGGGAGGCCTT

GGACT---TCTGTCCTTTCTCCGGGTTGCTTGGAAT-CCCTGCCAGCTGT

GCTGAGTGGTGG-------------CTTTCCAGGT--CCTTTTT-AGTGT

CCCAGGAC--G-AGGAGCACAGCTGCCAA------CCTGTCTGCTGGACC

ATGGGATTACCCCCCTGTCACCCCCGCAAAGGGGACTCTGA---TGATAA

GCACAAGCAGAGAGG---TGCCCAGGCTGATGC---CAACATCTGA-GGC

AGCCC---------TGGCC-------TGACCGCACCCCCCCTGCACGGCA

GCCTCCTGGCTGTGCATGGGGAGCTCCTGAAAGCAGAGCTCCTT--CACC

ACACCAGAGGAAGCTCCTATTTTAAGTTTCC

>rat

GTGGCAGCTCCCCAAGCAAC-AGGCCTGGTAGGGGGAAGGGGACA-CATC

CCCTCAGTTATTA------CTA----------------------------

--------------CAGAGTACTGATGGAAACACCAGAAAAGGGGGGGG-

--gtcactaaataccgctgagttctctagtaaaaat---cagttttatgc

tatatgaatttcacctccattt---CTTTCAAGGGGGGAAG---GGAGGC

AAGGG-CCAACACTACTT--------------------------------

--------------------------------------------------

--------------------------------------------------

--------------------------------------------------

---CCTGTCT----------------------------CTTCAGCCCCAC

C---CAAGT-ACTCCTCCAGGTGACCTAT---------TCACTCCAGCAT

CTGCATGTTCTAGCTTTTCTGCTG-TCATCTTCTT---------------

---CCCCTTTCCCAAAGGACGGGCCTCA-TCTGG--------CTGGGTCC

CCTATGCCGGTGT-TACAGGAG-TAGATGTATGA---------------A

CCTGGCTTCTGACCAATAGGACTCCTGGGCTGACTCCATT-AGCGCTGCT

GGCAAGCCTGGGATGTGTGTCTCCAGGCCACTGTGAGAGCTGCCAGCTAT

AGAGAGGGGTGGCCT-CTGTGGAAGAGCAAGACTACTCAGCCTGGGGCCT

TCCAGAT---GTAGGCACTCCCTTGTGATGGGCGTGGTTGCTA-TGATCT

GTGCCA--------------GCCTG----ACACGTCTAAGATCTTGGGGT

ACTC--TACG-TCC-AGTGCCCTGTCAGC---------------------

----------------------------CTCTCTGCCAACGGCTGTGCTC

CAAGCTGCCTGAAGCCCGTGGTGGTTCGTAA-GGGGTGCTTCAGA-AACC

AGAGCTGCAGCAGCTCCTATTTTAAGTTTTC

>rhesusmonkey

ATGGCAGCTCCCCAGAGCACCAGGCCTGG--------------CT-GCTA

TTGTCATAACTGA------CTG----------------------------

-----------AACACAAAAATAGTGGTAGGGCGGGGAGGGAGGGAAGA-

--gtagtaaatgccactgaattgctcactgtaaaatggctaattttacgt

tatgtgaatttcacctggattt---TTTTTAAAAGGAGGGG---CAGGGC

TAGGGGCCACTACTACTT--------------------------------

--------------------------------------------------

--------------------------------------------------

--------------------------------------------------

---CCTGTCTAGAGAATCCTAAATGCATCTCACCAGGCCTGCAGCTCAGC

C---TGAGTTGTTCCTCCAAATAGCCCATCTGCCCATCACTGGCCTTCAT

CAGCCCTCTGGAACTGTTCTAGGAATCAGCCACTT---------------

---CCCCTTTCCCAGAGGACAGACCAGAGCCTG-CTCCCCTGTCGCCTCC

CCTGGGAGGAGAAGCATCAAGGGGAGTCAGGA----------------CA

TTGGGCTTCTGCCC---TTGGCTCCATTACAGAATC-ACTGGGAAGCCTT

AGACA-----TTCATCCTCTCTGGGCTGCTGTGAAATC--TGCCAGCTAT

GCCGAGTGGTGGCTT-CTTGG--TCCTTTCTA---GTCCTAGGACACCTC

TGCCAAG---CACAGCAGCTCAGCGTGT------------CCCCTGGACT

GTGAG---------------GTCTCTGCCCACAGACATGGACTTTGATGA

TCGGCACACTCTCCGAGGTGCCTAGGCTGATGTGGTTAA-----------

----------------------------CGCCCAGCTTTCTGGATGCGCC

GGAAGCATGGTGTGTGTGGACTGCGTCTGAAATCAGAGCTTCTTT-CATT

GCACCAGGGGAAGCTCCTATTTTAAGTTTCC

>human

ATGGCAGCTCCCCAGAGCACCAGGCCTGG--------------CT-GCTA

TTGTCATTACTGA------CTG----------------------------

-----------AACACAAAAATAGTGGTAGGGCGGGGAGGGAGGGAAGA-

--gtagtaaatgccactgaattgctcactgtaaaatggctaattttacgt

tacgtgaatttcacctggattt---TTTTTAAAAAGAGGGG---CAGGGC

TAGGGGCCACTACTGCTT--------------------------------

--------------------------------------------------

--------------------------------------------------

--------------------------------------------------

---CCTGTCTAGAGAATCCTAAATGCATCTCACTGGGCCTGCAGCTCAGC

C---TGAGTTGTTCCTCCAAGTAGCCCATCTGCCCATCATTGGCCTTCAT

CAGCCCTCCGTAACTGTTCTGGGGATCAGCCGCTT---------------

---CCCCTTTCCCAGAGGACAGACCACAGCCTG-CTCCCCTGTACCCTCC

CCTGGGAGGAGAAACATCAAGGGGAGTCAGGA----------------CA

TTGGGCTTCTGCCT---TTGGCTCCATTACAGAATC-ATTGGGAAGCCTT

GGACA-----TTCATCCTGTCTGGGCTGCTGTGAAATCTCTGCCAGCTAT

GCCAAGTGGTGGCTT-CTTGG--TCCTTTCTGGATGTCCTAGGACACCTC

TGCCAAG---CACAGCAGCTCAGCCTGT------------CCCCTGGACT

GTGAG---------------GTCCCTCCCCACAGACATGGACTCTGATGA

TCGGCACACTCTCCGAGGTGCCTAGGCTGATGTGGTTAA-----------

----------------------------CGCCCGGCTTTCTGGATGCACC

GGAAGCGTGGTGTGTATGGAGTGCGTCTGAAATCAGAGCTTCTTT-CATT

GCACCAGGGGAAGCTCCTATTTTAAGTTTCC

>chimpanzee

ATGGCAGCTCCCCAGAGCACCAGGCCTGG--------------CT-GCTA

TTGTCATTACTGA------CTG----------------------------

-----------AACACAAAAATAGTGGTAGGGCGGGGAGGGAGGGAAGA-

--gtagtaaatgccactgaattgctcactgtaaaatggctaattttacgt

tacgtgaatttcacctggattt---TTTTTAAAAAGAGGGG---CAGGGC

TAGGGGCCACTACTACTT--------------------------------

--------------------------------------------------

--------------------------------------------------

--------------------------------------------------

---CGTGTCTAGAGAATCCTAAATGCATCTCACTGGGCCTGCAGCTCAGC

C---TGAGTTGTTCCTCCAAGTAGCCCATCTGCCCATCATTGGCCTTCAT

CAGCCCTCCGTAACTGTTCTAGGGATCAGCCGCTT---------------

---CCCCTTTCCCAGAGGACAGACCACAGCCTG-CTCCCCTGTACCCTCC

CCTGGGAGGAGAAACATCAAGGGGAGTCAGGA----------------CA

TTGGGCTTCTGCCC---TTGGCTCCATTACAGAATC-ATTGGGAAGCCTT

GGACA-----TTCATCCTGTCTGGGCTGCTGTGAAATCTCTGCCAGCTAT

GCCAAGTGGTGGCTT-CTTGG--TCCTTTCTGGATGTCCTAGGACACCTC

TGCCAAG---CACAGCAGCTCAGCCTGT------------CCCCTGGACT

GTGAG---------------GTCCCTCCCCACAGACATGGACTCTGATGA

TCGGCACACTCTCCGAGGTGCTTAGGCTGATGTGGTTAA-----------

----------------------------CGCCCGGCTTTCTGGATGCACC

GGAAGCGTGGTGTGTATGGAGTGCGTCTGAAATCAGAGCTTCTTT-CATT

GCACCAGGGGAAGCTCCTATTTTAAGTTTCC

>seacow

GTGGCAGTTCCCTGGAGCATCAGGCCGGGCAGAGGGAAGGGGACTCAATT

TTGTCATTCTT------TGCTGTTTTTTGTCATTACTGACTCTACCCTTA

ACTTGA---TA---------------------------------------

--------------------------------------------------

-------------------------AAAATAAAAAAGAAGG---CAGGGC

TGGAGACCACTACTTCCTgcctgttgttgtgtgccattgagttgatttag

tgacccaatgtgacagagaagaactgccccataggg-tttcctaggtt--

----gtaattttta---tgggagcctctgggtcagtttaaactgctgact

tttcagttagcagctgagcactttaaccattGTGCCACCAGGGCTCCTTA

CTTCCTGTCCAGAGGACCCTAAACGCATCTTGCTGGACTGGCACCTCAGC

C---TGAGTTGCTCCTCAAAGCAGTCCACCTGCCCCACAGCCACTTTCAT

CAGCCTTCTGTACCTGTTCTGGGGGTCAGCCACTTCCTGGTGACAGGGTA

GGTCCCCTTTCCCAGAGGACAGACGAGAGCTTGGCTCCCATGTGGG-CCC

TGGCTGGCATCATGGCAGGGAGGGGAGAAGATGGAGGGGAGGCAGGATAC

TGGGCTCTTGCCCT-------GCCCCATCAAAGAGCCACTGGGAGGCCT-

GTATG---TCTGTCCTTCCTCTGGTC-ACTGTGAAGTCCCTGCTGGCTCT

GAGGAGTGGTGGCTTTTCCAGG-TCCTTTCTAGATGTCTTAGGATATGTC

TGCTAAGAGGCACAGCAGCTCAGTCTGC------------TCCCTGGACC

CTGAG---------------GCCCC------ACACAGGGGACTCTGAGGA

TCAGCACAACCTTGGAGGTGCCCAGGCTGGTGTAGTTAATGCCTCAAAGC

AGCCCAGCTCTGCCTGCCGCATGGATGTCACCAGCCTT-CTGGATGTGCC

GGAAGTATGGTGTGCATGGGGTGCTTCTGAAATCAGAGCTTCTTTTCATC

GCTCCCGGGGAAGCGCCTATTTTAAGTGTCC

>elephant

GTGGCAGCTCCCTGGAGCACCAGGCTGGGCAGGAGGAAGGGGACTCAATT

TCGTCATTCTC------TGCTGTTGT----CATTACTGACTCTACCCTTA

ACTTGA--------------------------------------------

--------------------------------------------------

-------------------------TTAAAAAAAAAAGAGG---CAGGGC

TGGAGACCACCACTACTTcacac-----gagtcgattcttgactcatagt

aa-cccaatgtgacagagtagaactgccacatagggtttttctaagatcc

ccaggtcttttttccccttggagcctcagggggaatttaaactgctgacc

tttcagttagcagccaagcactttaaccattGTGCCACCAGGGCTCCTTA

TTTCCTGTCCAGAGGACCCGAAATGCATCTTGGTGGCCTTGCAGCTCAGC

C---TGAGTTGCTCCTCAAAGCAGTCCACCTGCCCCACAGCCGCTTTCAT

CAGCCTTCCATACCTGTTCTGGGGGTCAGCCACATCCTGGTGACAGGGCA

GGTCCCCTTTCCCAGAGGACACAGCAGAGTTTGGCTCCC-----------

---CTGGCATCGTGAAAGGGAGGGGAGAGGATGGAGGGAAGACAGGATTC

TGGGCTCTTGCTCT-------GCCCCATCAAAGAGCCACTGGGAGGCCT-

GGATG---TGTGTCCTCTCCCTTGTC-GCTGTGAAGTCCCTGCTGGCTCT

GATGAGTG-TGGCTTTTCCAGG-TCCTTTCTAGATGTCCTAGGATATGTC

TGCCTTGAGGCACAGCAGC---------------------CCCCTGGACC

ATGAG---------------GCCCCC-----ACAGAGGGGACTCAGGATC

AGTACA--ACCTCGGAGGTGCCCAGGCTGGTGTGGTTAAAGCCTCAGAGC

AGCCCAGCCCTGCCGGCCCCATGGGTATCACCAGCCTT-CTGGATATGCT

GGAAGCGTGGTCTGCATGGGGCGCTTCTGAAATCAGAGCTTCTTTTCATC

ATACCCGGGGAAGCACCTATTTTAAGTGTCC

>sloth

GTGGCAGCTTCCAGGAGCACGGGGCCTGGCAGGGGGAAGGGGACTCAATT

CTGTCATTCTCGTTCTCTGCTTTTGTTTGTCATTACGGACTCTACCCTTA

ACTCTA---TGCTGAGCCC-------------------------------

--------------------------------------------------

-------------------------AAAAGGGGGGTGGGGG---CAGGGC

TGGAG-CCTCCCCTACCT--------------------------------

--------------------------------------------------

--------------------------------------------------

--------------------------------------------------

---CCTGACTAGAGGACTCTAAATACATCTCACAGGGCCTGCGGCTCAGC

CGCCAGAGTTGCTCCTGAAAGCAGCTCACCTGCTCCACAGTCGCCTTC--

--------TGCAACTGCTCTTGGGGCCAGCCACTTCCGGGTGTCAGGACA

GGCCCCCTTTCCCAATGGTCATGCCAAAACCTGGTTCCCCTGTGGGGCCC

TGGCGTCAATGGAAAGGGAGGAAGAAAAGGGAATGGAGG------CGAGT

CTGGACCTTGGGCTCCTGCC------------------------------

--------------------------------------------------

--------------------------------------------------

--------------------------------------------------

--------------------------------------------------

--------------------------------------------------

--------------------------------------------------

--------------------------------------------------

-------------------------------

Marker 3b:

>L1MB4

--------------------------------------------------

--------------------------------------------------

--------------------------------------------------

-------------------------------ttatt-cacaatagccaaa

aggtggaaacaacccaagtgtccata-------acggatgaatggataaa

caaaa-tgtggtatatacatacaatggaatattattcagccttaaaaagg

aaggaaattctgatacatgctacaacatggatgaaccttgaaaacattat

gctaagtgaaataagccagwcacaaaaggacaaatattgtatgattccac

ttatatgaggtacctagaatagkcaaattcatagagacag-aaagtagaa

ta---gwggttaccaggggctggggggaggggaaaatggggagttactgt

ttaatgggtacagagtttctgtttgggatgatga--aaaagttctggaaa

tggatagtggtgatggttgcacaacamtgtgaatgtacttaatgccactg

aattgtacac----------------------------------------

--------------------------------------------------

-------------------------------------ttaaaaatggtta

aaatggtaaattttatgttatgtatattttaccacaataaaaaa------

--------------------------------------------------

--------------------------------------------------

--------------------

>dog

GGCCTTATATCTCACCTGCGTGTGAAGCAGTAAGA---------------

-GTGAAATCAC---ATGAATGGGGACC--GTTAAG-----CAGTACTAAC

GGAAGCCAT---GGTAAGACCCCCCT-CCCGACATGCAG-GCTGGAGATG

G---GGAG----GAAGG--GCCACACGC---ctcgttcacaggagctcaa

aagcagaaacaactcgggtgtccacca------gcagatgaatcagcg--

---------ggcacacaccacg---gga-tgtggctgagtcataagagga

gatgtagttctgacaaatgcttccacaggggtgaaccctgaaaacattat

gctaagtcaagccagacag--acaaaaggacaagtgttgtatgattccac

tgatgtcagattctctagg--aggcgaactcac--------aaagtaggt

tatcgaggtta-caaaggctgtggggagagagaagtggagagct------

--------------aggg--cttaagaggcgcagaatttctgtttgg--g

gaga---tggaaataatggcagtagctggacagcactgtaaacgtgaccg

at-gccactcagcggtacgtctatttgtttatgttagcaacctccgcgtc

caacgtgtggctcaaagtcacgacccccgagatcaagagtcttacgctct

cctgactgagccagccaggggcccctcaactgcacatttagaaatagtta

aaatggcaaatttcacgtta--tatattttaccccaattagagaaaacaa

aca---GA-CCATACCTCAC---TTGCGAGATTGTTAGCATCTTTCATGA

CTTTGTAGAGCTGGCTGTCTTTTGGGTTGTATTTTGGTGTTTTGCCCTTC

TCTTTGTTGAAATTTTCTCG

>pangolin

AGCCTTGTACTTAACCTATTTGTGAGGGGGGAAAA---------------

--GAAAAACAC---ATAAACAGGAAAC--GGACG-----ACAGTACTAAG

GGAAACCCT---AGTAAGACACTCCA-TCCTGGAACAGACGCTGGAGATG

G---TGGT----GAAGA--GCCATACC----tcagcct-cactcgt----

-ggtagctgaaga-gtagttagg---aaggaagtcagtttgccacat---

----------gctacagca-----tgga-tgaacactgaaaatgtatctt

acgtgaaataagcca---ga--ccaaaaaaataggaca---catattatg

tgattccacttacagtaaatctctaataggcaagt-----------ccac

agaagc-----------------------------------aaagt----

tatggaggttaccaggggctggaggcgaggaggaa-gag--tctctgctt

catagtta-cggtttctc--tttggggtgacataaaa---gttttggttt

ggg----tgatggaaataatggtggtagttggacaacattgtgaaggtaa

ttaatgtctctca-------------------------------------

--------------------------------------------------

---------------------------------acacttaaagatggtta

aaatggcaaatttcatgttg--catgttttaccacaattaaaa-------

------AAAACATACCTCGC---TCGCAAGATTGTTAGCATCTTGCATGA

CTTTGTAGAGCtGGCTGTCTTTGGGATTGTATTTTGGTGTCTTGCCCTTC

TCTTTGTCGAAATTTTCACG

>mouse

AGCCTTATATTTGACCTGGTGGTAAAGAAGGAAAT---------------

-------TAAC---ATATGTAGGAAAT-GGGAA------ACAGCACTAAG

GAAGATGGC---------------CGTTCATGAAACGAGTTCTGGGGGTG

GC--TGA-----GAAGG---AAAACCAC---tttgtgcatagctgccaaa

aagtagaaatgaccggagtgtccatcaatagataaacgggtatcattaaa

aaaaagtgtggcatacacac----tggaattttactcagccataaaatgg

gatatagtgctaattaatgctgcacta-----------tgaa-atgctat

gctaaataaaatag----gacacaaagagacat--actgtaggattccat

gtat----------------------------------------------

----gaaggagacgtta-ccaggagcta-agggaagaaagggtt------

------gcttcatggatttgtttggggccatta-caaa--gctttggaaa

tag----tggtggtaatttaagagcaccatgaatgtgatcaatgctcctt

aaatg---------------------------------------------

--------------------------------------------------

--------------------------------------------ggatta

aaatggcgaacagtatgtta--tgtcctttaccacaatgaag--------

------GAA-CATACCTCGC---TTGCAAGGTTGTTGGCATCTTTCATAG

TTTTGTAGAGCTGGCTGTCTTTCGGGTTGTATTTGGGTGTTTTGCCCTTC

TCCTTGTCAAAATTTTCGCG

>rat

AGCCTTGTATTTGACCTAGTGGTAAAGAA---------------------

-------TCAC---ATAAAGAGGAAGT-GAGAA------ATAGCGCTAAG

GAAAATGAT---AGTCAAGATACCCCTTCATGAAACGAATTCTGGGG-TG

GC--TGC-----AAAGG---AAAACCAC---tttgtgcatagttgccaaa

aagaagaaatgaccagagtgtagatacata-----atgggtgtcgtc-aa

cagaagggtggcacacacgcg----ggactgatactaagccataaaatgg

gctgcaatgctaattaatgctgcactg-----------ggaa-atgttgt

gctaaataaaatag----gacacggagagaaat--attgtaggattccat

gtat----------------------------------------------

-------------------caggagctt-agggaagtaagggtt------

------gcttcatggttttgtttggggccaataaaaaa--gctttggaaa

tag----tggtggtaactggagagcaccgtgaatgtggttaatgctcctt

aagta---------------------------------------------

--------------------------------------------------

--------------------------------------------ggatta

aaatggcagacagtatgtta--tgacctttaccgcaattaaa--------

------GGA-CATACCTCAC---TTGCAAGGTTGTTGGCATCTTTCATAG

TTTTGTAGAGCTGGCTGTCTTTTGGGTTGTATTTGGGTGTTTTGCCTTTC

TCCTTGTCAAAGTTTTCTCG

>rhesusmonkey

AGCCTTATATTTAACCTATGTGTTAAGGGCGGAAGA--------------

AAGGAAATCAC---ATAAATAGGACAT--GGAA-------CAGCACTAAT

GAAAATCAA---AGTGAGATAGCCCA-TCATCAAACAAATGCTGGAGATG

G---TGAT----GAAAAACCATACC------tcatt-catagtagccaga

aggcagaaacaacccaagtgtctgtga------gcagatgcatggataaa

caaaa-tgtggcatatacatacaatggaatattatttagccataaaaagg

catgaagttctgatatatcctagaacatggatggaccttgaacatattat

gctaagtaaaataagccagacacaaaaggacaaatactgtgtgattccat

ttatatgcgctctctaaaataggaaaatgcagagagacaaaaaagtagat

ta--gaggttcccaggagctgacgggaggagggaatggagagtt------

tttgcttaatggttatagagtttctgttggtatga-aaacattttggaaa

tag----tgacagtagttggataatattgtgagtgttataaatgctactg

aattgcacac----------------------------------------

--------------------------------------------------

-------------------------------------ttaaaaacagtta

aaatggcaaataccatgtta--tatattttaccacaatttta--------

------GAAACATACCTCAC---TTGCAAGATTATTAGCATCTTTCATGA

CTTTGTAGAGCTGGCTGTCTTTTGGATTGTATTTTGGTGTCTTGCCCTTT

TCTTTATCGAAATTTTCTCG

>human

AGCCTTGTATTTAACCTGTGTGTTATGGGGGAAGA---------------

AAGGAAATCAC---GTAAATAGGAAAT--GGAA-------CAGCACTAAT

GGAAATCAA---AGTGAGATAGCCCA-TCGTCAAACAAATGCTGGAGATG

G---TGAT----GAAAAATCATCATACC---tcatt-catagtagccaga

aggcagaaacaacccaagtgtctgtca------gcagatgaatggataaa

caaaa-tgtggcatatacatacaatggaatattatttagccataaaa-gg

aatgaagttctgatatatcctacaacatggatggaccttgaacatattat

gctaagtaaaataagccagacaccaaaggacagatactgtttgattccat

ttatatgcactctctaaaacaggcaaatgcatagagacaaaaa-gtagat

aa--gaggttcccaggagctgacgggaggagggaatggagagtt------

tttgcttaatggttata--gtttctgttggtatga-aaacattttggaaa

tag----tgacagtagttggataatattgtgagtgttataaatgctactg

aattgcacac----------------------------------------

--------------------------------------------------

-------------------------------------ttaaaaacagtta

aaatggcaaataccatgtta--tatattttaccacaatttta--------

------GAAACATACCTCAC---TTGCAAGATTATTAGCATCTTTCATGA

CTTTGTAGAGCTGGCTGTCTTTTGGATTGTATTTTGGTGTCTTGCCCTTT

TCTTTATCGAAATTTTCTCG

>chimpanzee

AGCCTTGTATTTAACCTGTGTGTTATGGGGGAAGA---------------

AAGGAAATCAC---GTAAATAGGAAAT--GGA-------ACAGCACTAAT

GGAAATCAA---AGTGAGATAGCCCA-TCGTTAAACAAATGCTGGAGATG

G---TGAT----GAAAA---ATCATACC---tcatt-catagtagccaga

aggcagaaacaacccaagtgtctgtca------gcagatgaatggataaa

caaaa-tgtggcatatacatacaatggaatattatttagccataaaaagg

aatgaagttctgatatatcctacaacatggatggaccttgaacatattat

gctaagtaaaataagccagacaccaaaggacagatactgtttgattccat

ttatatgccctctctaaaataggcaaatgcatagagacaaaaa-gtagat

aa--gaggttcccaggagctgacgggaggagggaatggagagtt------

tttgcttaatggttata--gtttctgttggtatga-aaacattttggaaa

tag----tgacagtagttggataatattgtgagtgttataaatgctactg

aattgcacac----------------------------------------

--------------------------------------------------

-------------------------------------ttaaaaacagtta

aaatggcaaataccatgtta--tatattttaccacaatttta--------

------GAAACATACCTCAC---TTGCAAGATTATTAGCATCTTTCATGA

CTTTGTAGAGCTGGCTGTCTTTTGGATTGTATTTTGGTGTCTTGCCCTTT

TCTTTATCGAAATTTTCTCG

>tenrec

TGCCTTATATTTCACCTGCATGTGAAGACAGGAAA--------------C

CAGCAGATCACCTGGCGAGCAGGTAAT--GAAGG-----GCAGGGCCATG

GGAGAGCACCCGACCTGGATACCCGA-CCCGGAAACCGAGGCTGGGGAGG

GCAACAAAA-----------------------------------------

--------------------------------------------------

--------------------------------------------------

--------------------------------------------------

--------------------------------------------------

--------------------------------------------------

--------------------------------------------------

--------------------------------------------------

--------------------------------------------------

--------------------------------------------------

--------------------------------------------------

--------------------------------------------------

--------------------------------------------------

------GGACCATACCTCGC---TTGCGAGATTATTAGCATCTTTCATGA

TTTTGTAGAGTTGGCTGTCTTTTGGATTGTATTTTGGTGTCTTGCCTTTC

TCTTTGTCAAAATTTTCTCG

>elephant

TGCCTTATATTTAACCTGCATATGAAGGAAAAAAA---------------

-AGGAGATTAC---AAATACAGGAAAT--GGAAA-----ACAGTGCTGTT

GGAGATCAT---AGCGACATATCACA-TCGTGAAACAAATGCTGCAGAAG

GCAAGGA-------------------------------------------

--------------------------------------------------

--------------------------------------------------

--------------------------------------------------

--------------------------------------------------

--------------------------------------------------

--------------------------------------------------

--------------------------------------------------

--------------------------------------------------

--------------------------------------------------

--------------------------------------------------

--------------------------------------------------

--------------------------------------------------

------AAAACATACCTCAC---TTGTGAGATTATTAGCATCTTTCATGA

CTTTGTAGAGCTGGCTGTCTCTTGGATTGTATTTTGGCGTCTTGCCCTTC

TCTTTGTCGAAATTTTCTCG

>sloth

AGCCTTATATTTAACCTGCATATTAAGCAAAAAAA------------GAA

AAAGAAACCGC---ATAAATAAAAAAT--GGAAA-----AAGGCACTCAT

GGAAATCAT---AGTAATATATCCCA-TTCTGAGACAAATGTTGGAGCTG

GCAATGAA------------------------------------------

--------------------------------------------------

--------------------------------------------------

--------------------------------------------------

--------------------------------------------------

--------------------------------------------------

--------------------------------------------------

--------------------------------------------------

--------------------------------------------------

--------------------------------------------------

--------------------------------------------------

--------------------------------------------------

--------------------------------------------------

------AAACCATACCTCAC---TGGCGAGATTATTAGCATCTTTCATGA

CTTTGTAGAGCTGGCTGTCCTTTGGATTGAATTTTGGTGTCTTGCCCTTT

TCTTTGTTGAAGTTTTCTTG

>armadillo

--CCTTATATTTAACCTGCATATTAAGAAAAAAAAATAAAGGAAGCGGGG

AAAGAATTCAC---ATAAATAGGAAAT-GGGAAA-----AGAGCACTAAT

GGAAACCAT---AGCAATATATCCCA-TCCTGAGACAAATGGTGGAGCTA

GCAATGC-------------------------------------------

--------------------------------------------------

--------------------------------------------------

--------------------------------------------------

--------------------------------------------------

--------------------------------------------------

--------------------------------------------------

--------------------------------------------------

--------------------------------------------------

--------------------------------------------------

--------------------------------------------------

--------------------------------------------------

--------------------------------------------------

------AAACCTTACCTCAC---TGGCAAGATTATTAGCATCTTTCATGA

TTTTGTAGAGCTGGCTGTCTTTTGGATTGAATTTTGGTGTCTTGCCCTTT

TCCTTGTTGAAATTTTCTCG

>opossum

AGCCTTGTACTTAACCTGCAAATTAAAAAAAAAAA--------------A

GTTGAGGCTAC--CTCAGAGAGAAGATGAGGAAACTGTCTACTCTTTAAT

GTCAGCCAC---AGCAGGACACGCCA-CCCTGGAGGAGGATCTA-AGGTG

GCATCGAG------------------------------------------

--------------------------------------------------

--------------------------------------------------

--------------------------------------------------

--------------------------------------------------

--------------------------------------------------

--------------------------------------------------

--------------------------------------------------

--------------------------------------------------

--------------------------------------------------

--------------------------------------------------

--------------------------------------------------

--------------------------------------------------

------GGAACATACTTCAC---TTGCGAGGTTGTTGGCATCTTTCATGG

TCTTATAGAGCTGGCTATCTTTCAAGTTGTATTTTGGTGTCTTGCCCTTC

TCTTTGTCAAAGTTTTCTCG

Marker 3c:

>L1MB3

--------------------------------------------------

--------------------------------------------------

-----------------------atggatggtggtgatggttgcacaaca

atgtgaatgtacttaatgccactgaactgtac----acttaaaaatggtt

aaaatggtaaattttatgtta--tgtatattttaccacaa----------

-ttaaaaa--aa--------------------------------------

--------------------------------------------------

--------------------------------------------------

--------------------------------------------------

--------------------------------------------------

--------------------------------------------------

--------------------------------------------------

--------------------------------------------------

--------------------------------------------------

----------------------------

>cow

CACAACCAGCACGAAGGTTAGATCCACCATCCAGATTTTCAGGAAGAAAT

GACAGAGGGGATCAAGTGCCCAACAGAAAAGATGACCGAAGGTATG----

---TTTTT-AGAAGTATACT---tttgatagtggtgatggttgt---aca

gtgtgaacgaatgcacttaatgccactgaactgtaaacctcaaaatgatt

aaaatagattatt--atattg--tgtgtattttaccacagtgttcaagaa

gttatt----------TAAAAAT--GTACACC----GTGAAATGTTGCTT

ATATTTATACGTA--TGCTTATA----CATATTAAA-TTTACTT----CA

TTAAAGAAC-----------------------------------------

--------------------------------------------------

--------------------------------------------------

--------------------------------------------------

--------------TCCTTGATAATGCTA-GAAAAA---GTAATTTTAGA

AAAATTTGCTTT--AAGTCGTGAAAGAGAAAGAGAAAGACGTAGATCCAG

AGAAAGATCTCCTCAGAGAAAACGTTCCCGCGAGAGATCTCCTCGGAGAG

AGAGGGAACGGTCACCTCGGAGAGTCCG

>dog

CACAACCAGCACGAAGATTAGATCCACCATCCAGATTTTCAGGAAGGAAT

GATAGAGGGGATCAGGTGCCTAACAGAAAAGATGACCGAAGGTATT----

---TTT----AAAGTATACT---tttgatggtggtgatggttgc---aca

ttgtgaatgcacttaatgccac-gaattgt---atgtttcaaaa-tggtt

atggggggag-ttt-gtgtatca--catattgtacta-------------

-tttt-----------TAGAAGTTGTTAAACT------------------

ATAT-TAGAGAATCTTGCTTATATT--ATCTATTATATTTATTT----TA

TCAAATAAC-----------------------------------------

--------------------------------------------------

--------------------------------------------------

--------------------------------------------------

--------------TCTTCAGTAATGCTG-GCAATATTAGTAATTTTAGA

AAATTTTGTTTT--AAGTCGTGAAAGGGAGAGAGAAAGACGTAGATCCAG

AGAAAGATCTCCTCAAAGAAAGCGTTCCCGGGAGAGATCTCCTCGAAGAG

AGAGAGAACGATCACCTCGGAGAGTTCG

>mole

CACAACCAGCACGAAGGTTAGATCCACCATCCAGATTTTCAGGAAGGAAT

GACAGAGGGGATCAAGTGCCTAACAGAAAAGATGACCGAAGGTATG----

---TTTT-TAAAAGCAAGTT---tttgatgatggtgatgactgc---aca

atgtgaatatacttaatt-tactaaactgt---atacctaaaaatta-tt

aaaatgataa-ttttatg--tcatgtatattttaccacaa----------

-ttt------------TAAGTTATTTTTTTAAA------------TGC--

ATAC-TGTAGAATCTTGCTAGTATT--AGTTATTATATTTATTT----TA

TTAAAGAACTGGGAAAAACTGAATAGTAAAGACTATAAAATAACTTGTTT

TGAGGGGTGGCTATATAGGGTAAACTAGGTCAGTTGTGTGGTAACGGATG

GAATTAGACTTTGATGGTAAGCTTAATATGGTATATACAGATGTAGATTT

ATAATGATGCACATCAAAAACTAACAATGCAGTGCTACATCAATAAAATG

TACAAAAAAACAGAACTTTGATAGAGACA-GAAGTATTGGTAATTTCAGA

AAATTTTGTTTT--AAGTCGTGAAAGGGAGAGAGAAAGACGTAGATCCAG

GGAAAGATCTCCTCAGAGAAAACGTTCCCGGGAGAGATCTCCTCGAAGAG

AGAGAGAGCGATCACCTCGAAGAGTTCG

>rabbit

CACAACCAGCGCGAAGATTAGATCCTCCATCCAGATTTTCAGGAAGGAAT

GACAGAGGGGATCAAGTGCCCAACAGAAAAGATGACCGAAGGTATG----

---TTTT-AAAAATTATACC---gtggctgatggtgat-taggtatataa

caataaatgtacttagaa----tga-catttttt-------aaagct-ta

tgg-tagtaaattggatgtta--tgtctgttttaccacaa----------

-tttt-----------TTAAAATT-GTGAAT-------------------

-----------------CTTTTATT--ACCTATTAGATTTGTTT----CA

TTAAAGAAC-----------------------------------------

--------------------------------------------------

--------------------------------------------------

--------------------------------------------------

--------------TTTTCCATAATGCCA-G-AATATTAGTAATTTTAGA

AAAATTTGCTTG--AAGTCGTGAAAGGGAAAGAGAAAGACGGCGATCCAG

AGAAAGATCCCCTCAGAGGAAGCGTTCCCGGGAGCGCTCTCCCCGGAGAG

AGAGAGAACGATCGCCTCGGAGAGTTCG

>human

CACAACCGGCACGACGATTAGATCCCCCATCCCGATTTTCAGGAAGAAAT

GACAGAGGGGATCAAGTGCCTAACAGAAAAGATGATCGAAGGTATA----

---TTTTTCTAAAGTGTACT---gtggatggtggcaatggttgtacaaca

atggaaacatagttaatgccactgaactatgcttataatataaaatt-tt

aaaatggtaaattttatgtta--tgtctattttactacaa----------

-ttt------------TTAAAAA--GTTAAAC--------------AAAT

ATAC-TGTGGATTCTTGCTTATATT--ACCTACTCTATTTACTT----CA

TTTGGGGAAGAG--------------------------------------

--------------------------------------------------

--------------------------------------------------

--------------------------------------------------

-------------CTTTTCCATAATATTA-GAAATATTAGTAATTTTAGA

AAAATTTGCTTT--CAGTCGTGAGAGAGAGAGAGAAAGACGTAGATCGAG

AGAAAGATCACCTCAGAGGAAACGTTCCCGGGAAAGATCTCCACGAAGAG

AGCGAGAGCGATCACCTCGGAGAGTTCG

>chimpanzee

CACAACCGGCACGACGATTAGATCCCCCATCCCGATTTTCAGGAAGAAAT

GACAGAGGGGATCAAGTGCCTAACAGAAAAGATGATCGAAGGTATA----

---TTTT-CTAAAGTGTACT---gtggatggtggcaatggttgtataaca

atgtaaacatagttaatgccactgaactatgcttataatataaaatt-tt

aaaatggtaaattttatgtta--tgtctattttactacag----------

-ttt------------TTAAAAA--GTTAAAC--------------AAAT

ATAC-TGTGGAATCTTGCTTATATT--ACCTA---------CTCTATTCA

TTGAAGAGC-----------------------------------------

--------------------------------------------------

--------------------------------------------------

--------------------------------------------------

--------------TTTTACATAATATTA-GAAATATTAGTAATTTTAGA

AAAATTTGCTTT--CAGTCGTGAGAGAGAGAGAGAAAGACGTAGATCGAG

AGAAAGATCACCTCAGAGGAAACGTTCCCGGGAAAGATCTCCACGAAGAG

AGCGAGAGCGATCACCTCGGAGAGTTCG

>tenrec

CACAACCAGCACGAAGGTTAGACCCCCCATCCAGATTTTCTGGAAGGAAT

GACAGGGGGGATCAAGTACCTAACAGAAAAGATGACCGAAGGTATG----

--------------------------------------------------

--------------------------------------------------

--------------------------------------------------

----------------TTAAAGTCTCTATTG-------------------

-------TGGCCATTTGTTTTTGTT--AC-CATTACA----CTTTATTTA

TTAAAGAAC-----------------------------------------

--------------------------------------------------

--------------------------------------------------

--------------------------------------------------

--------------TCTTCCATGTTGCCAAGAAATGTTAGTAATTT-AAA

TTTTTTTTCTTTT-AAGTCGTGAAAGAGAGAGAGAAAGACGGAGATCCAG

AGAAAGATCTCCTCAGAGAAAGCGTTCGAGGGAGAGATCACCCCGGAGAG

AGAGAGAGAGATCACCTCGGAGAGTTCG

>elephant

CACAACCAGCACGAAGGTTAGATCCACCGTCCAGATTTTCAGGAAGAAAT

GACAGAGGGGATCAAGTACCTAACAGAAAAGAGGACCGGAGGTATG----

--------------------------------------------------

--------------------------------------------------

--------------------------------------------------

-----------------TCAGTAG-GTATACT------------------

------GTGGAGTCTTGTTTGTGTT--AC-CATTATA----CTTACTTTA

ATAAAGAAC-----------------------------------------

--------------------------------------------------

--------------------------------------------------

--------------------------------------------------

--------------TCTTCCATAATGCCAA-AAATGTTAGTAATTTTAGA

AAACTTTGCTTT--AAGTCGTGAAAGGGAGAGAGAAAGACGGAGATCCAG

AGAAAGATCGCCTCAGAGAAAACGTTCCCGGGAGAGATCTCCCCGAAGAG

AGAGAGAGAGATCACCTCGGAGGGTTCG

>armadillo

CACAACCAGCACGAAGGTTAGATCCACCATCCAGATTTTCTGGAAGGAAT

GACAGAGGGGATCAAGTACCTAACAGAAAAGATGACCGAAGGTATA----

--------------------------------------------------

--------------------------------------------------

--------------------------------------------------

----------------TTTCAAAA-GTACACT------------------

------GTGGAATTGTGTTGTTCTT--AT-TACTTACTATATTTACTGCA

TTAAAGAGC-----------------------------------------

--------------------------------------------------

--------------------------------------------------

--------------------------------------------------

--------------TCTTCAAAATGCCCA-GAAATATCAGTAATTTTAGA

AAATTTTGTTTTTTAAGTCGTGAACGAGAGAGAGAAAGACGTAGGTCCAG

AGAAAGATCACCACAGAGAAAACGTTCCCGGGAAAGATCTCCCCGAAGAG

AGAGAGAACGATCCCCTCGGAGAGTTCG

>opossum

CACAACCAGCACGAAGGTTAGATCCACCATCCAGATTTTCAGGGAGAAAT

GACAGAGGGGATCCAGTTCCTAACAGAAAAGATGACAGAAGGTACGGTG-

--------------------------------------------------

--------------------------------------------------

--------------------------------------------------

----------------TTTCAGAAAGTGTGGC------------------

----AGGACCTCTTCGTTGTTTGTTGGACCTGTCAAAA-TGTTT--TTCT

TTAAAGATT-----------------------------------------

--------------------------------------------------

--------------------------------------------------

--------------------------------------------------

--------------TGCTTTCTTAAGCTAA-AAATAAAAGGAATTTTT-A

TTACTTTTCTTAT-AAGTCGTGAAAGGGAGCGAGAAAGACGTAGATCTCG

GGAAAGATCACCTCAGAGGAAACGTTCCAGGGAGAGATCTCCCCGAAGAG

AGAGAGAGCGATCACCTCGAAGACCACG

Marker 3d:

>L1MB5

--------------------------------------------------

--------------------------------------------------

--------------------------------------------------

--------------------------------------------------

atncatacaatggaatattattcagcca---taaaaaggaatgaagtact

gatacatgctacaacgtggatgaa----cctcgaaaacattatgctaagt

gaaagaagccagacacaaaaggcc--------------------------

--------------------------------------------------

--------------------------------------------------

--------------------------------------------------

----------------------acatattgtatgattccatttatatgaa

atatccagaataggcaaatccatagagacagaaagcagattggtggttgc

ca--ggggctggggggaagggggaatggggagtgactgcttaatgggtac

ggggtttccttttggggtgatgaaaatgttctggaactagataga-ggtg

gtggttgcacaacattg---------------------------------

--------------------------------------------------

--------------------------------------------------

--------------------------------------------------

----------------------------------tgaatgtactaaatgc

cactgaattgtwcactttaaaatggttaattttatg-ttatgtgaatttc

acctcaa----tt---twaaaaa---------------------------

--------------------------------------------------

--------------------------------------------------

--------------------------------------------------

-

>dog

CGAGCATCCAAAGGCTGATTTTTGTTTTGTTCCTGATTACATTTTCGATT

TCCTCCGCCTGAGCTTATA-CTTTTAGTTTGTCCCACAGAACTTGAAGCA

GTAGTGGATAATACAGGTGTGTTGATACCAGATACAGATGATGTACTGTT

TCCATTTATTGACCCATTTACACC---TTGAAAATAAAAACAAACAT---

gtttatccaagagaatattcttcagtca---taaaaaggaatgaagtaat

g-atgtatgctataacatgaagtga-ctctctgaaatatcacac-taagt

gaaaaaaagtcaga--caagaagtc-------------------------

--------------------------------------------------

--------------------------------------------------

--------------------------------------------------

----------------------acatactgtatgattccatatatatgaa

atagtcaca----gtaaatctgtagggatggtgcacaagctagtgattgc

ca--gagaatgaggaaaagaagaatggagaaaaactgcttaaggggtatg

gagttttcttttagagtgatgagatgttttgaaactacacaga----tgg

tgggtgttcaacac-ca---------------------------------

--------------------------------------------------

--------------------------------------------------

--------------------------------------------------

----------------------------------tgaatgcattaaatgc

cactga-ttgttcactttaaaaagg-taatttgatatttatctgaattta

acttcaa----TT---AAACAAAT------GTTTAGAAATATAATTAAAT

GCGGACATTTCCCTTTTCAGAG-CAAACTCAC-CAAAAACTCT-------

--TATCCAAATGAAAAAAGA---TAAAAATAAACCATCCAAAATGCTATT

AGAA-TTTGTAAATGCTATTCTAACA--ACAGTTATCAACTGCAGAAATA

T

>bat4b

CTAGCATTCAAAGGTTTTTTTTTATTTTGATCATGTTTACATTTTCGATT

TCCTCCACCTGAGCTTATTACTTTTAGTTTGTCCAACGGCACTGGAAGCA

GTAGTGGATAGTACAGATGTGTTGATACCAGACACAGATGAAGTACTGTT

TCCATTTTTTGACCCATTTACACC---TTGAAAAAATAAATAAAAAT---

gtttatataatagaa--ttcatttgaccacataaaa--gaatgaagtaat

g-ctatatgctctaatgtgatgattctcgaaaacataatgcaaagtgaaa

gcgtcacaaattgtacgataagatc-------------------------

--------------------------------------------------

--------------------------------------------------

--------------------------------------------------

-------------------------tat-gagatattcacaacaca-taa

at------------------------------------------------

--------------------------------------------------

-------ccttttgggatgatgaaaatgttttgaaactacatggagg-tg

ccaaaaccggtt-tggctcagtggatagagcgtcggcctgcggactcaaa

ggtcccaggttcggttccggtcaagggcatgtacctgggttgcaggcaca

tccccagtaggagatgtgcaggaggcagctgatcgatgtttctaactatc

tctctcctttcctctctgtaaaaagtcaataaaatatatatatataaaaa

aaaactacatggaggtgatgcttacacaatgatgtgaatgtattaagtgc

cactgaattattcactttaaaatggttaattttacatttatgtgatttac

acttcaa----TTTTTAAAACATCAA---------GAAATGTGGGA----

---------TCC---TCTAGAGTCGACTGCAG-CA-----TCTGTCTTA-

----------TAAAAAAAG-------------------------------

--------------------------------------------------

-

>hedgehog

CTAGCATCTA-AGGCTGGCTTTTGTTTTGTTCCTGAT-ACATTTTCGACT

TCCTCCACCTGAAGTTTATACTTTTAATTTGTCCCACAGAACTTGAAGCA

GTAGTGTATAGTATAGATGTGT-GACACCAGATACAGATGATGTACTGTT

TCCATTTACTGACCCATTTACACC---TTGCAAAAAAAA-CAAACAA---

aaaacatgtttaataacccagaatatta---ttcagaca--tgaaaggaa

atgaagtaatgatgtacactaaaatattagggactctaaaaca-ttaagt

taag------cataagaaattagtc-------------------------

--------------------------------------------------

--------------------------------------------------

--------------------------------------------------

----------------------acagagtcatatattatataatcacatt

ag-----------gtaaattcttagaaaaa--gtgaaaggtggtatttat

cagggactaggggattgaagaatgtgga--gaaatta-cttaacgcttat

agagtttcctttaagggtgacaaaagtgtttaaaaactttatagaggtag

tgattg-tataacactgt--------------------------------

--------------------------------------------------

--------------------------------------------------

--------------------------------------------------

----------------------------------tgcatc---taa-tac

tactaaattgatcactttaaaatggttaa--tgtt--tgtgtgaatttta

ctttgat----TA---AAATT---------TTTCAGAAAGATCGTTAAAT

GCAATTAATACCCCTTTTAGAGACATTTTCTT-CAAATGCATACTAAG--

---------GTGGAAAGAGAA----------CTA-GTGGTCCAGGAGGTG

GCGCAGTGA---------TAAAGCTTT-GGACTC--TCAAGCATGAGGTT

C

>shrew

CTAGCATCTAGAGACTGGTTTTTATTTTGTTCCTGATTACATTTTCTATT

TCCTCCACCTGAGCTTATA-CTCTTAGTTTGTACCATAGAACTTGAAGCA

GTAGTGGACAGTACAGATGTGTTGAGACCAGATACAGATGATGTACTGTT

CCCATTTATTGATCCATTCACACC---TTGAAAATAAAAACAAAGAT---

acttacgtaatagactattattcagcta---taaagacaggattgaataa

taaatgagtcttggaaacattaacttaaaccaaagaagttcaacacaaag

gctcacatactgtaagaa-tacatct------------------------

--------------------------------------------------

--------------------------------------------------

--------------------------------------------------

----------------------atatgagagagg----caaaa---tga-

--------------taaatctctagagactgagtacaagtttgtgactgt

cacggagcacaggagggaggaaggtta---gaaatgg-cctaacagatat

gcagttttctttttaggtgatgaaaatgttttaaaactagatagagggaa

tcattg-cacaaaactgt--------------------------------

--------------------------------------------------

--------------------------------------------------

--------------------------------------------------

----------------------------------taactgtactaaatgc

cactaaattgtttgctttaacatggttaattatatatttatgtgaatttc

acttcaa----TA---GAA-----------TGTTAGAAAGATATAAATGC

AAGAAT----CCCCTTTCAG---CACATATCCC--TAAACA---CGAA--

---------GTGAAAAGATAA-----GCAT-CTAAATGCTGCTGTATTGC

AGTGATTA-GTAACAATACAAATATT--GTCATTCCATGAAGGG-AG-TG

A

>mouse

CTGGCGTCCACAGGCTGGCTTTTGTTTTGTTCCTGATGACATTTTAGGGT

TCCTCCACCTGAGCTTGTG-CTTTGGTTCTGTCCCATGGAACTTGAAGCA

GGAGTGGCTACTACAGATGCATTGACACCAAGTACTGATGCTGCACTGGT

TCCATTAACCGACCCATTTATACC---TTG------AAAATAAACAG---

ttttatatattgggacagtatttggcca---gaacaaggagtaggg----

------tatg--ca-ttgttgtgt----atgtgcaactgtg------agt

gaaaaaaga-------------gtcacggggctggagagatgactcagcg

gttaagagcactgactgctcttccagaggtcctgagttcaattcccagca

accacatggtgtctcacaaccacctataagaagatctgatgccctcttct

ggtgtgtctgaagacagctacaatgtactcatataaataaaataaataaa

tcattaaaaaaaaaagaaaaaa------gatactcactaaaagtgacata

acattcctcca-catgaaggattcatcatgcatactgtagaaacaagata

caaactaat-gctcacaatgggcctgggaggggtttattttaaagggtga

agaa----ctgttg--gagataaaaa--ttttagagtatgttggagggtg

gccaatatgcaacacta---------------------------------

--------------------------------------------------

--------------------------------------------------

--------------------------------------------------

----------------------------------tgaacgcagtaaattc

aactgaataattcac-----------tagcattaacctga-gtggctatc

atagacg----TT---AAACAGA-CAT---GTTTAGAAAACAAAT-----

-CACTTGTAAG------------------CATTGTC--TGTTT-AGAAAC

AC----TCAGGGCTAGCTACAAC----------------------ACTGC

---AACTTCTAAACACTCTTGTAACA--ATACTTAGAGTCCACAGAGATC

C

>rattus

CTGGCATCCACAGGCTGGCTTTTGTTTTGTTCCTGATGACATTTTAGGTT

TCCTCCACCTGAGCTTGTG-CTTTGATTCTGTCCCATGGAACTTGAAGCA

GGAGTGGACAGTCCAGATGTATTGACACCATGTACAGATGCTGCACTGTT

TCCATTAACTGACCCATTTATACC---TTA------AAAATAAACAG---

gtttatatattgggacaacatt-ggcca---gaacaagaaatagag----

------tgtg--ta-ttgctggta-----tgtagaactgtg------agt

gaaaaaaga-cact----aaaagcc-------------------------

--------------------------------------------------

--------------------------------------------------

--------------------------------------------------

---------------------------acatatcattgc-tctccatgaa

agattcataatgcata---ctgtagagacaaggtacaaagtcat-cctta

caatgggcctgggag-------------gggtgattattttaaagagtgc

agaa----ttgttg--gaggtaaag---ttttagagtgtgttggagggtg

gccaatatacaacacta---------------------------------

--------------------------------------------------

--------------------------------------------------

--------------------------------------------------

----------------------------------tgaacatattcaat-c

agctgaattattcac-----------tagcattaaactga-gtgaatatc

acggaag----TT---AAACAAA-CAT---GTTTAGAAAGCAAAT-----

-CACATGTAAG------------------CATTGTC---CTTT-TAGAAC

AC----TCAGTA-TGAATAAG-----CACTGGGCTAGCTAAAA-CACTGC

---ACCTTCTAAACACTCTTGTAACA--ATACTTAGAGACTACAGACTAC

A

>rhesusmonkey

CTAGCATCCAAAGGCTGGTTTTTGCTTTGTTCCTGATTACATTTTCGATT

TCCTCCACCTGAGCTTGTA-CTTTTAGTTTGTCCCATGGAACTTGAAGCA

GTAGTGGATAGTACAGATGTGTTGATACCAATTACAGATGATGTATTATT

TCCATTTATTGACCCATTTACTCC---TTGAAAATAAAAATAAACAT---

gcttatacaatggaatattattgcatcatttaaaaatgaagtacag----

---atatatgtacaattggatgaa-----cttgaaaacatgg---taagt

gaaagaaga-caga----aaaggtc-------------------------

--------------------------------------------------

--------------------------------------------------

--------------------------------------------------

----------------------atct--tgtatgattccatctatatgaa

atattcggaataggtaaacctatagggacaaagtgaaaactgatggtttc

ca--ggggctgagggagaggggaatggggagcaactg-cttaatgggtat

aaagtttccttttgcagtgataaaaatgtttcagaagaagataga-ggtg

gtg-ttgtacaacattg---------------------------------

--------------------------------------------------

--------------------------------------------------

--------------------------------------------------

----------------------------------caaaagcattaaatat

cactaaattattcgctttaagatggttaatattatgttaa-ctgaatttc

acctcaa----tt---AAACAAA--AT---GTTTAGAAAGATAATGAAAT

GCAAATGTTCTCCTTTTAGAACA--------------AACTTTGTAAAAC

AC---TTAAGTGAAAAGA--------TAATAAACCATCTAAAA-TGTTGT

TATAATTTTCAAATTATATTCTAACA--AGTTATTAATTGCAGAAATATT

A

>human

CTAGCATCCAAAGGCTGGTTTTTGCTTTGTTCCTGATTACATTTTCGATT

TCCTCCACCTGAGCTTGTA-CTTTTAGTTTGTCCCATGGAACTTGAAGCA

GTAGTGGATAGTACAGATGTGTTGATACCAATTACAGATGATGTATTACT

TCCATTTATTGACCCATTTACACC---TTGAAAATAAAAATAAACAT---

gtttatacaatggaatattattgcatcatttaaaaatgaagtacag----

---atatatgtacagttggatgaa-----cttgaaaatatga---taagt

gaaagaaga-caga----aaaggtc-------------------------

--------------------------------------------------

--------------------------------------------------

--------------------------------------------------

----------------------atgt--tgtatgattccatctacatgaa

atattcagagcaggtaaacctacagggacaaagtgaaaactgatcgcttc

ca---gggctgagagagaggggaatgggaggcaactg-cttaatgggtac

aaagtttccttttgcagtgataaaaatgtttcagaacaagataga-ggtg

gtggttgtacaacactg---------------------------------

--------------------------------------------------

--------------------------------------------------

--------------------------------------------------

----------------------------------caaatgcattaaatac

cagtaaattattcactttaagatggttaatattatgttaa-gtgaatttc

acctcaa----TT---AAACAAA--AT---GTTTAGAAAGATAATGAAAT

GCAAAACTTTGTGTTTTAATTGTAAAACACATTAGAGAACTTTGTAAAAC

AC---TTCAGTGAAAAGAAAGA----TAGTAAATCATCTAAAA-TGTTGT

TATAATTTTCAAATTATATTCTAACA--AGTTATTAATTGCAGAAATATT

A

>chimpanzee

CTAGCATCCAAAGGCTGGTTTTTGCTTTGTTCCTGATTACATTTTCGATT

TCCTCCACCTGAGCTTGTA-CTTTTAGTTTGTCCCATGGAACTTGAAGCA

GTAGTGGATAGTACAGATGTGTTGATACCAATTACAGATGATGTATTACT

TCCATTTATTGAGCCATTTACACC---TTGAAAATAAAAATAAACAT---

gtttatacaatggaatattattgcatcatttaaaaatgaagtacag----

---atatatgtacagttggatgaa-----cttgaaaatatga---taagt

gaaagaaga-caga----aaaggtc-------------------------

--------------------------------------------------

--------------------------------------------------

--------------------------------------------------

----------------------atgt--tgtatgattccatctacatgaa

atattcagagcaggtaaacctacagggacaaagtgaaaactgatggtttc

ca---gggctgagagagaggggaatgggaggcaactg-cttaatgggtac

aaagtttccttttgcagtgataaaaatgtttcagaacgagataga-ggtg

gtggttgtacaacactg---------------------------------

--------------------------------------------------

--------------------------------------------------

--------------------------------------------------

----------------------------------caaatgcattaaatac

cactaaattattcactttaagatggttaatattatgttaa-gtgaatttc

acctcaa----TT---AAACAAA--AT---GTTTAGAAAGATAATGAAAT

GCAAAACTTTGTGTTTTAATTGTAAAACACATTAGAGAACTTTGTAAAAC

AC---TTCAGTGAAAAGAAAGA----TAGTAAATCATCTAAAA-TGTTGT

TATAATTTTCAAATTATATTCTAAAA--AGTTATTAATTGCAGAAATATT

A

>elephant

CTAGCATCAGA-GGCTGC--TTTACTTG-TTCCTGATA-CATTT-CGATT

T-CT-CAC-TGAGCT-ACA-GTTTTAGTTTGTCCCGTGGAACTTGAAGCA

GTAGTGGATAGTACAGATGTGCTGATACCAGATACAGATGATGTACTGTT

TCCATTTATTGACCCATTTACACC--------------------------

--------------------------------------------------

--------------------------------------------------

--------------------------------------------------

--------------------------------------------------

--------------------------------------------------

--------------------------------------------------

--------------------------------------------------

--------------------------------------------------

--------------------------------------------------

--------------------------------------------------

--------------------------------------------------

--------------------------------------------------

--------------------------------------------------

--------------------------------------------------

--------------------------------------------------

--------------------------------------------------

-----------TTGAAAAAAAATACAC----GTTTAAAAAGGTAATTAAA

TGAAAATATTTCCCC----------------TTA--AAACTCTCCAAAAC

AC-TTACTAGTCAAATGAAAAGACATTAATAAGTCATCTAAAAATGCTAA

TATAATTTTCAAATGATATTCTAATA----AAACCAAAACCAAACCCACT

-

>armadillo

CTAGCGTCCAAAGGTGGGTTTTTACTTGGTTCCTGATTACATTTTCGATT

TCCTCCACCTGAGCTTATA-GTTTTAGTTTGTCCAGTGGAACTTGTTGCA

GTAGTGGATAGTACAGATGTGCTGATACAAGATACAGATGATGTACTGTT

TCCATTTATTGATCCATTTACACC--------------------------

--------------------------------------------------

--------------------------------------------------

--------------------------------------------------

--------------------------------------------------

--------------------------------------------------

--------------------------------------------------

--------------------------------------------------

--------------------------------------------------

--------------------------------------------------

--------------------------------------------------

--------------------------------------------------

--------------------------------------------------

--------------------------------------------------

--------------------------------------------------

--------------------------------------------------

--------------------------------------------------

-----------TTAAAAAAC-----AT---ATTTAGAAAGA-TAATTAAT

TGCAAATA-TTCCT--CTTTGAAA----------GGAAACTCCCCCAAAC

ACCATTCAAGTGAAATGAAAGAT--AATAACAAACCATATAAAAGAATAT

TAAAAATTTCAAATGATATTCTAACAATACTGATCAATTCCAGAAATATT

A

Marker 3e:

>L1MB3

--------------------------------------------------

--------------------------------------------------

--------------------------------------------------

--------------------------------------------------

--------------------------------------------------

--------------------------------------------------

------------------tttaatgggtacagagtttcagtttgggaaga

tgaaaaa-gt----tctgga-gatggatggtggtgatggttgcacaacaa

tgtgaatg----tacttaatgccactgaactgtacacttaaaaatggtta

aaatggtaaattttatgtt-atgtatattttaccacaattaaaaaaa---

--------------------------------------------------

--------------------------------------------------

--------------------------------------------------

--------------------------------------------------

--------------------------------------------------

--------------------------------------------------

--------------------------------------------------

--------------------------------------------------

--------------------------------------------------

--------------------------------------------------

--------------------------------------------------

--------------------------------------------------

--------------------------------------------------

----------------------------------

>dog

TACGGGCAACTCACTGAATGAACCCGAAGAGCCCTCAGACATCCCGGAGA

GGAGGGATTCTGGGGTTGGGGCCTCCCTGACAAGGTCCAACAGGTGAGCA

CTTTGCTTCTGTC-TGGAGATAGGGGAAGGAG-ACCAGGAAAGGGACTGC

TTCTGCTCTT--GGGGGTGGGTAGGG---GGGTGGAATGGGTCACATCTG

GCATTCATAGCCCTGTGGGTCCCAAAGAAAAGTGATA--GACCAGAAGAT

C----CCAACATCCAAAA--------------------------------

---CCATGGATATTG---tttc-tgggtatagagtttcagtttgggaaga

ggaggtaggt----cttatg-gatgattggtggtgatggttgcac---aa

-gggggtgggggtacttaatgcccctgagctgtacacttagcagcagtta

agatgatcatgtatataat-gtg--tattttaccacagc-----------

----------GGGGGGAGG-AAAAGAATG------GAATGAAAG-TCGAG

TCCTCTCTGTT----------CCAGTGA-------------------GCA

G-CCACTGCTGATTTT-GCATCTACCTGTTGAAAAGTCCTTG--------

--------------------------------------------------

--------------------------------------------------

--------------------------------------------------

--------------------------------------------------

--------------------------------------------------

--------------------------------------------------

--------------------------------------------------

--------------------------------------------------

--------------------------------------------------

-------------------------------------------TACT--C

ACTCCCTCCCCTGCTCTTCTCCTGCAGGCACAGG

>pangolin

CACGGGCAACTCGCTGAACGAACCCGAAGAGCCCTCGGACATCCCGGAAC

GGAGGGATTCTGGGGTCGGGGCCTCCCTGACAAGGCCCAATAGGTAAGAG

CTGGTCTTCCACC-TCCAGACTGGGGGGTGGGAGTGGGGCCAGAAAGTGC

----------------GGGGGAAGCC---AGATGAAGTCCGGCATTCAGC

GCCATGTGAGTT---------CCTAA-----GTGACA--AACCAGGAAAA

TC---CTACCATACAAAC--------------------------------

-AAGAATGGATATTG---attaatgggaacagagtaatagtttgggaaga

tgaaataagt----tccgtg-gatagatggtggtgatggttgtacaacac

gaa-------catacttaatgccgctccactgtacgcttaaagatgggta

agacggtaaattttatggt-atgtatattt-accaca-------------

----------GGGAGAGAA--AATAGCGT-----GGAATGGAAG-TTGGG

TCCT-----------------CCAGTGCTTCTGATAAGAAGCCAGTGGGC

AGCCTGCTGCCGATTTTGCGAGTACCTCCTGCAAAGCTTTTG--------

--------------------------------------------------

--------------------------------------------------

--------------------------------------------------

--------------------------------------------------

--------------------------------------------------

--------------------------------------------------

--------------------------------------------------

--------------------------------------------------

--------------------------------------------------

-------------------------------------------TACC--C

ACGCTCTTCCCCTCTCTTCCCCCGCAGGCACAGG

>shrew

CACAGGCAACTCACTGAATGAGCCAGAGGAGCCCTCAGACATCCCCGAAA

GGAGGGATTCAGGCGTCGGGGCCTCCCTGACCAGATCTAATAGGTGAGAA

GATCTCTTCTCCC-TCCAGAATC-TGGGT------------AGAGGGGA-

CCCCTCCTGC---TTAGCGGGTCTT---GGG-TCAATGTGGGCCTTCACA

GCCTTATCAGTT---------CATGATGAGAGCAACC--AGCCAGGAGCA

TC---CGACT-TCCAAGA--------------------------------

--AGAGTGGGTGCTG---tttgaggagaacttagtttcagatagggggc-

tgg-taaagtcagctctgtg-gatggatggcgatggtggtagtagt--ac

ca------tggtggtaatatcctggcccctgcctcccc-aaagggat-cc

aatagttca----tggaatggtttatatgtctcct---------------

----------CAGAAGAAG--TATGAGAA------AAATGGAA------T

TTCCATCCTCA----------CGTGCAAG--GGTGCAACCTCAGTGAGAA

ATCCCTACAGGTTTTATGTT-GAGG-CCTTG----GTCTTTC--------

--------------------------------------------------

--------------------------------------------------

--------------------------------------------------

--------------------------------------------------

--------------------------------------------------

--------------------------------------------------

--------------------------------------------------

--------------------------------------------------

--------------------------------------------------

-------------------------------------------TCT----

CACCCTCTCCTGCCCTTCTCTCTGCAGGCACAGA

>mole

CACAGGCAACTCACTGAATGAACCAGAAGAGCCCTCAGACATTCCAGAAA

GGCGGGATTCTGGTGTTGGCGCCTCCCTGACAAGGTCCAATAGGTAAGAG

CTTTGTGTCTCTC-TGCAGGAT------------------CAGAAA---G

CTGTTGCTCGT---ATAGGGGCCTT---GGGGTTAAGTGTGGCATTCAGT

GCCGCAATAGA----------CCAGGGAGGAAAGTGACAGGCCAGAAAGT

CC---TAAC-ATCAAAAA---------------------------AAAAA

AAAAAATGGGTTTAA---gttt-ggaaa--------------------gg

tgaagtaagt----tcggtg-ggtgggtggtgtggatggttggacatcac

ctgaa------ctgtac-tttta-------aaagtggttaa-gatga-ta

aa---------ttttaggttctgtatattctatcat--------------

----------GGGGTGGCA-----GGGGT---GGAGAATGGAATGTTGAT

TTCTCTCATTT-------------CCTAA--GATGAGAGGCCAATGAGCA

ACCCTCTGCTAATTTTGTAGCTACCTGTT--AAAGCCTTTA---------

--------------------------------------------------

--------------------------------------------------

--------------------------------------------------

--------------------------------------------------

--------------------------------------------------

--------------------------------------------------

--------------------------------------------------

--------------------------------------------------

--------------------------------------------------

-------------------------------------------TCTCACT

CACTTTCCTCTGCTCTTTTCTCAT-AGGCACAGA

>mouse

CACAGGCAACTCCCTCAATGAGCCCGAAGAGCCCACTGATATCCCGGAAA

GAAGAGACTCAGGGGTGGGGGCTTCCCTGACCAGGTGCAATAGGTAAGGA

AGGGCGTTGCTTC-TCGATGCATCCAGAGGTGGAGAATCAAGAAGGG---

-TCT---GCTTGGT--------------GGGATTGAGTCTGATATTTGCA

GTCCTGTAAATT---------CCTAGGGACGGCGTCC--AACCAGGAGAC

CC---CAACAATCCCAACGGGAAAGGAGTATTATAAA-------------

----CTAGGTATGAA---cctt-tggtcatc--------------aagga

tgcagacagtggaccctggaagatggtggtgtttgaacg-atatagtcag

gccttat-ccaccgtggggtgt---acttagacgtgcttaaag-tgcatg

ca----------tcttgattctcctgcagttccaaatct-------TCAG

TTTCAGCCAGGCACAGATGAGAACTACTC----AGGGGAGAAACTGTCTT

CTCCGTCATTATAC----------CCTGGGTAATAGAGTGTGACCGTGAA

CTACTAGCAGGTTGTTATAGCAATCTGGCTTATAAACTTACATTAAAT--

--------------------------------------------------

--------------------------------------------------

--------------------------------------------------

--------------------------------------------------

--------------------------------------------------

--------------------------------------------------

--ggggagggtgctcccgatgtgcgttgacactatccatcttttttaaga

gacctgagtgtactgagtccacatatctgctatgttctggaaccaacctt

caggggttacaaagacagt----gggggtgggggggaggcagggaaagga

agatcgatgctcttggt-tcctgatgat-cagagatt---GGTCCCAGCT

CACTCCTTCCGCCCTGTTCTCTTGCAGGCACAGA

>rat

CACAGGCAACTCCCTCAACGAGCCCGAGGAGCCCACCGATATCCCTGAAA

GAAGAGACTCAGGGGTGGGGGCTTCCCTGACCAGGTGTAATAGGTAAGGC

CCTTACTTCC-GGCTCCAGACT----GAGGTG-GGGATCAAGAAGGG---

-TCT---GCTTGGC--------------GGGATCGAGTCTGACATTCGCA

GTCCTGTAAATT---------CCTAG------CGCCC--GACCAGGAGGA

GACCCTAGCAATCCCAAAGAAGGGAGGGTATTGTAAACCTTT------GG

TCATGAAGGATGCAG---acag-tggatcc--------------------

----------------tggaaggtggtggtgtttgcaca-atataatcgg

actttatgccaccctggggtgt---acttagaagtggttaaaa-tggtCT

AAgtgcctgtacacttggttttcctgcagctccaaatct-------TCAG

ACTCTGCCAGGCACAGACGAGAAATGCTC----AGGGGAGAAACTGTCTT

CTT-GTCACTTAGCAGGTTATTATAAGCAATCGGGCTTATAAACTTGCAC

TGAGTAGGAGGGCGCTCCCG-----ATATGCATAGACATTGT--------

--------------------------------------------------

--------------------------------------------------

--------------------------------------------------

--------------------------------------------------

--------------------------------------------------

--------------------------------------------------

-----------ctgtcttctgtaa-gaggtctaagaggcccgagtgtccc

ga-gt-----ccacatatctgcagtgctctaga-accaaccttcggggtt

acataggaagggctattttacaatggagggaaatcgggaaaggagtg-tc

agtgctgggggtccctgtgagcagcttc-tgacctgtcttGT-CTCGCCT

CACTCCTCCCGCCCTGTTCTCTTGCAGGCACAGA

>rhesusmonkey

CACAGGCAACTCCCTGAATGAACCAGAAGAGCCCTCTGATATCCCCGAAA

GGAGGGATTCTGGGGTTGGGGCTTCCCTAACCAGGTCCAACAGGTAACAA

TTTTTC-TCCTCCCTCCAGGTGTGAGGCA-CGGGGG-TCA-GAAAGAGAT

TGCCTGTGCTTGTTCTGTGGGAGGAAATGGGATTAAGTCTGGCATTCTTA

GCCATGTCCATT---------CCTAAGGAAGGTGTCA--GACCAAGAAGC

AC---CAA-AATTCAAA---------------------------------

---AAATGGATATGA---TTGaatgggtatggagttctggttggggaaga

tgaaaaaagt----tctgga-gatggttggtagagatggt--caca-caa

tgagaatg----aacttggtgccgctcaactgcacacttaaaaatggtga

aaacgatacactttatatt-gtgtatattttcccataat-----------

----------GGGGATTA---AAG--CAT-----GGAATAGAAA--TCAA

GTCCTCTCAGTT------------CCTAAGACAAGATG-CCAGCAAGCAG

CCCATTGCTAAT-GTT--GCTTCTACCTATTAAAAAGCCTTTG--ggcca

ggcacagtggctcactgtaat---ccaatactttgggaggctgaggtggg

aggatcacttgaggccaagagtttgagactaggctggacaacataggtag

actccgtctctacaaaaaatttaaaaatgagccagacatggtggcacagg

cctgtagtcctggctactcaggaggctgaggccagaggatcactcgagcc

caggagttcaaggctgcagtgagctctgatggcaccactgcctgggtaac

agagcaagaaaaatcaaataaataaaatcaaataaaaa------------

--------------------------------------------------

--------------------------------------------------

--------------------------------------------------

----------------------------------------GCCTTCACAC

TCACTTTTCTCTCCATTTCTCTCGCAGGCACCGA

>human

CACAGGCAACTCCCTGAATGAACCGGAAGAGCCCTCCGAGATCCCGGAAA

GAAGGGATTCTGGGGTTGGGGCTTCCCTAACCAGGTCCAACAGGTAAGAA

CTTTTC-TCCTGCCTCCGGGTGTGGGGCA-CTGGGGGTCA-GAAAGACAT

TGCCTGTGCTTGTTCTGTGGGAAGAAATGGGATTAAGTCTGGCATTCTTA

GCCATGTCCATT---------CCTAAGGAAGGTGTCA--AACCAGGAAGC

AC---CAA-AATTCAAAG--------------------------------

---AAATCGATAGGA---ttgaatgggtatagagttccggttggggaaga

tgaaaaaagt----tctgga-gatggttggcagagatggt--caca-caa

tgagaatg----aacttggtgccactcaactgcacatttaaaaatggtga

aaatggtgcactttatatt-gtgtatattttcccataat-----------

----------GGGGATTA---AAG--CAT-----GGGATAGAAA--TCAA

GTCCTATCAGTT------------CCTAAGATAAGATG-CCAGCAAGCAG

CCCATTGCTAAT-GTT--GCTTCTACCTATTAAAAAGCCTTTG--ggcca

ggcacagtggctcacacttgtaatccaatactttgggaggccgaggtggg

aggatcacttgaggccaagagtttgagaccaagctggacaacatagggag

actctatctctacaaaaaattaaaaaatgagtcagatgtggtggcacagg

cctacagtcctggctactcaggaggctgaggcaggaggatcacttgagct

gaggagttcaaggctgcagtgagctgtgatggcaccactgcactccagcc

tgggcaacagagcaagaaaaaataaaataaataaaatcaaataaaaa---

--------------------------------------------------

--------------------------------------------------

--------------------------------------------------

----------------------------------------GGCTTCACAC

TCATTTTCCTCTCCTCTTCTCTCGCAGGCACCGA

>chimpanzee

CACAGGCAACTCCCTGAATGAACCGGAAGAGCCCTCCGACATCCCGGAAA

GAAGGGATTCTGGGGTTGGGGCTTCCCTAACCAGGTCCAACAGGTAAGAA

CTTTTC-TCCTGCCTCCGGGTGTGGGGCA-CCGGGGGTCA-GAAAGACAT

TGCCTGTGCTTGTTCTGTGGGAGGAAATGGGATTAAGTCTGGCATTCTTA

GCCATGTCCATT---------CCTAAGGAAGGTGTCA--AACCAGGAAGC

AC---CAA-AATCCAAAG--------------------------------

---AAATCGATAGGA---ttgaatgggtatagagttccggttggggaaga

tgaaaaaagt----tctgga-gatggttggcagagatggt--caca-caa

tgagaatg----aacttggtgccactcaactgcacatttaaaaatggtga

aaatggtgcactttatatt-gtgtatattttcccataat-----------

----------GGGGATTA---AAG--CAT-----GGGATAGAAA--TCAA

GTCCTATCAGTT------------CCTAAGATAAGATA-CCAGCAAGCAG

CCCATTGCTAAT-GTT--GCTTCTACCTATTAAAAAGCCCTTG--ggcca

ggcacagtggctcacacttgtaatccaatactttgggaggccgaggtggg

aggatcacttgaggccaagagtttgagaccaagctggacaacatagggag

actccatctctacaaaaaattaaagaatgagtcagatgtgatggcacagg

cctacagtcctggctactcaggaggctgaggcaggaggatcacctgagcc

gaggagttcaaggctgcagtgagctgtgatggcaccactgcactccagcc

tgggcaacagagcaagaaaaaataaaataaataaaatcaaataaaaa---

--------------------------------------------------

--------------------------------------------------

--------------------------------------------------

----------------------------------------GGCTTCACAC

TCACTTTCCTCTCCTCTTCTCTTGCAGGCACCGA

>seacow

CACAGGCAACTCCCTGAATGAACCCGAGGAGCCCTCCGACATCCCCGAAA

GGAGAGACTCTGGGGTTGGGGCTTCCCTGACAAGGTCCAACAGGTAAGAG

CTTTTCCTC-TCCCTGCAGAT------CTAGT-GGAGGCAGGAAGGG-AC

TCCTTCTGTTTGTTCCGTGGGAGTAAATGGGATTAAGCATGGCACTTATA

GCTGTTTAAGTT---------CCTAAAGAAGGTGTCA--GGCCAGGACAT

CC---TAAC-ACTCAGAAA-------------------------------

--------------------------------------------------

--------------------------------------------------

--------------------------------------------------

--------------------------------------------------

------------GGATGG---AATGGAAG-----------------TTGA

GTCCTCCTG-------------TTGGCAGAGTAAGGTG-TCAGCGAGCAC

CCCATT-GCTAA--CCT-GTGTCTGCTGGTTAGGAAG-CTTTG-------

--------------------------------------------------

--------------------------------------------------

--------------------------------------------------

--------------------------------------------------

--------------------------------------------------

--------------------------------------------------

--------------------------------------------------

--------------------------------------------------

--------------------------------------------------

-------------------------------------------TACTCAC

GCGCTGTCCCCTGCCCTTCTCCCACAGGCACAGA

>elephant

CACAGGCAACTCCCTGAACGAGCCTGAGGAGCCCTCCGACATCCCCGAAA

GGAGAGACTCTGGGGTCGGGGCTTCCCTGACAAGGTCCAACAGGTAAGAG

CTTTTCCTC-TCCCTCCAGAT-TGGGAAGG---GGAGCCA-GAAGGGGAC

AGCTTCCACTTGGTCTATAGGAGTAAATGGGATTAAGCATGGCCTTTATA

ACTGTTTAAGTT---------CCTAAGGAAGGTGTCA--GGCCAGGAAAT

CC---TAAC-ACTCAAAAA-------------------------------

--------------------------------------------------

--------------------------------------------------

--------------------------------------------------

--------------------------------------------------

------------GGACGG---AATGGAAG-----------------CTGA

GCCCTCCCAT-------------TGGTAGAGTAAGATG-TCAGCGAGCTC

CCCGTT-GCTAG-TTTT-GCGTCTGCTGCTTAAGAAGCCTTTG-------

--------------------------------------------------

--------------------------------------------------

--------------------------------------------------

--------------------------------------------------

--------------------------------------------------

--------------------------------------------------

--------------------------------------------------

--------------------------------------------------

--------------------------------------------------

-------------------------------------------TACTCAC

GTGCGTTCCCCTGCGCTTCTCTCACAGGCACAGA

>sloth

CACAGGCACCTCACTGAATGAGCCTGAGGAGCCCTCAGACATCCCGGAAA

GAAGGGATTCTGGGGTTGGGGCTTCCCTGACAAGGTCCAATAGGTAAGAG

CTTTTCCTTCTGTGGCCAGATTGGGGAAGGGG-GGA-TCA-GAAAGGGGC

TGC---------TTCAGTAGGAGTAAATAAAATTAAGTGTGGCATTCATA

GCCATCCAAGTT---------CCTCAGGAAAGGGCCA--GCTCAGAAATT

CC---TAAC-ACTCAGAAAG------------------------------

--------------------------------------------------

--------------------------------------------------

--------------------------------------------------

--------------------------------------------------

---------------------AAAGAGT----------------------

--CCTCTCAGA------------CCTAAAATTAAGGTG-TCAGCCAGCAG

CTCATT-GCTGA-TTTC-GCATCTACCTATTAAAAAGCCTTTG-------

--------------------------------------------------

--------------------------------------------------

--------------------------------------------------

--------------------------------------------------

--------------------------------------------------

--------------------------------------------------

--------------------------------------------------

--------------------------------------------------

--------------------------------------------------

-------------------------------------------CACTCAC

TTGTGTTCCCTCACTCTTCTCTCCCAGGCCCGGG

Marker 3f:

>L1MB3

--------------------------------------------------

--------------------------------------------------

-------------------------------------------------t

tt------ttttaattgt----ggtaaaatata--cataacataaaattt

accattttaaccat-------------------tttta-agtgtacagtt

cagtggcattaagtacattcacattg--ttgtgcaac-catcaccacc--

-----------------------------at-------------------

--------------------------------------------------

--------------------------------------------------

--------------------------------------------------

--------------------------------------------------

--------------------------------------------------

--------------------------------------------------

--------------------------------------------------

--------------------------------------------------

--------------------------------------------------

--------------------------------------------------

--------------------------------------------------

------------

>cow

CAGCTCGGGATGTATTTTTAATGCTAAAGAAGCCAAACTATAAGAAGTTG

GAACTTCAAGTGTATGCAACCTTTTTTGAAATTTATAGTGGTAAG---GT

AAGTT---GAAATTTTAAAATAATTT-----------------------t

tgtaagctttttaaattgtggtttaaaaatac-------atataaaattt

accattttaactat-------------------tttttagtgtagaagtt

caccagaattgagtgcattcacattg--ttggacagttgttgccaccac-

-----------------------------ATAATTGCT----ATATGTAT

TTAGAATATTAC----------TTT-AAAGTTTTGTGTATAAGATTTTTT

TTTAATT-CCCTTT--GAAATTCTTTAA-CTTCAATATCAATCATTACTA

TTCTAAT-AAC-----AATTATTACTAGGATTCTCTGAAAA---------

-AATTTTTTAGACTCTGAATGACTCATTT---------------------

--------------------------------------------------

--------------------------------------------------

--------------------------------------------------

--------------------------------------------------

--------------------------------------------------

--------------------------------TTATAGAACTGTTCTTAT

TTTCAAAAGCTTTTTTTTTTTTTTTTTTTAGGTGTTTGACTTGCTAAATA

GGAAAACAAAAT

>dog

CAGCTCGAGATGTCTTTTTAATGTTAAAGAAGCCAAATTATAAGAAGTTA

GAACTTCAAGTATATGCAACCTTTTTTGAAATTTATAGTGGGAAG---GT

AAGTG---GAAACTTAAAAAAAAAACCCTGATTGGGGGGGGCGGTG----

---------tttaaattgtggtttaatatgtg---cataacataaaattt

aacactttaaccaa-------------------ttgtt-gtgtacaagtt

cagtcgcattaactactttcatgttg--taaaa---ctattgccagcat-

-----------------------------GTATTTTT-----ATTCACA-

-----------TTTTTTG----TTAG-CATT----TTGTTGAGATTTTT-

AAAAATT-CCCTTT--GAAACTCTCAA--CTTGAGTTTTGAGCATTA---

TTCTAATTATTCT---AATTTTCTGAAATAATGTTTGGATTCTGATT---

-CATTTTTACAGAACTGAAGTTTTTTTTT---------------------

--------------------------------------------------

--------------------------------------------------

--------------------------------------------------

--------------------------------------------------

--------------------------------------------------

-------------------------------TCCCCTTAAAGCTAGTTTT

CTAATTTGGCTTTTTTT-------CAAACAGGTGTTTGACTTACTAAACA

GGAAAACAAAAT

>bat4b

TAGCTCGAGATGTCTTTTTAATGCTAAAGAAGCCAAACTATAAGAAGTTA

GAACTTCAAGTTTATGCAACCTTTTTTGAAATTTATAGTGGAAAG---GT

AAGTG---GAAACTTAAAAAAAAAAATTTGTTT-----------------

---------tttaaattgtgattaaatta-tg---cataacataaaattt

attattttaaccat-------------------ttttaagtatacaaatt

caatggcat--gtacattttaca-----ttgtgcaaaaatggcatgtaca

ttttacaatgtgcaaccattgccaccacATCACTTTT-----ATATGTAT

TTGGATTGGAATATTATG----TTAGAATTTTGTTGAGATTTCTTTT---

---AATT-CCCTTT--GAAACTTTTCAA-CTCAAATATTGAGCATTTTAT

TTCTGAAATGGTTTCAGACTTGAATGACTGAATCCACCACATGATGGTC-

--------------------------------------------------

--------------------------------------------------

--------------------------------------------------

--------------------------------------------------

--------------------------------------------------

--------------------------------------------------

--------------------------------------------------

--------------------------------------------------

------------

>guineapig

CAGCTCGAGATGTCTTTTTAATGTTAAAGAAGCCAAACTATAAAAAGTTA

GAACTGCAAGTGTATGCAACCTTTTTTGAAATTTATAGTGGAAAG---GT

ATGTG---GAAACTTCAC--------------------------------

-------ttttgttttttaatcat--tataaaatacatataaaccatttt

t-aaatgtacaactcagtgatatttgaatatgtttgctcttttacagctt

taccattataaactatatgtatttggactgcaa---ctt-ag----aat-

-----------------------------TTATGTGA-----ATGTCACT

TAAAGCT----CTTAGTG----TTAAACATT-ATTGTTCTAATAATTAT-

---TTTC-ATCTCT--GAAATAACTTCCAGATTCATTTTGATAAACTAAT

TCTTCTTTTTTTAAAACAGCTCATTTT------------CTGTTTGAATA

TGATTTTTCATAGG-TGTTTGACTTGCTA---------------------

--------------------------------------------------

--------------------------------------------------

--------------------------------------------------

--------------------------------------------------

--------------------------------------------------

--------------------------------------------------

----------------------------------------------AATA

GGAAAACAAAAT

>rhesusmonkey

CAGCTCGAGATGTCTTTTTAATGCTAAAGAAGCCAAACTATAAGAAGCTA

GAACTTCAAGTATATGCAACCTTCTTTGAAATTTATAGTGGAAAG---GT

AAGTG---GGAACTTT---------------------------------t

ttaa----ttttaattt----ttttaaatatatatatatatatatatata

tatatatatatatatatataacataacatttgccattttaaccattttta

agttcagtggcattaaatatattcacattgttgtgcagccttgaccacca

------------------------------tAATTT------ATGTGTAT

TT-AGAA----CTTTTATG---TTAGAATTTAT--GAG-----TTTTTTT

TAAAATG-TCCTTT--GAAACTTTTCAA-CTTCAATGTTGAGAATTCTTA

TTCTAATTATTATTTTAATCTCT-GAAATAATTCTTAAACTCTGGGTGAC

TTATTTTTATAGAACTGATATTTTAATTG-CCacaggatctcgctctgtc

acccatgctggagtgcagtggcatgatcacagctcactgtagcttcaatc

tcgtgagc----------ttcctgcctcagctttcctagtagctgcgact

ataggcatgcaccaccatgcccagctaattttttttgtttttagcagaga

cagcgtctcactatgttgcccaggctggttttgaactcctgagctcaagc

tatcctcctgcctcagcctcccaaaatgctgggattgcaggcgtgagcca

ctgtgcctagccTC--TTTTTTACTTTAAAG-----CTAGTTTTCTAATT

TGAATATGGTTTTT------------CATAGGTGTTTGACTTGCTAAATA

GGAAAACAAAAT

>human

CAGCTCGAGATGTCTTTTTAATGCTAAAGAAGCCAAACTATAAGAAGCTA

GAACTTCAAGTATATGCAACCTTCTTTGAAATTTATAGTGGAAAG---GT

AAGTG---GGAACTTT---------------------------------t

ttaa----ttttaattt-----tttaaaatatatatataacataacattt

gccattttaaccat-------------------tttta-agt-------t

cagtggcattaaatatattcacattg--ttgtgcagc-gtttaccacc--

-----------------------------ATAATTT------ATGTGTAT

TT-AGAA----CTTTTATA---TTAGAATTTAT-G-TTATGAGATTTTTT

TAAAAAG-TCCTTT--GAAACTTTTCAA-CTTCAGTGTTGAGAATTCTTA

TTCCAATTATTATTTTAATCTCT-GAAATAATTCTTAAACTCTGGGTGAC

TCATTTTTATAGAACTGATATTTTAATTG-ccacaggatctcgctgtgtc

acccatgctggagtgcagtggcatgatcacagctaactgtagcttcaatc

tcctgagctcaagtgagcttcccgcctcagctttcctagtagctgcgact

ataggcatgcaccaccatgcccagctaatttgttttatttttagcagaga

caaggtctcactatgttgcccaggctggttttgaactcctgagctcaagc

catcctcctgcctcagcctcccaaaatgctgggattacaggcgtgagcca

ttgcacctagCCTTTGTTTTTTACTTTAAAGAAGCTCTAGTTTTCTAATT

TGAATATGGTTTTT------------CATAGGTGTTTGACTTGCTAAACA

GGAAAACAAAAT

>chimpanzee

CAGCTCGAGATGTCTTTTTAATGCTAAAGAAGCCAAACTATAAGAAGCTA

GAACTTCAAGTATATGCAACCTTCTTTGAAATTTATAGTGGAAAG---GT

AAGTG---GGAACTTT---------------------------------t

ttaa----ttttaatt-t----tttaaaatatatatataacataacattt

gccattttaaccat-------------------tttta-agtt-------

cagtggcattaaatacattcacattg--ttgtgcagc-gtttaccacc--

-----------------------------ATAATTT------ATGTGTAT

TT-AGAA----CTTTTATA---TTA-GAATTTATG-TTATGAGATTTTTT

TAAAAAG-TCCTTT--GAAACTTTTCAA-CTTCAGTGTTGAGAATTCTTA

TTCTAATTATTATTTTAATCTCT-GAAATAATTCTTAAACTCTGGGTGAC

TCATTTTTATAGAACTGATATTTTAATTG-ccacaggatctcgctgtgtc

acccatgctggagtgcagtggcatgatcacagctaactgtagcttcaatc

tcctgagctcaagtgagcttcccgcctcagctttcctagtagctgcgaat

ataggcatgcaccaccatgcccagctaatttgttttatttttagcagaga

caaggtctcactatgt-gcccaggctggttttgaactcctgagctcaagc

catcctcctgcctcagcctcccaaaatgctgggattacaggcgtgagcca

ttgcacctagCCT--GTTTTTTACTTTA-----AAGCTAATTTTCTAATT

TGAGTATGGTTTTT------------CATAGGTGTTTGACTTGCTAAACA

GGAAAACAAAAT

>tenrec

CAGCTCGAGATGTCTTTTTAATGCTTAAGAAGCCAAACTATAAGAAGTTA

GAACTTCAAGTATATGCGACCTTTTTTGAAATTTACAGTGGTAAG---GT

AAGTG---------------------------------------------

--------------------------------------------------

--------------------------------------------------

--------------------------------------------------

-----------------------------CCCATTTTTC---ATA--TAT

TCTGGAATA-------------TTG-TTAAAATTCGTTAGGAA-ATTTTT

TTAG-TA-CCCTTT--GAAAGCACTTCAGCTTTACTGTTGAGCA-TTATT

AGTACTCTAAAAA------------TGCTTTGAAATTTTCTAACTTTGGT

TCATTTT-GGAAAACATCATTTTTTTAAA---------------------

--------------------------------------------------

--------------------------------------------------

--------------------------------------------------

--------------------------------------------------

--------------------------------------------------

---------------------------------AAACAACTTGTAATTTT

AAAATGCCTTTTTGT--------------AGGTGTTTGACTTGCTAAACA

GAAAAACAAAAT

>elephant

CAGCACGAGATGTCTTTTTAATGCTAAAGAAGCCAAACTATAAGAAATTA

GAACTTCAAGTATATGCAACCTTTTTTGAAATTTATAGTGGAAAA---GT

AAGTA---------------------------------------------

--------------------------------------------------

--------------------------------------------------

--------------------------------------------------

-----------------------------AA--TTTTTT---ATGTGTAC

T--GGAATATT-----------TTA-TTAAAATTTGTTAGGAGA-TTTTT

TAAAATG-TGCTTT--GGAACTTTTCAG-CTTTAATGTTGAGCA-TTACT

ATAAATTTAAAAA-----------------------TTTCAGCCTTTGAC

TCATTTTTT-AGAACTGATTCTTTCTTTT---------------------

--------------------------------------------------

--------------------------------------------------

--------------------------------------------------

--------------------------------------------------

--------------------------------------------------

----------------------------CTGAAAAACTAGTTAGCTAATT

TAA-CATGGTTTTTTG----------C--AGGTGTTTGACTTGCTGAACA

GGAAAACAAAAT

>armadillo

CAGCTCGAGATGTCTTTTTGATGCTAAAAAAGCCAAACTATAAGAAGTTA

GAACTTCAAGTATATGCAACCTTTTTTGAAATTTACAGTGGAAAG---GT

AAGTG---------------------------------------------

--------------------------------------------------

--------------------------------------------------

--------------------------------------------------

-----------------------------TAGATTTTTT---ATATATAG

T-GGAAATT------------TTATGTCAGAATTTATGCTGTGAAATTTT

TTTAAATGCCCTTT--GAAACTCTTCAC-CTTCAGTGTTGAACA-TTATT

ATTTTAATAATAC------------TCAGAATTTTCTGACTCTGAATGAC

TCATTTTTACAGAACTGACTCTTTTTCTC---------------------

--------------------------------------------------

--------------------------------------------------

--------------------------------------------------

--------------------------------------------------

--------------------------------------------------

-----------------------------TTTAAAACTAGTTTTCTAATT

TGAATATGATGTTT------------CATAGGTGTTTGACTTGCTAAACA

GGAAAACAAAAT

>opossum

CAGCTCGAGATGTCTTTTTAATGCTAAAGAAACCAAACTATAAGAAGCTA

GAGCTTCAAGTATATGCAACATTTTTTGAAATTTATAGTGGAAAG---GT

AAGTA---------------------------------------------

--------------------------------------------------

--------------------------------------------------

--------------------------------------------------

-----------------------------TAGGCTTAGT---ATTTAAAA

TACTAAAAGTTCTTTTGTTAATTTCATTTGAATACAAATTTAATTATTTT

TAAAATTTGGCTTTTTGAAACTGTTTAAATTCATCACATAATCA-TTGTT

TAAAATCTACTT-----------------------AACCAGCTCTCCTCT

GTTCTTT-GAGTCCCTCAGTTCTATTAGC---------------------

--------------------------------------------------

--------------------------------------------------

--------------------------------------------------

--------------------------------------------------

--------------------------------------------------

---------------------------------------ATTTCATTATT

TAACATAGATGTTATTT--TTTTTCCCAAAGGTATTTGACTTGCTTAATA

GGAAGACAAAGT

Marker 3g:

>L1MB3

--------------------------------------------------

--------------------------------------------------

--------------------------------------------------

--------------------------------------------------

--------------------------------------------------

--------------------------------------------------

--------------------------------------------------

--------------------------------------------------

--------------------------------------------------

--------------------------------------------------

--------------------------------------------------

--------------------------------------------------

--------------------------agatatt-tgtacacccatgttca

tagcagcattattcacaatagcc---------------------------

--------------------------------------------------

--------------------------------------------------

--------------------------------------------------

-------------------------------------------------a

aaaggtggaagcaacccaagtgtccatcgacgg-atgaatggataaacaa

aat-----------------------------------------------

--------------------------------------------------

--------------------------------------------------

--------------------------------------------------

--------------------------------------------------

--------------------------------------------------

---------------------gtggtatatacatacaatg-gaatattat

tcagccttaaaaaggaaggaaattctgacacatgctacaacatggatgaa

ccttgaggacattatgctaagtgaaataagccagtcacaaaaggacaaat

actg--tatgattccacttatatgaggtacctagagtagtcaaattcata

gagacagaaagtagaatggtggttgcc-aggggctggggggaggggggaa

tggggagttaktgtttaatgggtacagagtttcagttt---gggaagatg

aaaaagttctggagatggatggtggtgatggttgcacaacaatg-tgaat

gtacttaatgccactgaactgtacacttaaaaatg-----gttaaaatgg

-taaattt-tatgttatgtatattt-------------------------

--------------------------------------------------

--------------------------------------------------

--------------------------------------------------

----------------------taccacaattaaaaaaa-----------

--------------------------------------------------

--------------------------------------------------

--------------------------------------------------

-------------------

>dog

ACTTACTCCTCCAGGACTTTCATTTTCTCTCCTTTCTTGAAAGACAAGTC

ATCTGGATGGATGCCATCATAGGGGTACAAGGCTACCACGATGTCTCCTT

GCTCCTCTGGATCTAAAGATGAA-GGAGGAAAGGGAAAATTTTCAAGAAT

CCAT-CAATGGTAACATTTTAACAA-------------------------

--------------GTCTTTATCAGGCACATAATGAATAATGAGTGTGTT

TCC-------------TGCCTAGTGGGGGCTCAGA---------------

--------------------------------------------------

--------------------------------------------------

--------------------------------------------------

--------------------------------------------------

GCCCACATGGTCTGCAGTAAGTAACATATTACACACACTGGATACAGTAT

ATGTTATCTCCAATCATACAGACCCTACCACTATACTTAA----TTCCAG

CTCTATATC-TAAGAAAT----------------gatacagccatgttca

gagcaggattatttgcaatagct---aaaaagagataacacgggtacctg

ggtggctcagttggttaagcgtctgcctttggctccggtcatgatctcag

ggtcctgggacggagccccatgttgggctccatgttcggggggagtctgc

ttctccctctgtctctgctcttccctccccacacaggtcatgctcactca

ctctctctctcttcctcaattaaatagaatctttaaaaataataataata

aaaggaggaaacagtccaaatgttcagtgacaa-atgaatggctaaacaa

aatgtggcttgtaca-----------------------------------

--------------------------------------------------

--------------------------------------------------

--------------------------------------------------

--------------------------------------------------

--------------------------------------------------

----------------------caga--------tca----gaatactat

tcagctttaaaatggaaggacattctgacaccagctacaacatggatgaa

ccctgcagacattacacgaagtgaaataagccagacatgaaagggcaaaa

acacgctaagattccatttatttgcggtccctagagtagtcaaaatcatt

gagatggaaagtataatagtggctgccaagggctgaagggagtaggggag

cagggagttagtgcttaattcggacagagttccagtttggggagatgatg

aag---ctccagagatggatggtggtgctggctgcccaatgatg-tgagt

gtacttaatgctgctcaactggacactaaaaaatg-----gttacaatga

-tatattt-tatactacatgtattt---tttttcaagatttaatttattt

attcacgagaaacacagagagaggggcagagacacaggcagagggagaag

cagtttctctgcagggagcccaatgcaggacttgatcccaggaccccggg

atcacgacctgagccaaaggcagacgcccaaccactgagccacccaggtg

acccCATGTATT----------TTTCTACAATGAAAATATAAACAA----

--GAATACCCAAGCTGACTACCTT-ATAGCAACTCTAACTGTAAAGAAAG

GGGGAAAAAATCCAAAAAACCCCACTGTGTTGCCATGTAAACATACCTTT

AGTTTGAAACCTCTGTCCTGG-TAAAAGCTGAGATTCTGAAACCTAGGAA

AGAAGATTTGTTAGGAAAA

>mouse

ACTTACTCTTCTAGAACTTTCATCTTTTCTCCTTTCTTGAAGGACAAGTC

ATCTGGGTGGATGCCATCATAAGGGTATAAGGCCACCACAATGTCACCTT

GTTCCTCTGGATCTAAGAACAAAAGATACAAGAAGAG-CTCTTCAA-AAC

CTAT-CAACATAAAC-----ACAAA-------------------------

AGAAGAGAGGAAGCAATGGAGAGAGGGCCA-TGGAGTGAAGCTCTGTGCT

TCCCACCT------------------------------------------

--------------------------------------------------

--------------------------------------------------

--------------------------------------------------

--------------------------------------------------

--------------------------------GTAAATGACATGTGGTGC

ATCCCATGTATA-----CCAATACCCACAGCAATCTACTA----TAAC--

-TTCAAAACCATGC-TTAG-------gaaaggacgtacacacgatcatgg

caacactgtt-------atggtctc-------------------------

--------------------------------------------------

--------------------------------------------------

--------------------------------------------------

-------------------------------------------------c

ataggcagaaaccactctagtatccattagtggaatggacaaaatatgac

atatacacagaata------------------------------------

--------------------------------------------------

--------------------------------------------------

--------------------------------------------------

--------------------------------------------------

--------------------------------------------------

-----------------------gaatatacatat----gcacagtctag

tcagccttaaaaagcattaaagttctgacacacaatggactatggatgag

ctttgaaaactt-----tcagttaaatgagccaggcacaaaatgacaaag

accatat----ttccagtcgcatgtgttacatagaatc---aaatccata

aggacagagggtgtaatggtggctgccc-agagcagaagggagaggagag

caatgagtgagtgtttaat---gacagcattccactt----ggaataatg

aaaa-cctcctgatatgaacactggtgatgcttgcacagcaatg-----t

gtacttcctgtt-cagaacttgaca-ttgaaaacagtgactgaaactgtg

ataaactt-gtgt-tgtgtgta----------------------------

--------------------------------------------------

--------------------------------------------------

--------------------------------------------------

--------------------TTTTTCCGCAATGACAAACAA---------

--------------------------------------------------

----------------AACATGAATTATGTTGGTTAGGAAACATACCTTT

TGTTTGAAATCTCTGTCCTGG-TAAAAGATGAAATTCAGGAACCTATGGA

AGAGTATTTGTTAGAGGAA

>rat

ACTTACTCTTCCAGAACTTTCATCTTTTCTCCTTTCTTGAAAGACAAGTC

GTCTGGGTGGATGCCATCATAGGGATACAAGGCCACCACAATGTCCCCTT

GCTCCTCTGGATCTAAGAACAAAAGATACAAGCAGAG-CCCTTCAA-AAC

CTAG-CAACATAGAC-----ACAAA-------------------------

GGAAGAGAGGAAGCAACGGAGAGAGGGCCG-TGGAGTGAAGCTCCGTGCT

TCCCACCT------------------------------------------

--------------------------------------------------

--------------------------------------------------

--------------------------------------------------

--------------------------------------------------

--------------------------------GTAAATGGCATGAGGTAC

ATCCTGTGCA-----CACCAAGACCCACAGGAACCCACTA----TACC--

-TTTAAAACCATGT-TTAG-------gaaaaaatgtacgcgtaatcgtgg

caacactgcc-------atagtccc-------------------------

--------------------------------------------------

--------------------------------------------------

--------------------------------------------------

-------------------------------------------------c

aaaggcagaagctaccctagtgagcactagtggaatgcataaagtgtgac

acatacacagaata------------------------------------

--------------------------------------------------

--------------------------------------------------

--------------------------------------------------

--------------------------------------------------

--------------------------------------------------

-----------------------ggacatccacat----acacagagcag

tcagccttaaaaagcattgaagttcttacacacaatggagtgtagatgag

ccttgacgac-------caagtcaaacgagccaggcaggaaatgacaaag

actatat----ttccagttgcttgtgctacatagaatc---aagtccata

gggacagagagtgcaatggtggctgccc-agagcagaagggagtggagaa

caatgagtgagtgcttaat---gacagtgttccactc----ggaataatg

aaaaccttctgtgacgga-cactggtgacacttgcacagcaatgcgtact

tcctgttctgagctcgacattaagaaaaaaaaaaaaccactgaagctgtg

ataaattt-gagt-tacatata----------------------------

--------------------------------------------------

--------------------------------------------------

--------------------------------------------------

---------------------TTTTCCCATAACGACAAACAA--------

--------------------------------------------------

----------------AACATGAATAAGGTTGAAAGGAAAACATACCTTT

TGCTTGAAATCTCTGTCCTGG-TAAAAGTTGAGATTCAGGAACCTACGGA

AGAGTATTTGTTAGAGGAA

>rhesusmonkey

ACTTACTCCTCCAGGACTTTCATCTTCTCTCCTTTCTTGAAAGACAAGTC

GTCCGGGTGGATGCCATCATACGGGTACAAGGCTACCACAATGTCTCCTT

GTTCCTCTGGATCTAAAGATGAA----CACAAGAGAACATCTGTAGGAAT

CCATCAGCAGAAGCATTTTAACAAA--------TCTCATGGGGCACAAAG

GATGAGAGGAAG-CACTAAGAGAGGGGCGACTGGAAGAAGGACATGTGCT

TCC-------------TGCCTAGTGGGCTCAGG-----------------

--------------------------------------------------

--------------------------------------------------

--------------------------------------------------

--------------------------------------------------

--GTCTGCAC-GCCCACAGAGAGTAGCTTGCTGTATACACTGTGT-CCAG

TATTCATTATTG-----CAAATCACTTAAAACTAAAACTA----TAA---

-TAT-AACTCTATATTTAA-------gaaat---gatacacccatgttca

cagcaggattatttcaaatagcc---------------------------

--------------------------------------------------

--------------------------------------------------

--------------------------------------------------

-------------------------------------------------a

aaatgtggaaataatgcaaacgtccgttgatgg-aggaatggataaacat

catggggca------ttttttttttttttttttttggagatggactctca

gtcttgtcacccaagctggagtgcaatggtgcaatctcagttcactgcaa

cctccacctcctgggttcaagcgattctcctgcctcagcctcctcagtag

ctgggattacaggtgcccactaccacacccggctaattttttgtgttttt

agtagagatggggtttcaccgtgttgaccaggctggtctcaaactcctga

ccgcaggtgatccacctgccttggactcccaaagtgctgggattacaggt

gtgagccacggcacctggcc--agggcatatacttacaaggaaatatt--

-caccctt-agaaggaaataaactctaacactttctacagcatggatgaa

ccttgaggacatcacactaagtgaaataatccaaacacagaaggtcaatt

actg--tatgattccacttctaggaggtacctagagtagtgaaattcata

aagacacaaag---agcggagtttgcc-agcggctggggg-aggaggcaa

tggggacttattgtttaatgg-------gtatgcagtttcagtttgagaa

gatgcgttctggagatggagggtggtactggttgtacaacaatg-tgaat

gtacaaaatgccgcagaaatgtatacttaaaaatagt-----tatgaggg

-taaatttttatgttatacgtg----------------------------

--------------------------------------------------

--------------------------------------------------

--------------------------------------------------

------------------------TCCCACAATAAAAAAA----------

------TAAAA-CATTTAGGACAATCACTTTATAGAAGCTCTAATAGTAA

AGAA--------AAAAAAATTTAACTATGTTGCTATGAAACCATACCTTT

AGCTTGAAACCTCTGTCCAGG-TAAAAGCTGAGATTCTGGAACCTATGAG

AGAATATTTGTTAGAAAAA

>human

ACTTACTCCTCCAGGACTTTCATCTTCTCTCCTTTCTTGAAAGACAAGTC

GTCCGGGTGGATGCCATCATAGGGGTACAAGGCTACCACAATGTCTCCTT

GTTCCTCTGGATCTAAAGATGAA----CACAAGAGAACACCTGTAAGAAT

CCATCAGCAGAAGCGTTTTAACAAA--------CCTCATGGGGCACAAAG

GATGAGAGGAAG-CACTAAGAGGGGG-CAACTGGAAGAAGGACATGTGCT

TCC-------------TGCCTAGTGGGCTCAGG-----------------

--------------------------------------------------

--------------------------------------------------

--------------------------------------------------

--------------------------------------------------

--GTCTGCACGGCCCACAGAGAGTACCTTGCTGTATACACTGTGT-CCAG

TATTTGTTCTTG-----CAAATCACTTAAAACTAAAACTA----TAAA--

-TAC-AACTCTATATTTAA-------gaaat---tatacacccatgttca

cagcaggattattcaaaatagcc---------------------------

--------------------------------------------------

--------------------------------------------------

--------------------------------------------------

-------------------------------------------------a

agatgtggaaataatgcaaacatctattgatgg-aggaatggataaacat

catgtagca---------ttttggtttttctcttttgagacggagtctcg

ctcttgtcgcccagactggagtgcaatagtgcaatctcggctcactgcaa

cctccacctcctgggttcaagctattctcctgcctcagcctccccagtag

ctgggattacaggcgcccaccaccacgcctggctaattttttgtgttttt

agtagagacggggtttcaccatgttgaccaggctggtctcgaactcctga

ccacaggtgatctacctaacttggcctcccaaagtgctgggattacaggt

gtgagccactgcacccggcgc-agggcatatacttacaaggaaatattat

tcactatt-agaaggaaataaattctaacactttctacagcatggatgaa

ccttgaggacatcaggctaagtgaaataatccaaacacaaaaggacaatt

actg--tatgattccacttctaggaggtacctagagtagtgaaattcata

aagacagaaag---agcggaggttgcc-agcggctggggg-aggaggcaa

tggggacttattgtttaatgg-------gtatggagttt---gagaagat

gca---ttctggagatggagggcggtattggttgtacaacaatg-tgaat

gtacctaatgctgctgaaatgtatacttaaaaatagt-----taagaggg

-taaatttttatgttatatatg----------------------------

--------------------------------------------------

--------------------------------------------------

--------------------------------------------------

------------------------TCCCACAATAAAAAAAAA--------

------TAAAAACATTTAGGACAATCACTTTATAGAAGCTCTAATAGTAA

AGAA--------AAAAAATTTTAACTATGTTGCTATGAAAACATACCTTT

AGTTTGAAACCTCTGTCCAGG-TAAAAGCTGAGATTCTGGAACCTACGAG

AGAATATTTGTTAGAAAAA

>chimpanzee

ACTTACTCCTCCAGGACTTTCATCTTCTCTCCTTTCTTGAAAGACAAGTC

GTCCGGGTGGATGCCATCATAGGGGTACAAGGCTACCACAATGTCTCCTT

GTTCCTCTGGATCTAAAGATGAA----CACAAGAGAACACCTGTAAGAAT

CCATCAGCAGAAGCGTTTTAACAAA--------CCTCATGGGGCACAAAG

GATGAGAGGAAG-CACTAAGAGGGGG-CGACTGGAAGAAGGACATGTGCT

TCC-------------TGCCTAGTGGGCTCAGG-----------------

--------------------------------------------------

--------------------------------------------------

--------------------------------------------------

--------------------------------------------------

--GTCTGCACGGCCCACAGAGAGTACCTTGCTGTATACACTGTGT-CCAG

TACTTGTTATTG-----CAAATCACTTAAATCTAAAACTA----TAAA--

-TAC-AACTCTATATTTAA-------gaaat---tatacacccatgttca

cagcaggattattcaaaatagcc---------------------------

--------------------------------------------------

--------------------------------------------------

--------------------------------------------------

-------------------------------------------------a

agatgtggaaataatgcaaacgtctattgatgg-aggaatggataaacat

catgtagca---------ttttggtttttctcttttgagacggagtctcg

ctctcgtcacccaggctggagtgcaatggtgcaatctcggctcactgcaa

cctccacctcctgggttcaagctactctcctgcctcagcctccccagtag

ctgggattacaggcgcccaccaccacgcctggctaattttttgtgttttt

agtagagacggggtttcaccatgttgaccaggctggtctcgaactcctga

ccacaggtgatctacctaacttggcctcccaaaatgctgggattacaggc

gtgagccactgcacctggcc--ggggcatacacttacaaggaaatattat

tcactatt-agaagtaaataaattctaacactttctacagcatggatgaa

ccttgaggacatcatgctaagtgaaataatccaaatacaaaaggacaatt

actg--tatgattccacttctaggaggtacctagagtagtgaaattcata

aagacagaaag---agcggaggttgcc-agcggctggggg-aggaggcaa

tggggacttattgtttaatgg-------gtatggagttt---gagaagat

gcg---ttctggagatggagggcggtactggttgtacaacaatg-tgaat

gtacctaatgccgctgaaatgtatacttaaaaatagt-----taagaggg

-ttaatttttatgttatatatg----------------------------

--------------------------------------------------

--------------------------------------------------

--------------------------------------------------

------------------------TCCCACAATAAAAAAAAA--------

------TAAAAACATTTAGGACAATCACTTTATAGAAGCTCTTATAGTAC

AGAA--------CAAAAATTTTAACTATGTTGCTATGAAAACATACCTTT

AGTTTGAAACCTCTGTCCAGG-TAAAAGCTGAGATTCTGGAACCTACGAG

AGAATATTTGTTAGAAAAA

>elephant

ACTCACTCCTCTAAGACTTTCATTTTCTCTCCTTTCTTGAAGGACAAGTC

GTCTGGGTGGATGCCATCATAGGGGTACAAGGCCACCACAATGTCTCCTT

GCTCCTCTGGATCT--AAAACAAAAATCACAGC-CAAGACCTTTAAGAAT

CCATCAGCAGTTACACTTCAATGAATCATCATTAGGCCGAAAGAGCAAGA

CAAAGCACTAAG-CAAGG-GAGGGCGGTTA--GAATGATAGCGCAGTTCC

TCCCCCAGGGAGCTGATGCCTACTCACCCACAGAATGCAAGTTACTGCAC

ATGTTGTGCACAACATTTGCTAGCTCAAAACACTAAACAAACAAAAACTC

ACTGCCATCGAGTCAATTCTGACTCATAGCAACCCTGCAGGACAGGGTAG

AAGTGACCCTGGGGGTTTCCAAGACTGCAAATCTCTATGGGAGTAGACAG

CCTCATCTTTCTCCCGTGGAGTGGCTGGTAGTTTTGAACTGCTGACCTGG

TGGTTAGCA--GCCCTAT--------------GTATATGTATGTCACCAG

GACTCCCCT--------CAAAACACAAGAGCCAACAAATA----TAAA--

-TAA----------------------------------------------

--------------------------------------------------

--------------------------------------------------

--------------------------------------------------

--------------------------------------------------

--------------------------------------------------

--------------------------------------------------

--------------------------------------------------

--------------------------------------------------

--------------------------------------------------

--------------------------------------------------

--------------------------------------------------

--------------------------------------------------

--------------------------------------------------

--------------------------------------------------

--------------------------------------------------

--------------------------------------------------

--------------------------------------------------

--------------------------------------------------

--------------------------------------------------

--------------------------------------------------

--------------------------------------------------

--------------------------------------------------

--------------------------------------------------

--------------------------------------------------

-----------------------TTCCAGTTCTATATTTTAAAAA-----

------GGTAAATATCCAGAAGAACTAGTTTATAGAAACTCTACCAAAAG

AAAC------------AAAAAGAAGTATATTGCTATGAAAACATACCTTT

AGTTTGAAACCTTTGTCCTGGGCAAAAGCTGAGATTCTGGAACCTATGAG

AGA-TATTTGTTAGAAAAA

>armadillo

ACTTACTCCTCTAAAACTTTCATTTTCTCTCCTTTCTTGAAAGACAAGTC

ATCGGGATGGATGCCATCATAGGGGTACAGGGCGACCACAATGTCCCCTT

GCTCCTCTGGATCTAGAAAAGAGGAACGCATGC-GCAGACCTTTAAGACT

GCCATTGGCAGTAACGTTAGCCCAAA-----CCCTCGCGGGGCGCGTAGA

TGAAGCCGGGAAAAGAGG-AACAGCTGGAG--GAATG----GCAAGGGTT

TCC-------------TGCCTAGTGGAGTCCATGA---------------

--------------------------------------------------

--------------------------------------------------

--------------------------------------------------

--------------------------------------------------

------------CCCACAGAGA----------GTAAAACTGCAT-ACACT

GCATACACT-----GAGCACATTTGTCCTCTCTAA-ATAAT---TAA---

--------------------------------------------------

--------------------------------------------------

--------------------------------------------------

--------------------------------------------------

--------------------------------------------------

--------------------------------------------------

--------------------------------------------------

--------------------------------------------------

--------------------------------------------------

--------------------------------------------------

--------------------------------------------------

--------------------------------------------------

--------------------------------------------------

--------------------------------------------------

--------------------------------------------------

--------------------------------------------------

--------------------------------------------------

--------------------------------------------------

--------------------------------------------------

--------------------------------------------------

--------------------------------------------------

--------------------------------------------------

--------------------------------------------------

--------------------------------------------------

--------------------------------------------------

-----------------------TTCCAACTTTATATTTTAACAG-----

------TGAAACCATCCAGGACAAGGACTTCACAGAGTTAAGGGGG----

---------------TTAAATGAACTATGTTGCTATGAACACTTACCTT-

--TTTGAAACCTCTGTCCTGG-CAAAAGCTGAGAGTCTGGAACCTACGAG

AGAACAGTTGTTAGTGAAA

Marker 3h:

>L1MB2

--------------------------------------------------

--------------------------------------------------

---tacctagagtagtcaaattcataga------------gacagaaagt

agaatggtggttgccaggggctggggggagggggaaatg--gggagttgt

tgtttaatgggtatagagtttcagttttgcaagatgaaaa--agttctg-

--------------------------------------------------

--------------------------------------------------

--------------------------------------------------

--------------gagatcggttgcacaacaatgtgaatatacttaaca

ctactgaac-tgtacac-ttaaaaatggttaagatggtaaattttatgtt

atgtgtw-ttttaccacaa-ttaaaaaaa---------------------

--------------------------------------------------

--------------------------------------------------

--------------------------------------------------

--------------------------------------------------

--------------------------------------------------

--------------------------------------------------

--------------------------------------------------

--------------------------------------------------

--------------------------------------------------

--------------------------------------------------

--------------------------------------------------

--------------------------------------------------

-----------------------------------------------

>dog

CAGACCAAATACCGAAGAGGT----ATAACTACTATTTTAAATGTGTTTA

AAGCTTCTGGTTGAAAAA--------------------TGTCCTC-----

---ttcctagagtagacaaattcatgga------------gacagatag-

-----ggtggctgccaggagtt--ggggggaggaaagaacaaggagttat

tggttaatctgtatagagtttcagttttgcaagatgaaaag-agttcta-

--------------------------------------------------

--------------------------------------------------

--------------------------------------------------

-----------------gtttgttgcacaataatgtaaatgtacttaaca

ctactgaaa-catac---ttaaaaatggttaagatgatgaataa------

catgtatattttaccacag-tttcaaaaattaaaaaaaaaaa--------

--------------------------------------------------

--------------------------------------------------

--------------------------------------------------

--------------------------------------------------

--------------------------------------------------

----------------------------CTCTCCT---------------

--------------CTTTAACTGATCTGATAC--ATTGTAGATTTTAGCA

TAACATA---TAGTTTGACTAGCTT-GGAAGTCACCATAGTCCTACTTGG

TCCACAATTAATGAGACTAATGATATTTTCATGTATCACGTATCATACAT

AATAAAGCAGCATGCCAATGGTACCTCTGTAGATTCCTCTTGCC------

---------TTTGACCTCCTGTAGGAGCTGCAGGTGAGTTCACACTT---

------CACCTCACATCACTGATATCATGGTATGGCTTTCTGGTCTCAGG

CACTGAACTACTTCCTGAAGCGACAGAAAATTGGAAGATTTGCAACC

>mouse

CAGAGACAATCCCAAAGAGGTACATATATCTAGCAGTTTGAAAGTGTTCC

AACCTTTTGGCTAAAATTAAAAAAAAAAATTTAAA---TGCCCTG-----

---ttccttatccagacagattcatagatctatgaatctagagagaaaag

aaggaggtggctgccaaggcctgagggagggggaagttgc----------

tgcttgcccggtggagattttca-ttttgcgagatgagaagtagtacca-

--------------------------------------------------

--------------------------------------------------

--------------------------------------------------

-------aggagcttgcagctgacagcactaagtg-ttttgtacttctta

cccctgagc-tgtgcac-ttagaa-tggccgagatggtcagtttaatat-

cttatgtctgtgagtacattgcttgtaggagatagagagacgt-cttctg

t-agcccacgtcggctgtgatttgtgtctccccctcagctccccatgctg

gtattacaatcgtgcaccggctgtgaccacagtgggcattcctagtggct

ttctggttttgtttattttgttcttgttttggtttttggcttttcaatgc

gagatttctctgtgtagctcttgctctcctggaatgtactctgtagacca

ggctgacctcagactcacagatccactacctctgccttccaagcactgga

actaaaactgtgcgaccccacagcc----AGCCCT---------------

---------CGAGGCTTTCTTACGTATCACAGTATCCATGGATGTCAGTG

TATCATGAC-TAGTTCGGGTAACAT-GGCTGGAAATTTAAAAGCAACTTC

GGATGA--------------------------------------------

------------TGGCCCTAGTCCCTCCGTAGGTTCCTCTGACC------

---------CCAAGCCCCTTGAGGATGTTACAAG-GG---CACACGTGT-

------GCCAGCTGACATCTCTTGTA-TGGAACTG-TCTCTGGTCACAGG

CACTGAAAACTTTCCTAAACAGACAAAAGACGGGAAGGTTTGCGTCG

>rat

CAGAGATGATCCCAAAGAGGTATGAGCATCTAACAGTTTGAAAGTGTCCA

AACATTTTGTCTAAAATTAAAAAAAAATTAAA------TGCCCTG-----

---ttccttatctagacagattcatagatctatgaatctagagagaaaac

aaaggattggggg--------------aggggaaagttac----------

tgcttgctcaatggagattttcactt--gccagatgaaaagcagtagcc-

--------------------------------------------------

--------------------------------------------------

--------------------------------------------------

-------gggagcttgtggtcagtggcactaagtgtctatggacttatta

cccctgagc-tgtgcac-ttagaa-tggccgagacggtcagtttaatatt

acgc-gtctgtg--------------------------------------

-------------------------gcctcccgcctcagctcccatgctg

atattacaatcacgcactggctttgaccacagtgggca-tcctagtggca

ttctggttttgtttattttgttcttgttttggttttcgacttttcaatac

aagatttctctgtgtagccctggctgtcctggaattcactctgtagatca

ggctggcctc--aaactcagatccactgcctctgcctcccaagcactgga

actgaaactgtgtgaccgcatgtcc----AGCCCC---------------

---------AGAGGCTTTCTTACCTGTCAAAGTATCCATGGATGTCAGTG

TATCATGAT-TAGTTTGGGTAACAT-GGTTGGAAATCTCAAAGCAACTTC

AGCTGA--------------------------------------------

------------TGGCCCTCGTTCCTCTGTAGGTCCCTCCGACT------

---------CCAGGTCCCCTGACAATGGTACAAG-GG---CACACATGT-

------GCCAGCCCACATCTCTTATA-TGGAACTG-TCTCTGGTCATAGG

CACTGAAAGCTTTCCTAAACAGACAGAAGACCGGAAGGTTTGCATCT

>rhesusmonkey

CAGAGGAAATCCTGAAGAGGT----ATATCTAGCATTTAAAATGTG-TTA

AACCTTTGG-ATAATAAA--------------------TACCCTCTTCCA

---tccctatagcagacaaattcataga------------gacagaaagt

gaaatggtagttgccaggggcttggaaggagggaaatga---gtagttaa

tgcttaatgagtagagaggttccattttgtaagatgaaaag-agctctgc

ttgggaagctgaggttgggggatcgcttgagcccaggagtttgaggtaca

acagtgaggtatgatcacaacactgcactccagcctgggcaacagagaa-

gactctgtctctaagaaaaaaaaataatactaaaaataagagttctggag

atggacactggtgat----ggtggcacaa-cagtgtgcatgtacataaca

ctattgaactactacacacttaaaatggttaagatagtgaattttatatt

ctgtatac-tttaccacaatttttgaagatgaaaaaaaataatg------

--------------------------------------------------

--------------------------------------------------

--------------------------------------------------

--------------------------------------------------

--------------------------------------------------

-----------------------------CCTTC---------------A

TCCTTTAATATTAGCTTTCTGATCTGTCACAATATCTGTGGGTTTTAATA

TAACA-CAAACAAT-----TAACTTTGGCGAAAGAATCACCATAACCCT-

AGCTGATTATTCATCAAGGCAG----------------------------

----------------------CAAGCCAGTGGATTCTCTTGCC------

---------TTTAACTCCTTGAAGAAGGTGCAGGTAAG--CAGATATAG-

------CTGCTCCCATCACTGATGTC-TGGTATGG-TCTCTGGTCACAGG

CACGGAATGATTTCCTGAAGAGACAAAAGACGGGAAGATTTGCAACT

>human

CAGAGGAAATCCTGAAGAGGT----ACATCTAGCATTTAAAATGTATTTA

AACCTTTTG-TTAATAAA--------------------TGCCCTCTTCCA

---tacctatagcagacaaattcataga------------gacagaaagt

gaaatggtagttgccaggggcttggaaggagggaaaaga---gtagttaa

tgcttaatgagtagagaggttccattttgtaagatgaaaag-agttctgc

ttgggaagctgaggctgggggatcgcttgagcccaggagtttgaggtaca

acagtgagctatgatcacaacactgcactccagcctgggcaacagagcaa

gactctgtctctaagaaaaaataataataataaaaataagagttctggag

atggacagtggtgat----ggtggcacaa-cagtgtgaatgtacctaaca

ctattgaac-tgtaca--cttaaaatggttaagatagtaaattttatatt

ctgtatac-tttaccacaacttttgaagatgaaaaaataatg--------

--------------------------------------------------

--------------------------------------------------

--------------------------------------------------

--------------------------------------------------

--------------------------------------------------

-----------------------------CCCTCAT---CCTTTAACATT

AGCTTTAACATTAGCTTTCTGATGTGTCACAATATCTGTGGGTTTTAGTA

TAACA-CAAACAATTTGAGTAACTTTGGCAAAAGAATCACCATAACCCT-

AGTTGATTATTCATCAAGGCAG----------------------------

----------------------CAAGCCAGTGGATTCTCTTGCC------

---------TTTAACTCCTTGAACAAGATGCAGGTGAG--CACATACAG-

------CTGCTTCCATCACTAATGTC-TGGTATGG-TCTCTGGTCACAGG

CACGGAATGATTTCCTGAAGAGACAAAAGACGGGAAGATTCGCAACT

>chimpanzee

TAGAGGAAATCCTGAAGAGGT----ACATCTAGCATTTAAAATGTATTTA

AACCCTTTGGTTAATAAA--------------------TGCCCTCTTCCA

---tacctatagcagacaaattcataga------------gacagaaagt

gaaatggtagttgccaggggcttggaaggagggaaatga---gtagttaa

tgcttaatgagtagagaggttccattttgtaagatgaaaag-agttctgc

ttgggaagctgaggctgggggatcgcttgagcccaggagtttgaggtaca

acagtgagctatgatcacaacactgcactccagcctgggcaacagagcaa

gactctgtctctaagaaaaaaaaataataataaaaataagagttctggag

atggacagtggtgat----ggtggcacaaacagtgtgaatgtacctaaca

ctactgaac-tgtaca--cttaaaatggttaagatagtaaattttatatt

ctgtatac-tttaccacaactttcgaagatgaaaaaataatg--------

--------------------------------------------------

--------------------------------------------------

--------------------------------------------------

--------------------------------------------------

--------------------------------------------------

-----------------------------CCCTC---------------A

TCCCTTAACATTAGCTTTCTGATGTGTCACAATATCTGTGGGTTTTAGTA

TAACA-CAAACAATTTGAGTAACTTTGGCAAAAGAATCACCATAACCCT-

AGTTGATTATTCATCAAGGCAG----------------------------

----------------------CAAGCCAGTGGATTCTCTTGCC------

---------TTTAACTCCTTGAACAAGATGCAGGTGAG--CACATATAG-

------CTGCTTCCATCACTAATGTC-TGGTATGG-TCTCTGGTCACAGG

CACGGAATGATTTCCTGAAGAGACAAAAGACGGGAAGATTCGCAACT

>elephant

CAGATCAAATCCTGAAGAGGT----ATATCTGCTCTTTTAAATGTGTTTA

AATCTTTTGGTTACAGGA--------------------------------

--------------------------------------------------

--------------------------------------------------

--------------------------------------------------

--------------------------------------------------

--------------------------------------------------

--------------------------------------------------

--------------------------------------------------

--------------------------------------------------

--------------------------------------------------

--------------------------------------------------

--------------------------------------------------

--------------------------------------------------

--------------------------------------------------

--------------------------------------------------

---------------------------TGCCCTTTT--------------

--CTTTAGTATCAACTTTCTATTCTGTCACAGTGTCTGTGTGTTTCAGTA

TAACA-CAAATCGTTTGAGTAGCATGAGTGAAAGAGTCAGCATAAACCT-

GGTTGATCTGTAATTAGTGATATTCTTGTGT-ATCATGCATGATTAATCA

AGGTAG----------------CAAGCCAGTGGTACCTCTGTAG------

----------ATTTCCTTTTGACAGCATGAAGGAGTTG-CAGCTGAGCAC

ATACTTCAGCTCACACCACTGATGTC-TGGCACGG-TCTCTGGTCACAGG

CACTGAGCGATTTCCTGAAGAGACAG---GAGGGAAAGTTTGCAACT

>armadillo

CAATCCAAATCCTCATGTGGTGGA--------TTCTTTTCAGCGTGATTA

AAACTTTTGGTTGAAAA---------------------------------

--------------------------------------------------

--------------------------------------------------

--------------------------------------------------

--------------------------------------------------

--------------------------------------------------

--------------------------------------------------

--------------------------------------------------

--------------------------------------------------

--------------------------------------------------

--------------------------------------------------

--------------------------------------------------

--------------------------------------------------

--------------------------------------------------

--------------------------------------------------

---------------------------TGTTCTCTT--------------

--CTTTAATATCAACTTTCTAATCTGTCACAC----TGTGGGTTTTACTA

TAGCA-CTGATAGTTTGAGTAGCTTGAGTGAGAGGATCATCATAG-CCT-

GCTTGATCCACAATGAATGATATTCTTGTGTTATCATACATGATTAAGAT

AGGCAG----------------CAAGTCAGTGATTGCTTTGTAGAGCCCT

CATGCCTTTCACTACC-TTTGACCACCTGAAGGAGTTG-CAGGTGAGTAC

ATACTTCAGCTCACATGGCTCATGTC-TGGTATGG-TTTCTGGTGACAGG

CACTGAGTGATTTTCTGAAAAGACAGAAGACGGGAAGATTTGCAACC

Marker 3i:

>L1MB2

--------------------------------------------------

--------------------------------------------------

--------------------------------------------------

------------------------aaagaacttgaatagacatttctcca

aagaagatatacaaatggccaawaggcatatgaaaagatgctcaacatca

ctaatcatca--gagaaatgcaaatcaaaaccacagtgagatatc--acc

tcacacccattaggatggctactatc------------------------

-aaaaaaacagaaaataacaagtgttggcgaggatgtggagaaattgga-

acccttgtgcactgttggtgggaatgtaaaatggtgcagccgctwtgg--

--------------------------------------------------

--------------------------------------------------

--------------------------------------------------

--------------------------------------------------

--------------------------------------------------

--------------------------------------------------

--------------------------------------------------

--------------------------------------------------

--------------------------------------------------

--------------------------------------------------

--------------------aaaacagtatggaggttcctcaaaaaa---

-------------ttaaaaatagaa-ctaccatatgatccagcaatccca

cttctgggtatatatcca---aaagaattgaaagcagggtctc--gaaga

gatatttgcacacccatgttca-tagcagcattattcacaatagccaaaa

ggtggaagcaacccaagtgtccatcgacggatgaatggataaacaaaatg

tggtatatacatacaatggaatattattcagccttaaaaaggaaggaaa-

tcctgwcacatgctacaacatggatgaaccttgaggacattatgctaagt

gaaataagccagtcacaaaaagacaaatactgtatgattccacttatatg

aggtacctagagtagtcaaattcatagagacagaaagtagaatggtggtt

gccaggggctgggggg-agggggaaatggggagttgttgtttaatgggta

tagagtttcagttttgcaagatgaaaaa-gttctggagatcg--------

----gttgcacaacaatgtgaatatacttaacactactgaactgtacact

taaaaatggttaagatggtaaattttatgtt--atgtgtwttttaccaca

a-----aaaaaaa-------------------------------------

--------------------------------------------------

--------------------------------------------------

--------------------------------------------------

--------------------------------------------------

-------------------------------------

>cow

ATAAAGTAAATAGTCAGGAAGAGTAGAACAGGAGGATCTGAACCACAGGG

CTAACGTGTCACCATGAACTCGGAAATGCAGCAAGCAGGGGACGGATGGG

AGCAGTTGACAGGCCAACAGCCCTTCATTACCTAC-AACAAAGC-AAAAT

C---AGAAATTCTTTAATACA---aaaagatttcaacagacagttctaca

aagaagatatacaaatatccaataagcatgtgaaaagatactcaacatca

ctgatcatta--aggaaatgcaaatcaaaact-aaaatgacacac--tac

tcatacccgttaaaacagcaattatc------------------------

--aaaa--------tctacaccggggt--acataactaaaagaa-ctga-

aagcagggactcaaacacagattatttgtatatccatgttcaaa------

--------------------------------------------------

--------------------------------------------------

--------------------------------------------------

--------------------------------------------------

--------------------------------------------------

--------------------------------------------------

--------------------------------------------------

--------------------------------------------------

--------------------------------------------------

--------------------------------------------------

--------------------------------------------------

--------------------------------------------------

--------------------------------------------------

------------------------tgcagcattattcagaacagccaaaa

ggtggaagcaaaccaaatgtccatcaaaagattaactgacaaataaaata

tggtgtattcataaaatgtgactgtatttaggctt-acaagcaaggaaa-

ttttgacac-----ataacatggatgaaccttgaagacatt----aaagt

gaaataagccagtcacaaaaggatgaatactgtatgagtctacttagatg

aggtacctagagtagtcaaattcatagagacagaaaataggatggtggct

gccaagggctgcagagg-agaggcaatggaaag-tagtgcttaatggg--

tacagtttcagttaagaaagaggaaaag-gttctgaaaatgagagtggca

atggctgcacaacaaaatgaaatatatttaatgccactcaccaatacact

tt---atgatccaaatggtaaaattatgctacatat-actttaccacaga

a------AAAAATTCTTTAATAAA---GTCTGCACAACACACTTATCAAA

AAGATGCTCATAAAAAAAATGCTCATAACAGGATATACATCCCTAAGAAA

TTAGATATTTTTAAGAGTTTGCCTTCTG-----ATTGTAAGAAAAGGCCC

CATAAACACAAGACTGCCTTCTCAAAAACTCTACAAACCTATAATGTCAA

CAACATGAAGTCTTAGTTCCCGTTGCAGGGCCTTTAAGGTAGAACGCAGA

GCAGCTTGAGATTCTGACATCTTTACATTTTGTTTCA

>dog

ATACAGTAAGTAGTCAGGAAGAGAAGAGCAGGAGGATCTGAACCACAAGG

CCAATACATCACCATGAACTCGAAAGTGCAGCAAACAGGGGATGGATGGG

AGCAGTTGACAGGCCAAAAGCCCTTCGTTGCCTAC-AACAAAACAAAAAT

T---AAAAATTCCTAAGACAA----aagaactaaaatagacacttctcca

aagaagatatacaaatggccaataggcacgtgaaaagatatttgacatca

ctgatcagtaaggaaatgt-aaggaactactcatatatatatgatatata

tatcatatacatacacacacacacacacacatgcatacacatacacacat

actttcatgtatatataaacaccgttggtgaggatgtggaaaattggga-

acctctgtgcactgctggtgggaatgtaaatggtgcaaataatgtgg---

--------------------------------------------------

--------------------------------------------------

--------------------------------------------------

--------------------------------------------------

--------------------------------------------------

--------------------------------------------------

--------------------------------------------------

--------------------------------------------------

--------------------------------------------------

--------------------------------------------------

--------------------------------------------------

-----------------------aaaatcatatggtagtttctttaaaaa

-ttaatatagaattatataaccaaaagaactgaaagcagggacttgaaac

caaatagctgtacactcacactca-caacagcattattcctagtagccaa

aaggcagaagcaacccaagtgtccttcaacacatgaatggataaacaaac

tgtggtatattcatataatgaatagtattcaggctt-----aaaaaggaa

agatatcttgacacatggtacatgaatgaacgttgaagacatcacgctaa

gtgaaataagccagtcacaaaaggacaaatattgcataattcacctaata

tgagacacctagagtaattgaattcatagagacagaaggaagaatggtgt

tactaagagct---------agagggaatgataagttagtgtttaaaggg

cacaggattttagttaaggaagaggaaaagttctggaagtgatggtggca

acagttgtaca-acaatatgaatgtacttaatgtcaatgaactgtacagt

t-aaaattggttaaaatggaaaattttgttatg-----ttttaccacaca

a------AAAAAATACTTA--------GTTTGAACAACACACTCATTTGA

AAGA---------------TACTCATAACAGGAAATACAGCTCTAAGAAA

TTTGATACTCGTAAAA-GTTGATTCGTG----CTCTGTGAGAAAG--CCC

CTAT-AACACAAGATTGTCTTTATGTAACTCTACGAACCTATAATTTCAA

CAACATGAAGTCTTAGTTTCTGTTGCAGGGTCTTTAGGGCAGAAGACAGA

GCAGCTTGAGATTCTGACACTTTTACATTTTGCTTCA

>pangolin

ATACAGTAAATAGTCAGGAAGAGTAGAGCAGGAAGATCTGAACCACAGGG

CTAATACATCACCATGAACTCGGAAATGTAGCAAGCAGGGGATGGATGGA

AGCAGCTGACAAGCCAACAGCCCTTCATTACCTAT-AACAAAAGGAGAAT

T---AAAAATCCTTTAATACA---aaaggactttcatagacatttctcca

aagata----ccaagtggccaataagcacataaaaagatattcaacaaca

ctggtcattaaggaaatgcaaatt--------------------------

-----------aaaactacagtgaaatactacttcacacccattaagatg

gcaaatataaaataaataaataaagctattgatgaggatgtgaagaaatt

atcattttgcattgctactgggaatgt-----------------------

--------------------------------------------------

--------------------------------------------------

--------------------------------------------------

--------------------------------------------------

--------------------------------------------------

--------------------------------------------------

--------------------------------------------------

--------------------------------------------------

--------------------------------------------------

--------------------------------------------------

---------------------aaatggaatagttactatggaaaacgata

tggcaatttctccaaaaattaaatagttactatatgatccagcaattcta

gttctgggtatataacc---aaaagaactgaaagctgggacctgaacaca

gatatttgtacaccca----------------tattcacaacagacaaaa

ggtggaagcaatccaaatgtccattgacagatgaatggataaacaaaact

tggtgtatggaatggtattg---------agtctttaaaaggaaggaaat

tttgatgcatgttacaagatggatcaaccttga-agacattatgctaag-

--------------------------------------------------

---------------------------tg-aaataagctgaacagtgttt

gcc---aggagctggagagagggggatgggaagtttgtgtttaacaggta

cagagttttagttag--caagaaggaaaatctctgcagatgatggtggca

atggctgtaca-acaacatgaat---------------------------

------------------------------------------a-cacac-

------------TTACCTAATAAA---GTCTGAACAACATTTTCATCAGG

AAGA---------------TACTCATAACAGGAAATGTAACTCTAACAAA

TCTG-ACACTTGTAAAGGTTGATTTGTGTTCTAACTGTGAGAAAG--TCC

CTATAAATTCAGGATTGCCTTTTCATAACTCTACGAACCTATAATTTCAA

CAACATGAAGTCTTAGTTCCTGTTGCAGGGTCCTCAGGACAGAAGGCAGA

GCAGCTTGAGATTCTGACATTTTTACATTTTGCTTCA

>bat4b

ATACAATAAATAGTCAGGAAGAGTAGAGCAGGAGGATCTAAACCATAGGG

CTAATACGTCACCATGAACTCGGAAATGCAGCAAACAGGGGATGGATGGG

AGCAGCTGACAGGCCAACAGCCCTTCACTGCCTACAAACAA--CAAAA-T

G---AAAAATTCTTTAATACA---aaggaacttcagtacacagttctaca

aagaagatacacaa-tggccaataagcacatgaaaagatattcaacatta

ctgctcattaaagaaatgctaatcaaccc--cataatgaaataccactta

ccacttcacacc---------------------catgaggatggcaat-t

atcacaataaataaata-caagggttagggaggacatggagaactttga-

acc-ctgtacattgctgatgggaatgtaaatggtgccgttgctgtgaaaa

acgatatgatagcctggtgtggttcagtgattgagcactgacccatgaac

caggaggttactggttcaattcccagtcgaggcacgtgcccggttgcagg

tgcaatccccaataaggggtgtgcaggaagcagccaatcgatatttctct

ctcatcgatgtttctatctctctatccctctcccttcctctctctcaaaa

aatcaataaaaaacatttaaaaaaatgatatta---cttttccaaaaatt

acatata-------------------------------------------

--------------------------------------------------

--------------------------------------------------

--------------------------------------------------

--------------------------------------------------

--------------------------------------------------

--------------------------------------------------

----gaatta--ccatatgatctagcaattctacttctgggacttgaact

ctgatatctgtacacctctattcagagcagcattattcacagtagccaaa

aagtggaagcaactcaagtgttcatcgacagatgaatgga-taaaaaaaa

tgtggtatattcatacaatggaatagtattcatta-----------ggaa

agt-ttgacacatgccataataaggatgaaccttgaagatattatgttat

gtgaaataagccaatcacaaaagggcaaatattgtatgattccacttcta

tgaggtacctaacgt---caaattcatagagacagaaaataggatggtag

gtgtcagggactggagagggagatgatgacagctatt---gtttaatggg

tacag-ttttagttaggggaaaaggaaaagctcaggagatgatggtggca

atagttacaca-acaacgcgaatatacttaaagccactaaactgtacact

t-aagaacagttaaaatagcaaattttgtta--tgtata-ttttaccaca

a---AAATTCTTTACTAAAATC-----TGAAGAACACACTCCTC---TAA

AAGA---------------TACTAATAACAGGAAATATGGCTCTAAGAAA

TCTGACACTTGTGAGAGCTGATCTGTGT---TCTGGTTGTGAGAAAGCCC

TCATAAACACGGGATTGCCTTTTCGTAACTCTACAAACCTATAATGTCAA

CAACATGAAGTCTCAGTTTCTGTTGCAGTG-TTTCAGGGCAGAAGGCAGA

GCAGCTTGAGATTCTGACATTTTTACATTTTGCTTCA

>mole

ATACAGTAAGTAGTCGGGAAGAGCAGAGCAGGAGGATCTGAACCACAAGG

CTACGACATCAGCATGAACCCGGAAATGCAGCAAGCAGGGGGTGGATGGG

AGCAGCTGACAGGCCAAAAGCCCTTCATTACCTAC-AATGAAGCAAAAAT

A---AAAAATTACTTACTAAA---aaaggacttcagtaaacaattctcca

a-----------------------------tgaaaataccgaaa-ggcca

ataggcacgtaaaaagatattcaacgtcactgaa---taaggaaatgtaa

atc---------------------------------------acaaccgt

gaaacaattcaagctattaggatgagtgttggtgaggatatggagaaag-

tgcattgcgcattgctgataggaatataaatggtgttgttcctgtgg---

--------------------------------------------------

--------------------------------------------------

--------------------------------------------------

--------------------------------------------------

--------------------aaagtaatatggcgatttctcc--------

--------------------------------------------------

--------------------------------------------------

--------------------------------------------------

--------------------------------------------------

--------------------------------------------------

--------------------------------------------------

--aaaatgtaagtacagaa--------ttaccattag------------t

cagaaattccacttctaaccaaaagaactgaaagcagggac-tcaaacac

aaatcttagacacccatattcacaagcagaacttttcacaatagtc-aaa

gagggaagcaattcagtgt-----ccatcgatagatgaataaacaaaatg

tggtatgttcatataatgcaacaacgttcagagttaaaaaggaaggaaat

tgtgacaca-agctacaacatggatgatcttcattatgct-----aaatg

aaaaaaagccaatcgcaaaagg-ataattaaattatttgatgattctact

tatatgcggtactaagagtagtcaaactgataaaaagcagaatgttagtt

gccagggctagtgaagg-agggggtatggggagttagtgtttagtggtca

tagtgtttcaattatggaagatgagaaaactctagg--atggtggtggta

atggctgca-taataatgttaa-gtacttaatgccactgacctatacatt

tt-aaagtggttaaaatgataaattttgt-tatgtatattttactaccaa

a------AAAATGTCCTGGATAAA---ATCTGAA---CACGTTATCTGAA

AAAT---------------ACTCATAATAAG--AATACA---GTAACAAA

TC---CCATACTTATA-AATGAGTTATATTCAGTTTGTGAGAAAGACAAT

CCTAC-ACAAGGATTGTCTTTTTTGGAACATTACCAACCTATAATGTCAA

CAACATGAAATCTTAGTTTCTGTTGTAAGGTCTCTAGGGCAGAAGGCAGC

GCAGCTTGAGATTCTGACATTTTTACATTTTGCTTCA

>mouse

ACACGATAAATAGTCTGAAAGAACAGAGCTAGAGGACCGGAACCACAGAG

CTACCGCGTCAGCATGTACTCGGCAGTGCAGCACACAGGGGGTGGATGGG

AGCAGCTTACAGGCCAACAGCCCTTCATTGCCTGC-AACACAACAAAAAG

G---AGAAATTACTTTTAAA----aatggacttcaacagacaatcctcca

cagacatctacaaacgggcca-caagcacgggaaaagatgttta---tca

tcaatcaccaaacaa----gc------accttagggagagcacttcactc

ttatttggaagactattttatatatt---------------------aaa

aaacaaa-aca-------------------------aaacttgaa-----

--aaagtgctggagtggtggtgaa-----------tgcctt---------

--------------------------------------------------

--------------------------------------------------

--------------------------------------------------

--------------------------------------------------

--------------------------------------------------

--------------------------------------------------

--------------------------------------------------

--------------------------------------------------

--------------------------------------------------

--------------------------------------------------

--------------------------------------------------

--------------------------------------------------

--------------taatcca------agatgagagcaagaatatcaata

ggtattcccacactcacgtaca-cagcagcactgtccatatcagacaaaa

agtagaagtgtctcaagtacttagttaacaggctaac-agatactacagt

aataatatgatgtataataataaaatgtttaaagcgttaaagagaaaaag

aaaggctaaatgatagttaattatatact---------------------

--------------------------------------------------

--------------------------------------------------

--------------------------------------------------

--------------------------------------------------

--------------------------------------------------

-------------------------------gtga--acttttcaatcaa

-------ATA--CTCCTTAATACA---GTCTGGAAACACACTTATTTTAA

AGA----------------TACTCATGATAAGAA-----TCTCTAAGAAG

TATGACACTCAGAAAA-----------------------AAAAAA--TCT

ATATTCACATGGGGCTGCCGTTTCTTAATTCTACACACCTATGACGTCAA

TCACACGAAGTCTCAGGTTCTGCTGCAGCTCTGTTAGGACAGAAGTCAGA

GCAACTCCAGGCTCTGATATTTTTATAGTTTGCTTCA

>rhesusmonkey

ATACAGTAAATAGTCAGGAAGAGTAGAGCAGGAGGATCTGAACCACAGGG

CTAATACATCTGCATAAACTCGGCAATGCAGTAAGCAGGGGATGGATGGG

AGCAGCTGACAGGCCAATAGCCCTTCATTGCCTAC-AACAAAACAAAAGT

G---AAAAATGATTTAATGCA---aaaggacttaaacagacatttctcca

agaaaata-cacaaatggctaataagcacatgaaaagatacccagtatca

ctaatcaccaaggaaa-ctgcaaattgaaaccacaatgagatactacact

tcatacccattaggatggctaatatt--------------------aaag

aaaaaaaacaggtaataacaagtgttgatgaagatgtggagaaactgga-

accccagtacgttgctggtgggaatataaaatgatgcagttgctgtg---

--------------------------------------------------

--------------------------------------------------

--------------------------------------------------

--------------------------------------------------

--------------------aaaa-tgctatggtggtttctcaaaaaatt

aaatctaggccaggcacagtagttcatgcctgtaatcccagcactttggg

aggctgaggcaggcagattacttaagctcaggagttctagacctggctgg

gcaatatggtgaaaccccgtctctactaaaaaatacaaaaaattagctga

gcatggtgccacacaccagtaatcccagctactcgggaggctgaggcata

agaatagcttg--catggaaggtggaggttgcagtgagctcagatcgcac

-actgcactaaagcatgggcaacaaagtgagcctctgtctcaaaaaaaaa

aaaaa-----ttacatctataattatcatgtatataatctactaattcta

cttctgggtata--taaccaaataaagaacagaaagtagggacacaaaca

ggtatttgtataaccctgttca-tagcagcattactcacaatagccaaaa

ggtggaagtaacccaagtatccatttacagatgaatggataaacaaaatg

tatca--catt-----------------------------------aaaa

ttcagacacattctacaatatggatcaaccttgaagatattatgctcagt

gaaataagccagatataaaaggacgagtattgtatgattccacattatat

aaggtacctaacatagtcaagttcctagagacaagaggtaaaatggtagt

tgccaagaggtagagaggaaggggaaaggctgagttagtgtttaatgggt

atagtgttccagctgaggaagataaaaaagttctgaagacagatgatggt

gatagttgcacaacagtgtatatatacttaatgccatttaactacatg-t

ttaaaaattgttaaaatggtatattttatat--atgtgt-atttt--cca

a---taaaaaaaTTATTTAATACA---GTCTGAAAAGCACATTCATCGGA

AAGA---------------CAGTCATAATAGGAAATAAAGCTCTAAGAAA

TCCGATACTTGTAAGA-GTTAATTTGTGCTCCAATTATAAGAAAG--CTC

CTATAAACACAGAATAGCCTTTTCATAACTCTACAAACCTATAATTTCAA

TAATATGGAGTCTTAGTTTCTGTTGCAGAGTCTTTAGTGCAGAAGGAAGT

GCAGCTTGAGATTCTGACATTTTTACATTTTGTTTCA

>human

ATACAGTAAATAGTCAGGAAGAGTAGAACAGGAGGATCTGAACCACAGGG

CTAATACATCTGCATGAACTCGGCAATGCAGTAAGCAGGGGATGGATGGG

AGCAGCTGACAGGCCAATAGCCCTTCATTGCCTAA-AACAAAACAAAAGT

G---AAAAATGATTTAATGCA---aaaggagttaaacagacatttctcca

agaagata-cacaaatggctaataagcacatgaaaagatactcaatatca

ctaaccactaaggaga-ctgcaaattgaaaccacagtgagatactatact

tcatacacattaggatggctaatatt---------------------aaa

gaaaaaaacagataataacaaatgatgatgaagacgtggagaaactgga-

accctggtacattgctggtgggaatataaaatggtgcagttgctgtg---

--------------------------------------------------

--------------------------------------------------

--------------------------------------------------

--------------------------------------------------

--------------------aaaa-tgatatggtggtttctcaaagaatt

aaatctaggccaggtgcagtagttcacacctgtaatcccagcactttggg

aggctgaggcgggcagatcacttaagctcaggagttctagacctgcctag

gcaatatggtgaaaccccatctctactaaaaaatacaaaaaattagctga

gcatggtgccacacacctgtaatcctagctactcgggaggctgaggcacg

agaatagcttgaacacggaaggtggaggttgcagtgagctcagatcgcac

cactgtactccagcatgggtaacaaagtgagcctctgtctcaaaaaaaaa

aa-----------ttaaatctaaaa-ttatcatataatccaccaattcca

cttctgggtata--taaccaaataaagaactgaaagcagggacacaaaca

ggtatttgtataaccttgttca-tagcagcattactcaccatagccaaaa

agtggaagtaacccaagtatccatttacagatgaatggataaacaaaatg

tatca--catt-----------------------------------aaaa

ttcggacacattctacaacatggatcaaccttgaagatattatgctcagt

gaaataagccagatacaaagggacaagtattgtatgattccacttatata

aggtacctaacacagtcaagttcatagagacaagaggtaaaatggtagtt

gccaagaggtggagaggaaggggaaaggcggagttagtgtttaatgggta

tagtgttccagttgaggaggataaaaaa-gttctgaagatagatgatggt

gacagttgcacaacagtgtaaatacacttaatgccacttaactatatg-t

ttaaaaattgttaaaatggtatattttatgtatgtat-atttt-cca-at

a------AAAAATTATTTAATACA---GTCTGAAAAACACATTCATCTGA

AAGA---------------CAGTCATAATAGGAAATAAAGCTCTGAGAAA

TCTGATACTTGTAAGA-GTTGATTTGTGTTCCAATTATAAGAAAG--CTC

CTATAAACACAGAATAGCCTTTTTATAACTCTACAAACCTATAATCTCAA

TAATATGGAGTCTTAGTTTCTGTTGCAGAGTCTTTAGTGCAGAAGGAAGT

GCAGCTTGAGATTCTGACATTTTTACATTTTGTTTCA

>chimpanzee

ATACAGTAAATAGTCAGGAAGAGTAGAACAGGAGGATCTGAACCACAGGG

CTAATATATCTGCATGAACTCGGCAATGCAGTAAGCAGGGGATGGATGGG

AGCAGCTGACAGGCCAATAGCCCTTCATTGCCTAA-AACAAAACAAAAGT

G---AAAAATGATTTAATGCA---aaaggagttaaacagacattcctcca

agaagata-cacaaatggctaataagcacatgaaaagatactcaatatca

ctaaccactaaggaga-ctgcaaattgaaaccacagtgagatactatact

tcatacccattaggatggctaatatt---------------------aaa

gaaaaaaacagataataacaaatgatgatgaagacgtggagaaactgga-

accctggtacattgctggtgggaatataaaatggtgcagttgctgtg---

--------------------------------------------------

--------------------------------------------------

--------------------------------------------------

--------------------------------------------------

--------------------aaaa-tgatatggtggtttctcaaagaatt

aaatctaggccaggtgcagtagttcacgcctgtaatcccagcactttggg

aggctgaggcgggcagatcacttaagctcaggagttctagacctgcctag

gcaatatggtgaaaccccatctctactaaaaaatacaaaaaattagctga

gcatggtgccacacacctgtaatcctagctactcgggaggctgaggcacg

agaatagcttgaacacggaaggtggaggttgcagtgagctcagatcgcac

cactgtactccagcatgggtaacaaagtgagcctctgtctcaaaaaaaaa

aaa----------ttaaatctataa-ttatcatataatccaccaattcca

cttctgggtata--taaccaaataaagaactgaaagcagggacacaaaca

ggtatttgtataaccctgttca-tagcagcattactcaccatagccaaaa

agtggaagtaacccaagtatccatttacagacgaatggataaacaaaatg

tatca--catt-----------------------------------aaaa

ttcggacacattctacaacatggatcaaccttgaagatattatgctcagt

gaaataagccagatacaaagggacaagtattgtatgattccacttatata

aggtacctaacacagtcaagttcatagagacaagaggtaaaatggtagtt

gccaagaggtggagaggaaggggaaaggcggaattagtgtttaatgggta

tagtgttccagttgaggaggataaaaaa-gttctgaagatagatgatggt

gacagttgcacaacagtgtaaatatacttaatgccacttaactatatg-t

ttaaaaattgttaaaatggtatattttatgt--atgtat-atttt--cca

a---taaaaaa-TTATTTAATACA---GTCTGAAAAACACATTCAACTGA

AAGA---------------CAGTCATAATAGGAAATAAAGCTCTAAGAAA

TCTGATACTTGTAAGA-GTTGATTTGTGTTCCAATTATAAGAAAG-CTCC

TATAAACACAGAATAGCCTTTTTTATAACTCTACGAACCTATAATCTCAA

TAATATGGAGTCTTAGTTTCTGTTGCAGAGTCTTTAGTGCAGAAGGAAGT

GCAGCTTGAGATTCTGACATTTTTACATTTTGTTTCA

>tenrec

AGAGAGTAAATAGTCCGGCAGCGCAGAGCAGGAGGACCTGAGCCACAGGA

CTAAGACATCAGCATGAACGCGGAAATGCAGCAGGCAGGGTCTGGACGGG

AGTAGCTGACAGGCCAAAAGCCCTTCAGTACCTGC-AACAAAACACAGAA

C-------------------------------------------------

--------------------------------------------------

--------------------------------------------------

--------------------------------------------------

--------------------------------------------------

--------------------------------------------------

--------------------------------------------------

--------------------------------------------------

--------------------------------------------------

--------------------------------------------------

--------------------------------------------------

--------------------------------------------------

--------------------------------------------------

--------------------------------------------------

--------------------------------------------------

--------------------------------------------------

--------------------------------------------------

--------------------------------------------------

--------------------------------------------------

--------------------------------------------------

--------------------------------------------------

--------------------------------------------------

--------------------------------------------------

--------------------------------------------------

--------------------------------------------------

--------------------------------------------------

--------------------------------------------------

--------------------------------------------------

--------------------------------------------------

-------TGTCA-TTGCTAACACG---GCCCAAACGACACACTCACCTGC

AAGA---------------CACTCGGCAA--GAAATAACCGCTCGCTCTG

A-GAACTCTGACACCA-GTAAGAGAACTGGTTTGGATGACACGAGG-TCT

CAATTACAACCTGGTTGTCGTTTTATAATGTTACCAACCTATCATGTCAA

CAACATGAAGTCTTAGCTTGTGTTGCAGGGTCTTTAGAACAGAAGGCAGG

GCAGCTTGGGACGCGGACATTTTTACATTTTGCTTCA

>seacow

----AGTACATAGTCGGGAAGAGCAGAGCAGGAGGATCTGAACCACAGGG

CTAGTACATCAGCATGGACTCGGAAATGCAGCAGGCAGGGCATGGATGGG

AGCAGCTGACAGGCCAACAGCCCTTCATTGTCTGC-AATAAAACACAAA-

--------------------------------------------------

--------------------------------------------------

--------------------------------------------------

--------------------------------------------------

--------------------------------------------------

--------------------------------------------------

--------------------------------------------------

--------------------------------------------------

--------------------------------------------------

--------------------------------------------------

--------------------------------------------------

--------------------------------------------------

--------------------------------------------------

--------------------------------------------------

--------------------------------------------------

--------------------------------------------------

--------------------------------------------------

--------------------------------------------------

--------------------------------------------------

--------------------------------------------------

--------------------------------------------------

--------------------------------------------------

--------------------------------------------------

--------------------------------------------------

--------------------------------------------------

--------------------------------------------------

--------------------------------------------------

--------------------------------------------------

--------------------------------------------------

----tTTTACATTTTTTAACTTA----GTCTGAACAACACACTCATTGGA

AAGT---------------TTCTCCTAACAGGGAACACAGCTCTAAGAAA

CCTGACGCTCGTCAGA-GTTAGTGGGTGCTCCGACTGTGAGAAAG--CCC

CCACAAACACAGGGCTGCCTTTTTATAACTGTACCAACCTATAACGTCAA

CAATATGAAGTCTCAGCTTCTGTTGCAGGGTCTTTAGGGCAGAAGGCAGA

GCAGCTTGAGATTCTGACATTTTTACATTTTGCTTCA

>elephant

ATACAGTACATAGTCAGGAAGAGTAGAGCAGGAGGATCTGAACCACAGGG

CTAATACATCAGCATGAACTCGGAAATGCAACAGGCAGGGGACAGATGGG

AGCAGCTGACAGGCCAACAGCCCTTCATTGCCTGC-AACAAAACAAAAAT

T-------------------------------------------------

--------------------------------------------------

--------------------------------------------------

--------------------------------------------------

--------------------------------------------------

--------------------------------------------------

--------------------------------------------------

--------------------------------------------------

--------------------------------------------------

--------------------------------------------------

--------------------------------------------------

--------------------------------------------------

--------------------------------------------------

--------------------------------------------------

--------------------------------------------------

--------------------------------------------------

--------------------------------------------------

--------------------------------------------------

--------------------------------------------------

--------------------------------------------------

--------------------------------------------------

--------------------------------------------------

--------------------------------------------------

--------------------------------------------------

--------------------------------------------------

--------------------------------------------------

--------------------------------------------------

--------------------------------------------------

--------------------------------------------------

-------TTACATTCTTTAATGTA---GTCTGAACAACACACTCATCGGA

AAGA---------------CTCTTCTAACAGGGAACACAGCTCTAAG-AG

ACCTGACCTCGTCAGA-GTTAATGTGTGCTCCGATTGTGAGAAAG--TCC

CCACCAACACAGCGCTGCCTTTTTATAACTATACCAACCTATAATGTCAA

CAATATGAAGTCTCAGCTTCTGTTGCAGGGTCTTTAGGGCAGAAGGCAGA

GCAGCTTGAGATTCTGACATTTTTATATTTTGCTTCA

>sloth

ATACAGTAAATAGTCAGGAAGAGTAGAGCAGGAGGATCTGAACCAGAGGG

CTAATACATCAGCACGAACTCGGCAATGCAGCAGGCAGGGAATGGATGGG

AGCAGCTGACACGCCAACAGCCCTTCATTGCCTAC-AACAAAATGAAAAT

G-------------------------------------------------

--------------------------------------------------

--------------------------------------------------

--------------------------------------------------

--------------------------------------------------

--------------------------------------------------

--------------------------------------------------

--------------------------------------------------

--------------------------------------------------

--------------------------------------------------

--------------------------------------------------

--------------------------------------------------

--------------------------------------------------

--------------------------------------------------

--------------------------------------------------

--------------------------------------------------

--------------------------------------------------

--------------------------------------------------

--------------------------------------------------

--------------------------------------------------

--------------------------------------------------

--------------------------------------------------

--------------------------------------------------

--------------------------------------------------

--------------------------------------------------

--------------------------------------------------

--------------------------------------------------

--------------------------------------------------

--------------------------------------------------

-------AA-AATTCTTTAATACT---GTCTGAACAACACACTTATCTGA

AAGA---------------TACTCATAATAGGAAATAAAGCTCTAAGAAA

TTTGATAGTTATAAGA-GTTAATTTGTGTTCTGATTATGAGAAAG--CCC

CTATAAACATAGGACTGCCCTTTCATAAGTTTACGAACCTATAACATCAA

CAACATGAAGTCTTAGTTTCTGTTGCAGGGTCTTTAGGGCAGAAGGCAGA

GCAGCTTGAGAGTCAGGCACTTTTACATTTTGCTTCA

>armadillo

ATACAGTAAATAGTCAGGAAGAGTAGAGCACGACGATCTGAACCATAGGG

CTAATACATCAGCATGACCTCGGAAATGCAGCAGACAGGGGATGGATGGG

AGCAGCTGACAGGCCAGCAGCCCTTCATTGCCTAC-AACAAAATGAAAAT

T-------------------------------------------------

--------------------------------------------------

--------------------------------------------------

--------------------------------------------------

--------------------------------------------------

--------------------------------------------------

--------------------------------------------------

--------------------------------------------------

--------------------------------------------------

--------------------------------------------------

--------------------------------------------------

--------------------------------------------------

--------------------------------------------------

--------------------------------------------------

--------------------------------------------------

--------------------------------------------------

--------------------------------------------------

--------------------------------------------------

--------------------------------------------------

--------------------------------------------------

--------------------------------------------------

--------------------------------------------------

--------------------------------------------------

--------------------------------------------------

--------------------------------------------------

--------------------------------------------------

--------------------------------------------------

--------------------------------------------------

--------------------------------------------------

-------AAAAATTCTTTAATATA---GTTTGAATAACACACTCATTTGA

ATGA---------------GATTCATAATAGGAAATAAAGTTCTAAGAAA

TGGAAT--------------------------------------------

-CCACCACATGAGG------------------------------------

--------------------------------------------------

-------------------------------------

Marker 3j:

>L1MB3

--------------------------------------------------

--------------------------------------------------

--------------------------------------------------

------------------------------------------taacataa

aa----tttaccatttta--------------------------------

--------------------------------------------------

--------------------------------------------------

--------------------------------------------------

--------------------------------------------------

--------------------------------------------------

------------------------------------accattttt---aa

gtgtacagttcagtggcattaagtacattcacattgttgtgc--------

--------------aaccatcaccaccatccatctccagaactttttcat

cttcccaaactgaaactctgtacccattaaacamtaactcc-ccattccc

ccctccccccagcccctggcaaccaccattctactttctgtctctatgaa

tttgactac-tctaggtacct---catataagtggaa-------------

--------------------------------------------------

--------------------------------------------------

--------------------------------------------------

----------------------------------------------tcat

acagtatttgtcctttt-gtgactggcttatttcacttagcataatgtc-

--------------------------------------------------

--------------------------------------------------

--------------------------------------------------

--------------------------------------------------

--------------------------------------------------

----------------cctcaaggttcatccatgttgtagcatgtgtcag

aatttccttcctttttaaggctgaataatattccattgtatgtata----

--------------------------------------------------

--------------------------------------------------

--------------------------------------------------

--------------------------------------------------

--------------------------------------------------

--------------------------------------------------

---------------------------taccaca----------------

--------------------------------------------------

--------------------------------------------------

--------------------------------------------------

--------------------------------------------------

--------------------------------------------------

--------------------------------------------------

--ttttgtttatccattcatccgtcg-atggacacttgggttg-cttcca

ccttttggc-----------------------------------------

--------------------------------------------------

--------------------------------------------------

--------------------------------------------------

--------------------------------------------------

--------------------------------------------------

--------------------------------------------------

--------------------------------------------------

--------------------------------------------------

--------------------------------------------------

--------------------------------------------------

--------------------------------------------------

--------------------------------------------------

------------------------------------------

>cow

TAAGTACCTTTTCAATGG--CTG---------TACAGAAAAAAAAAAA--

ATCACTGTTTTGAAAATAACAGAGTA---ATGTGATGGGAGGGAAATGTA

AGTCACAATGTGCCAGAAAAGCAGAGAGAGTAACTAATGTTGTTTCAGAA

ATAAC------TTTTAAAAG--TTATAGTGAAATATACA---taacataa

aa----tttaacattctta-------------------------------

--------------------------------------------------

--------------------------------------------------

--------------------------------------------------

--------------------------------------------------

--------------------------------------------------

--------------------------------------------------

-------------tcatgttaagtgtgttcattgttgtgcac--------

---------------ccatcaccaccatgtacctccagaac-tttttcat

catcccatactgaaa--ctgtatcctttaaacatgatctcccccgttccc

----------------tggcattaaccattccactttctgtttctatgaa

ctggactat-tctcagtatct---tgtataagtagaa-------------

--------------------------------------------------

--------------------------------------------------

--------------------------------------------------

----------------------------------------------tcat

acagtatttgaccttttataactgacttaatttca-ttagcatagtgtt-

--------------------------------------------------

--------------------------------------------------

--------------------------------------------------

--------------------------------------------------

--------------------------------------------------

----------------cctcaaagtttatccatgtagcagtatgtggtag

aatttccttccttttgaaggctgtgtaatatcccattatgtgt--c----

--------------------------------------------------

--------------------------------------------------

--------------------------------------------------

--------------------------------------------------

--------------------------------------------------

--------------------------------------------------

---------------------------tataccac---------------

--------------------------------------------------

--------------------------------------------------

--------------------------------------------------

--------------------------------------------------

--------------------------------------------------

--------------------------------------------------

-atttatttatccatttagctttcag---aaacatttggattg-tttcca

ccttttgaa---CAAAATGACTGTTTTTAA--------------------

--------------------------------------------------

--------------------------------------------------

--------------------------------------------------

--------------------------------------------------

--------------------------------------------------

-----AACCATTAAATCCAGTGGTCTAATATGGTTGTAATAGAGTTGAAC

AACTGTAAAAAGAGGGAGA-CAATTTACAATTACTTGT---CATAT----

--------------------------------------------------

--------------------------------------------------

--------------------------------------------------

--------------------------------------------------

--------------------TAAAATTTTTATAT-GAAATATAGAAAGGA

GCATTTAAATGTGGTTGTCTTTACAGGGATAATTATACCCTA

>dog

TAAGTA-GTTTCACAATGA-CTA---------TACAGAAAAAAG------

-TCGCTGCTTTGAAACTAACAAAGA-GTTGTATGATGCAAGGAAACTGTA

TCTTACAATGTGCCAGAAAAG-GGAAACTGTAACTAATGTGT---CAAAA

CA-------CTCTTAAAAAAA-TTGTGGCAAAATATACA---taacataa

aaccattttagccatttc--------------------------------

--------------------------------------------------

--------------------------------------------------

--------------------------------------------------

--------------------------------------------------

--------------------------------------------------

------------------------------------------------aa

gtgtgcagctcagtggtattaagtacatttacattgttgtgc-----aat

tgccagca-ccgtccatctccaaaacatccatctccaaaacattttta-t

tatcccaaactgaaa--ctgtacccatgaaacactaactccattattccc

----------------gggcagtcatcattctgccttctatcttggtgaa

tttaactgc-tttaggtacttctgcatagaagtagaa-ttgtacaggatt

ttcctttttttttttaattttttaaaaatttatttgtgatagtcacacag

agaaagagagagaggcagagacataggcagagggagaagcaggctccatg

caccgggagcccgacatgggattcgatcccgggtctccaggatcgtg---

CCCTGGGCCAAAGGCAGGCGCTAAACCGCTGCGCCACCCAGGG---ttcc

cAagga-ttttcctttt-gtgattgacttatttca-ttagc--tatgcc-

--------------------------------------------------

--------------------------------------------------

--------------------------------------------------

--------------------------------------------------

--------------------------------------------------

----------------cctcaaggttcatccatggcattgcatatgtcag

gattttcttcctgtatgaagctgagtaacaccccattgtgtaaagg----

--------------------------------------------------

--------------------------------------------------

--------------------------------------------------

--------------------------------------------------

--------------------------------------------------

--------------------------------------------------

---------------------------agcaccac---------------

--------------------------------------------------

--------------------------------------------------

--------------------------------------------------

--------------------------------------------------

--------------------------------------------------

--------------------------------------------------

-attttgcatattca-tcatcggttattg-aacatttgagttgttttcca

ccttttgac---AGAAATG---GCTTTTAA---AAAAAAaa-agatttta

ttttatctattcatgagagacacagagagggaagaagagacataggcagt

gggagaagcaggctccccgtagggaacctgatgcgggactggatcccagg

gccctaggatcattccctgagctgaaggcagatgctcaaccactgaacca

cccaggagtccc-AGAAGTGACTATTTTTAA-------------------

--------------------------------------------------

-----AAACATTAAGTCCAGGGGTCTAACATGGTTCTAATAGAGGTAAAC

AACTTCC-AAAGAGGGAGC-TAATTTATAGT--TTTGT---CATATTAAA

ATTTTTATTTGAGGGTAAcctgggtggctcagttggttaagcatctgcct

ttggctcaggtcatgatcccccagtccttggattgagccccacatcaagc

tccctactcagtggggaacctgcttctccctctccctctgcagctccctc

tgtcactctccctgcatgtgcatgcgtgctctctgtcgaataaataaata

aagtctttttaaaaaTAAAATAAAATTTTTATATAGAAGTATAGAGAGAA

GCATTTAAATGTGGTTGTCTTTACAGGGATAATTACACCCTA

>mouse

TACGTGA---TCGGG--GCCACAAGGAGATATTTCAGAAA----------

-TAGCT-TTTTAAAGATTGTTTTATA-----GGACCTGAAAA------TT

GTATATAATG-TACATAGAAATTACCATTTTAACCTTGTGTGTGTGTTGT

GGA---------TTGCACGTGGTAGGCAGGCCGTCCTCC---acagaatg

cccacct-cacagacctc--------------------------------

--------------------------------------------------

--------------------------------------------------

--------------------------------------------------

--------------------------------------------------

--------------------------------------------------

-------------------------------------------tgtaagc

acacacttggctggcgtttcaagtgc-ttc-ctgtattgttgatctctgt

acctttcccatcctcctgaataattctacattcattaaacactttt----

--------------------------------------cccaccatatac

----------------aggcagccaccagtctgctat---tctctgtgcc

tctgactacatcca--tacat---tagatgcatggga-------------

--------------------------------------------------

--------------------------------------------------

--------------------------------------------------

----------------------------------------------ccat

acagcact-gggctgaa-gtgtatggattatgtcacttaacata-tgtc-

--aagtgacatttcctaccaaagata-------gggtaccaagaacagac

caaccttttattcttaccaaagcccaacatggaaaaccaatgggctaact

tgaagaacttttgagacatgtgggtg-gggactcttctggcatcaggcat

gactcttgggcagcagagaagcccttcctagcatgggtgaggccctacaa

aggctgtgtccctggagctccctgcatgaccacaggagctctgttggtca

gagtctcttctcc---cctgctgctgctttacctcacccaggggagtggc

tttgtaga-tcttttac-gttttcataa---cttcctggatctttgttt-

----ttagtttcatttaatgtgaatagtttttcacctgcatgtatgcctg

tgcaccatac-gcatgtagtaccctcagaggccacaagggggcaacggaa

ctgatgttccagatggttgttagttgccatgtgggtgctggaaattcaac

tgcaggtaggtcttctacaacagcagcaggtgctcttcctgggtcttgtg

agcctctgtg---TCTTTCCAGGGGCAGTATTTGAATTCAATTCAGAGAA

ATTGTCCAACAACCTGTCTTTAAAGTCTAGCCATGGTA-TTCCAAGCAGA

AAGGTTTTTTTCTTTAT-CCTTTG---tatatacaaataaaa--------

--------------------------------------------------

--------------------------------------------------

--------------------------------------------------

--------------------------------------------------

--------------------------------------------------

--------------------------------------------------

--tagttttta---atctatctgtttacgtgtcctttgggactatttcca

tctttggac---AGAAGTGAC--TATTTAA--------------------

--------------------------------------------------

--------------------------------------------------

--------------------------------------------------

--------------------------------------------------

--------------------------------------------------

-----GACCATTATCAGATCCAGTG-------ATTTTA-----GCACAGG

AAAATAACAAAAGGGGAGA--TTTTCATAATCGTTTATTTACATAAA---

--------------------------------------------------

--------------------------------------------------

--------------------------------------------------

--------------------------------------------------

--------------------TTATTTTGTTCTATGAACTATTGAGAGGGT

CACATGAGTCTGGTTTGTCTTTCCAGGGATAATTACACCCTA

>rat

ATTGTGA-CACAGGGAAATTATG-ACTGATATTTCAAAAA----------

-CAGCT-TTTTAAAAATTGTATTATA------------------------

----------GAACATAAGAATTACCATTTTAACCTTGTGTGTGCTGTGT

-----------CTTGTACCTAGTGGGCAGGCCCTCCTCC---acagagtg

cccacaa-cagccctctg--------------------------------

--------------------------------------------------

--------------------------------------------------

--------------------------------------------------

--------------------------------------------------

--------------------------------------------------

-------------------------------------------ta--agc

acatactcgg--ccgacttcttgagtgcattctggattgtcctctcca--

accttttcatcctcctgccataattctacattcattaaacactttt----

--------------------------------------ccctccatctcc

----------------aggcagccaccagtctactat---tctctgtgac

tctgactgcatact--caaat---tagatacatgaga-------------

--------------------------------------------------

--------------------------------------------------

--------------------------------------------------

----------------------------------------------ccat

acagctct-tggctgta-ttatgggcaatttt--acttagcata-tgtc-

--aagtggcatttccttggaaaacatgaacaaacctctcttcatcccaga

tcagcttaggattctaccagagcccaacatggcaaaccagtgggctttct

tgaagtacttt-cagaagtgtggttgagggattcttctggcagcaggcat

gactcaagggtaacagagaagcccttcctagcatgggtgagg-cccacaa

aggctgtgtccctggagttcctgcacgactt-caggagctctgttggtca

gagtatcttctct---cctgctgctactttacctagcccaggggag-gac

tttggagatcctatagacggttttgata--gctccctgggtctttgttt-

---tttaatttcatttcatgtgaataggttttcacctgcatgcttgtcta

tgccccacaccacatgcatgcagt--------------------accctc

tccggccagaagatggctgccaattgtaatgtgggtgctgggaattcaac

a-taggt----catctataaaagcatca-gtgctcttcctgagtctctc-

----tctg-----TCTTTACAGGGGCCG----------------------

--------------------------------------------------

--------------------------------------------------

--------------------------------------------------

--------------------------------------------------

--------------------------------------------------

--------------------------------------------------

--------------------------------------------------

--------------------------------------------------

-------------tttctatctgtttatgtgttcttaggaactatttcca

ccttttaac---GGAAGTGGC--TATTTCA--------------------

--------------------------------------------------

--------------------------------------------------

--------------------------------------------------

--------------------------------------------------

--------------------------------------------------

-----GACCATTATCAGAGTCACTG-------GTATTA-----GGAGAGA

AAAGTAGCCACAGGGGAGA---CTTCATTACCATTTGT---CTACATA--

--------------------------------------------------

--------------------------------------------------

--------------------------------------------------

--------------------------------------------------

--------------------GACTGTTGCTCTATGAGATACCAAGAGGAT

CACGTGAGTGTGGTTTGTCTT-ACAGGGACAATTACACTCTG

>rabbit

TAAGTTCCTTTTCAACCACTTAC----------ACACAAGGGAGACA---

-TCACTGTGTACAAAGTGGCAGTTAC---ACGATACGAGGGAGATGTGGG

TTC--TGGAATACCAGGGAGG-GGAGGCCGCAACTGATG---TTTTAGAA

GTGAC-----TTTTTTAAGCGGTTGTGGTAGCACATGCC---tcacataa

gac----ttcccactctg--------------------------------

--------------------------------------------------

--------------------------------------------------

--------------------------------------------------

--------------------------------------------------

--------------------------------------------------

-------------------------------------------accgtgg

gccgtgagcactgtgacgccagctacatgcacgctgctgtgcggtc----

----------------------accaccactgctccggaccgtttt--gt

cttctcagggtgaagctctgacttctgt------------------tccc

----------------tgcagtcgccgtccgttctcc---tttctgtctt

tgcaaatccagctaccccacttccctcacgtgagcgg-------------

--------------------------------------------------

--------------------------------------------------

--------------------------------------------------

----------------------------------------------agtc

acgcagcgtgttgtccttggctccgacttattgtacttgccacgccgtc-

--------------------------------------------------

--------------------------------------------------

--------------------------------------------------

--------------------------------------------------

--------------------------------------------------

---------------------------tt-cagggtgcatccgtggcaca

gtc-gccttcctaa------------------------------------

--------------------------------------------------

--------------------------------------------------

--------------------------------------------------

--------------------------------------------------

--------------------------------------------------

--------------------------------------------------

--------------------------------------------------

--------------------------------------------------

--------------------------------------------------

--------------------------------------------------

--------------------------------------------------

--------------------------------------------------

--------------------------------------------------

-ggctaaagaatacccgca-acatca-gtgggtgtttgta-ttatttcct

ccttttgag---AGAATTTACTGTTTTTCA--------------------

--------------------------------------------------

--------------------------------------------------

--------------------------------------------------

--------------------------------------------------

--------------------------------------------------

-----AACCATTCAATCCAGTGGCCAAATGTGGTTCT--------GATCA

ACTCCAAAGGAGGAGGAGATCCATTTATACTGATTTGT---CAGCT----

--------------------------------------------------

--------------------------------------------------

--------------------------------------------------

--------------------------------------------------

--------------------TAAACATTTTGTCT-GTGTTACCAACAGAA

GTATTTCCGTGTGGTTCTCTCTGCAGGGACAATTACACCTTA

>rhesusmonkey

TAAGTACCTTTACAATGATTATA---------GGCAGAAAAAGAAAAA--

-TCACTGCTTTAAAAGTA--AGAGTC---AAATGATACAAGGAAAATATA

TGT-----CATGCCTGAAAAG-GGCAATTTTAACTAATGTTGCTTCAGAA

GTGAC---TTTTTTTAAA---GTTGTGGTGAAATACACA---ttacataa

aa----tttaccgtttta-----ggccaggcgcggtggctgttgcctgta

atcccagcactttgggaggccaaggcgggcggatcatgaggtcaggagat

cgagaccatcctggctaacacagtgaaaccccatctctactacaactaca

aaaaaaattagccgggcgtggtggtgggcacctgtagtcccagctatttg

ggaggctgaggcaggagaatggcatgaacctgggaggcagagcttgcagt

gagccgagatcacatcactgcactccagcctgggtgacagtgcgagactc

cgtctcaaaaaaaaaaaaaaa----TTACTGTTTTAacca-tttt---aa

atatacaatttagagtcattacatacattc-tattgttgtgc--------

--------------tgccatcaccaccatccaactctagaactttttcat

cttcccaaaatgaaactccgtacccagtaagcacttgctcc-ccattccc

----------------tggcagccaccattctgctttctgtccctgtaaa

tttgactcc-tctgggtacct---cacataagtggaa-------------

--------------------------------------------------

--------------------------------------------------

--------------------------------------------------

----------------------------------------------tcat

tcaatatttgtcttttt-gtgaccagctcatttcacttggcatgatgtc-

--------------------------------------------------

--------------------------------------------------

--------------------------------------------------

--------------------------------------------------

--------------------------------------------------

----------------cttcaggattcatccatgttgtagcatgtatcag

aatgtccttcctttttaaggctgaataatattccattatttttgca----

--------------------------------------------------

--------------------------------------------------

--------------------------------------------------

--------------------------------------------------

--------------------------------------------------

--------------------------------------------------

---------------------------tataatgctttgttta---ggcc

aggcgcggtggctcacgcctgtaatcctagcactttgggaggccgaggca

agtggatcacaaggccaggagttcaagacaagcctggccaacatagtgaa

atcctgtctctactaaaaatacaaaaattaaccaggcgtagtggtgggca

cctctaatcccagctacttgggaggctgagacagggaaatcacttgaacc

tgggaggcggaggttgcagtgagctgagaccatgtcgttgcactccagcc

tggggaacagagcaagactccatctc----gaaaacaaacaaacaaaa--

--ctttgtttatgcattcatctgccaag--gacatttggg-ttatttcca

ctttttgac---AGAAATTACTATTTTGAA--------------------

--------------------------------------------------

--------------------------------------------------

--------------------------------------------------

--------------------------------------------------

--------------------------------------------------

-----AACCATTAAATTCAGTGGAGGAATGTGGTTCTAACAGTAGTAAAC

AACTCTTAAAAGAGGAAGA-TTATTTATAATTATTTGT---CATAT----

--------------------------------------------------

--------------------------------------------------

--------------------------------------------------

--------------------------------------------------

--------------------TCAAGTGTTT------------CAAATGAA

GTATTTAAATGGGGTTGTCTTTACAGGGACAATTATACCCTA

>human

TAAGTACCTTTACAATGATTATCCCGGCGGCAGGCAGAAAAAGAAAAA--

-TCACTGTTTTAAAAGTA--AGAGTC------------------------

--------------------------------------------------

--------------------------------------------------

-----------------------ggccgggtgcagtggctcatgcctgta

atcccagcactttgggaggccgaggcgggcggatcacaaggtcaggatat

cgagaccatcctagctaacacggtgaaacccc--gtctctactaaaaata

caaaaacttacccaggcgtggtggcgggcgcctgtagtcccagctactca

ggaggctgaggcaggagaatggcgtgaacccggaaggcggagccgagatc

acgcc-----------actgcactccagcctgggcaacagagcaagactc

ctctaaaaaaaaaa----------GTTACCATTTTAgcca-tttt---aa

atatacaatttagagtcattacatacattcacattgttgtgc--------

--------------tgccatc-ccactatccaactctagaactttttcat

cttcccaaaatgaaaccctatacccagtaagcactcgctcc-ccattccc

----------------tggcagccaccattctgctttctgtccctatgaa

tttgactcc-tc-gggtacct---cacataagtggaa-------------

--------------------------------------------------

--------------------------------------------------

--------------------------------------------------

----------------------------------------------tcat

tcaatatttgtcttttt-gtgaccagctcatttcacttaacatgatgtc-

--------------------------------------------------

--------------------------------------------------

--------------------------------------------------

--------------------------------------------------

--------------------------------------------------

----------------cttcagggttcatccatgttgtagtatgtatcag

aatgtccttcctttttaaggcagaataatattccattatttttgca----

--------------------------------------------------

--------------------------------------------------

--------------------------------------------------

--------------------------------------------------

--------------------------------------------------

--------------------------------------------------

---------------------------tataatgctttgttta---ggct

gggcgtggtggctcatgcctgtaatcccaccactttgggaggccaaggct

ggtggatcacaatgccaggagttcaagaccagcctgaccaacatagtgaa

accctgtctctactaaaaatacaaaaattaaccaggcatggtggcgggcg

cctgtaattccagctacttgggaggctgagacaggagaatcacttgaacc

tgggaggcggaggttgcagtgagctgtgaccgtgtcattgcactccagcc

tggg--cgagagcaagactccatctcaaaaacaaacgaacaaacaaaa--

--ctttgtttatacattcatctgccaaag-gacatttggg-ttatttcca

ctttttgac---AGAAATTACTATTTTTAA--------------------

--------------------------------------------------

--------------------------------------------------

--------------------------------------------------

--------------------------------------------------

--------------------------------------------------

-----AACCATTAAATCCAGTGGAGGAATGTGGTTCTAACAGTGGTAAAC

AACTCTTAAAGGAGGAAGA-TTATTTGTAATTATTTGT---CATAT----

--------------------------------------------------

--------------------------------------------------

--------------------------------------------------

--------------------------------------------------

--------------------TAAAGTTTTT------------CAAATGAA

GTATTTAAATGGGGTTGTCTTTACAGGGACAATTATACCCTA

>chimpanzee

TAAGTACCTTTACAATGATTATC---------GGCAGAAAAAGAAAAA--

-TCACTGTTTTAAAAGTA--AGAGTC------------------------

--------------------------------------------------

--------------------------------------------------

-----------------------ggccgggcgcagtggctcatgcctgta

atcccagcactttgggaggccgaggcgggcagatcacaaggtcaggagat

cgagaccatcctagctaacacggtgaaacccc--gtctctactaaaaata

caaaaacttacccaggtgtggtggtgggtgcctgtagtcccagctgctcg

ggaggctgaggcaggagaatggcgtgaacccggaaggcggagctgagatc

acgcc-----------actgcactccagcctgggcaacagagcgagactc

ctctaaaaaaaaaa----------ATTACCATTTTAgccg-tttt---aa

atatacaatttagagtcattacatacattcacattgttgtgc--------

--------------tgccatcacccctatccaactctagaactttttcat

cttcccaaaatgaaaccctatacccagtaagcactcgctcc-ccattccc

----------------tggcagccaccattctgctttctgtccctatgaa

tttgactcc-tctgggtacct---cacataagtggaa-------------

--------------------------------------------------

--------------------------------------------------

--------------------------------------------------

----------------------------------------------tcat

tcaatatttgtcttttt-gtgaccagctcatttcacttaacatgatgtc-

--------------------------------------------------

--------------------------------------------------

--------------------------------------------------

--------------------------------------------------

--------------------------------------------------

----------------cttcagggttcatccatgttgtagcatgtatcag

aatgtccttcctttttaaggcagaataatattccattatttttgca----

--------------------------------------------------

--------------------------------------------------

--------------------------------------------------

--------------------------------------------------

--------------------------------------------------

--------------------------------------------------

---------------------------tataatgctttgttta---ggct

gggcgtggtggctcatgcctgtaatcccaccactttgggaggccgaggca

ggtggatcacaatgccaggagttcaagaccagcctgaccaacatagtgaa

accctgtctctactaaaaatacaaaaattaaccaggcatggtggcgggca

cctgtaatcccagctacttgggaggctgagacaggagaatcacttgaacc

tgggaggcggaggttgcagtgagctgagaccgtgtcattgcactccagcc

tggg--cgagagcaagactccatctcaaaaacaaacgaacaaacaaaa--

--ctttgtttatacattcatctgccaaag-gacatttggg-ttatttcca

ctttttgac---AGAAATTACTATTTTTTA--------------------

--------------------------------------------------

--------------------------------------------------

--------------------------------------------------

--------------------------------------------------

--------------------------------------------------

-----AACCATTAAATCCAGTGGAGGAATGTGGTTCTAACAGTGGTAAAC

AACTCGTAAAGGAGGAAGA-TTATTTGTAATTATTTGT---CATAT----

--------------------------------------------------

--------------------------------------------------

--------------------------------------------------

--------------------------------------------------

--------------------TAAAGTTTTT------------CAAATGAA

GTATTTAAATGGGGTTGTCTTTACAGGGACAATTATACCCTA

>elephant

TAAGTACCTTTACAGCGACCATG---------TACAGAAAAGTATC----

-TCACTGTTCTGAAAGTAGGAGAGTC-GCATGATACAGGGAAAATTTATA

TTACATGTTG--CCAGAACAG-GGAGACGATAACTAATGT---TTCAGAA

ATGAC---------------------------------------------

--------------------------------------------------

--------------------------------------------------

--------------------------------------------------

--------------------------------------------------

--------------------------------------------------

--------------------------------------------------

--------------------------------------------------

--------------------------------------------------

--------------------------------------------------

--------------------------------------------------

--------------------------------------------------

--------------------------------------------------

--------------------------------------------------

--------------------------------------------------

--------------------------------------------------

--------------------------------------------------

--------------------------------------------------

--------------------------------------------------

--------------------------------------------------

--------------------------------------------------

--------------------------------------------------

--------------------------------------------------

--------------------------------------------------

--------------------------------------------------

--------------------------------------------------

--------------------------------------------------

--------------------------------------------------

--------------------------------------------------

--------------------------------------------------

--------------------------------------------------

--------------------------------------------------

--------------------------------------------------

--------------------------------------------------

--------------------------------------------------

--------------------------------------------------

--------------------------------------------------

--------------------------------------------------

--------------------------------------------------

----------------------TGTTCCAA--------------------

--------------------------------------------------

--------------------------------------------------

--------------------------------------------------

----------------------------------gaaaaccatatggggc

agttctactctgccacgtgaggtcgttaggagtcagcaatgactcgatgg

ta----CCCAACAACAACGAATGTG-------GTTCCAGTAGAGGTGAAC

AATTCTAAAAAGCGGGAGA-TAATTTACAATTATTTGT---CATATT---

--------------------------------------------------

--------------------------------------------------

--------------------------------------------------

--------------------------------------------------

--------------------AAAATGT-TTATATGAAATGTGGAGAGGAG

TATTTAAGTGTGGTTT-TCTTTGCAGGGATAACTATACCCTA

>armadillo

TAAGTGCCTTTACAATGACTATA---------TACAGAAAAGAAAA----

-GCACTCTTTTGAAAGTAATGGAGTT-GCATGATACAAGGAAAATGTATA

TTACAATATG--CCAGGAAAG-GGATACTACATCTAATGCTGATTC----

--------------------------------------------------

--------------------------------------------------

--------------------------------------------------

--------------------------------------------------

--------------------------------------------------

--------------------------------------------------

--------------------------------------------------

--------------------------------------------------

--------------------------------------------------

--------------------------------------------------

--------------------------------------------------

--------------------------------------------------

--------------------------------------------------

--------------------------------------------------

--------------------------------------------------

--------------------------------------------------

--------------------------------------------------

--------------------------------------------------

--------------------------------------------------

--------------------------------------------------

--------------------------------------------------

--------------------------------------------------

--------------------------------------------------

--------------------------------------------------

--------------------------------------------------

--------------------------------------------------

--------------------------------------------------

--------------------------------------------------

--------------------------------------------------

--------------------------------------------------

--------------------------------------------------

--------------------------------------------------

--------------------------------------------------

--------------------------------------------------

--------------------------------------------------

--------------------------------------------------

--------------------------------------------------

--------------------------------------------------

--------------------------------------------------

------------AGAAATTACTGTTTTTAA--------------------

--------------------------------------------------

--------------------------------------------------

--------------------------------------------------

--------------------------------------------------

--------------------------------------------------

-----AACCATTAAGTCTGACAATCTAAT---GTGGTTGTA-ACAGAGAT

AACTCTAAAAAGAGGGATA-TAATTTATA----ATTGT---CATGTA---

--------------------------------------------------

--------------------------------------------------

--------------------------------------------------

--------------------------------------------------

--------------------AAAAT-T-TTATACGAAATATAGAGAA---

CATTCAAATGTGGTTT-TCTTTACAGAGATAATTATACCCTA

Marker 3k:

>L1MB3

--------------------------------------------------

--------------------------------------------------

--------------------------------------------------

--------------------------------------------------

--------------------------------------------------

--------------------------------------------------

--------------------------------------------------

--------------------------------------------------

--------------------------------------------------

--------------------------------------------------

--------------------------------------------------

--------------------------------------------------

--------------------------------------------------

--------------------------------------------------

--------------------------------------------------

--------------------------------------------------

--------------------------------------------------

--------------------------------------------------

--------------------------------------------------

--------------------------------------------------

--------------------------------------------------

--------------------------------------------------

--------------------------------------------------

--------------------------------------------------

--------------------------------------------------

--------------------------------------------------

--------------------------------------------------

-----------------------------------------tggttgcac

aacaatgtgaat----gtacttaatgccactgaactgta--cact-taaa

aatgg-tt-aaaatgg------taaa---ttttatgttatgtata-tttt

accacaattaaaaaaa----------------------------------

--------------------------------------------------

--------------------------------------------------

--------------------------------------------------

--------------------------------------------------

--------------------------------------------------

--------------------------------------------------

--------------------------------------------------

----------------------------------

>cow

CATGGATCTGCAAAGGTAACATAACGGCATATG-AACACACACCTCTGTG

ACACT-----AACC-TCTGTCACAGTCCAAAAAGGAGTTATGAAAAAC--

ACATAAAATGTGATGTCAATAGAAAATCAAAAGGTAGAAAATAC------

-AAA-CACAATTTT-ATTCATAGAGAAAA--------TAAACAGGCTACC

ATTATCACATCT--------------------------------------

--------------------------------------------------

--------------------------------------------------

--------------------------------------------------

--------------------------------------------------

--------------------------------------------------

--------------------------------------------------

--------------------------------------------------

-----------------------------TAAGAGGCTATCTAAAATCTT

GTTGTGATTATTCTTTTTCACTTCTCTTCCTGACCCTGTTATTGCCTTTC

--ACCGAACAAAAGG-----------------------------------

--------------------------------------------------

--------------------------------------------------

--------------------------------------------------

--------------------------------------------------

--------------------------------------------------

--------------------------------------------------

--------------------------------------------------

--------------------------------------------------

--------------------------------------------------

--------------------------------------------------

--------------------------------------------------

------CAAATCAACTGGTTCCCAAAT------CACTTGGTTATATAGAA

AATACATAACATGCAATATTTTCC----AGAAGTTTCC---tgagagcac

aacaatgtgaatctacttaatg----tcactgaactgta--tac------

------ttagaaatgg-----ttaaagcagtaaattttgtgttttt----

-cacaaattattaaaaaaagacac---ACACAGTTTCCTGG---------

--------------------------------------------------

--------------------------------------------------

--------------------------------------------------

--------------------------------------------------

--------------------------------------------------

--------------------------------------------------

--------TGAT-TCTA-CCCA----GCTGATTAGTCC-TCATTAGTTAA

CATATGGAAGGGGGCACTCTACAT----AAAGCA

>dog

CATGGATCTGCAAAGTTGTTGAC-----------AAAATGACATGAATAA

ACCAG-----GACACT-GACCAATACCCAAAAGGGAGTTCTGAAAAAC--

CCAGAGAATGTGATGTCAG-AGAAAATAAAAAGCTAGAAAATGCTTT---

-AAAGCCACATTCTTATTCATATATG-------------AATAGAGTACC

AATGTCACATCC--------------------------------------

--------------------------------------------------

--------------------------------------------------

--------------------------------------------------

--------------------------------------------------

--------------------------------------------------

--------------------------------------------------

--------------------------------------------------

------------------------------AACAGGCAATCTACAACCTT

GTCTT-CATTATTCTTTTAACTTTTCTTCCTGACCTTGTCATTGCTTTTC

TTTCCAACCCAAA-TAAATTAACCCAG----GAAAACACCGAGAACTTAA

AATACAAATTATTCCCGTA-------TAATTTTT-----CTCTTAAACAC

AGTGATTTC---gggacgcctgggtggcttctgcctttggctcagagcgt

gatcccggagtcctggaatcaagtcccacattgggcttcctgcagggagc

ctgcatctccctctgcctacgtctatgtctctgtctctcatgagtaaata

aataaaatattaaaaaCACACATACACACACAAAACAA------------

--------------------------------------------------

--------------------------------------------------

--------------------------------------------------

--------------------------------------------------

--------------------------------------------------

--------------------------------------------------

------AACTGGTTT----TTAGGGAAAAGTATGTATTTGGTTACATAGA

AAACATATAATAT-----TTTCCC----ATAAGTTTCC---tggtagcac

aaca---tgaatgtacttaatg------------ct-ta--tac------

-----ttaaaaaatga-----ttaaaataataaattttatgt--------

-----------------------------ACAGTTTCCCAGT--------

--------------------------------------------------

--------------------------------------------------

--------------------------------------------------

--------------------------------------------------

--------------------------------------------------

--------------------------------------------------

--------TAAT-TCTA-CCCACTTGACTGGCTAGTGACTTGCTAGTTCA

CA--CAAAAGGAGGCATTCTACAC----AAACTA

>pangolin

CATGGATCTACAAAGTTGTTGACATGACGACATATTAACAAA---CTGTG

CCACTAACAAATATCTCTATCACAACCCCAGAGGAA-TTCTGAAAAACAC

ACAACCAATGTGATGTCAT-AGAAAATCAAAAGCTAGAAAATGCTTT---

-AAAGTTACAATTTTACTCATAAA--------GCAAATGAATAAAGTACC

ATTATTACATCT--------------------------------------

--------------------------------------------------

--------------------------------------------------

--------------------------------------------------

--------------------------------------------------

--------------------------------------------------

--------------------------------------------------

--------------------------------------------------

-----------------------------CAACAGGCTATCTAAACTCTT

GCCTTGATTATTTTTTT-CATTACTCTTCCTAACCCTATCATTGCCTTTC

T-ATCAAGCAAAA-TGAACTATTGGAGGA--GAAATCGCTGGGAGCTTAT

AATT----CCTGTACCATACT-----------------TCTCTCA-----

--------------------------------------------------

--------------------------------------------------

--------------------------------------------------

--------------------------------------------------

--------------------------------------------------

--------------------------------------------------

--------------------------------------------------

--------------------------------------------------

--------------------------------------------------

--------------------------------------------------

------AAACAGTTTTTAGGTTAAAGTGTG---TATCTGGTTATACAGAA

AATATGTAACACatAGTATTTTTC---AGAAGTTGTC----tggcagcac

aag----tgaatatgcttaatg---ccaccaaagtgta---tac------

------tttaaaatgg-----ttaaaatgttatgtttcatgtaatggtaa

atccaccacaactaaaa----------TGTTATTTTCCTGGT--------

--------------------------------------------------

--------------------------------------------------

--------------------------------------------------

--------------------------------------------------

--------------------------------------------------

--------------------------------------------------

---------AAT-TCTA-CCCATTTGACTGGTTAGTCCCTTCCTACCTCA

CA--TATAGAGGGGCATTCTACAC----AAAGCA

>mouse

CGTGGATCTAAAAAGTTGTTAATATT------------------------

--------------TTAAAAACTACTCAAAACACAG-TCCTTAG-AACAC

A-----GTATATACCACTATAGAAAATCAAAATGTA------GCTGTACT

TAGTGGCACAGGCTTATAGTTTAA-----CTAGGTGATAAAAGCAGAAAG

ATAGCAAGTTT-GAGTCTAACCTGGGTAACTTAA---GACACAATCTCAA

AAACAAAAACTAGGAAGATACAAACAGAAGAGGGCTGGGCATATAGCTCA

AAGGCTGTGTTCAATTCCTATTACTG------------------------

-------------AAAACACACACACACACACAAACAGCCACCAAAACAA

GAACAACCATTTAAAAATGAAAAAGGAAAAACCTAGAACATGCTTAAAAT

CGAAATTCTTTTGTTTCTTTTCATTTCTG-tttttttgttttgaggccga

atctctctatatattactgtctggcctggagcttgctatgtagaccaggc

tggcctcaaagtcagaaaattctcctgcctcagcctcccaagtgctggga

ttaaataaatctcctactacatccagct-TAATCATAAAACAAGGCTATG

GTCTTCATTT----GCATCATTCATCTTCTTGATCCAGTCTATTTACC--

-----AAG-AAAATTAAATTCACTATACA--GAAAACATTGTGAGCTTAT

AATATAAACAATTCCCATAATACCCACA----------CCCCTT------

--------------------------------------------------

--------------------------------------------------

--------------------------------------------------

--------------------------------------------------

--------------------------------------------------

--------------------------------------------------

--------------------------------------------------

--------------------------------------------------

--------------------------------------------------

--------------------------------------------------

------TGTGACTTTAAAAAAAAAAAAAAG---CTACCCTTATTACGGAA

A-TATGT------TCACATTTCTC-----AAAACTTC-------------

-aca--gggaagataa-taatgtgaatatgcttaatgtcagccactgaac

tagatacttaaaatgggttagttaagatggccaattttatatgtagtttt

atcatatttaaaacaa--------------GAG-TTGGAGG----aggtt

ggggagatggttcagtgggttaaacacttgccacgcaagtgtaagaaatg

acattcagacttctagagtccacataaaagctagtgggactggtgccctg

ccaatgaccccaggagtacccaggaggtggagagagaatatgtggaacag

cccgctcaagccaaagtagc-agtactagctcaccaaatgggaagataaa

tcctggAAGACACTCCAAAGTCAGCCTCCGACTTCTCTTATAGA--ACCA

CATGTATGCGTGTG--------------CCAAGCAACAATAACTAAACTT

CACAGA--TCAT-TCTG-CTCACT----------GTCCCTTGCTAGTCCA

AAAGTAGAAAGGGGCCTTTTATAC----AAAGCA

>rat

CGTGGATCTAAGAAGTTGTTAATAATATTTTAAAAACTATAAACTTAAAA

AAAAAAA-------TA-GAGTTTTTACCCAGTTTGAGTTCTGAA-AACAC

A-----GTATGTGCTATTATAGAAAATTAAAATGTA------GCTGTGCC

TAGTGGTATAGTATAGGCTTGTAATTTAACTAGGTGGTTAAAGCAGAAAG

ATGGCAAGTTTTAAATCTAACCTGGGTAACTTAACAAGACACAATCTCAA

AAA-ACAAGCTGGAGAAAAAGAAACAGGTCTGGGCATATAGATCAACACT

TGTCTGGCATTCAAGGGTTGTGTTCAATTCCTATTACTGAAacacacacg

cacgcacgcacacgcacacacacCCCAACCACCAAAACAATAACAACCAT

AAAAAACAAAAACCTGTTTAAAATCATGATCATTAGAACATGCTTAACGT

CAAAATTCTTTTGTTTC-TTTAGTTTCTG-tttttttgttttgaggtaga

atctctgtttctgtctgtcctgaatctgccctggcctcctgagcgctgga

acaggatgcatcggctaccacacccagct---------------------

-----------------------------TAACCACAGAACAAGGCTATG

GTCTTCACTTGAA-TCACTTACCTTCTTCTTGATCCAGTCTTTCTATC--

-----AAG-AAAACTGAACTCACTACACA--GAAAACACTGTGAGCTTAT

AATATAAACAATTCCCACAATACCCACA----------CCATTT------

--------------------------------------------------

--------------------------------------------------

--------------------------------------------------

--------------------------------------------------

--------------------------------------------------

--------------------------------------------------

--------------------------------------------------

--------------------------------------------------

--------------------------------------------------

--------------------------------------------------

------TGAGAGTT-----AAAAAAAAAA-------TCCTTATTATTGAA

AATATGT------AACATTTTTTC-----AAGACTTC-------------

-aca--gggaagataa-taatgtaaaaaacttaa-tgtcagccactgaac

tagatatttaaaatgg-----gtaagatggccaattttgtatacagtttt

atcatatttaaaacaa--------------AAG-TCAGAGG----aagtt

ggggagatggttcagtgggctaaacacttgtcatgcaagtg--aggaatg

gcactgtgacttgtagaagccaca--caagctggtggg--acgtg-cctg

ccaaggaccccaggagcatccaggaggtggagacagaatatgtggaacag

-cctcgc-agccaggctagc-agtaatagctcagcaaatgtgagagtgaa

-ccctgACGACAC-CCAAAGTCAACCTCAGACTTCTCGTATGTGACTCCA

CATGTATGTGTGTGCCAAGCACGCACCTCCAAGCAACAATAACTAAACTT

TATAGA--TCGT-TCTA-CTCACT----------GTCC-TTGCTAGTCCA

AATGCAGAAAGGGGCCTTTCACAG----GAAGCA

>human

CATGGATCTACAAAGTTGTGAATAATAT-----ATTAACAAACTGTGACA

CTAAC-----AAATAC-CTGTCACAACCCAAACGAAGTTCTGAAAAAC--

----AGAATGTGATGTCAT-AAAAAATCGAAACCTAGAACATACTTT---

-AAAACCACGATTTTATTCATAAAGGTATAAA-----TGAATGGAATACC

ATTATTGCATCT--------------------------------------

--------------------------------------------------

--------------------------------------------------

--------------------------------------------------

--------------------------------------------------

--------------------------------------------------

--------------------------------------------------

--------------------------------------------------

-----------------------------CAACAGATTATCTAAAATCTT

TTATTCATTTTACCC----CCTTCTCTTCCTGATCCTGTCACTGCTTTTC

T-ACCAAGATAAAATGAACTCATCCTACA--GAAAA-ACTGTCAGCTTAT

AATACAAACAATCCCCATATCACAATAGGCTTTT----CCTCTTAAA---

--------------------------------------------------

--------------------------------------------------

--------------------------------------------------

-------------------------------AAAAGTAGTTCTggccggg

cacagtggctcacacctgtaatcccagcactgtgggaggccaaggcaagc

agatcacttgaggtcaggagtttgagaccagcctggccaacatggtgaaa

ccccgtctctactaaaaataaaaaaattagccaggtgtggtggcgtgtgc

ccgtgatcccagcctctcggctgaggcaggagaattgcttgaaactggga

ggtggaggttgcagtgagctgagatcacaccactgcactccagcctgggc

aaaagagtgagactctgtctcaaaaaaagcaaataaataaaaataaataa

aaa---CAGTAGTTTTTA---GGGGAAAATATGTATTTGGTTATATAGAA

AATATTTAACACATAATACTTTCC----AAAAGTTTCC---tggtagcac

aacactgtgaatatacttaatg----ccactgaactgta--ca-------

------ttaaaaatgg-----ttaagataatcaattttgtgtgta-tctt

atcacaattaaaaa-------------AAAAAGTTTCCTGG---------

--------------------------------------------------

--------------------------------------------------

--------------------------------------------------

--------------------------------------------------

--------------------------------------------------

--------------------------------------------------

--------TCAT-TCTACCTCACTTGACTGGCTGGTCCCTTGCTAGTCCA

CATATGGAAGGAGGCATTCTATAG----AAAGCA

>chimpanzee

CATGGATCTACAAAGTTGTGAATAATAT-----ATTAACAAACTGTGACA

CTAAC-----AAATAC-CTGTCACAACCCAAACGAAGTTCTGAAAAAC--

----AGAATGTGATGTCAT-AAAAAATCGAAACCTAGAACATACTTT---

-AAAATCACGATTTTATTCATAAAGGTATAAA-----TGAATGGAATACC

ATTATTGCATCT--------------------------------------

--------------------------------------------------

--------------------------------------------------

--------------------------------------------------

--------------------------------------------------

--------------------------------------------------

--------------------------------------------------

--------------------------------------------------

-----------------------------CAACAGATTATCTAAAATCTT

TTATTCATTTTACCC----CCTTCTCTTCCTGATCCTGTCACTGCTTTTC

T-ACCAAGATAAAATGAATTCATCCTACA--GAAAA-ACTGTCAGCTTAT

AATACAAACAATCCCCATATCACAATAGGCTTTT----CCTCTTAAA---

--------------------------------------------------

--------------------------------------------------

--------------------------------------------------

-------------------------------AAAAGTAGTTCTggccggg

cacagtggctcacacctgtaatcccagcactgtgggaggccaaggcaagc

agatcacttgaggtcaggagtttgagaccagcctggccaacatggtgaaa

ccccgtctctactaaaaataaaaaaattagccaggtgtggtggcgtgtgc

ccgtgatcccagcctctcggctgaggcaggagaattgcttgaaactggga

ggtggaggttgcagtgagctgagatcacaccactgcactccagcctgggc

aaaagagtgagactctgtctcaaaaaaagcaaataaataaaaataaataa

aaa---CAGTAGTTTTTA---GAGGAAAATATGTATTTGGTTATATAGAA

AATATTTAACACATAATACTTTCC----AAAAGTTTCC---tggtagcac

aacactgtgaatatacttaatg----ccactaaactgta--ca-------

------ttaaaaatgg-----ttaagataatcaattttgtgtgta-tctt

atcacaatttaaaaa-------------AAAAGTTTCCTGG---------

--------------------------------------------------

--------------------------------------------------

--------------------------------------------------

--------------------------------------------------

--------------------------------------------------

--------------------------------------------------

--------TCAT-TCTATCTCACTTGACTGGCTGGTCCCTTGCTAGTCCA

CATATGGAAGGAGGCATTCTATAG----AAAGCA

>elephant

--------------------------------------------------

--------------------------------------------------

--------------------------------------------------

-------------------------------------TTATCATATCTCA

ACAAGCTATCCT--------------------------------------

--------------------------------------------------

--------------------------------------------------

--------------------------------------------------

--------------------------------------------------

--------------------------------------------------

--------------------------------------------------

--------------------------------------------------

-----------------------------GAAC-TCTTGCTGTG--ACTA

TTCTTTTTTTTCCCC----ACTTCTCATCTTGAATCTGTCATTGCCTTTC

T-ACAAGC-AAAATGAATTAAAAGTCAGGAGGAAAACACTGGGAGTTTAC

ATTAC-AACAATTCCCGTACCATCCCCATTTTTTTTTCTCTCCTAAAA--

--------------------------------------------------

--------------------------------------------------

--------------------------------------------------

--------------------------------------------------

--------------------------------------------------

--------------------------------------------------

--------------------------------------------------

--------------------------------------------------

--------------------------------------------------

--------------------------------------------------

------CAGCCGTTTCTATCTAGGGAAAAGTGTGATATTTGGT-------

-----ATAACACATAATACTTGCC--------------------------

--------------------------------------------------

--------------------------------------------------

----------------------------AAAAGTTTCCAGG---------

--------------------------------------------------

--------------------------------------------------

--------------------------------------------------

--------------------------------------------------

--------------------------------------------------

--------------------------------------------------

--------TAAT-TCTG-CCCATGTGACTGGCTAGTTCCCTACTAGTTCA

CATATGAAGTGGGACTTACTTACTACACAAAGCA

>armadillo

--------------------------------------------------

--------------------------------------------------

--------------------------------------------------

-------------------------------------GCAAGCAGAATAG

CATTTCACATCT--------------------------------------

--------------------------------------------------

--------------------------------------------------

--------------------------------------------------

--------------------------------------------------

--------------------------------------------------

--------------------------------------------------

--------------------------------------------------

-----------------------------CAACAGGCTACCTAAAACCTT

GCCTT-GATTACTCTTTTTTTTTTTCACTTCTCTTCCTAACCCTTTCT--

--ACCAAG-AAAAATGGACTAACCCAGT-AGGAAAACACTGAGAGCTTAC

AAAAAA--CAACTCCCATGCCATAATAAATATTT---CTCTTTTAAAA--

--------------------------------------------------

--------------------------------------------------

--------------------------------------------------

--------------------------------------------------

--------------------------------------------------

--------------------------------------------------

--------------------------------------------------

--------------------------------------------------

--------------------------------------------------

--------------------------------------------------

------CATTATTTT----CTAGGGAAAAGTGTGGTATTTGGTTATATAA

AAAAGATTAACACAAAATACCTG---------------------------

--------------------------------------------------

--------------------------------------------------

----------------------------ACAAGTTTTAAC----------

--------------------------------------------------

--------------------------------------------------

--------------------------------------------------

--------------------------------------------------

--------------------------------------------------

--------------------------------------------------

---------TATGCCTAAGTGACT-ACTTGGACAGTCCTTTGCTAGTCCA

TATATGAATGAGGGCATTCTATAC----AAAGCA

Marker 4a:

>MLT1A0

-----------------------------------------------tgc

tatggtctgaatgtttgtg---------tccccccaaaattcatatgttg

aaa--cctaatccccaa--tgtgatggta-ttag-gaggtggggcctttg

ggaggtgattaggtcatgagggcggagccctcatgaatgggattagtgcc

cttataaaagagaccycagagagctcccttgccccttccgccatgtgagg

acacag---------------------------tgagaaggcgccgtcta

tgaaccaggaaatgggccctcaccagacactgaatctgccggcgccttga

tcttggacttc------ccagcctccagaactgtgagaaataaatttctg

ttgtttataagctacccagtctatggtattttgttatagcagcccgaaca

gactaagaca----------------------------------------

--------------------------------------------------

---

>cow

CATTGTAAGTCAAGAAGGGATTTTTAACATCAAAC---CTTCAG---tgt

tacaaactgaatgcctgtgtccctttcacactt--cccatttca--gttg

aaa--tcttaaccccaac--gtgatggtagctggagagg-----------

-ttctgctgtcatgaatggacttagtgttcttagacaaagagatggggca

gag-----------cttgcttcctgtctctctgctctccaccaagggaga

agacaa---------------------------caagaat-----tcagc

catctgcaaaccaggaagctggctctcaccacatgccagatctgctggca

ccttgattgtggacttcccagcccccagcactgtgagaaataagtgtttg

ttg--tttaagccacccagtct--ggcaatttgttttaacagcctgaa--

--ctaagaca---CTAAGA---CAATAGGAGTC-AAAAAAA---------

-----TAA---GACAACAGTATCAGCCCAGTTGCTTAGAGGTGGTAAATT

AAT

>dog

CTTTGTAAGTCCAGAGATGATTT-CAGCATCAAA----CTAAAG---tgt

tatggactgaatcttcgtgtccctgccctcctcaccccatgcgcatgttg

aaa--tcctaactcccga-tgtgatagta-ctgg-gaggtaaggtgagag

gttccaccctcatgaatgggaagagtgtccttataaggagagatgggaca

c-----------agcttgctt-ctctctctctgctctctgccaggtgagg

atacag---------------------------tgaaaaga----cagcc

catcaacaaaccgggaagtgggtcctcaccagacactagatctgccaacg

ccttgatcttggacttcccagcctccagaactgtgagaagaaaatgtca-

-----tttaagcacc-aggtctgcagtaatttcttatagtaggctgaact

gacaaagaca---CTAAGA---CAGTAGGAATCTGAAAAA----------

-----TAAGAAGACAACAATACTGGCCCAGTTGCTTAGAGGTGATAAATT

TAT

>bat2

CCTTGTAAATCAAGGGAAGATTT-CAACATCAAGC---CTAAG----tgt

taaggactgagtgtttgtttgtg-ctaacccccacccctttcatgtgttg

ata--tcctaacctcccaatgtgatggtattgggaggtaa-tttgagagg

gt-cctccctcatgaatgggattagtgcccttataagaagagatgagaca

gag-----------cttgctt-c-ctctctctgcactccactgtgtgaga

atacaa--gaaggacgccatctgcaacc-----caggaagc----tgcct

ctcaccaggcaccagacc------------------------tgctggcc

ccttgatcttggacttcccagcctccaacaccgtgagcaat-aatgtttg

ttg--tttaagccgcccagtctatgatactttgttatagcagcctgaact

gactaagaaa---CTAAGA---CAATAGAAGTCTAAAAAA----------

-----TAAGA---CAACGATATTGGCCCTGTTGCTTAGAGGTGATAAATT

TAT

>shrew

CA---AAAGTGAAGAGGGGATTT-CAACATCAGGG---CTTATG---cgc

tgtggactgaatatttatt----------catctgcccagttcaaatgtg

aaatatcctaa-tgcctt-tgtgatggta-cagg-gag------------

--------ttagtgaatggggttagtgctcttgtcaaggaggagaggtag

gac-----------tgaacttgcttccctactctctgctgtccacagaag

ataccaggaacatgtgagtgaacaacacaaggatacaaagagaagacaac

catctgcacaccagaaaactggctctcaccagacaccagatctgctggca

ccttgatcttggactttccaggcttcacaaccgtgagaaatcaatgtttg

ttg--tttaagccacc--ttctatggtaatttgttagagcagccggaact

gactaagata---CCAAGA---CAATAGGAGTCTAGAAAA----------

-----TAAGAAGACAACAATATTGGCCCAGTTGCTTAGACGTGATAAATT

TAT

>mouse

CACTGGGAGTCAACAGATGGCTT-CAGCATCAAGC---------------

--------------------------------------------------

--------------------------------------------------

--------------------------------------------------

--------------------------------------------------

--------------------------------------------------

--------------------------------------------------

--------------------------------------------------

--------------------------------------------------

-------------CCAGGA---CAATAGGATCTAAAAA------------

------------TTAAGACTATTGGCCTAGTTCCTCAGAGGTGGCAAATG

TAT

>rat

CACTGGGAGTCAACAGTCGGCTT-AGACATCAAGC---------------

--------------------------------------------------

--------------------------------------------------

--------------------------------------------------

--------------------------------------------------

--------------------------------------------------

--------------------------------------------------

--------------------------------------------------

--------------------------------------------------

-------------CCAAGA---CAATAGGCTCTAAAAA------------

------------GTAGGAATATTGGCCCAGTGGCTCAGAGGTGGCAAATG

TAT

>rabbit

CA-TGTAAGTC-ACAGAG-ATTT-CAACATCAAGT---------------

--------------------------------------------------

--------------------------------------------------

--------------------------------------------------

--------------------------------------------------

--------------------------------------------------

--------------------------------------------------

--------------------------------------------------

--------------------------------------------------

-------------CTCAGA---CATGGGAACGCAAAAAA-----------

-----TAAGGAGACAATCATATGGGCCCAGTTGCTTCGCAGTGATAAGTT

GAT

>rhesusmonkey

CATTATAAGTTAACAGAGGGTTT-CAACATCAAGC---------------

--------------------------------------------------

--------------------------------------------------

--------------------------------------------------

--------------------------------------------------

--------------------------------------------------

--------------------------------------------------

--------------------------------------------------

--------------------------------------------------

-------------CTAAGA---CAATAGGAATGAAAAGAAAAAAAAAAAA

AGAA-------GACAACAATATTGGTCCAATTGTTTAGAGGTAATAAATG

TAT

>human

CATTATAAGTTAACAGAGGGTTT-CAACATCAAGC---------------

--------------------------------------------------

--------------------------------------------------

--------------------------------------------------

--------------------------------------------------

--------------------------------------------------

--------------------------------------------------

--------------------------------------------------

--------------------------------------------------

-------------TTAAGA---CAATAGGTATCAAAAAAAAAAAAAAAAG

AAGAAGAAGA---CAACAATATTGGTCCAATTGCTTAGAGGTAATAAATT

TAT

>chimpanzee

CATTATAAGTTAACAGAGGGTTT-CAACATCAAGC---------------

--------------------------------------------------

--------------------------------------------------

--------------------------------------------------

--------------------------------------------------

--------------------------------------------------

--------------------------------------------------

--------------------------------------------------

--------------------------------------------------

-------------TTAAGA---CAATAcGTATCAAAAAAAAAAAAAAaag

AAGAAGAAGAAGACAACAATATTaGTCCAATTGCTTAGAGGTAATAAATT

TAT

>elephant

CATTGTAAGTCAACAGAGGATTT-CAATATCAAGG---------------

--------------------------------------------------

--------------------------------------------------

--------------------------------------------------

--------------------------------------------------

--------------------------------------------------

--------------------------------------------------

--------------------------------------------------

--------------------------------------------------

-------------CTAAGA---CAATAGAAGTCTAAAAAA----------

-----TGAGAAGACAACAATATTGGCCCAGTTGCTTAGAGGTGATAAATT

TAT

>armadillo

CG---TCAGTCTACAGAACATTT-CAACGTCAAGC---------------

--------------------------------------------------

--------------------------------------------------

--------------------------------------------------

--------------------------------------------------

--------------------------------------------------

--------------------------------------------------

--------------------------------------------------

--------------------------------------------------

-------------GTACGA---CAGTCGGAGCCTGAGAAA----------

-----TACACAGACAACAATGTTGGCTCAGTGGCTTAGAGGTGATAAATT

TAT

Marker 4b:

>L1MA9

--------------------------------------------------

--------------------------------------------------

-----------------------------------gtacaaagtttcagt

tatgcaggatgaataagttctggagatctaatgtacagcatggtgactat

agttaata-atactgtattgtatacttgaaatttgctaagagagtagatc

ttaagtgttct--------caccacacacaaaaaaatggtaactatgtga

ggtgatggatatgttaattagcttgattgtggt---aatcatttcaca--

----atgtatacgtatatcaaaacatcacgttgtacaccttaaatatata

caatttttatttgtcaattatacctcaataaagctggaaaaa--------

--------------------------------------------------

----------------

>cow

TAAAAACCC-TGTATGTA---TAGGATAGAGGCTTAAAGGTTAATGCCAG

TGTTCTTGGTGATACCTAGAGGTGGGC---ACTGTGTTTGCCTTGCCTTA

GAAATC-CGTTAAAA---AAAAAGCTTGTCCT---gtacaaaatttcaat

t----gtaagctctgaga----------------atctaata-tatagta

t-at-ctgtattgtgtacttgaaatttgccgagagtagatcttaagcatt

ca-----------------------cac----acacacacacacacacac

agtaatagatgtgttaattgttatcaactttgtgaaaatcatttcaca--

----gtgtgtatgtatatcaaatcaccatgttgtacacttcaaatatatt

acaattttatttgttaatcaaacctcaggaaagata-ggaatttttcatt

ttaatctg---TCCTAAATCGTTCCT----GCTCTTC----AAGTG----

--ATTTCCAGCCCTCA

>dog

TAAAACTTTGTGTATTTA---AGCATAAAATATTTAAAGATTAATGAGAA

GGTTATCAATTATACCTAGAGGTGGGCAATATTGTGTTTGCCTTGTCTTA

G-AAAC-CGTTTT-----AAAGGGCCTGTCCT---gtacaaattttcagt

t----ataagttttgaggctctaatgtatagtatgggg---actatagtt

a-atacgatattgtatacttgaaatgtgct--gagagtagatcctaaata

ttctcat--------------------------aaaaaaataactgtgtg

aagtgagggtatgttaattagcttaattatgtg---aatcatttcacaat

gtatatgtgtgtttatatataaaaatcatattgtacattttaaatatatt

aca-ttttatttgtcaatcatgcttcaataatactggggaagaagaaa--

-----------AAAAAGATCTGTCCTA---GGTTA-TCCCTACCTGTCAA

TTATTTCCAGCCCTTA

>bat6

TAAAATCCTGTGTATTTCA---GGATAGAATATTTAAAGATTGATGTCAA

GGTTCTCAGTGATACCTAGAGGTCAGC---ATTGTGTTTGTCTGGCCTT-

CAAAAC-CATTTT-----AGAAGAACTGTCCT---gtacaaacgttcagt

t-----tacgttctgaggacctaatgcacagcacggtg---actatagtt

aaccacaaggtattatatacttagatcttaagcattcacacacacacaca

cacacacacacagacacacacatacacacagacacgaagtgactcagtga

ggggatggatgtgttaattaacctgattgggggg---atcatttcacca-

---------tgtatatatcaaattatcctgttgtacactttaaagaggtc

acaatgttatttgtcaattagtcctcaatggaact---aggaataaaa--

-----------AATGAGATCTGTCCTG---GGTTG-TTCCTACTTGTCAA

TTATTTCCAGCCCTTA

>shrew

TAAAATCCTGAATTTTTAGATTAGACAAAAGATTTAAAGATTAATTCCAA

GGTTATCTCTGA----TAGAGGTGAGA---AATGTGTTTGCTTTCCCTTA

GAAACT--ACTTAA----AAATCACCTGGTTT---gaacaagttttctga

-----tttattgtgtaacctg-------------atgattgg----agtt

acattttgtactgtatatttcaaatgtgctaagagcaaatgttaagcatt

at-----------------------ctctttctctctcataactatgtaa

aatgatggatgtgttaattagcttgattatggg---actcatttcaca--

----atgtatatagatatcaaatcatcactttatgtattttaaatgtatt

ataattttgtttgtcaatccgacctccatacagctgggaaagaagaat--

-----------AAAAGGTCTGCCCTGG---TGATTTTACTTACCTTTTAA

TTATTTCCAGTTCTCA

>rabbit

TAATGCTCTGTGTGTCTG---AGCACAGAGGAGTTCAAGATCAATGTCAG

AGTTCTCTGTGATGGCTGGAGGTGGGC---ATTATGTTTACCTTGCTTT-

G-AAAACCACTTT-------------------------------------

--------------------------------------------------

--------------------------------------------------

--------------------------------------------------

--------------------------------------------------

--------------------------------------------------

--------------------------------------------------

-----------AAAAAGACCTACCCT----CTGGTATACCTGTTTGTCAA

TTATTTCCAGCCTTTA

>rhesusmonkey

TAATACCCTGTATATTTG---AGCATAGAAGAGTTATAGATTAATGTCAG

GGTTCTCACTGATGCCTAGAGTTAGGC---ATCACATTTGCCTTGCCTTG

G-AAAC-TACTT--------------------------------------

--------------------------------------------------

--------------------------------------------------

--------------------------------------------------

--------------------------------------------------

--------------------------------------------------

--------------------------------------------------

-----------AAAAAGACCTGCGCT----GGGTTGTTCCTATGTGTCAA

TTATTTCCAGCCTTTA

>human

TAACAACCTGTGTATTTG---AGCATAGAAGATTTATAGATTAATGTCAA

GGTTCTCAGTGATGCCTAGAGTTAGGC---ATTATGTTTGCCTTGCCTTG

G-AAAC-TACTT--------------------------------------

--------------------------------------------------

--------------------------------------------------

--------------------------------------------------

--------------------------------------------------

--------------------------------------------------

--------------------------------------------------

-----------AAAAAGACCTGAGCT----GGGTAGTTTCTATGTGTCTA

TTATTTCCAGCCTTTA

>chimpanzee

TAACAACCTGTGTATTTG---AGCATAGAAGATTTATAGATTAATGTCAA

GGTTCTCAGTGATGCCTAGAGTTAGGC---ATTATGTTTGCCTTGCCTTG

G-AAAC-TACTT--------------------------------------

--------------------------------------------------

--------------------------------------------------

--------------------------------------------------

--------------------------------------------------

--------------------------------------------------

--------------------------------------------------

-----------AAAAAGACCTGAGCT----GGGTAGTTTCTATGTGTCTA

TTATTTCCAGCCTTTA

>elephant

TAAAACCCTGTGTAACCG---AACATAGAGGACTTAAAGATTAATGTCAA

GGTTCTCAGTGATGCCTAGAGGTAGGC---ATTTTATTTGCCTTATCTTA

G-AAAC-CATTT--------------------------------------

--------------------------------------------------

--------------------------------------------------

--------------------------------------------------

--------------------------------------------------

--------------------------------------------------

--------------------------------------------------

-----------AAAAAGACTTGTCAT----GGGTTATCCTTACTGGTCGA

TTATTTTCTAGCCCTG

>armadillo

TAAAACCCTGTGTATTTG---AACATAGAGGAGTTAGAGATTAACGTCAA

GGTTCTTAATGATGTCTAGAGATGGGC---ACTGTGTTTGCTTTACCTTT

C-AAGC-CATGT--------------------------------------

--------------------------------------------------

--------------------------------------------------

--------------------------------------------------

--------------------------------------------------

--------------------------------------------------

--------------------------------------------------

-----------GAAAAGACCTGCCCT----AAGTTGTCCCTACTTGTTAT

TTATTTCCAGACATTA

Marker 4c:

>MLT1A0

--------------------------------------------------

--------------------------------------------------

----------------------------tgctatggtctgaatgtttgtg

t--cccccc---aaaattcatatgttgaaacctaatccccaatgtgatgg

ta-tta--ggaggtggggcctttgggaggtgattaggtcatgagggcgga

gccctcatgaatgggattagtgcccttataaaagagaccycagagagctc

ccttgccccttccgccatgtgaggacacagtgagaag-gcgccgtctatg

aaccaggaaatgggccctcaccagacactgaatctgccggcgccttgatc

ttggacttcccagcctccagaactgtgagaaataaatttctgttgttt-a

taagctacccagtctatggtattttgttatagcagcccgaacagacta--

--------------------------------------------------

--------------------------------------------------

--------------------------------------------------

--------------------------------------------------

--------------------------------------------------

---------------------------------------------

>dog

ATAGAAAATGCTTTCTCGAGGGAGGGAAAGAAAAATCCCAATA-GTATTT

TAAGATTAAGACAAC--TTTCTAAATCTCGAAGTA-TACATATTT----C

AGATAATGAGATA---ATCTGCAC----tgtgacggactcgatgtttgtg

tccccctcacctcaaattctt-ctttgaaacccattcttcaatttgttag

tattca---gaagtgggggctttgggaggtgataaggtcatggggggaga

agtctcatgaatagggttagtgtccttattaaagaagcctcagagatctc

ccttgtcccttctgccatgtaatgacagagcaagaagacggtagtctgtg

aaccaggaagcaggctcttaccagacaccaaatctgttggcaccttgatt

tttgacttctcagcctctggaactgtaagaagtaaatttctgttttttga

taaaccatatagtctctggtattttgttacagcagcctgaatggacta--

-----TGCT-CAC--------------------TTAAAAATGAGAAGTTT

AAAAAAAAAAATAAATGAATAAAAAGAAGTTTCAAATACAGTGTGGTTAA

AATGGTGAAGTCATTTTTATTTCAGTACTTGACAAAATACCCCATAAAAT

TCAAAACTTCTACCATTATACAAAAATAGGTCTCTACAAGTGATAGTATC

TGTCTTCATCACCAGTAGCAAATATATTAGGGCAGAAACATGTAA-TTCA

GTAGCCTTATAAGAAAAGTGCTGG-ACACCTCAAGCTACACATTC

>bat1

ATGGAAAATGCT-----GCAATAGAAGGAGAGAGATCCCAGTAGGACCTC

CAAGATTATCACAAC--TTTCTGAATCTCTAAGTCCCACGAATTTCTGGT

AGGTAATGACACA---ATATGCAC----tgccacagactgaatgtttgtg

t--cccccaccccca-ctcatatgtagaaacctaatccccaatttgttgg

tattca---ggaatggggccttt---------------------ggaggc

gattccatc-atggtggtgctctcctgaacgggagagttcccacgca---

---tgtcccttctgccatgtgaggacacaccaagaagacggctgtctacg

agccaggaagcaggcccctaccagtcactgaatctgg-------------

------ttcccagtctccaaaacggtgaggaataaatttctattgtttt-

taaggtaggcagtctatggtattctgttatagcatgctgagtggactaAG

AA---CTATGCAC---CAAGAACTATGGCTCAGTT-AAAATG--------

-----------------------AAGAGGGTTCAAATACAGTATGGTTAA

AATGGTGAACTCATTTTTATTTCAGTACTTGACAAAATACCCCATAAAAT

TCAAAACTTCTACCATTATACAAAAATAGGTCTCTACAAGTGATAT---C

TGTCGTCATCACCAGTAGCAAATATATTAGG-CAGAAACATGTAACTTCA

GTAGCCTTTTAAGAAAAGTGCAGG-ACA-CCTCAGCTACACATTC

>shrew

ACGGAAAAATGAAAAGGTATAAAAGAAATGG--AACTCTAGTAAG-AATA

TCCGAA----TATCTCGATGGAATCTTCTGATCTAGTCCAAATTT-----

----CGAGCTACG---TAGTTTAC---ttgccat-------tgtttagtg

t--cgcc-tat-cca-ttcata-tttgtgaccca-----------tttcc

tgttcactgagagtggggcttttgg-aggtgactgggtca-cagg--agt

agtttgcatggagggtttaattccct----gaataagctaccctgtgtcc

ttcagagctccctagcactttacaccatatggggacagcaagaagctata

gaccatgacgctggccttca-ataaacatg-tcttgg-------------

---------caccctgtcaaaacgg---------aaatctccttttaatg

attagtcaccattgtatgggattttgttggagtagcctgaatgtacca--

-------------------------------AGTGAAAAA----------

------------------------GAAGGTTCATTATACAGTATATTTAA

AATGGTGAAGTCATTTTTATTTCAGTACTTGACAAAATACCCCATAAAAT

TCAAAACTTCTACCATTATACAAAAATAGTTCTCTACAAGTGATAT---C

TGTCCTCATCACCGGTAGCAAATATATTAGG-CAGAAACATGTAACTTCA

GTAGCCTTAGAAGAAAAGTGCAGGAACACCTCAAGCTACACATTC

>mouse

ATTAAGAATGTTCTTT---AATGAAGGCAGAGCAAAACCA----------

---------------CACTTTGGATCCT-GAGCT-----------GAGAC

AAGTAACAAGATG-------------------------------------

--------------------------------------------------

--------------------------------------------------

--------------------------------------------------

--------------------------------------------------

--------------------------------------------------

--------------------------------------------------

--------------------------------------------------

-----ATAGGGAC---CACGAGTTGTGGCTTAGTGAAAAATCA-------

------------------------AGAAGGTTCAACTACAGTAAGGTGAA

AATGGTGAGGTC-TTTTTATTTCAGTACTTGACAAAATACCC-ATAAAGT

TCAAAACTTTGACCATTATACAAAAATAGGTCTCTACAAGTGATATG--T

TGTCTTCATCACCAGTAGCAAATATATTAAG-CAGAAACATGTAACTTCA

GTAGCTTTGTAAGAAAAGCGCAGG-GCACCGCAAGGTACTCGTCC

>rat

ATTAAAAATGTCCTTT---AATGGAGGGAGAGAGAAACCA----------

---------------CTTTTTGGAACCT-GAGCT-----------GAGAC

AAGTAACAAGATG-------------------------------------

--------------------------------------------------

--------------------------------------------------

--------------------------------------------------

--------------------------------------------------

--------------------------------------------------

--------------------------------------------------

--------------------------------------------------

-----ACAGGGAC---CACAAGTTGTGGCTTGGTGAAAAATCA-------

------------------------AGAAGGTTCCACTACAGTACAGTGAA

AATGGTGAGGTCATTTTTATTTCAGTACTTGACAAAATACCC-ATAAAGT

TCAAAACTTAGACCATTATACAAAAATAGGTCTCTACAAGTGACATG--T

TGTCTTCATCACCAGTAGCAAATCTATGAAG-CAGAAACTTGTAACTTCA

GTAGCTTTGTAAGAAAAGCGCAGG-ACACCGCACGCTATGCATCC

>rhesusmonkey

ATGGAAAA--CTTTTC--------------------AACAGTATTAACTC

TAAGATTAGGACAACTTTCTCTGAATCTCTCACTCCTACATATTTGACAT

AAGTAATGAGATG-------------------------------------

--------------------------------------------------

--------------------------------------------------

--------------------------------------------------

--------------------------------------------------

--------------------------------------------------

--------------------------------------------------

--------------------------------------------------

-----CTATGTAC---CAAGAACTATAGCTCAGTTAAAAATGA-------

------------------------AGAAGGTTCAAATACAGTGTGGTGAA

AATGGTGAAGTCATTTTTATTTCAGTACTTGACAAAATACCCCATAAAAT

TCGAAACTTCTACCATTATACAAAAATAGGTCTCTACAAGTGGTA---TC

TGTTTTCATCACCAGTAG--AATATATTAGG-CA-AAACATGTAACTTCA

GTAGCCTTATAAGGAAAGTGCAGG-ACACCTCGAGCTACACATTC

>human

ATGGAAAA--CTTTC---------------------AACAGTATTAACTC

TAAGATTAGGACAACTTTCTCTGAATCTCTCACTCCTACATATTTGACAT

AAGTAATGAGATG-------------------------------------

--------------------------------------------------

--------------------------------------------------

--------------------------------------------------

--------------------------------------------------

--------------------------------------------------

--------------------------------------------------

--------------------------------------------------

-----CTATGTAC---CAAGAACTATGGCTCAGTTAAAAATGA-------

------------------------AGAAGGTTCAAATACAGTGTGGTGAA

AATGGTGAAGTCATTTTTATTTCAGTACTTGACAAAATACCCCATAAAAT

TCAAAACTTCTACCATTATACAAAAATAGGTCTCTACAAGTGATATTATC

TGTCTTCATCACCAGTAG--AATATATTAGG-CA-AAACATGTAACTTCA

GTAGCCTTATAAGAAAAGTGCAGG-ACACCTCGAGCTACACATTC

>chimpanzee

ATGGAAAA--CTTTC---------------------AACAGTATTAACTC

TAAGATTAGGACAACTTTCTCTGAATCTCTCACTCCTACATATTTGACAT

AAGTAATGAGATG-------------------------------------

--------------------------------------------------

--------------------------------------------------

--------------------------------------------------

--------------------------------------------------

--------------------------------------------------

--------------------------------------------------

--------------------------------------------------

-----CTATGTAC---CAAGAACTATGGCTCAGTTAAAAATGA-------

------------------------AGAAGGTTCAAATACAGTGTGGTGAA

AATGGTGAAGTCATTTTTATTTCAGTACTTGACAAAATACCCCATAAAAT

TCAAAACTTCTACCATTATACAAAAATAGGTCTCTACAAGTGATATTATC

TGTCTTCATCACCAGTAG--AATATATTAGG-CA-AAACATGTAACTTCA

GTAGCCTTATAAGAAAAGTGCAGG-ACACCTCGAGCTACACATTC

>elephant

GAGAAAAACCCCAAGAGTACC---------------------TAATTCTA

AGATCAGGACAGCTTTCTTTCTGAATCCATAAGTCCCATATACCCGAGAC

AAGCAATTGAGAG-------------------------------------

--------------------------------------------------

--------------------------------------------------

--------------------------------------------------

--------------------------------------------------

--------------------------------------------------

--------------------------------------------------

--------------------------------------------------

----GATATGTAC---CAAGAATTATGGCTCAGTTGAAAAT---------

-------------------------GAAGGTTCAAATGTACAGTGGTTAA

AATGGTGAAGTAATTTTTATTTCAGTACTTGACATAATACCCCATAAAAT

TCAAAACTTCTTCTATTATACAAAAATAGGTTTCTACAATTGATATTTTC

TGTCTGCCTCACCAGTCTCAAATATATTAGG-CAGAAACATGTAACTTCT

GTAGCCTTT-TAAGAAAAGCGCAGCCTCTCTCAAGCTACACATTC

>armadillo

AT--------CTTTGGACACAAGT------------AACAAAGTATCATT

T-GTTAAGATTAAGTACAACTCTCAACCTGTAAATCATA-TATTTGAGAC

AAGTAATGAAAGG-------------------------------------

--------------------------------------------------

--------------------------------------------------

--------------------------------------------------

--------------------------------------------------

--------------------------------------------------

--------------------------------------------------

--------------------------------------------------

-----ATATGCAC---CAAGAATCATGGCTTGGTTAAAATGA--------

-----------------------AGAGGGTTCAAATAAACAGTTTGGTTA

AAATTGTGAAGTCAGTTTTATTTCAGTACTTGACAAAAACCCCATAAAAT

GCAAAACTTCTGCCATTATACAAAAATAGGTCTCTACAAGTGATATTTTC

TGTCTGCATCACCAGTAGCAAATATATTAGGCAGATACAAGTAACTTCAG

AAGGCTTATATAAGAAAAGTGCAGGACACCTCAAACTACACATTC

Marker 4d:

>MER34C

--------------------------------------------------

--------------------------------------------------

--------------------------------------------------

--------------------------------------------------

---tgtaggagaccagaatatgccaccccaaaatatgcctctttggcata

aggattattttgagctgattatt--ttgagaaactgcagacacaggagaa

gctctgaaaacagagtagaagttacccttt--tgtaagggaaatttacat

ctataaaggaaatctccatttgtaagggt---------------------

gtctccctctctgt-------accaggaagagaagga---tgactctaaa

tcactagagactctta---tc-------aatggagaaggcacc-aactta

aatctgcataacaaaccttacc---------cttgtttaccat--gcttt

tcc--tggtcacctccccataactggccttccccac--acc------ctt

ctttctttgt--------------------------ttcagcgg--aaga

tggtnatttaagcctgaatt-ctaagcca-cctctttgagatttactcat

ttctctgggtat---------------ctcccatgtatacatgaggtata

catgttattaaacttctgtttgtttttctcttgttaatctgtcttttgt-

---------tacaggggtctgtcccagctaagaactatgaagggtagaga

gaaaatta-tttttttcc-tcccctaca----------------------

--------------------------------------------------

--------------------------------------------------

--------------------------------------------------

--------------------------------------------------

--------------------------------------------------

--------------------------------------------------

--------------------------------------------------

-------------------------------------------------

>dog

CTTGAATGTAGTTGTTTTGCCAGCTCCATTCACTCCCAGAAGACCAAAGC

ACTGAGGGAGAAGACACAGAAATGGCCTCATCCTAGAGCCCAAGA-GGCC

CAAAGAGGATAGA-CT---GTCAG----GTATGGGTAGGTGGGAAA--CA

TTTTTGACTTGTGC-TGCCATTACTGAGGCCACTAGACATGT---GTGGA

----ataggctagctgagtttgccaccccaagatatgctactttggtgta

aggatgactttgagctga-agggaattaagaagtagcagatacaagaaaa

gctct----------------cttcccttccctgtttgg-----------

cta-aaagtggggcatatatttataaggt---------------------

gtc-ccctct-tgtcctctctaccaggaagagcaaaa-gttaact----g

ccagtagcaact-ttagagtc---------tggagacagtgccagaagta

--tctacatagcaaac-ttcactaactagtctttatttagcatcagttta

ccccatatttgcctttctattatttgccacccctactgacttatggtctt

tttcctttgtcacgtcacttctctaaaaatgtatcattcctttgtcaaga

agct-atataagcccaagtt-ctaaccac-tgcttgtgtcatttatt---

---tctgatcat----------------tcctttgtgcatgtgccatgca

catgttaataaatgtctgtttgcttttctcttcttaatctgtcttccat-

-------cagtccaatttacaaagcccta--ccaa--gagaacccagaag

ggtaga------ttttcc-tcccttaca---ATGAC---CACTGAGCACT

TGATATGCATC-TAGTCTGAACTGACATGTGCTGTAT--GTGTAAAA-CC

ACCCTGGATTTTGAAAACT-AAAAAAAAAT--------------GCAAAA

TATTCTATCAATAATGTTTTATATAAATTACATGTTGATGTGACAATATT

TCAGATATATTGGGTAAAAGA--------TCAAGA--TTAATTTCACCTG

TTTTCTTTTTTTTTTTTTACCTTTTTCATGGGGCTGCTGGGAAATTTAAA

A---------TGACCTATGTGG-TTACATTTGATTTCTTTGAGATAGCTC

TGCTTTAGCGGGGAGAAGAGGCAT--------------------------

----------GCACAGGATTTTGCTGGGTACCCACCTCTCCAGGGCGGA

>pangolin

CCTGAATGTGGTTGTTTTGCCAGCTCCATTCACTCCCAGGAGGCCAAAGC

ACTGAGGGAGAACACACAGAATTGCCCTCACCCTAGAGCCCAGGACGGTC

TGGGATGTCAGGCCCTAGGCCAA-----GTGTGTGAGTAAGGGAAAGGCA

TTTAAGACCTGTGC-TGCCATTATGGAAGCCACCGCCCACAT---GGGGC

----atggagtagtagaatgtgccactccaaaataagccactttggcata

aggatgattttgagctga-aagcagttaagaagatgcagatgtaagaaaa

gctct----------------ctgctctcccctatttgc-----------

cta-aaaaagg-acatgaatttaaaaggt---------------------

gtcccccctcccctctctatca----ggaaggacaaaagttaatc-----

accagagataactttaggcccttagcagcttgaagacagccccagaggaa

--tctacacaaccac-ctttactaattagcccatatctaccattagtgtc

ccacgtatttgcc-------------------------------------

---tttctacaatttgagcctcagagactccagatcctttccctttatct

tgtcacttctataaaaatgttcctttgctaaaatgctatataagcccaaa

cctaaccacccgtttgagtcattcatctccaattgctcccctgtgcatgt

gcggtgcacatgctaacaagcttatatttgtttttctcttcttaatctgt

cttttgycagtccagtcttcagtgccccagccagagaacataggcaggta

gtgagaaagagaatttccctccccaaca---GTGGC---TACTGAGCATT

TCAGACACATC-TAGTCTGAACCGACATGTGCTATAA--GTGTAAAACAC

ACATTGGATTTTGAAAACTTAAAATGAAAAAAAA----------GTAAAA

-----TATTAATACTATTTTATATGAATTACATGTTGAAGT-ACA----T

TCAAATACATTGGGTAAGATAAA---CAATCAAGA--TTAATTTCTCCTA

TTTTCTTTTT--------ATTTTTTTCATGTGACTA---GAAAATTTAAA

A---------TGACCTGTGTGCCTTGCATTTTATTTCTGTTGGCTTGCTC

TCCTCTAGAGGGGAGACGAGGCCTGAGGCCTGTGCATAAGCTGGCTTCCT

GACGCCCTTTCACATGCGATCTGCAGACTACCCACCTCTCCAGGGCGGA

>bat3

CCGGAAAGTGGTTGTTTTGCCAGCTCCATTCACTCCCAGGAGGCCGAAGC

ACTGAGGGAGAACACATAGAACTGGCCTGGCCCTAGAGCGCAAGAAGGTC

TAGAGTGCCAGGCCCTGGAGCAAAAA--GTGTGTG-TGC-----------

---------------TTCTGTCAG-GTGGCCACCGGCTCTGT---GTGG-

---tgccggg-agcgggataggctaccccaaa-catgccacttcggcata

aggaggattctgagctga-aggcaattaagaagatgcagatacaagaaaa

gctct----------------ctgccctcccctatttgc-----------

ctg-aaagcagggccca-atttacaaggt---------------------

gttttccctcccctctct---accagggaaggcaggg-gttaatc-----

cctgaagacgactttggactcctatcagcctggagatggcaccggaggaa

cctacataacaaata---tttctaactggctactatttaacattagtccc

tcatatatttgct-------------------accctggtaacaaagtcc

tttcttctgccttgtcacttt-ctaacagtctaaggtccctttgctaaaa

tgct-gtgtaagcccacatttgtaaccaactgttctagtcatttgtt---

---tccttgagtgttctagtcatttgtttcctt-gagtgtacgc--gaca

catgtgataacaaacttctctgtttctctcttgttaatctgtcttttgt-

-------cggaacaacgtagagccccagaaccggggagggtagtgggaga

ggggac-------tttcc-tcccctgca---GTGGC---CGCTGAGCACT

AGGGATGCA-C-CAGTCTGAGCTGGTGTGTGCTGTAA--GTGCCAA---C

GCACTGGGTTTCGAAGACTTAGAACAAAAGAAC-----------GTAAAA

TACCTGGTAAATAAAGCTCTATATGAATTACATGTTGAAGTGATAATATT

TCAGATATATCGGATACAATAGG---TAATTATGA--TTAATTTCACCTG

CTTCT-------------ACCTTTTTCCTGTGGCTACTAGGAAAGCTGAA

A---------TGACCTA--TGGTGACTTGTG-------------------

--------------------------------------------------

-------------------------------------------------

>mole

CTTGAATATGGTTGTTTTGCCAGCTCCATTCACTCCCAGGAGGCCGAGGC

ACTGAGGGAGAACACAAAGAACTG-CCTTGTCCGAGAGCTCAAAAAGGCC

TAGAATGTTAGGTCCTGGGGCAA-----GTATGTGGGTTACAGAGAGGCA

TTTTAGACCTGCGC-TTCCATTACAGAGGCTACCAGGCATGT---GTGGC

----aagggcaggcagaacatgccatccccaaacaggccactatggtgta

ggaatgatt-tgatctga-aggccactgagaagcagcaggtacgagaaaa

actct----------------ttgttctcccccatttgc-----------

ata-aatgcaggatagaaatgtataatttatgtaaatttgtccccagatt

gtcccccatctcaccactctac--tgtaaaggacagacactaacc-----

actggagacaactttagaccctcatcagcttggagatgacaccacaggaa

---ctg-cacaataaacatcactaactagcctttatctcccactagtttc

ccatatggcagccttcctgcaatttccgccctacacactcacagtccctt

tccctgtcctgtcacttctct---gaaaatgcatcattccttggctactc

cg------------------------------------------------

--------------------------------------------------

--------------------------------------------------

--------------------------------------------------

--------------------------------------------------

--------------------------------------------------

--------------------------------------------------

--------------------------------------------------

--------------------------------------------------

--------------------------------------------------

--------------------------------------------------

--------------------------------------------------

-------------------------------------------------

>mouse

CTTGAATGTGGTGGTTTTGCCTGCACCATTCACTCCCAGGAGGCCAAAGC

ACT--AGGAGAAAACAGAGAACAGGTGTTGCCCTAGTGACCAAGGACATC

TTCATTGCCAGACCCCAGG-CCA-----GTGTGAG-AGCTGGGATA----

-CTCATACCTGGG--TGCCATTACTGAGATTATTGGTCATGT--------

--------------------------------------------------

--------------------------------------------------

--------------------------------------------------

--------------------------------------------------

--------------------------------------------------

--------------------------------------------------

--------------------------------------------------

--------------------------------------------------

--------------------------------------------------

--------------------------------------------------

--------------------------------------------------

--------------------------------------------------

--------------------------------------------------

-------------------------------GTGGC---TACC-AACA--

TGAAACACATCTAA-TCCTAACCAACATGGGCACTAAACGTGGAA----C

ACACTGG-CTTTGAAGACTTACAACAAGGACAAAAAAGAAAAATGAAAAA

TATCTCAGTAATAGAGCTGTACATTAACTACACATCAAAATTTTG---TT

TTAGATCTATTGTGTGAAATAAAAATAGTAAAC----TTAACCCCCCCAC

ACAC--------------ACACACCTTAT-CTACTAG---AAAGTTTGAC

TTT-----------------------CATTAGATTTCTACTGTCAAGCTC

TG-GTCAGAGGGAGAAGACTCATGGTG-GAGGGCTGATTTTTC----TCA

AGAGCTATTTCATGT-TAGTTAGTACGGTACCCACCTCTCCAGGTCGAA

>rat

CTTGAATGTGGTGGTTTTGCCTGCACCATTCACTCCCAGGAGGCCAAAGC

ACT--AGGAGAAAACAGAGAATAGGAGTTGCTCTAGTGACGAAGGACATC

TTCATTGCCAGACTCCAGG-CCA-----GTGTGAG-AGCTAGGAGAATCA

TCTTAGACCTGCCTCTGCCATTACTGAGATTATTGGTCATGT--------

--------------------------------------------------

--------------------------------------------------

--------------------------------------------------

--------------------------------------------------

--------------------------------------------------

--------------------------------------------------

--------------------------------------------------

--------------------------------------------------

--------------------------------------------------

--------------------------------------------------

--------------------------------------------------

--------------------------------------------------

--------------------------------------------------

-------------------------------GTGGG---TACC-AACA--

TGAAACATATCTAA-TCCTAACCGACATGGGCACTAACTGTGTAA----C

ACGCTGG-CTTTGATGACTTAGAACAAGAACCAAAA-CAAAA--GAAAAA

TATCTCAGTAA---TATTTTATATTAATTACACATCGAAA---TTATATT

TTGGGCATATTGTATAAAATAAAAAAAAA--------TTTAATGCC----

------------------ACCTGCTGCC-ATATCTAGTAGAAAGTTTGAC

TTT-----------------------CGTTAGACTTTTACTGTCCAGCTC

TG-CTTAGAGGGAGAAGATTCGTGGTA-GAGGGCTGATTCTTC----TCG

AGAGCTATTTCATGT-CCGTTGGTATGGTACCCACCTCCCCAGGGTGGA

>rhesusmonkey

CTTGAATGTGGTTGTTTTACCAGCACCATTCACTCCCAGGAGGCCAAAGC

ACT--AGGAAAAAACACAGAGCTAGCCTGGCCCTAGAGACCAAGAAGGTC

TAAAATGTCAGACCCTAGGGTAA-----GTGTGTG-AGCTGGGGGAGGCA

TTTTAGACCTGGG---CTG-CATCAGAGGCCACGAGCCACGT--------

--------------------------------------------------

--------------------------------------------------

--------------------------------------------------

--------------------------------------------------

--------------------------------------------------

--------------------------------------------------

--------------------------------------------------

--------------------------------------------------

--------------------------------------------------

--------------------------------------------------

--------------------------------------------------

--------------------------------------------------

--------------------------------------------------

-------------------------------GTGGC---TACTGAGCACT

TGAAACACATCTAAGTCCCAATTGACATGGGCCCTGA--GTGTGAA----

-----GA--TTCAAAGACTTAAAAACAACAAAAACTT-------GTCAAA

TATCTCATTAATAAGGTTTTCTATT----ACATGTCGAAATGATAATATT

TTGGATATATTGAGTAAAATAAAAAATACTGGTAAAATTAATTTTACCTA

TTTTCTTTTT--------ACTTTTCTCATGTGGCTAGTGGAAGATTTAAA

ATTATATATGTGAC-TTGCATTATGGCATTATGTTCCTACTGAATATCTC

TGCCTTACAGGGAGGAGAGGCAGGCAC-AAGAGCTGATGT-------TCA

GAAGCCTTTCACCC-GTGGTCTGCAGAGTACCCACCTCTCCAGGGCGAA

>chimpanzee

CTTGAATGTGGTTGTTTTGCCGGCACCATTCACTCCCAGGAGGCCAAAGC

ACT--AGGAGAAAACACAGAGCTAGCCTGGCCCTAGAGACCAAGAAGGTC

TAAAATGTCAGGCCCCAGGGTAA-----GTGTGTG-AGCTGGGGGAGGAA

TTTTAGACCTGGG---CTGCCATCAGAGGCCACAAGCCATGT--------

--------------------------------------------------

--------------------------------------------------

--------------------------------------------------

--------------------------------------------------

--------------------------------------------------

--------------------------------------------------

--------------------------------------------------

--------------------------------------------------

--------------------------------------------------

--------------------------------------------------

--------------------------------------------------

--------------------------------------------------

--------------------------------------------------

-------------------------------GTGGC---TACTGAGCACT

TGAAACACATCTAAGTCCCAATTGACACGGGCCCTGA--GTGTAAA----

-----GA--TTCAAAGACTTAAAAACAACGAGAAATT-------GTCAAA

TATTTCATTAATAAGGTTTTCTATTAATTACATTTTGAAA---TAATATT

TTGGTTATATTGAGTAAAATAAAAAATACTAATGAAATTAATTTTACCTA

TTTTCTTTTT--------ACTTTTCTCATGTGGCTAGTGGAAGATTTAAA

ATTACATATGTGAC-TTGCATTATGGCATTGTGTTCCTATTGAATAGCTC

TGCCTTATGGGGAGGAGAGGCAGGCAC-AAGAGCTGATGT-------TCA

GAAGCCTTTCACAC-GTGGTCTGCAGAGTACCCACCTCTCCAGGGCGAA

>human

CTTGAATGTGGTTGTTTTGCCGGCACCATTCACTCCCAGGAGGCCAAAGC

ACT--AGGAGAAAACACAGAGCTAGCCTGGCCCTAGAGATCAAGAAGGTC

TAAAATGTCAGGCCCCCAGGGTAAGTGTGTGTGTG-AGCTGGGGGAGGAA

TTTTAGACCTGGG---CTGCCATCAGAGGCCACGAGCCATAT--------

--------------------------------------------------

--------------------------------------------------

--------------------------------------------------

--------------------------------------------------

--------------------------------------------------

--------------------------------------------------

--------------------------------------------------

--------------------------------------------------

--------------------------------------------------

--------------------------------------------------

--------------------------------------------------

--------------------------------------------------

--------------------------------------------------

-------------------------------GTGGC---TACTAAGCACT

TGAAACACATCTAAGTCCCAGTTGACACGGGCCCTGA--GTGTAAA----

-----GA--TTCAAAGACTTAAAAACAACGAGAAATT-------GTCAAA

TATTTCATTAATAAGGTTTTCTATTAATTACATTTTGAAA---TAATATT

TTGGTTATATTGAGTAAAATAAAAAATACTAATGAAATTAATTTTACCTA

TTTTCTTTTT--------ACTTTTCTCATGTGGCTAGTGGAAGATTTAAA

ATTACATATGTGAC-TTGCATTATGGCATTATGTTCCTATTGAATAGCTC

TGCCTTATGGGGAGGAGAGGCAGGCAC-AAGAGCTGATGT-------TCG

GAAGCCTTTCACAC-GTGGTCTGCAGAGTACCCACCTCTCCAGGGCGAA

Marker 5a:

>MER93B

--------------------------------------------------

--------------------------------------------------

--------------------------------------------------

--------------------------------------------------

-----------------tgttaaagtaaattaaaatggagaccaggcctg

aagaatccctgagcagacaaagccagttaggcctcataagtgaccttaac

cttgcttgatttgcaaacataagcaaaactta-acttga---gctatttc

ttgtaaatgcctatattaaagaaaaacagaacttaagctc-aaccaatca

gaagcagccaacaaact-tataattatataactagggactttccaatggg

atagaccaaataaggcaactgtataactgtaaccaatcaaatattttctt

tgctttacttctgtgttcatcctataaaagcctcccccttgcattccctc

ggtggagctcccgaaccacttctggtttggagctgcccaattcatgaatt

gctgtttgctcaaataaactctttaaaattttttattgtgcctcagttta

ccttttt---aaca------------------------------------

--------------------------------------------------

----------

>cow

TTCTGAGTCGTGTATAGAGGTGAGGGGAAGGAAATTTCACATCCTAAAGA

ACTTCAA-TATCTTTATTC-ATTTATTTCTTCTACAAATGGTCACTGAGC

CACTCCTAAGCACCAGACACTATGTTTGGTAA-CAAAAAAAGACA-CAGG

AAACTTGTTCTAGTGGAACTTAGAG--TCAAGTGGAAG-AAACAAT-AGC

CA------------------------------------------------

--------------------------------------------------

--------------------------------------------------

--------------------------------------------------

--------------------------------------------------

--------------------------------------------------

--------------------------------------------------

--------------------------------------------------

--------------------------------------------------

----------GTAGAAAG---TAAACAAACTCATCAGATCTTCACAGTTT

ATCAGGACTGTTATTCATTCATGAAAAGCTGAATGCCCAGAGATTG-CTG

GCATTGGGCC

>dog

TTCTGCCTGGTATGTAAAGCCGCAGAGAACGAAGTTTCATTTCCTAGAGA

A-TTCGAATATCTTTGTCC-ATTGATTTATTCTA-AAGCTGTTATTGGGC

CCCCACTAAGTGCTGAGCACTATGTTTGGTG-CCTAAACAAGACA-CAAG

----TTCCCCCC-TGGAACTCACAG--TCGAGTAGAGG-AAACAA-----

--------------------------------------------------

--------------------------------------------------

--------------------------------------------------

--------------------------------------------------

--------------------------------------------------

--------------------------------------------------

--------------------------------------------------

--------------------------------------------------

--------------------------------------------------

----------GAGAAAGT---CAA-CAAACTAATCAAATAATCACAACTT

ATCAAGACTGCTATTCATTCAGGAAATACTGAGTGCCCACAGAGTGACAG

GCATTGGGCC

>mouse

TTCTCACTGGAATGCGAAGCCATGAACAGAGAAACTTGACTTCCTGGAGC

ACTTCAGACAGGTGTATTC-ATTTCCGTGTTCTGCAAGCAACCATTTAGT

CCCCAGCA-GAGCCAAGAGCTGAATCGGGTT-CCTGAACAAAAAGCTGGG

GCTTCTCACAGAGTTTGCAGTT-GTTCTGGAGAAACAATGACAGGGCAGC

CAC---CAACTCAA---gatcagaacaggttgaagtagacaccaggccag

aagagctgctgagcagacacacagtccac--cctccc-tgtgaccttggc

tttgtttgatttgcaaacataagcaaaccttgcactccactcgccattcc

ttctaagtgcctccactaaaacaaaagggggcccaagcac----------

--------------------------------------------------

--------------------------------------------------

--------------------------------------------------

------------ag-ccaatcccggg-------cacccagcaactgaatt

gctctttgtccaaataaactctttaag-ctttt-attgtccctcggttta

c-ttttt---AACAAAGG---AAAATAACCATAA-------------TGT

ATCAAAGGCCCTATTCATTCATGAAACACTGAGTGTCC--AGTGTGGGTG

CCCTTCAGCC

>rhesusmonkey

TTCTGAGTGGTATGTAAAGTCAAGGAGAAAGACATTTCACTTTCTAGGGA

ACTTTAAATCTCTTTATTC-ATTTATTTATTTTGCAAATAGTCATTGAGC

CACTATTAAGTGTTAAGCACTACGTTTGGTGACCAAAGAAGAGG--TGGG

GACCTTGCTTTCATGGACCTCA-CAGCCTAA-TGGGGG-AATCTACAGTA

TTC---CAACACAG---tggtaaagtaagttaaaatgg---tcaggcctg

aagaaactatacgcagacatagccagcaagacctcagatgtgaccgtaac

cttgcttgatttgcaaacataagcaaaactga-acttga---gcaatttc

ttgtaaatgcctatgttaaagaa---tacaacttaaactccaaccagtca

gaagttaacaaacaaatgtgtaattatgtaactagggaatttccaacagg

atagatcaaataaggtaactgtataactgcaatcagtcaaatatt-tccc

ttcattacttccatgtttatcctataaaagcctcccccttgcaatccctc

g-tggagctcccagaccagttctggtttggagccacccagttcttgaatc

actgtttgctcaaataagctcattaaa-ttttt-actgtgcctcagttta

c-tcttt---AGCAGAAG---TAAACAAACTAATAAAATAATCACAATTT

CTGAAAAGTGTGATTGATTTATGAAATTTGGAGTGCCCC-AGTATGACAG

GCAGTGGGCA

>human

TTCTGAGTGGTATGTAAAGTCAAGGAGAAAGACATTTCACTTTCTAGGGA

ACTTTAAATCTCTTTATTC-ATTTATTTATTTTACAAATAATCATTGAGC

CACTATTTAGTGCCAAGCACTATGTTTGGTGA-CCAAAGGAGAGG-CGAG

GACCTTGCTTTCGTGGACCTTG-CAGCCCAA-TGGGGT-AATCAACAGTA

TTC---CAACACAG---tggtaaagtaaattaaaatggagaccaggcctg

aagaaactataagcagacatagccagcaagacctcagatgtgacctgaac

ctagcttgatttgcaaacataagcaaaactta-acttga---gcaatttc

ttgtaaatgcctatattaaagaagaatacaacttaaactccaactaatca

gaag-taacaaacaaatgtgtaattatgtaactagggaatttccaacagg

atagatgaaataaggtaactgtataactgcaatcagtcaaatattttctc

tgcattacttccatgtttctcctataaaagtctccccattgcaatccctc

a-tggagttcccaaaccagttctggtttggagccacccaattcttgaatc

actatttgctcaaataagcccattaaa-ttttt-attgtacctcggttta

c-tcttt---AGCAAAAG---TAAACAAACTAATAAAATAATCACAATTT

CTGAAAAGTGTGATTTATTTATGAAATTTGGAATGCCCC-AGTATGACAG

GCAGTGGGCA

>chimpanzee

TTCTGAGTGGTATGTAAAGTCAAGGAGAAAGACATTTCACTTTCTAGGGA

ACTTTAAATCTCTTTATTC-ATTTATTTATTTTACAAATAATCATTGAGC

CACTATTTAGTGCCAAGCACTATGTTTGGTGACCAAAGGAGAGG--CGAG

GACCTTGCTTTCGTGGACCTTG-CAGCCCAA-TGGGGT-AATCAACAGTA

TTC---CAACACAG---tggtaaagtaaattaaaatggagaccaggcctg

aagaaactataagcagacatagccagcaagacctcagatgtgacctgaac

ctagcttgatttgcaaacataagcaaaactta-acttga---gcaatttc

ttgtaaatgcctatattaaagaagaatacaacttaaactccaactaatca

gaag-taacaaacaaatgtgtaattatgtaactagggaatttccaacagg

atagatgaaataaggtaactgtataactgcaatcagtcaaatattttctc

tgcattacttccatgtttatcctataaaagtctccccattgcaatccctt

g-tggagttcccaaaccagttctggtttggagccacccaattcttgaatc

actatttgctcaaataagcccattaaa-ttttt-attgtacctcggttta

c-tcttt---AGCAAAAG---TAAACAAACTAATAAAATAATCACAATTT

CTGAAAAGTGTGATTTATTTATGAAATTTGGAATGCCCC-AGTATGACAG

GCAGTGGGCA

>elephant

TTCTGAGTGTTATAGAAAGCTAAGGAGGAAGAAATTTCACTTCCTGGAGA

ACTTTCAAA------TATC--TTTATTTATTCTACAAATAGTTATTGAGC

AACTCCTAAGTGCCAGGCATTATGTTTGGTGG-CTAAACAAGAGA-TAGG

GACTCTGTTCTCCTGGAACTTA-CAG-TCTAGTGAAAG-ACACCA----C

CA------------------------------------------------

--------------------------------------------------

--------------------------------------------------

--------------------------------------------------

--------------------------------------------------

--------------------------------------------------

--------------------------------------------------

--------------------------------------------------

--------------------------------------------------

----------CCACAAAG---TAA-CAACAATGATAAATA-TCACAGTTT

ATCGAAGTGTTTTT-CATTCAGTAAATATGAAGTGTCTACAATGTGACAG

GCATTTGGCC

>armadillo

TTCTGAGCAGTATATAAAGCTAAGGAAAAAGACATTTTATCTTCTGGAGA

ACTTCCAGAAATATTTACTCATTTATTCATTCTACAAAAAGTTATTGAGC

TGCTACTAAGTGCCAGGCACTATATCAG-TGC-TAAAACAAGAGAACAGG

GACCTTGTTTTCATGGAACTTAACAGTTCTAGTGGAGGGAAACAA-----

--------------------------------------------------

--------------------------------------------------

--------------------------------------------------

--------------------------------------------------

--------------------------------------------------

--------------------------------------------------

--------------------------------------------------

--------------------------------------------------

--------------------------------------------------

----------CAATTAAC---AAAGTTAAC--------------------

--------------------------------------------------

----------

Marker 5b:

>L1MA9

-------------------------------------------agtagat

ctta-agtgttctcaccacacacaaaa---aaatggtaactatgtgaggt

gatggatatgttaattagcttgattgtggtaatcatttcacaatgtatac

gtatatcaaaacatcacgttgtacaccttaaatatatacaatttttattt

gtcaattat-----------------------------------------

---------acctcaataa----------------agctggaaaaa----

--------------------------------------------------

--------------------------------------------------

-------------------------

>cow

TAGGCTCTTTATAACTATAAA-----------------------------

--------------------------------------------------

--------------------------------------------------

--------------------------------------------------

--------------------------------------------------

-------------------------------------------------G

AATGTGACCCATTGC---AGAGAAGGGGGTATTTCTTTAAAGAATCCTA-

-----TTTGTGTATCAAAAACAATGCAGTTATCTAATATAAGTGCAAAGA

CATTACAAATACTATTTAACAAT--

>pig

TAGGCTTTCTATAACTGTAAA-----------------------------

--------------------------------------------------

--------------------------------------------------

--------------------------------------------------

--------------------------------------------------

-------------------------------------------------G

AATCTGACCCACTTC---AGAGAAAGGGATATTTCTTTAAAGAATCCTA-

-----TTTGTGTATCAAAAACAATGTGGTTATCCAATATAAGTGCAAAGG

CATTACAAATACTGTTAAAAAATTC

>dog

TAGACTCTCCATAACTGTAAA-----------------------------

--------------------------------------------------

--------------------------------------------------

--------------------------------------------------

--------------------------------------------------

-------------------------------------------------G

AATGTGACCCATTTC---AGAGAAAGGGGTATTTTTTTAAAAATCCTGT-

-----TTGCATATCGAAACACAATGTAGTTATCTAATATAAATGCAAGGA

CATTACAAATATATTTTTTAAATTC

>cat

TAGATTCTCGGTAACTGTAAA-----------------------------

--------------------------------------------------

--------------------------------------------------

--------------------------------------------------

--------------------------------------------------

-------------------------------------------------G

AACGTGACCCATTTC---AGAGAAGGGGTTTGTTTTTTGTTTTTTTCAAT

CCTGCTTGTGTATCAAAACACAATGTAGTTATCTAATATAAATGCAAAGA

CATTACAAAATATTTTTTAACCTTC

>bat4b

TAGACGCCCCGTAACTGTCTA-----------------------------

--------------------------------------------------

--------------------------------------------------

--------------------------------------------------

--------------------------------------------------

-------------------------------------------------G

CATGTGACCCATTTC---AGAGAAAGGGGTATTTACAAAAAATCCTATT-

------TGTGTACCGAAAA-CAATGTAGCTCTCTGATATAAATGCAAAGG

CGTTACAGATATTGTT-AAAAATGC

>mouse

TAGCCCCTCTGTAATTGTAAA---GGATGTGACCCATTTC---agtagat

cttaagtgttctca-catacacagaaatgaaaatagtaaccaggcgaggg

gatggctgtgctaattagtttgcttatggtgattatttcacagtgcatac

tatatgcaaacaacaagttttatgccttaaatatatactactttttactt

gccattcactgttcagtaaccccccaaaaaatcagtaacag---------

-------aaacccaaaaccgaacacccccaaa--acaaataaaaca---A

ATC-CAACCCATTTC---AGAGAAACAG-TATTTTGAGTAAAATCTCAT-

------TTGTGTGTCAAGAACA-TGTAGTTACCAAATATTAATGCAAAAA

TATTTCAAGTATTGTTTAAAAATTT

>rat

TAGCCCCTCTGTAATTGTAAA---GGATGTGACCCATTTC---aggagat

cttaagtgttctca-catacacagaaatgaaaatagtaaccaggcgagga

gatggctatgctaattagtttgcttatggtgattattttacagtgcatac

tatatgcagacatcaagttttatgccttaaatatataccacttactgctt

agcagtcactgttcagtaaacaaaaataaaaaagaaagaaaaagaaaaat

caacaacaaaaacaaagcc--aaacacccccacaacaaataaaata---A

ACC-CAATTGATTTC---AGAGAAACGG-TATTTTGAATAAAACATCAT-

------TTGTGTGTTAAGAACA-TGTAGTTACCAAATATTAATGCAAAAA

TAATTCAAGTATTGTTTTAAAATTT

>rhesusmonkey

TAGACTCTCTGAAACTGCAAA---GAATGTGAGCCATTTC---agtagat

ctt--gtgttctcaccacacacagaaaggaaactagtaactatgtgaggt

gatgaatattttaattagcttaattctgttgattatttcacaatgcataa

tatgtcaaaatgtcaagttgtacaccttaaatata---taatttttattt

gtcagttat-----------------------------------------

---------acttaaagct----------------------aaaaa---G

ATA-TGACCCATTTT---AGAAAAGTGGTTATTTTTTTTAAATTCCTAC-

------TTATATGTCAA-AATAATGTAGCTATCCAATGGAAATGCAAAGT

CCTTACAAATATTGTTTAAAAATT-

>human

TAGACTGTCTGTAACTGTAAA---GAATGTGAGCCATTTC---agtagat

ctt--gtgttctcaccacacacaaaaaggaaactagtaactatgtgaggt

gatggatattttaattagcttgattctgttgattatttcacaatgcataa

tatgtcaaaatgtcaagttgtacaccttaaatata---taatttttattt

gtcagttat-----------------------------------------

---------acttaaagct----------------------aaaaa---G

ATA-TGACCCATTTT---AGAAAAGCGGTTATTTTTTTAAAATTCCTAC-

------TTATATGTCAA-AACAATGTAGCTATCCAATACAAATGCAAAGT

CCTTACAAATATTGTTTAAAAATTC

>chimpanzee

TAGACTGTCTGTAACTGTAAA---GAATGTGAGCCATTTC---agtagat

ctt--gtgttctcaccacacacaaaaaggaaactagtaactatgtgaggt

gatggatattttaattagcttgattctgttgattatttcacaatgcataa

tatgtcaaaatgtcaagttgtacaccttaaatata---taatttttattt

gtcagttat-----------------------------------------

---------acttaaagct----------------------aaaaa---G

ATA-TGACCCATTTT---AGAAAAGTGGTTATTTTTTAAAAATTCCTAC-

------TTATATGTCAA-AACAATGTAGCTATCCAATACAAATGCAAAGT

CCTTACAAATATTGTTTAAAAATTC

>elephant

TCGACTGTCTGTGATTGTAAG-----------------------------

--------------------------------------------------

--------------------------------------------------

--------------------------------------------------

--------------------------------------------------

-------------------------------------------------G

AATGTGGCCCATTTC---TAAGAAAGAGGTAGTTAAACAAAAATAAAAAT

CCCTATTCATGTATCAAACACAATGCAGCTGTCTAATATAA-TACAAAGA

CATGACAAATATTGTTGAAAA-TGC

Marker 5c:

>MLT1A0

----------------------------------------tgctatggtc

tgaatgtttgtgtccccccaaaattcatatgttgaaacctaatccccaat

gtgatggtattaggaggtggggcctttgggaggtgattaggtcatgaggg

cggagccctcatgaatgggattagtgcccttataaaagagaccycagaga

gctcccttgccccttccgccatgtgaggacacagtgagaaggcgccgtct

atgaaccaggaaatgggccctcaccagacactgaatctgccggcgccttg

atcttggacttcccagcctcca-gaactgtgagaaataaatttctgttgt

ttataagc-tacccagtctatggtattttgttatagcagcc----cgaa-

----cagactaaga------------------------------------

--------------------------------------------------

--------------------------------------------------

-------------------

>dog

AAATTATTTCAT-AGTACATCTAGAAG-----------------------

--------------------------------------------------

--------------------------------------------------

--------------------------------------------------

--------------------------------------------------

--------------------------------------------------

--------------------------------------------------

--------------------------------------------------

-----------------GGCCT-TC----CTCATAGAAAATTACCCTCTA

TCAACCA-AATGTAAAATCTTGTGCTATCATCCTACC--CCAAGATCCC-

--AGGACTCCTGCCTTAGAATTG---CCCTCTGCTAGGGGCTGGTCTCTG

ATCCAAGCAGGGCAGCCTT

>bat4

AAATTATTTCAT-GGTATATTGGGAAA-----------------------

--------------------------------------------------

--------------------------------------------------

--------------------------------------------------

--------------------------------------------------

--------------------------------------------------

--------------------------------------------------

--------------------------------------------------

-----------------GGCCC-TC----CACATGGAAAAGTACCCACAA

TCAGCTA-AATGTAAAGCCTCATACTATCACCCTAC---CCAAGCTCCCA

ACAATGCTGCTGCCTTAGAACT----TCCTCTGCTAGAGGCGGGTCTCTG

GGCCAAG-AGGGCTACCTC

>bat5

AAGTTGTTTCAT-GGTATATTGGGAAA-----------------------

--------------------------------------------------

--------------------------------------------------

--------------------------------------------------

--------------------------------------------------

--------------------------------------------------

--------------------------------------------------

--------------------------------------------------

-----------------GGCC-TTC----CACATGGGAAAGTACCCACAA

CCAACTA-AATGTAAAGCCTCATACTGTCATCCTACCTACTAAGCTCCCA

ACAATGCTGCTGCCTTAGAACTGTCTAACACTGCTAGAGGCAGGTCCCTG

GGCCAAGA-GGGCTACCTC

>mouse

----------------------AGAAA---TGGCTTC---cactgtgaca

tgaaggacaatgtccctctaaagttcatatgttgtaacctaatattgtat

gtgatactatgaaaaagt-----ga-------------------------

------------------------------------------------tt

cctctggacaagtgattgagttatgaaaactcagggagaagttaacattg

gtgaagtggagaag------------------------------accttg

atcctggactttacagcctctaaggactaaggacaataaagtgctgttgt

ttataaat-tatcaagtgtgaagttttttattatcccatcc----tgaa-

----tggactaaga---TGGCT-----------CAAAACGTTACCCATAA

TCAAGTG-ATTGTAAAA--CCTTGTGTTATCTTACCATG---AACTCCC-

--AAGCCTTTTGGTTCAATATTG---TTCTCTGCTAGA------------

----AACCA------CTTC

>rat

---------------------TAGAAA---TGGTTTC---tgctgtgaca

tgagtattaatgtccttctaaagttcatatgttggaacataatactgtat

gtgatactattaagaaat-----ga-------------------------

------------------------------------------------tt

cctccagacaagtgattgaatcatgaaatctcaatgagaagctatcattg

gtaaagttgaaaag------------------------------accttg

atcttggattttataccttccaaggactgcagacaataaagttctgttgt

ttataaattgtgaaatgtgaagg-attttattataccaact----tgaa-

----tggactaaga----------------------AACTTTACCCACAG

TCACCTG-ATTGTAAAG--CCTTGTGTTATCCTATGATG---AACTCCC-

--AATACTTCTGTTTTAGAATTG---TTCTCTGCTAGA------------

----AACCA------CTTC

>squirrel

AAATTATTCCCCTGTTCATTTTGAAAA----GTCTTC---tactatgatc

ttcatgttgatgttcccctaaactcatattttataatctaataccctact

gtga---tattaagaggtggggcctgttggg-------------------

-------------------------------cccaccttccctgttcacc

cctaccccaaagtgcttaactcatgagagctcaatgagaagtggcatcaa

tgaagaagagtgag--acc-tcaccggacactgcctctcccactgccttg

accttaaactttccagcctcta-gagccatgggtgatagatttctgttgt

ttacaaat-tatccagtatgagatatttcgtta-----------------

-----------------CACCCTTC---------TGAAT-GTACCCATGA

TCAACTA-AATGTAAAA--CACCACTCAGTTATCCGACCCCAAGCTCTCC

---AGACTGCTGGCTTATAACTG-CTCTCTGCTAGAGGCACACTTCTGAG

A-CT--GCTGGGCCACTGT

>rhesusmonkey

AAATTATTTCAT-CATACATTTGGAAA---GGCCTTC---tgctatggtc

tgaatgccggtgtacctccaaaatttatatgttgaaacttaatacccaga

gtgatagtattaaggggtggggc---------------------------

--------------------------------------------------

-cttttgtgaggtgattaagtcatgagggaggagcaaggaggtgccatca

ttgaagcagaga--gcgccttcaccagacacgaaatctgccagcacctcg

atcttggactttccagcctcca-gaaaagtgagcaataaattcgtgttgt

ttataaat-tgcccagtgtaaggtgtttttttgtgccatcc----tgaa-

----ccaattaaga---TGTGTTTC---TCACA-GAAAAAGGA-CCACAA

TCAACTA-ACTGTAAA---TCTTGTGCTATTATTCTATGGCAAGCTTCC-

--AAGACTGCTGCTTTAGAATTG---ACCTCTGCTAGAGGCAGGTCTCTG

AACCAACTGGGGCTGCCTT

>human

AAATTATTTCAT-TGTACATTTGGAAA---GGCCTTC---tgctatggtc

tgaatgctggtgtaccctcaaaatttatatgttgaaacttaatacccaga

gtgatagtattaaggggtggggaga-------------------------

--------------------------------------------------

-cttttgtgaggtgattaagtcatgagggaggagcaaggaggtgccatca

gtgaagcagaga--gggccttcaccagacacggaatctgccagcaccttg

atcttggacttgccagcctcca-aaaccgtgagcaataaatttgtgttgt

ttataaat-tacccagtgtaaggtgttttgttgcaccatcc----tgaa-

----ccaactaaga---TGCCTTTC---TCACA-GAAAAAGGA-CCACAA

TCAACTA-ACTATAAA---TCTTGTACTATTATTCTATGGCAAGCTCCC-

--AAGACTGCTGCTTTAGAATTG---CCCTCTGCTAGAGGCAGGTCTGTG

AGCCAACCAGGGCTGCCTT

>chimpanzee

AAATTATTTCAT-TGTACATTTGGAAA---GGCCTTC---tgctatggtc

tgaatgctggtgtaccctcaaaatttatatgttgaaacttaatacccaga

gtaatagtattaaggggt--ggaga-------------------------

--------------------------------------------------

-cttttgtgaggtgattaagtcatgagggaggagcaaggaggtgccatca

gtgaagcagaga--gggccttcaccagacacagaatctgccagcaccttg

atcttggactcgccagcctcca-gaaccgtgagcaataaatttgtgttgt

ttataaat-tacccagtgtaaggtgttttgttgcaccatcc----tgaa-

----ccaactaaga---TGCCTTTC---TCACA-GAAAAAGGA-CCACAA

TCAACTA-ACTATAAA---TCTTGTACTATTATTCTATGGCAAGCTCCC-

--AAGACTGCTGCTTTAGAATTG---CCCTCTGCTAGAGGCAGGTCTCTG

AGCCAACCAGGGCTGCCTT

>treeshrew

ATATCATTTCAC-TGCACATTGGAAAA---GGCCTTC---tgctacggtt

tgaatgctggtgttcccccagaacccatgtgttggaccctaatacccaat

atgatagtattaag------tgcca-------------------------

-----------------------------------------------ggt

cctttggggacatgattaagtcatgagggcagagtgagaagtcttcattg

-aggaatcagag-----ccttcactggacattgaattggctagtgccttg

attttggatgtcccagcctcct-gaacggtaagcagtacatttctgttgt

ttacgaat-tacccaacagaaggtatttcattaaaccatctagaatgaat

gcctgtgaggaaga---CACCTACC---TCCCA-G-AAAAGTAGCCACCA

TCAGCTAAATGTGAAA---CCTCGTGTTATTTCCCTACCCCAAGTTCCC-

--ACTACCGCTGCCTTCTAATTG--CCCTCCTGCTGGAGACAGGTCCCTA

AGACAACATGGACCGCTTC

Marker 5d:

>MLT1A

--------------------------------------------------

-------------------------------tgctat-gga--ctgaatg

tttg-------------tgtcccccc------------------------

-aaaattc-atatgttgaag-ccctaatc-cccaatgtgatggtattagg

a-ggtggg-gcctttgggagg-tgattaggattagatgaggtca-tgagg

gcggggccctcataatgggattagtgcccttataaaagagaccycagaga

gctcccttgccccttccgccatgtgaggacacagtgagaaggcgccgtct

acgaaccagggaatgagccctcaccagaaactgaatctgccggcgccttg

atcttggacttcccagcctccagaactgtgagaaataaatttctgttgtt

taagctacccagtctatggtattttgttatagcagcccgaacagactaag

aca-----------------------------------------------

----------------

>cow

CACTAATAACATTTTTAGTTTGGGTGAGGGTGGAAATGGACACTGCT--C

ACTCTGGCAATTACTCTACTCT----------------------------

--------------------------------------------------

--------------------------------------------------

--------------------------------------------------

--------------------------------------------------

--------------------------------------------------

--------------------------------------------------

--------------------------------------------------

--------------------------------------------------

------CCTGG---TAATAAATGCAATCATCATAAAAATCAAAATTAATA

AACTGTGTTTGCAAAC

>pig

CACTAATAACATTTTGGGTTTGGGTGAGGG-CAGAATGGCCACTGCT--C

ACTCTGGATATTACCTTACTCT----------------------------

--------------------------------------------------

--------------------------------------------------

--------------------------------------------------

--------------------------------------------------

--------------------------------------------------

--------------------------------------------------

--------------------------------------------------

--------------------------------------------------

------CCTAG---TCACAAACGCAATCAGCATAAAAATCAAAATTAATA

AACTGCAGTTGCAAAC

>dog

CAGCATTAATATTTTTAGTTTGGCTGAGGG-TGGAATAGATACTGTT--C

ACTATGGCAATTACCTTACTCT----------------------------

--------------------------------------------------

--------------------------------------------------

--------------------------------------------------

--------------------------------------------------

--------------------------------------------------

--------------------------------------------------

--------------------------------------------------

--------------------------------------------------

------CCCAA---TAACAAATGCAATAATCGTAAAAATCAAAATTAATA

AACTGTGGTTACAAAC

>shrew

CACTAATAACA-TTTTGAGTTTGGATGGGGGTGGAA-GGACACCGTT--C

GCTATGGCAATTACCCTCCTCC----------------------------

--------------------------------------------------

--------------------------------------------------

--------------------------------------------------

--------------------------------------------------

--------------------------------------------------

--------------------------------------------------

--------------------------------------------------

--------------------------------------------------

------CCTGG---TGACAAACACAATCATCATAAAAATCAAAATTAATA

AACTGTACTTGCAAAC

>hedgehog

CACTAATTACATTTTTAGTTTAGGTGAGTGTAGAAGGGACACTGTT---G

GCTGTGGCAATTTCCCTACTCT----------------------------

--------------------------------------------------

--------------------------------------------------

--------------------------------------------------

--------------------------------------------------

--------------------------------------------------

--------------------------------------------------

--------------------------------------------------

--------------------------------------------------

------CCTAG---CAACAAATGCAATAATCATAAAAATCAAAATTAATA

AAGTAGAGCTGCAAAC

>mouse

CATCCACAACATTTTTAATTTGGATGAAGC-TGAGATGGACTGTGTT--C

GCCAGGGCAATTGCTCTACTC---TACAG--cattatgaga--gctcatg

ttta---tcttggccacccctcccccactcccca----------ccccct

acaaattc-atggattgaaa-cattca-ttgccaatgctatg-catttgg

a--tatgaggcctttaggaag-tcattggatttagatttgatca-tgaag

gtcggccctt-------------cacataaaggttaa---tgtcttcaga

agatgaagtaga---atc-caccatctc------------------cttc

tgcccaaccccatgcgcaaccaagaaaaagccgaggaagaacacaccaag

aagacggcggtcaata--ggcagaactgtggggaatgaaggcttgttgtt

taaactgcccactccatgacattttgttatagcagccccagctgactaag

cca---CCCAG---GAGCAAATGCAATAATCGTAAAATTCAAAATTAATA

AACTGTAGTTGCACAT

>rat

GATCCA---CATTTTTAACTTGTATGAAAC-TGAGATGGGCTGCATT--C

ACCAGGGCAATCA--CTGCTCT--CCCAG--cattatgaga--gctcgtg

ttta---tcttgaccaccccaccc-----caaccccggtccctgccacct

acaaactc-atggattaaaa-cattca-ctcccgatgtcacg-catttgg

a--tatgaggcctttaggag-ttcattggatttagatgtgatcactgaat

gtcagcccct-------------cacataaaagttaa---cgtcttcaga

agatgaagtaga---act-cacccgcccaccatct-----------cctc

tgcccaaccccatgtgcaaccgagaaaaagccaaggaagaacactgcaag

aagacagtggtctacatagccagaacggtggggaataaatgctcattgtt

taaactgtccactctgtgacattctgttatagcagccccagctgactaag

cca---CCCAG---GAGCAAATGCGATAATCATAAAATTCAAAATTAATA

AACTGTAGTTGCACAT

>marmot

------------TTTCCATTTGGATGAGGG-CAGACTGGCCACTGTT--C

ACTGTGCCAATGACACCATTAT--CCCAA--tgttac-aga--ctgaatg

ttta---tgtg---------------------------------cctcct

--aaattc-acaggttgaat-tcctaa-cccccatatgatgg-tatttgc

a--tgtgggacctttaggaag-taattaggtctagtttagatcatgaggg

atagaaccct-------------catgatagggttca---agtgctta--

-gaagaggtagacaga-----------------------------ttctc

tg----------tgcaaaacaaaggaaaggccttataagcccaaagcaag

aaggcagcaatctaca-gccagaactataaaaaaataaatgtttgctgtt

taagccacaaggtctatggtattttgttatagcttccccagctgactaag

cca---CCTAA---TAACAAATGTAATAATCATAAAAATCAAAATTAATA

AACTGTAGTCACACAC

>rhesusmonkey

TCTAAT-AACATTTTTAATTTTGGTAAGGG-TGGGA-GGACACTGTT--C

ATTACGGCAATTATCCTACTCT--CCCCA--tgttat-gga--ctgaatg

tg----------------cctccccc------------------------

--aaattc-acaccctgaag-ccttaacc-cctaatgtgatggtatttgg

a-ggtgag-gcatttgggagg-taattaggtttagattaagtca-tgagg

gtgggac---------cca----catgatggggttaa---cgctcttata

agaagaggtagacagatctctctctctttctgttctctcttg------tc

tctctctctctctgag-----------------------caaacagcaag

agggcggcagtcagca--gccagaactgtgagaaataaatgtctattact

taagccacccagtctatggtattttgttatagcagctccagctgactaag

ata---CCTGG---TAATAAATTTAATAATCATAAAAATCAAAATTAATA

AACA--AGTTATAAAC

>human

TCTAAT-AACATTTTTAATTTTGGTAAGGG-TGGGATGGACACTGTT--C

ACTATGGCAATTATCCTTCTCT--CCCTG--tgttat-gga--ttgaatg

tttg----------ca--cctccccc------------------------

--aaattc-ataccttgaag-ccttaacc-cctaatgtgatggtatttgg

a-ggtgag-gcatttgggagg-taattaggtttagattaagtca-tgagg

gtgggac---------cca----catgatggggttaa---tgctcttatg

agaagaggtagacagatctctctctctctttctttttttttt----tctc

tctctctctctctgagcaa-----------------------acagcaag

agggcggcagtctgca--gccagaactgtgagaaataaatgtctattact

taagccacccagtctatggtattttgttatagcagctccagctgactaag

ata---CCTGG---TAATGAATGTAATAATCATAAAAATCAAAATTAATA

AACA--AGTTGCACAT

>chimpanzee

TCTAAT-AACATTTTTAATTTTGGTAAGGG-TGGCATGGACACTGTT--C

ACTATGGCAATTATCCTTCTCT--CCCTG--tgttat-gga--ttgaatg

tttg----------ca--cctccccc------------------------

--aaattc-ataccttaaag-ccttaacc-cttaatgtgatggtatttgg

a-ggtgag-gcatttgggagg-taattaggtttagattaagtca-tgagg

atgggac---------cca----catgatggggttaa---tgctcttatg

agaagaggtagacagatctctctctctctttctttttttttttttctctc

tctctctctctctgagcaaa-----------------------cagcaag

agggcggcagtctgca--gccagaactgtgagaaataaatgtctattact

taagccacccagtctatggtattctgttatagcagctccagctgactaag

ata---CCTGG---TAACGAATGTAATAATCATAAAAATCAAAATTAATA

AACA--AGTTGCACAT

>treeshrew

GCGGTT-AA-ATGTTTAATCGGGAGAGGCG-TGGGGGAAACCCTGTTCCC

ATAAGGACAGGTACCCAACTCT--CTCTG--atatataagacaccaaatg

ttttg--g-----------------------------------aaagtcc

ccaaatttacccaattgaaacccctaactccacaccccgatggtttttgg

aaagtaggttcctttgagaagtaattgagttttagatta-ggtaatgggc

atgggac------tctccatgattgagttaatatcatcacaataagaggg

tagacagagaaaca-ttcccctctctctctctctctctctctctctctct

ctctctctctctctgtcttcca----------tgtgcacaccccaataag

aagggcactatctaca-gccaggactataaggactatgtagtctgttatt

taagtcacccagtctatggtattttgttacagcaaccctagcttactaag

aca---TTTAG---TAGCAAGTGTAATAATAATAAAAATCAAAATTAATA

AACCGTAGTTGCACAC

>elephant

GCTTTAATAGCATTTTTAGTGTGGGCGGGGGAGG-ACGGACAGTGC---C

ACTACAGCAGTTACCCTGCACT----------------------------

--------------------------------------------------

--------------------------------------------------

--------------------------------------------------

--------------------------------------------------

--------------------------------------------------

--------------------------------------------------

--------------------------------------------------

--------------------------------------------------

------CCCAG---TAACAAATGTAATAATCACAAAAGACAAAATTAATA

AACTGTAGTCACAAAC

>sloth

CACTAATAACA-TTTTTAGTTTGGGTTGGGGTG--GGATGGACTATT--C

GCTACGGCAATTACCCTAGTCT----------------------------

--------------------------------------------------

--------------------------------------------------

--------------------------------------------------

--------------------------------------------------

--------------------------------------------------

--------------------------------------------------

--------------------------------------------------

--------------------------------------------------

------CCTGG---TAACAAACATAATAATCATAAAAATCAAAATTAATA

AAGTGTAGTTGCAAAC

Marker 5e:

>MLT1A0

--------------------------------------------------

--------------------------------------------------

-----------------------------------------tgctatggt

ctgaatgtttgtgtccccccaaaattcatatgttgaaacctaatccccaa

tgtgatggtatta---ggaggt-ggggcctttgggaggtgattaggtcat

gagggcggagccctcatgaatgg--ga------ttagtgcccttataaaa

gagaccycagagagctcccttg-ccccttccgccatgtgaggacacagtg

agaaggcgccgtctatgaaccaggaaatgggccctcaccagacactga-a

tctgccggcgccttgatcttggacttcccagcctccagaactgtgagaaa

taaatttctgttgtttataagctacccagtctatggtattttgttatagc

agcccgaacagac----taaga-ca-------------------------

--------------------------------------------------

-----------------------------

>cow

CCAAAACTTTTCCAGGTCCATCAAAAGGCAAAAAGTCCCCATGTTCTAAG

AGAGAGAGAGAAAAAA---TTATTGGA-TGATCAATAATTAGGATCTCTT

T-GCAATCTTTTT-CTAATGAAA---------------------------

--------------------------------------------------

--------------------------------------------------

--------------------------------------------------

--------------------------------------------------

--------------------------------------------------

--------------------------------------------------

--------------------------------------------------

------------------CAACA-----C--CAGATAAAATTTTGTGTGG

GTTTTTTCCCCCTTGTTTCTGTTTTTTTTTACCTCTAATTGCAAATATGA

TCATTATGTCAGCTTCTCCTTCATAGATC

>dog

CCAGAGCACGCCCTCATCCATCAAAAGGGTTAAAGTCTCCATGATCTAAC

AGAGAAAATGTTT------TTATTGGAAATATAGGTAATTAGGATCTCTT

TGGAACATCCCT--CACCCTTCCGCT-CCC--------------------

--------------------------------------------------

--------------------------------------------------

--------------------------------------------------

--------------------------------------------------

--------------------------------------------------

--------------------------------------------------

--------------------------------------------------

-----------------TCAACAA----CGGCAGCTAAA--TGGATGCGT

TTC--ATGTTTTATTTGTTTTTGTTCCTCTACCTCTAACTGCAAAAGAAA

TCTTTATGTCAGCCTCTCCTTCATCAGTC

>mouse

CCAGACTCTGTCCTGGCCCATCAAAAGGGTAAAAGTCTCCGTGTTCTGAT

GGTAAA--------CAAATGTACAG-AAATGTAAGTATT-AGAATTTCCT

C-CAATCCCTTTT-CAGTAAGAGAGC--TC---AGGGG---ctgtatggt

ttgacagtttatttctcttg-aaatctagatgt--------aatcttcag

tgtgatgggaatgctaggcagccatcacctttagaatacgataagggtgt

gagt-------cttcatggatgcttagtgtccattagtgtctatgagaaa

gaag-ttggaggcacctcttgg-ctgcttttgccataggaggtaaca-ca

gg-----------tgctacctaagaa-------cacaccag--attga-g

ttttcccacccccttattttatatttttcagattcaggaagtc-tgagtg

atacctttgtgttgtttacagatcttccaatgtgagatattttgtttgag

tagcctgaataact----CAAA------CACCAGA---------------

------------TGAGAG--------CATTACCTCCAACTGCAAAGGAGA

TCATTATGTCAGCCTCTCCTTCAGAGATC

>rat

CCAAGCTCTGTCCCACTCCATCAAAAGGGTAAAAGTCTCCATGTTCTGAT

GGAAAA---------AAATGTACAG-AAATGTAAGTATT-AGAATTTCCT

C-TAGCCCCTTTT-CAGAAAGAGAGC--AG---AGGGG---ctgtgtggc

ttgactattgatttctctca-aactccagatgtctaaatgtaatcttctg

tatgacaggaatgctaggcagccatggtctttagaagatggccagggtgt

gagt-------ctctgtggatg----------ctcagtgcctaggggaaa

gaag-ttggaggggggccctcgtctgcttttgccacaggaggtaata-ta

gg-----------tgccacctaagaa-------cacaccag--ccgga-g

tctgaccaccccgttattttat-tttctcaggttctggaagtc-tgaacg

atacatttgtgttgtttacagatttcccaatgtgaggtattttgtttgag

tggcctgaacaacc----CAAA------TATCAGAT--------------

---------GAATGACTATACATCTGCATTACCTCCAACTGCAAAGGAGA

TCATTATGTCAGCCTCTCCTTCAGAGATC

>guineapig

CCAAACTCATTCCTGGTCCATCAAAAGAATAAAAATCTCCATGTTCTGGG

GAGAAAAT------CAATGGTAGCATAAA------TGTTCAGAATTTCTT

TTTATCCTTTT--CCAGTCACACAGC--AT---CAGAC---gctattgtc

tgaacgttaacactttctca-aagtctgtatgttgaaagttagtattaaa

aggtagaacctgc-----------gtattgtta------ttatttccatg

atgt-------ccccatggatg--------agagcaatatccttatataa

gagg-ctgagggaacctctgga-ccctt-ctgccacatgaggacaccgaa

tgc-----------accacgatgaagaatgcaccacattaacaaagattt

tctg---accccaggacctttgacttctcagtctcagaactat-gaaaac

tatcatttctgttgttcacaaattaaccagtgtaaggtattttgttttaa

taacctgagctgac----AAGAA-----CATTTGAT--------------

---------AAATGGATATGTTATTTCATTACCTCTGACTGCAAAAGAGA

TCATTATGTCAGCCTCTCCTTCATATATT

>marmot

CCAAACTTTGTCCTGGTCCATCAAAAGGTTCAAAGTCTCCATGTGCTGGT

GGAAAAA-------CAAT--TAATGTAAATATGAGTATTCAGATTTTTAA

AAAAATTCATTTT--AGTAAGATAATACCA------A----tgcaatgat

ctgaatgttagtgtctcctcaaaatcca-tatgttgaaacaatttctggt

atgaaag----tattagaaa--tgggccctttaggaagtaatttggtctt

gagag-----------tttaatgaatggga---ttagtgtcccctataaa

agaggctggaggaagtctcttggcccctt-ctaccaagtgggaacacaga

at---------------------------agaccctcatcagacacacca

tctgctggcaatttgatcttggctttctcagcctccagaacgct-aaaca

atgaatttctgttgtttataaataacccaaccaaatgtattgttttttta

g--cctgaactgat----CATAA-----CACGAAAT--------------

---------GAATTGGTATTTTATTTCATTACCTCCAACTGCAAATGAGA

TCATTATGTCAGCCTCTCCTTCATGCATC

>rabbit

CCAGACTGTATCCTGGTCCATCGAAAGGATAAAAGTCTCCGTGTTCTGAC

AGGGGCAGA-------AAATTAATGGAAAT-TAAGTGTCTAGAATTTCTT

T--CTACTCTGA-CCAATAGGACAACACCC-----GAT---tccatggtc

tgaatgttgataaaacctcacaaatctccaaagtgaccgtattcagtgat

ggggacatcaggagg----------------tgggtaggc-------tcg

aatctcc-----------ataaatggga-----ttagtg-cccttacaag

agaggcttgagggaggcccctggctcctt-ctgccacgtgaggaggcaga

cgg----------tgccatctatgaggaaggtaccttcaccagacatcag

atctgctggcaccttgattttggacttcccagccttcagaactgtaagca

ataagttcctgctgtttataaattacccagtct---gatttttgggatag

aagcctgaggtgac----TAAGA-----CATAAAAA--------------

--------------TATAGTTTATTTCATTACCCTTAACTGCAAAAGAGA

TCATTATGTCAGCCTCTCCTTCGTGCTTC

>rhesusmonkey

CCAAGCTGTGTCCTGAGCCATCAAAAGAGTAAAAGTCTCCATGTTCTGAT

AGGAAAAAAAAA------TTAATGG-AAATATAAGGGTTCAGAATTTATT

T--TTACCCTGTTCCAATAAAACAA---CA---GTAGA---tgctatagt

ctgaatgctggtgtctactgcaaattcatatgttgaaactccatattcac

tgtgat-atagtattaagaggttag-gtctttaggaagtgattaggtcat

aggtgctctgccctcatgaatg---ag-----attagtgctcttacgaaa

gagg-cttgagggaacctcctgg-ccctttctattgtgtgaggacacaga

aggt---------accatctatgaagaataggttctcatcagacaccaa-

atctgctgacaccttaatcttggacttcccagcctccaggattgtgagca

ataagtttctgttgtttataaattacacggtccaaggtatctggctctag

cagtctaaacagat----TAATA-----CACCAGAT--------------

---------AAATGGATGCTTTATTTGATTACCTTTAACAGCAAAAGAGA

TCATTATATCAGCCTCTCCTTCATACAGC

>human

CCAAACTGTGTCCTGGGCCATCAAAAGAGTAAAAGTCTCCATGTTCTGAT

AGGGGATAGGGAA-CAAATTAATGG-AAATATAAGTGTTCAGAATTTCTT

T--TTACCCTGTTCCAATTAAACAA---CA---GTAGA---ttctatagt

ctgaatgttggtgtctgctgcaaattcatatgttgaaactcaatgcccaa

tgaaat-atagtattaagaggttag-gtctttaggaagtgattatgtcgt

aagtgctctgccctcatgaatg---ag-----attagtgcttttatgaaa

gaag-cttgagggaacctcctgg-ccctttctatcgtgtgaggacacaga

aggt---------accatctatgaggaataggctctcatcagacactaa-

atctgctggcaccttaatcttggacttcccagcctccagaattgtgagca

ataaatttctgttgtttataaattacccagtctaaggtaacttgctatag

cagtctgaacagat----GAATA-----CACCAGAT--------------

---------AAATGGATGCTTTATTTGATTACCTTTAACTGCAAAAGAGA

TCATTATATCAGCCTCTCCTTCATACAGC

>chimpanzee

CCAAACTGTGTCCTGGGCCATCAAAAGAGTAAAAGTCTCCATGTTCTGAT

AGGGAA--------CAAATTAATGG-AAATATAAGTGTTCAGAATTTCTT

T--TTACCCTGTTCCAATAAAACAA---CA---GTAGA---ttctatagt

ctgaatgttggtgtctgctgcaaattcatatgttgaaactcaatgcccaa

tgagat-atagtattaagaggttag-gtctttaggaagtgattatgttgt

aagtgctctgccctcatgaatg---ag-----attagtgcttttatgaaa

gaag-cttgagggaacctcctgg-ccctttctatcgtgtgaggacacaga

agg---------taccatctatgaggaataggctctcatcagacactaa-

atctgctggcaccttaatcttggacttcccagcatccagaattgtgagca

ataaatttctgttgtttataaattacccagtctaaggtatcttgctatag

cagtctgaacagac----GAATA-----CATCAGAT--------------

---------AAATGGATGCTTTATTTGATTACCTTTAACTGCAAAAGAGA

TCATTATATCAGCCTCTCCTTCATATAGC

>flyinglemur

-----------CCTGGTCCATCAAAAGGGTAAAAGTCTCCATGCTCTGAC

AGAGAAAAAAA--------TAATGC-AAATAGAAGTTTTCAGAATTTCTT

T---TACTCTTTTCCAATAAGACAAC--AC----CAGA---tgc------

-ga----tggtc-------taaatg------ttggtgtctcc--cccaaa

--------agc-tgaaac--ttaa--tacccaattcacatagtat---ta

agagat-gcagattttaggagg---tg-----attaaaac-tttaa---g

agga--tt-agtgcccttcctgg-ccc-ttccactttgttaggacacaga

-ggtt--------gtcatctct--gaggtaggctcttaccatagactga-

atctgccaacactttgatcttggacttcctaacctccagaactatcagca

atatatttttgctgtttataaattacccagtataaggtattttggcttag

cagcctgaaaggac----TAAAG-----CACCAGAT--------------

---------AAATGGATATTTTATTTTGTTACCTTTAACTGCAAAAGAGA

TCATTATGTCAGCCTCTCCTTCATACATC

>treeshrew

CCAAACTGGATCCTGGCCCGTCAAAAGGGAAAAAGTCCCTGTGTTCTAAT

AGAGAGCAAAATAAATAAATAACAG-AAATAGAAGCATTCAGAATTTCTT

T--TTACCCTTGTCTAATAAGGTAAT-ACC-----AGT---ttctatggt

ctgaatattggtgttaccccctaattcatctgctggaa--caatctccag

tgcag---tagtagt-ataggtggggg-ctttaggaggttattaaatcat

aaaagctgttccccta--aatg---gg-----attagtgctcttagaaat

gaga-cttgaagaagcctcctct-at----tcttcttataaggacacaga

agata--------accatctataacaaacaggctct-------cacggg-

acattgactctgccgacatctggatctcccaggctctaggacaacgaata

ccgaacttctgttgtttgtaaattatccagactaagatattttgtt----

-acacaaccaggac----TAGGA-----CACCAGAT--------------

---------AAGCAGATATTTTATT-----ACCTTTAACTACAAAAGAGA

TCATGATG---------------------

Marker 5f:

>L1MC1

--------------------------------------------------

--------------------------------------------------

--------------------------------------------------

--------------------------------------------------

--------------------------------------------------

--------------------------------------------------

--------------------------------------------------

--------------------------------------------------

----------------------aaaaag--aaatgagctatc--------

aagccatgaaaagacatggaggaaacttaaatgcatattactaagtgaaa

gaagccaatctgaaaaggctacatactgtatgattccaactatat--gac

attctggaaa-aggcaaaactatggagacagtaaaaagatcagtggttgc

caggggtt---gggggggagggagggatgaacaggtggagcacagaggat

ttttagggcagtgaaactactctgtatgatactataatggtggatacatg

tcattatacat-ttgt-ccaaacccatagaatgtacaacaccaagagtga

accctaatgtaaactat-ggactttgggtgataatgatgtgtcaa-----

tgtaggttcatcgattgtaacaaatgtaccactctggtggggg----atg

ttgatagtgggggaggctgtgcatgtgtgggggcagggggtatatgggaa

atctctgtaccttccgctcaattttgctgtgaacctaaaactgctctaaa

aaataaagtc-tatttaaaaa-----------------------------

--------------------------------------------------

--------------------------------------------------

---------------------------

>cow

TTCTAAAAACTTCAAATCCATTCTCAGGAACCACCTTAAATGAAAGGCCA

G--GGTAGCTGTTA-GGCTCTCCCTTTATACACTATTCTCAGGATATTGA

CTCAGCGGGAAAGACAAG------GCGTATCTCTAAATCACTCTTCTGTT

TCACTAGCAGGGCCTTTGATAATTCTCC---CAAGAGAAGGTGAT-----

------TCTAGGAGCACCTAGTAGGAAGG--TGTAATACAGCTC------

--------------------------------------------------

--------------------------------------------------

-----------ACCACCTTGC--TATTAATA------TCTTCAGCCTC-T

TTTCCC--------------------------------------------

--------------------------------------------------

--------------------------------------------------

--------------------------------------------------

--------------------------------------------------

--------------------------------------------------

--------------------------------------------------

--------------------------------------------------

--------------------------------------------------

--------------------------------------------------

--------------------------------------------------

------------------------------AGAGAACTC---------AA

AAAGCAACTAA--AGC----------TCACCTCGCCTTTGATCAAATCCC

GATGTCGTTGGCAATACACTTTTTTATCATCCAGAAACACACAGTTCTTG

GCTCGGGAACACATGAAGTGATAGTTG

>dog

TTCTAAAAACTTCAAATCCATTCTCAGGAACCACCTGAAATGAAAACCCA

A----CTGTTAATA-GGCTGCCCCTTTACATGTTACACGCATGATACTGA

TTTTCTTGGAG-GGCATGTACGGAGTGTATCTCCAAATCACCCAGCTATT

TCA--TAGGAGGACTGCCAT--ATTT-----CT-AAGAAGGTGAT-----

------TCTAGGAACACACAGTAGGGAGG--TAAAACAAAGCTT------

--------------------------------------------------

--------------------------------------------------

-----------ACCATCTTGC--TGTTTTCAGCCTGAACTGAACTCCTGA

ACTCCT--------------------------------------------

--------------------------------------------------

--------------------------------------------------

--------------------------------------------------

--------------------------------------------------

--------------------------------------------------

--------------------------------------------------

--------------------------------------------------

--------------------------------------------------

--------------------------------------------------

--------------------------------------------------

------------------------------ATAGAATTT---------GA

AAAGCAGCAGA--AGC--------TCTCACCTCGCCTTTGATCAAATCCC

GATGTCGTTGGCAATACACCTTCTTATCATCCAGAAAGACACAGTTCTTG

GCTCGAGAACACATGAAGTGGTAGTTG

>bat4

TTCTGAAGACTTCAAATCCATTCTCGGGAACCACCTAAAATGAAACCCCA

GAGGAGCCGTTACTCGGCTTTCCCTTCGCAGGTTATGCTCACAATACTGC

CTCTCT-GAGGGGCAGGGCACA--GC---TCT-GGAATCACCTCTTCC--

-ACCAGGAGAG-----TGCCTGCA-TCCCGCTAAGAGGAGCAGGT-----

------GCTAGGGACAGCTACAGAAGTAA---AGCCAAATTACG------

--------------------------------------------------

--------------------------------------------------

-----------ACTTGCTTGC--TATTTT---CCATCTCTTCT-CCCTGA

ATTCCC--------------------------------------------

--------------------------------------------------

--------------------------------------------------

--------------------------------------------------

--------------------------------------------------

--------------------------------------------------

--------------------------------------------------

--------------------------------------------------

--------------------------------------------------

--------------------------------------------------

--------------------------------------------------

------------------------------GGAGAATTC---------AA

GAAGCAACGCAA-AGC------TCTCTCACCTCGCCTTTGATCATATCGC

GGTGCCGCTGGCAGTACACCTTCTTATCGTCCAGGAAGACACAGTTCTTG

GCCCGGGAGCACATGAAGTGGTAGTTG

>shrew

TTCTGAAA-CTTCAAATCCATTCTCGGGATCACTGAAGTGCAAGCCCGCA

TA-----GTTATTATAGCCTGTCTCGTAGCACTCACTGCCCTGAGGAAGT

G---TTGAGTGATCTCAGCCCCCTTTAGCTTCATAACAGCACCCCCCACT

GCTGCTAGGAGGGG------------------------------------

-CAAC-TCTA-GGGAAATCCAACGGGAAG-GCAAAGCAGAGTCT------

--------------------------------------------------

--------------------------------------------------

-----------GCCATCT----------------GTTTTCCAGGGTTTTT

TTCCTG--------------------------------------------

--------------------------------------------------

--------------------------------------------------

--------------------------------------------------

--------------------------------------------------

--------------------------------------------------

--------------------------------------------------

--------------------------------------------------

--------------------------------------------------

--------------------------------------------------

--------------------------------------------------

-------------------------------AAGTTTTA---------GA

GAAGTTGAAAAACAGCTTAAAGC-TCTTACCTCGCCTTTGATCAAATCCC

GATGTCGCTGACAATATACTTTTTTATCATCCAGAAAGACGCAGTTCTTG

GCTCGAGAACACATGAAGTGATAGTTG

>hedgehog

TCCTAAAAACTTCAAATCCATTCTCAGGAACCACCTTAAACGAAAACCCA

GGGTAGCTGTTAATAGGCC-----TTATCCCTACACATTAATCTTATGAT

ACTGACTGTCTCAGAGGGAAAATACAGAGCGTATTTCAAAATCACCCACC

ACCCTTCTGTTTCACTAGTGGTCTTTCTCATAAAAGGTGAG---------

------TCTGGGAAAATCTAACAGGAAA--GAAAAGTAAGACTG------

--------------------------------------------------

--------------------------------------------------

-----------ACCACACTA------------CCAA-TTTCAACCTCTTT

CCCCCG--------------------------------------------

--------------------------------------------------

--------------------------------------------------

--------------------------------------------------

--------------------------------------------------

--------------------------------------------------

--------------------------------------------------

--------------------------------------------------

--------------------------------------------------

--------------------------------------------------

--------------------------------------------------

---------------------------------AATTTC-------TAGA

AAATTTAAAAAACAGCTAAAGCTCTCTTACCTCGCCTTTGATCAAATCCC

GATGTCGCTGGCAATATACTTTTTTATCATCCAGAAAAACACAGTTCTTG

GCTCGGGAACACATGAAGTGATAGTTG

>mouse

TTCTAAAAACTTCAAATCCATTCTCAGGAACCACCTTAAATGAA-CCCAA

GGCTGCGGTTAGTG-GACTGTTCCTTTACACAGATACCAACCTCT-----

--GCAGACAAAGCAGAGG------GCCCATCTTAAAATCACCCTCCTGTT

TCATT------ACCACCCTTCGTTTCTT-AAAACAGGAGATGATG-----

------TCTTCATACATC--TGAAAGAGG--CAAAACGGCATTT------

--------------------------------------------------

--------------------------------------------------

-----------ACCATCCTGC--TATTTT---TAGTATTTCCC-TCCTAA

ATT-CT---TAGAGATTTC---aaaag--aggaggaatctt---cagacc

ttcttgcaaagcagaaaggctactaaccgcaagattccaaccacaa-gaa

accccgcaagtagaaaactgcttggatgtagggtaaaagtacttg-taga

tattagcaa-caataataataaaataaaatttaaaa---tcag----tgt

tttttta----gggggaaag----agaaaaaacaggcagaacaaagaatt

cttaaaa-aatgaaaatact--taaatgga-ctacagtgatgaacacgca

gtattaaacat-ttgt-ctgaacccacaggatgtagaacgtcaaaagtga

accctgatataaaagct--gactctggggaaatgcgtatgtgtccatatt

cacgtagaccatctataccgcaaatacaccactctagtgggagaggctat

gttagtgtggaggcaa-------------------------ggccagtaa

atcactgtgccttttgct-aattttactgtgaacttaagtctgctctcag

-ataatagagcctgttatta----------ATAGC-TTC-------AACA

GAAGCAACCAAACAGC--TTAGCTTCTCACCTCGCCTTTGATCAAATCCC

GATGCCGCTGACAATACACTTTTTTATCATCCAGGAAGACACAGTTCTTG

GCCCGGGAACACATGAAATGGTAGTTG

>rat

TTCTGAAGACTTCAAATCCATTCTCAGGGACCACCTTAAATGAGAG-CCA

AGGTTGCCATCAGT-GACTGTCCCTTTACACATTACTTTCA-GAT-----

--TCCAACC-TCTGGAG-------GCAAATCTTAAAATCACCCTTCTGTT

TTATC------ACCACCCTTTGCTTCTC-AAAACAGGAGATGATG-----

------------------------------------TATTCAGA-catct

tttttttttctttttcctttttttcggagctggggaccgaacccagggcc

ttgtgcttgcgctctaccactgagctaaatccccaaccctgtattcaggc

atcttaaagagACAAAACTGC--TATTTTTT-CAGTATTTTCC-TCCTAC

ATTTCT---AAGAGATTCC---aagggtgaggaagaatctt---caatgc

ttattgctaagtggaaaggctacgaactgcaagattccaaccaaaa-gaa

actctggaagtagaag-ctgcttagaggaaaggtaaaagtacttg-taga

tattagc---caaaaaaaataaaataaaataaaatttaaaaaaaa-t--c

agtgggttttagggggaaaagcaggaaaaaagagccagaaaaca-gaatt

cttaggacaatgaaaatactaatttgaa---cta--gtgatgtatacaca

gtatcagacat-ttat-ctaaacccaccc---------------------

--------------------------------------------------

--------------------------------------------------

--------------------------------------------------

------------tttggctaattttactgcgaacttaagtctgctctgag

-ataataaagcctgttataa----------ACAAC-TTC-------AACA

GAAGCAACCAAACAGC--TGAGCTTCTCACCTCGCCTTTGATCAAATCCC

GATGCCGCTGACAGTACACTTTTTTATCGTCCAGAAAGACACAGTTCTTG

GCACGGGAACACATGAAATGGTAGTTG

>marmot

TTCTAAAAACTTCAAATCCATTCTCAGGAACCACCTTAAATAAAAACCCA

AGATTGCTGTTAATAGATTGTCTCTTTACACAGTATATTCATGGTACTGG

CTCTCTCT----------------GAGCATCTCAAAATCACCTTTCAATC

TTACT-AGAAGCACC--TCTTTATTTTT---CATAAATAGGTGAC-----

------CCTTGGAATGCCTAGTAGGAAAAG-CAAAACTGAATTT------

--------------------------------------------------

--------------------------------------------------

-----------ACCATTTTGC--TATTTTTTTTTTAGTATTTTTTCCTAA

ATTCCT---GAAGAATTC----aaaaagctactataagcca---------

------taaaaagatgtggaagaaacttaaaggtatattgtgaatt-gga

ataaactggtctgaaaaagctacatactatagcattccaactatatagga

agctctggaaatggcaaattcatggagatagtaaaaagatcagtggttgc

caagggtt------------------ggtggtgttgacaggcagaacaca

ttctagggcagtaaaaaca-tttgcttgatattatagtggtgaatacctg

ttatcatacat-ttgc-ctaaacccatagaatgtacactaccaagagtga

acccaaggtataaagtatagtcgctgagtggtaatgatgtattga----t

a-gaggttcatcaattgcaacaaatagacaactctggaggggg---acac

aattaatgagggaggctgcatattgtgtagaggcagggagtatataggaa

atctctgtaccttctgcttaattttgcagtgaacctaacactgctttaaa

taaat-aaagtcaaacat------------ATA-CATA------------

AAAGCAACTAA--AGC------TATCTCACCTCGCCTTTGATCAAATCCC

GATGTCGTTGGCAATACACTTTTTTATCATCCAGGAAGACACAGTTCTTG

GCTCGGGAACACATGAAATGATAGTTG

>rabbit

TTCTAAAAACTTCAAATCCATTCTCAGGAACCACCTTAAATAAAAACCCA

AGACAGCTGTCAACAGGCTGTCGCTTTGTACTTTATGTTCACGGTACTGG

CTCTC--AGAGGAAAA-GCACAAGGCATATCTTAAAATTACCATTCTGTT

TTAATTTCTCG--------------------CTAGAGGAAGTGAT-----

------TCTAGGGACACCTAGTAGGAAGG--CAAAACACAGTTT------

--------------------------------------------------

--------------------------------------------------

-----------ACCATCCTGC--TATTATT--CAGTCTTTTGTCTCCTAA

ATTCCC---AGAAAACTC----aaaaggctaataaacaaagcat------

------ggaaagacttggaggtaaacgtaaa-tgtgtatttctgggtagg

cgaagcaagtccaaaaagactatgtactgtatgacttcaagtactcagaa

a-tcttggaggtggcaagactgtgaagacagcaacgagaccagcagttgc

ccagggttggggagaatgaatgggtagaacaccgagagatgccttctgtc

cacaccgaggacatccagggcaatgaaaatactcataaggatgctgccat

ggcggacacaggctgttacacaacacaacaatacacaaccgcaagagtga

accctaa-tctacaccatagactctggtga-taaggatgcaccaa-----

tgt-ggttgttacca-g-aaggggatgctgatatgggggaagg---ttgt

gtacatgctggggaggggggtaaat-------------------gggaaa

tctctataccttccctcttaattttgctgtgaacctaaaattgctctaaa

aaaattaaagtctattat------------TAAGGGA------AAAAAAA

AAGACAGCTAA--AGC------TCTCTTACCTCGCCTTTGATCAGATCCC

GATGTCGTTGGCAATACACTTTTTTATCATCCAGAAAGACACAGTTCTTG

GCTCGGGAGCACATGAAGTGATAGTTG

>human

TTCTGAAAACTTCAAATCCATTCTCAGGAACCACCTTAAATAAAACCCAT

GGTAGCTGTTAATA-GGCTGTCCCTTTGCACATTATACTTATGATCCTGA

CTGTCTCAGAGTGGAAGGCACATAGCATATCTCAAAAGCATCCTTCTGTT

TCACTTAGCAGGACACTTCTGTATTTCC---CAAAAGAGGTGGTG-----

----CCTCTAGGAACACCAAGTAGAAAAG--CAAAACAG--TTT------

--------------------------------------------------

--------------------------------------------------

-----------ATCATCTTGCTGTATTTTT----GT-TTTT--CTTTTGC

ATTTCG---AAATAATTC----aaaaag--caactaaatt----------

aagccatgaaaagacttgaaggaaacttaaa-tgc-------tatgcaga

agaagccagtctgaaaaggctatgtactatatgattccaactatgt-gaa

actctgcaaa-tggcaagactatggagatagtaaaaacatcagtggttgc

ccagggtt---ggggag-aggcagggatgaataggcagagcacagaggat

gtttaaggcagcgaaaatactccatatgatggtataatggtggatataca

tcattatacat-ttgt-ccaaacccacagaatgtacaacaccaagagtga

atgctgatgtaaactacaggagtcagggtgataaccatgtgtcca----g

tgtaggtttatccactgtaacaaatgcaccactctggtggggg----atg

ctgatcatgggggaggctgtgcatatgtgggtgcagggggcatatgggaa

atctctgtaccttccagtcaattttgctgtgaacctaaaactgctctaaa

aaa------gtcttttttttttttttt-----------------------

AAAGCAACTAA--AGC------TCTCTCACTTCGCCTTTGATCAAATCCC

GATGTCGTTGGCAATATACTTTTTTATCATCCAGAAAGACACAGTTCTTG

GCTCGGGAACACATGAAGTGATAGTTG

>chimpanzee

TTCTGAAAACTTCAAATCCATTCTCAGGAACCACCTTAAATAAAACCCAT

GGTAGCTGTTAATA-GGCTGTCCCTTTGCACATTATACTCATGATCCTGA

CTGTCTCAGAGTGGAAGGCACATAGCATATCTCAAAAGCATCCTTCTGTT

TCACTTAGCAGGACACTTCTGTATTTCC---CAAAAGAGGTGGTG-----

----CCTCTAGGAACACCAAGTAGAAAAG--CAAAACAG--TTT------

--------------------------------------------------

--------------------------------------------------

-----------ATCATCTTGCTGTATTTTT----GT-TTTT--CTTTTGC

ATTTCG---AAATAATTC----aaaaag--caactaaat-----------

aagccatgaaaagacttgaaggaaacttaaa-tgc-------taagcaga

agaagccagtctgaaaaggctatgtactatatgattccaactatgt-gaa

-ctctgcaaa-tggcaagactatggagatagtaaaaacatcagtggttgc

ccagggtt---ggggag-aggcagggatgaataggcagagcacagaggat

gtttaaggcagtgaaaatactccatatgatgctataatggtggatatatg

tcattatatat-ttgt-ccaaacccacagaatgtacaacaacaagagtga

atgctgatgtaaactacaggagtcagggtgataaccatgtgtcca----g

tgtaggtttatccattgtaacaaatgcaccactctggtggggg----atg

ctgatcatgggggaggctgtgcatatgtgggtgcaggaggaatatgggaa

atctctgtaccttccagtcaattttgctgtgaacctaaaactgctctaaa

aaa--ta--gtcttttttttttttttt-----------------------

AAAGCAACTAA--AGC------TCTCTCACTTCGCCTTTGATCAAATCCC

GATGTCGTTGGCAATATACTTTTTTATCATCCAGAAAGACACAGTTCTTG

GCTCGGGAACACATGAAGTGATAGTTG

>flyinglemur

TTCTAAAAACTTCAAATCCATTCTCAGGAACCACCTTAAATAAAAGCCCA

GGGTAGCTGTTAACAGGCTGTCTCTTTTCACACTATACTCATGATACTGA

CTCTCTCACAGGCCAAG-TACAGAGCTTAAATCAAAATCAGCCTTCTGTT

TTACTAGC-AGGAC--TTCTGTATTCCT---CATAAGAGGAAATG-----

----ACTCTAGGAATACCTAGTAGGAAGG--CAAAATAG--TTT------

--------------------------------------------------

--------------------------------------------------

-----------ACCATCTTGC--TATTTTT--CAGTCTTTTT-TCCCTGA

ATTCTT---AGAGAATTC----ataaag--caactaaat-----------

aagccatgaaaagacttggagaaaacttaaa-tgcatattgctaagtaga

agaagccagtctgaaaaggcatactac---acgattggaactatat-gaa

aatctggaaa-tggcgagactatggagacagtagaaagatcagtggttgg

ccagggttaaaggggag---atagggatgaacaggcagagcacagagcat

tttttgggcagggacaatactctacatgatactataatgatggatacatg

tcattatacat-ttgt-ccaaacccacagaatgtacaccaccaagagtaa

at-ct--------------------------------------------t

agtaggttcatcaattgtaacaaacataccactccagtggggg----atg

ctaataatgggggaggctgtgcctatgtgagggcagggggtatatgggaa

atctctgtgccttct-gctaattttgctgtaaacctaaaactgctcttaa

aag--taaagtctatta-------------TTAAAAG--------CAAAA

TAGGCAACTAA--ATC------TCTCTCACCTCGCCTTTGATCAAATCCC

GATGCCGTTGGCAATACACTTTTTTATCATCTAGAAAAACACAGTTCTTG

GCTCGGGAACACATAAAATGATAGTTG

>elephant

TTCTAAAAACTTCAAATCCGTTCTCAGGAACCACCTTAAATGAAAATCCA

GAGTAGCTGTTAATACGCTATCTCTTTACACATTACATTCATCACATCGA

TCCTCTCAGGGGAAAAGTATAAAACGTATCTCAAAACCACCATTCTGCTT

TACCAGCAGGACTTCCGTATTTCTTG----TAAAAGGACGTGAC------

------TCTAGCAATAGCTAACAGGGAGG--CAAAACTAAGTTT------

--------------------------------------------------

--------------------------------------------------

-----------ACCATCTTGC--TATTTT---CCATCTATTTG-CCCTAA

CCTCCT--------------------------------------------

--------------------------------------------------

--------------------------------------------------

--------------------------------------------------

--------------------------------------------------

--------------------------------------------------

--------------------------------------------------

--------------------------------------------------

--------------------------------------------------

--------------------------------------------------

--------------------------------------------------

------------------------------AAGGAATTT---------AA

AAAGCAACTGA--AGC------TCTCTCACCTCTCCTTTGATCAAATCCC

GATGTCGTTGACAATACACCTTTTTATCATCTAGAAAGACGCAGTTCTTG

GCTCGGGAACACATGAAGTGATAGTTG

>opossum

TTCTAAGAACTTCAAATCCATTTTCAGGAACCACCTTAAATAAGAAATCA

GGGACATTATTAAAATGCT-AGTTTTAACTCACAGGATATGTTCACCATT

TAGTCTATTCCAAACCTAAAGA--GCAGAGTTAATTTAACACTGGTATTT

TCTCAGTAACTTCAATATGTCTAAT---CAGAAGAGAGGTTAAGG-CAAT

AAAAAAAGTCACAGGAACACTCTTTAGAAGGACAAATGAATTTT------

--------------------------------------------------

--------------------------------------------------

-----------CCCATCCTGC--CATTTTT--TAGTCAGCA------TTT

TTTCCC--------------------------------------------

--------------------------------------------------

--------------------------------------------------

--------------------------------------------------

--------------------------------------------------

--------------------------------------------------

--------------------------------------------------

--------------------------------------------------

--------------------------------------------------

--------------------------------------------------

--------------------------------------------------

------------------------------CAAGTCTCC---AGATTAAA

AAAGCAA-TCA--AAC--------ACTTACCTCTCCTTTTATCAAATCAC

GATGTCGCTGGCAATAAACTTTTTTATCATCCAGAAAGACACAATTCTTG

GCTCGGGAACACATGAAGTGATAGTTG

Marker 5g:

>MLTA10

--------------------------------------------------

---tgctatggtctgaa-------------------tgtttgtgt--ccc

cccaaaattcatatgttgaaa-cctaatccccaatgtgatggtattagga

ggtggggcctttgggaggtgattaggtcatgagggcggagccctcatgaa

tgggattagtgcccttataaaagagaccycagagag-ctcccttgcccct

tccgccatgtgaggacacagtgagaaggcg-ccgtctatgaaccaggaa-

atgggccctcaccagacactgaatctgccggcgccttgatcttggacttc

ccagcctccagaac--tgtgagaaataaatttctgttgtttataagctac

ccagtctatggtattttgttatagcagcccga---------ac-------

--------------------------------------------------

--------------------------------------------------

--------------------------------------------------

--------------------------------------------------

--------------------------------------------------

--------------------------------------------------

--------------------------------------------------

--------------------------------------------------

--------------------------------------------------

--------------------------------------------------

--------------------------------------------------

--------------------------------------------------

--

>cow

TGTGACATCTAGCCTGAGCCTGAAAGAAAAAGGAAATGCAT---------

--------------------------------------------------

--------------------------------------------------

--------------------------------------------------

--------------------------------------------------

--------------------------------------------------

--------------------------------------------------

--------------------------------------------------

--------------------------------------------------

-------GGCCACC------------------------------------

--------------------------------------------------

--------------------------------------------------

--------------------------------------------------

-------------------------------------AGAGAAAGAGAAC

ACACCACTGCCAGTTACAAGA--GCATCTGTTC--------TGATAAATA

G-GGGTACCATTCAACACTGGAGGG----------------------TTA

ACACGCC-AAGAAATGACTTTCACATTATCCATCAATTGATTACCGGTTG

GTTCATTTG-CCCCAATCATGGGACACTGCCTGAGCGACGGCAGGATTTC

AATTTATTGGTCTGATGTAGCGGAACGACTTCCT----------------

-----------AAAGTGGGGGAGGGGGGTACCAGGAAGGTTTTCTTGCAT

GTTTACCTGGAGATCATTTTGGGGATTCCAGTCAGAATCGAAGATGATGC

AG

>dog

TATGACATCTAGCCTGAGCCTGAAAGAAAAAGGAAATGAAT---------

--------------------------------------------------

--------------------------------------------------

--------------------------------------------------

--------------------------------------------------

--------------------------------------------------

--------------------------------------------------

--------------------------------------------------

--------------------------------------------------

-------GGTTACC------------------------------------

--------------------------------------------------

--------------------------------------------------

--------------------------------------------------

-------------------------------------AAAGAAAGAAAAG

ACTCAAGTGTCAGTTATAAGA--GCACCCGTTC--------CAATAAGCA

G-AGGTGCAGTAC------AGAAGG----------------------TTA

ACACACTAAGAAATTAC-TTTTACGTTACTAGTAAATTGATTACCACTTG

GTTCATTTG--CCCAACCATGGGATGCTACCTGAGTGACTACACAACTTT

AATTTATTGGTTTGACTGGGGATTCTTCATCATAATGGAATAACTTAAAA

AAAAGAAAAGAAAAGAAAAGAAAAAAGGCATCAGGACGATTTTTGTGTAT

CTTTACCTGGAGATCATTTTGGGGATTCCAGTCGGAATCAAAGATGATGC

AG

>bat4b

TGTGACACCTTGCCTGGGCCTAGAAGAAAAGGGGAACTGAT---------

--------------------------------------------------

--------------------------------------------------

--------------------------------------------------

--------------------------------------------------

--------------------------------------------------

--------------------------------------------------

--------------------------------------------------

--------------------------------------------------

-------GGCTACC------------------------------------

--------------------------------------------------

--------------------------------------------------

--------------------------------------------------

--------------------------------------AAGAAAGAAAGG

-CTCCGTCGTCAGTTATGAGA--GCATCTGCTGTT-----CCAATGAACA

G-AGGTGCGGCTTC---GAAGAGGG----------------------TTA

ACACACAAAGAAACTGC-TTTTACATTATTAATAAATTGATTACTAGTTG

GTTCATTTGCCCCCA-TCATGGGATACCGCCTGAGTGACTACATGACTTT

AATTTATTGGTTTGAC-GGGGATTCTCCGTCACAGCAGAATGGCTTAAAA

AAAAAAAA------------------GGCATCGGGAGATTCTCTTGCATG

TTTACCTGGAGGTCATTTTTGGGGATTCCAGTCAGAATCAAAGATGATGC

AG

>shrew

GGTGACGTCGAGCTTGGGCCTGGAAGAAAGGGGAAATGAAG---------

--------------------------------------------------

--------------------------------------------------

--------------------------------------------------

--------------------------------------------------

--------------------------------------------------

--------------------------------------------------

--------------------------------------------------

--------------------------------------------------

-------GGCCTTT------------------------------------

--------------------------------------------------

--------------------------------------------------

--------------------------------------------------

-----------------------------------CAAAGGGGAAAATAT

GCCCAACTGCTGGCAATAAGA--GCATTTGTAC--------CAATAAACA

GAGGTGTGGCTTAGGAGAGGGTTAG----------------------TTA

CCATAGT-AGGAAATTACTTTCGTATTATTAGCTAATCGATTACTAGCTG

GTTCATTTG--CCCCGTCCCGTGATATTGCCTGAGGGACTATAAGACTTT

AATTTATTGGATTTCACTA-GGGAATCTCCATC-----------------

AGAGTAGAAAGGTCATGACTTACTATCAGGAAGACTTTTTTTTTTTACAC

CTTTACCTGGAGATCCTTGTGGGGGTGCCAGCCGGAATCTGAGATGATGC

GG

>mouse

TGTGGCATCTAGCTTGAGCCTAGAAAGAAAAGG-AACCAAA---GGCTGC

---tgcaatggagcacaggactgtggagcacacacaggtctgtg-tcctc

cct--aattacgtgggttaaaagc-tgatctcaatgttag---atttaga

gatgaggactcagag--gtaattaggtcacgagggtggtgccctgatgaa

caggattagtg-tcctgtaa------------------------------

-----------gaagcagcaggaggaagacagctgtctatg---------

---------cagcaataacctaatcttgctggcatccagacctcacagtt

-caa-ctccagaaccttga----aaaaggcatggatattgtttacgctac

cccatc-atagcattctgttatggcagccaaagtgatca--actcaacaa

acat---GTCTCAC------------------------------------

----------cgagtgtggtggcgcacgcctttaatcccagcactcggga

ggcagaggcaggcagatttctgagttcgaggccagcctggtctacaaagt

gagttccaggacagccagggctatacagcaaaaccctgtctctaaaaaca

aaaaacaaacaaaacaaaacaaaacaaaagaacga--AAAGAAACACATG

TCTCTCTAGTTAGTTCTAAAACCACACATTGTTG-------AAATAATGA

A-AGGTGCAGTTT---GGAACGATA----------------------CCA

ACACTCTAAAGGGTTTCC---------TCTCATATCACACATCAGGTGAT

CACTAGTTG--ATATCCTCTGAGTCAGGAGACAAAGGCAATGACACACTA

GTTCACTGGTGTGACTGATAAGTCTGTCACGAGAGGC-------------

-----------------------ACTGG---GGACAGGTGGCCTGCCCCC

ACTTACCTGGAGGTCATTCTGGGGATTCCAGTCTGAATCAAAGATGATGC

AG

>rat

TGTGGCATCTAGCTTGAGCCTGGAAGGAAAG-AAAACCAAC---AGCTGT

---tgcaatgga-caccg-------------------gactgtg-tcttc

cctacatgtgcatgttggaaaagc-tgaccgaagtgttag---gcttaga

ggtgagcactcaggag-gtaattaggtcacgagggcggcgccctgaagaa

cgggattactg-ttctataagaagaggcagg-------------------

-----------------------agaatacagctgcctagg---------

---------cagcaacaacccagtcttgctggcatccagacctcacagtt

tcaa-ctccagaaccttgagtggggcatggatggctattggttaagctga

ccaatc-acaggagtttgtcagggcagccaaagtgaccaataca------

-------ACCCCTC------------------------------------

--------------------------------------------------

--------------------------------------------------

--------------------------------------------------

--------------------------------------AAGAAACGCATG

GCTCTTCAGTCGGTTATAAAA--CTGCATTATTA-------AAATAATTA

A-AGGCACGGTTT------AGAATA----------------------CTA

ACACACTAAGGGGTTTCC---TCTCATATTA-CACATCGGGTGATCAATA

GTTGACTTG--CTCTGAAATGGGAGTCAAAGGCTGAGCGATGACACACTA

GTTCACTGGTGTGGCTG---------------------------------

-----------------------ACAGG-------AGGCGGCCGTCTCCC

GCTTACCTGGAGGTCATTCTGGGGATTCCAGTCTGAGTCGAAGATGATGC

AG

>rabbit

TATGACATCTAGCTTGAGCCTGGAAGAGAAGGAGAATTAAT---GGGTGT

---tgcaatggagtcaa-------------------tgtttgtgta--cc

tacaaagttcctaagaggaaa--ctccagtcccactgggcggtgtctgga

ggtgaggcctcagggaagtaattaggtcatgagggtggagatctcataaa

actgatgagggctcacataaatgaggccaggagagccagtgcactctcct

tgggatatgtgaggacgaagcagaaagacagctgtctgtaaaccaggaag

agggccctcaccaagaacccaaccctgctagcaccctgatctcaggttt-

ccagtctctg----------------------------------------

--------------------------------------------------

-------CAATTGT------------------------------------

--------------------------------------------------

--------------------------------------------------

--------------------------------------------------

-------------------------------------AAGAACTGTGAAA

ACTAAATATTCACTGCTAAAG--TCC-TTTCAGGGTATTCTCAATAAATA

G-AGGTAGAGTC----AGAAGAGGG----------------------TTA

ACATAGTAAGAAATTTCTTCTTATATTATTAACAAATTTGTTGCTGGTTC

GTTGATTTGCCTGGAATCATGAACCACCGTCTGAGCAACAACACAAGCTA

C------------------AGATTCTCTGTCAGAGTA----CAATGAC-T

TTGTTTT-----------AAAGATATCAGGAAGA-----TTTTCCTCTGT

GTTTACCTGGAGGTCATTTTGGGGATTCCAGTCTGAATCAAAGATGATGC

AG

>human

TATGACATCTAGCCTGAGCCTGGAAGAAAAATGAAATTAAT---AGTTAC

---tgcaatgaacctaa-------------------tgtttctgtctccc

caccaaattcatatgctgaaa-ccctcattccaatgtgatggtatttgga

ggtggcgccttagggaagtaattaggtcatgaaggtggagtcctcacgaa

caggattactgctcatataaagaggcgagaga------gctggctctctt

taagctgggtgaggacacagcaagatgataactgtccataaaccaggaag

agggccctcaccaagaacctgactctgctggcactctgattttggactt-

ccaacctccagaactgtgag--aaataaatgtctgttgtt--taagccac

ccaatttatggtattttgttaaggagacctgagctaag---ac-------

-------GGCTACC------------------------------------

--------------------------------------------------

--------------------------------------------------

--------------------------------------------------

-------------------------------------AAAGAATGACAAG

ACTCATCTGCCAGTTCTAAGA--CCCTATCTATGTG----CCAATAAATA

G-AGGTGCAGTTT---AGAAGAAGG----------------------TTA

A--AACTAAAAAATTTCTTCTCACATTATTAATCAACAGATTACTAGTTG

GTT-ATTTGCTCTGAATCATAGGACACAGCCTGAGTGACGACGCAACATT

AATTTATGGGTCTGACT-GGTACTCTCTGTCATAGTAAGCAGAATGACTT

CCTTTTT----------------AAAGG---CATCAGGACTTTCTTGTGC

ATTTACCTGGAGGTCATTTTGGGGATTCCAGTCTGAATCAAAGATGATGC

AG

>chimpanzee

TATGACATCTAGCCTGAGCCTGGAAGAAAAATGAAATTAAT---AGTTAC

---tgcaatgaacctaa-------------------tgcttgtgtctccc

caccaaattcatatgctgaaa-ccctcattccaatgtgatggtatttgga

ggtggcgccttagggaagtaattaggtcatgaaggtggagtcctcacgaa

caggattactgctcatataaagaggcgagaga------gctggctctctt

taagctgggtgaggacacggcaagacgataactgtccataaaccaggaag

agggccctcaccaagaacctgactctgctggcactctgattttggactt-

ccaacctccagaactgtgag--aaataaatgtctgttgtt--taagccac

ccaatttatggtattttgttaaggagacctgagctaag---ac-------

-------GGCTACC------------------------------------

--------------------------------------------------

--------------------------------------------------

--------------------------------------------------

-------------------------------------AAAGAATGACAAG

ACTTATCTGCCAGTTCTAAGA--CCCTATCTATGTG----CCAATAAATA

G-AGGTGCAGTTT---AGAAGAAGG----------------------TTA

A--AACTAAAAAATTTCTTCTCACATTATTAATCAACAGATTACTAGTTG

GTT-ATTTGCTCTGAATCATAGGACACAGCCTGAGTGACGACGCAACATT

AATTTATGGGTCTGACT-GGGACTCTCTGTCATAGTAAGCAGAATGACTT

CTTTTTT----------------AAAGG---CATCAGGACTTTCTTGTGC

ATTTACCTGGAGGTCATTTTGGGGATTCCAGTCTGAATCAAAGATGATGC

AG

>treeshrew

TGTGACATCTAGCCTGAGCCTGGAAGAAAATGAAAATCAGA---GGCCAC

---tgcgatgaactgaa-------------------tgtttgtttcttcc

c-ccaaattcaagtgctgaa--tcgattgcaccctgatgttctctggtgg

ccgggtattccagaggtgcagatgtcgggagggaagagctctcgccaatg

ggatcagcgtcatagagaaagacagcaggggacta---gctggctccctc

t--gctctctgaggaagcagcgagaagacAgctt----------------

----ccccgactaaggacctggccctgctggcaccctgacctcaaactc-

ccaacctccagatgtgtgag---aacaaatgctggttgct--gaag---c

ccaagctgagatagggaccaaaggcaaagctcagtgccagtcggga----

-------GGCTGCA-tgttccaacgaatacgggtgcaatttaggagggga

gcagaccaag----------------------------------------

--------------------------------------------------

--------------------------------------------------

---------------------------------------------GAAAG

TTCCTCTTGTATT--------------------------CCTAATAAAAT

G-GGTACTAGTTGGTTGATCTGCCC----------------------TGA

ATCACCAGACACCGCCCCAGCAACAACACGTGACTGGTCTGACAGGGGTT

C----TTTG-TCATAGAATGGCTTACAATTCATTCACTCATTCA------

------------------------------------TTAAAATGAGATTA

AATTAAAAATAAAATAAATAAATAAAGGCACCAGGAAGACTTTTGGGTGC

ATTTACCTGGAGGTCATTTTGGGGATTCCAGTCTGAATCAAAGATGATGC

AG

>elephant

GATGACATCTAGCCTGAGCCTGGAAGGAAAATGAAATCAAT---------

--------------------------------------------------

--------------------------------------------------

--------------------------------------------------

--------------------------------------------------

--------------------------------------------------

--------------------------------------------------

--------------------------------------------------

--------------------------------------------------

-------GGCCATC------------------------------------

--------------------------------------------------

--------------------------------------------------

--------------------------------------------------

-------------------------------------AAAGAAAGAAAAG

ACTCAACTGCCAGTTAGACAA--GTGTATGTTC--------CAAAAAATA

G-CAGTGCAGCTT---AGGGGAGTG----------------------TTA

ACATACTAAGAAATAATATTTTACATTATTAATAAATTGACCACCAGTTG

GTTCATTTGCTACGAATCGTGGGGCACTGCCTGAGCAATGGCGCAACTTT

AATTTATCGG-TTTGGCTA-GGGAGTCTCCATCATAGCAGAATGACTT--

----------AAAAAAAAAAAAAAAAAGCATCAGCAAGATTTCCCTGCAT

GTTTACCTGGAGGTCATTTTGGGGATTCCAGTCTGAATCAAAGATGATGC

AG

>armadillo

TATGACACCTGGCCTGAGCCTGGAAGGAAGG-GAAATGAAG---------

--------------------------------------------------

--------------------------------------------------

--------------------------------------------------

--------------------------------------------------

--------------------------------------------------

--------------------------------------------------

--------------------------------------------------

--------------------------------------------------

-------GGCTATC------------------------------------

--------------------------------------------------

--------------------------------------------------

--------------------------------------------------

-------------------------------------AAAGAAAGAACAG

ACTCAACTGCCAGGGGTAAAA------GCCCGTGTG----CCCTTAAATA

A-AGGTGCAGTTG---AGAAGAGTGCTAACGTACAAAGAGATTGCTTTTC

ACATGACTAATAAATTG----------ACTAATAAAATGA--ACCAGTTG

GTTCATTTG---TCACGAATTGTGGGGCACCGCCCTGTTACCCCAACTGT

ACTTTTTTAT-TTCAAATA-GGGATTCTCCAGCACAGTCGAATGACTT--

------AAAAAAAAAAAAAAAAAAAAAGCATCAGCAAGATTTTCTTGCAT

GTTTACCTGGAGGTCATTTTGGGGGTTCCAGTCTGAATCAAAGATGATGC

AG
