# Supplementary material for: Retroposed Elements as Archives for the Evolutionary History of Placental Mammals
Source: PLoS Biol. 2006 Mar 14;4(4):e91. doi: 10.1371/journal.pbio.0040091 (PMC1395351; doi:10.1371/journal.pbio.0040091)
Supplement: Supplementary file 2 [file pbio.0040091.st001.doc]

#  Table S1. Detailed presence/absence patterns

|  |  | In silico | In vitro | **1a** | **1b** | **1c** | **1d** | **2a** | **2b** | **3a** | **3b** | **3c** | **3d** | **3e** | **3f** | **3g** | **3h** | **3i** | **3j** | **3k** | **4a** | **4b** | **4c** | **4d** | **5a** | **5b** | **5c** | **5d** | **5e** | **5f** | **5g** |
| --- | --- | --- | --- | --- | --- | --- | --- | --- | --- | --- | --- | --- | --- | --- | --- | --- | --- | --- | --- | --- | --- | --- | --- | --- | --- | --- | --- | --- | --- | --- | --- |
| ***Eutheria*** |  |  |  |  |  |  |  |  |  |  |  |  |  |  |  |  |  |  |  |  |  |  |  |  |  |  |  |  |  |  |  |
| **Laurasiatheria** |  |  |  |  |  |  |  |  |  |  |  |  |  |  |  |  |  |  |  |  |  |  |  |  |  |  |  |  |  |  |  |
| Bos taurus | cow | **X** |  |  | **+** |  | **+** |  | **+** |  |  | **+** |  |  | **+** |  |  | **+** | **+** | **+** | **+** | **+** |  |  | **-** | **-** |  | **-** | **-** | **-** | **-** |
| Sus scrofa | pig | **X** |  |  |  |  |  |  |  |  |  |  |  |  |  |  |  |  |  |  |  |  |  |  |  | **-** |  | **-** |  |  |  |
| Felis catus | cat | **X** |  |  |  |  |  |  |  |  |  |  |  |  |  |  |  |  |  |  |  |  |  |  |  | **-** |  |  |  |  |  |
| Canis familiaris | dog | **X** |  | **+** | **+** | **+** | **+** | **+** | **+** | **+** | **+** | **+** | **+** | **+** | **+** | **+** | **+** | **+** | **+** | **+** | **+** | **+** | **+** | **+** | **-** | **-** | **-** | **-** | **-** | **-** | **-** |
| Manis javanica | pangolin |  | **X** |  |  |  |  |  | **+** | **+** | **+** |  |  | **+** |  |  |  | **+** |  | **+** |  |  |  | **+** |  |  |  |  |  |  |  |
| Rhinolophus ferrumequinum | bat1 | **X** |  |  |  |  |  |  |  |  |  |  |  |  |  |  |  |  |  |  |  |  | **+** |  |  |  |  |  |  |  |  |
| Rhinolophus hipposideros | bat2 |  | **X** |  |  |  |  |  | **+** |  |  |  |  |  |  |  |  |  |  |  | **+** |  |  |  |  |  |  |  |  |  |  |
| Carollia perspicillata | bat3 | **X** |  |  |  |  |  |  |  |  |  |  |  |  |  |  |  |  |  |  |  |  |  | **+** |  |  |  |  |  |  |  |
| Myotis daubentonii | bat4 |  | **X** |  |  |  |  |  |  |  |  |  |  |  |  |  |  |  |  |  |  |  |  |  |  |  | **-** |  |  | **-** |  |
| Myotis lucifugus | bat4b | **X** |  | **+** |  |  | **+** |  |  |  |  |  | **+** |  | **+** |  |  | **+** |  |  |  |  |  |  |  | **-** |  |  |  |  | **-** |
| Pipistrellus pipistrellus | bat5 |  | **X** |  |  |  |  |  |  |  |  |  |  |  |  |  |  |  |  |  |  |  |  |  |  |  | **-** |  |  |  |  |
| Plecotus austriacus | bat6 |  | **X** |  |  |  |  |  | **+** |  |  |  |  |  |  |  |  |  |  |  |  | **+** |  |  |  |  |  |  |  |  |  |
| Erinaceus europaeus | hedgehog | **X** |  |  |  |  |  |  |  |  |  |  | **+** |  |  |  |  |  |  |  |  |  |  |  |  |  |  | **-** |  | **-** |  |
| Sorex araneus | shrew | **X** | **X** |  |  |  |  |  | **+** |  |  |  | **+** | **+** |  |  |  |  |  |  | **+** | **+** | **+** |  |  |  |  | **-** |  | **-** | **-** |
| Talpa europaea | mole |  | **X** |  |  |  |  | **+** |  |  |  | **+** |  | **+** |  |  |  | **+** |  |  |  |  |  | **+** |  |  |  |  |  |  |  |
| **Euarchontoglires** |  |  |  |  |  |  |  |  |  |  |  |  |  |  |  |  |  |  |  |  |  |  |  |  |  |  |  |  |  |  |  |
| Mus musculus | mouse | **X** |  | **+** | **+** | **+** | **+** | **+** | **+** |  | **+** |  | **+** | **+** |  | **+** | **+** | **+** | **+** | **+** | **-** |  | **-** | **-** | **+** | **+** | **+** | **+** | **+** | **+** | **+** |
| Rattus norwegicus | rat | **X** |  |  | **+** | **+** | **+** | **+** | **+** | **+** | **+** |  | **+** | **+** |  | **+** | **+** |  | **+** | **+** | **-** |  | **-** | **-** |  | **+** | **+** | **+** | **+** | **+** | **+** |
| Cavia porcellus | guineapig | **X** |  |  |  |  |  |  |  |  |  |  |  |  | **+** |  |  |  |  |  |  |  |  |  |  |  |  |  | **+** |  |  |
| Sciurus vulgaris | squirrel |  | **X** |  |  |  |  |  | **+** |  |  |  |  |  |  |  |  |  |  |  |  |  |  |  |  |  | **+** |  |  |  |  |
| Marmota marmota | marmot |  | **X** |  |  |  |  |  |  |  |  |  |  |  |  |  |  |  |  |  |  |  |  |  |  |  |  | **+** | **+** | **+** |  |
| Oryctolagus cuniculus | rabbit | **X** | **X** | **+** |  |  |  |  |  |  |  | **+** |  |  |  |  |  |  | **+** |  | **-** | **-** |  |  |  |  |  |  | **+** | **+** | **+** |
| Macaca mulatta | rhesusmonkey | **X** |  | **+** | **+** | **+** | **+** | **+** | **+** | **+** | **+** |  | **+** | **+** | **+** | **+** | **+** | **+** | **+** |  | **-** | **-** | **-** | - | **+** | **+** | **+** | **+** | **+** |  |  |
| Homo sapiens | human | **X** |  | **+** | **+** | **+** | **+** | **+** | **+** | **+** | **+** | **+** | **+** | **+** | **+** | **+** | **+** | **+** | **+** | **+** | **-** | **-** | **-** | **-** | **+** | **+** | **+** | **+** | **+** | **+** | **+** |
| Pan troglodytes | chimpanzee | **X** |  | **+** | **+** | **+** | **+** | **+** | **+** | **+** | **+** | **+** | **+** | **+** | **+** | **+** | **+** | **+** | **+** | **+** | **-** | **-** | **-** | **-** | **+** | **+** | **+** | **+** | **+** | **+** | **+** |
| Cynocephalus variegatus | flyinglemur |  | **X** |  |  |  |  |  |  |  |  |  |  |  |  |  |  |  |  |  |  |  |  |  |  |  |  |  | **+** | **+** |  |
| Tupaia belangeri | treeshrew | **X** | **X** |  |  |  | **+** |  |  |  |  |  |  |  |  |  |  |  |  |  |  |  |  |  |  |  | **+** | **+** | **+** |  | **+** |
| **Afrotheria** |  |  |  |  |  |  |  |  |  |  |  |  |  |  |  |  |  |  |  |  |  |  |  |  |  |  |  |  |  |  |  |
| Echinops telfairi | tenrec | **X** |  |  |  | **+** | **+** | **+** |  |  | **-** | **-** |  |  | **-** |  |  |  |  |  |  |  |  |  |  |  |  |  |  |  |  |
| Trichechus manatus | seacow |  | **X** | **+** |  |  |  |  | **+** | **-** |  |  |  | **-** |  |  |  | **-** |  |  |  |  |  |  |  |  |  |  |  |  |  |
| Loxodonta africana | elephant | **X** | **X** | **+** | **+** |  |  | **+** | **+** | **-** | **-** | **-** | **-** | **-** | **-** | **-** | **-** | **-** | **-** | **-** | **-** | **-** | **-** |  | **-** | **-** |  | **-** |  | **-** | **-** |
| **Xenarthra** |  |  |  |  |  |  |  |  |  |  |  |  |  |  |  |  |  |  |  |  |  |  |  |  |  |  |  |  |  |  |  |
| Choloepus hoffmanni | sloth |  | **X** | **+** |  | **+** |  | **-** | **-** | **-** | **-** |  |  | **-** |  |  |  | **-** |  |  |  |  |  |  |  |  |  | **-** |  |  |  |
| Dasypus novemcinctus | armadillo | **X** | **X** | **+** | **+** | **+** | **+** | **-** | **-** |  | **-** | **-** | **-** |  | **-** | **-** | **-** | **-** | **-** | **-** | **-** | **-** | **-** |  | **-** |  |  |  |  |  | **-** |
| ***Metatheria*** |  |  |  |  |  |  |  |  |  |  |  |  |  |  |  |  |  |  |  |  |  |  |  |  |  |  |  |  |  |  |  |
| **Didelphimorphia** |  |  |  |  |  |  |  |  |  |  |  |  |  |  |  |  |  |  |  |  |  |  |  |  |  |  |  |  |  |  |  |
| Didelphis virginiana | opossum | **X** |  | **-** | **-** | **-** | **-** |  |  |  | **-** | **-** |  |  | **-** |  |  |  |  |  |  |  |  |  |  |  |  |  |  | **-** |  |

Presence (+) and absence (-) patterns. *In silico* denotes markers supported by available GenBank entries. In vitro denotes markers supported by PCR amplifications. 1a-5g correspond

to the analyzed markers.
