# Supplementary material for: Retroposed Elements as Archives for the Evolutionary History of Placental Mammals
Source: PLoS Biol. 2006 Mar 14;4(4):e91. doi: 10.1371/journal.pbio.0040091 (PMC1395351; doi:10.1371/journal.pbio.0040091)
Supplement: Supplementary file 3 [file pbio.0040091.st002.doc]

# Table S2. Oligonucleotides used for PCR amplifications

| marker | (5’-3’) |
| --- | --- |
| 1a-F | GCTTCCATCTGAGCCAG |
| 1a-R | GAAGAGGCTAAAGAAGCAG |
| 1b-F | TTCCAAGAATATTTGACTACTC |
| 1b-R | GCTTTGAATACTGGCCTG |
| 1c-F | GCAGGACAGCCAGAAGTC |
| 1c-R | GACAGTCCTTGTTGCAGAAC |
| 1d-F | AGTACCTTAATGTCCTTAATGTG |
| 1d-R | TGACTACACCTTTGTGGCTG |
| 2a-F | CATGGAAAAGTATTTGAAGGC |
| 2a-R | CCTGTAGCCTATTTGCATTAG |
| 2b-F | CTGTGCAAATGTTAGGTTGTTC |
| 2b-R | AGAGGTAATTTACTGAAATACACAG |
| 3a-F | GGCATTAATTTAAATGGCCA |
| 3a-R | CAAGAGGGCTCAGCACATG |
| 3b-F | GTTTGTGGAGTTTCTTCAGGTC |
| 3b-R | GCATTTGCTGTTAGGTTAAATAC |
| 3c-F | CTGTTCGTATAGTTTCACAGC |
| 3c-R | CTGTGTAACGTGGAACAACAC |
| 3d-F | GAAGCAGTAGTGGATAATACAGATG |
| 3d-R | GGTACAGAATGGTGGCGAAC |
| 3e-F | TGGAAACTGTGCCATCTCAG |
| 3e-R | ACGCCCAGTGACCTAGACAG |
| 3f-F | AGAAGCTAGAACTTCAAGTATATGC |
| 3f-R | ATTTTGTTTTCCTGTTTAGTAAGTC |
| 3g-F | TCTCAGCTTTTACCAGGACAGA |
| 3g-R | TCTGGGTGGATGCCATCATA |
| 3h-F | AGCATGGCTATTTCTTCTGC |
| 3h-R | CCTGCAAGAAAGAATACAAGC |
| 3i-F | TGGTTATCCTTCGCAAATGATG |
| 3i-R | CCTCCATCACCTTTTGACACTG |
| 3j-F | AATTCAGAGTTTGGATTTATCTG |
| 3j-R | GTTGTTTGAATAATCTGCTACG |

| 3k-F | GAACTTTGCCAGCAGGTATG |
| --- | --- |
| 3k-R | CCAAACCGCTTACATTCTG |
| 4a-F | TTAAGTAGCATCTAGTGAAATCATC |
| 4a-R | GTTGTTTCTTACATACTTAAATACTGC |
| 4b-F | ACTCCCTGGATCTTGAGTAAATTG |
| 4b-R | TGCTTGCAATCTGATTGGC |
| 4c-F | AGACAGATACTATCACTTGTAGAGAC |
| 4c-R | GTATTTTAAGATTAAGACAACTTTCT |
| 4d-F | TGTGGTGTCCCCAGTAAGCAT |
| 4d-R | TGGACAGGCTATGTGTCGGAG |
| 5a-F | AAGACATTTCACTTTCTAGGGAAC |
| 5a-R | TGGGGCATTCCAAATTTCAT |
| 5b-F | TTCTCACTGGGCAGAAATAAG |
| 5b-R | CTGAATATATTAAACACTAGACTCAT |
| 5c-F | ATGACTATGAGACAGTAAGT |
| 5c-R | CCMTAAACCTACYCCAGTTATTC |
| 5d-F | GTGCTGATGACTTGTCATTAG |
| 5d-R | TAGGAAAAGAAAATGTAGCAG |
| 5e-F | CAGGTGGGTAGGCATGAG |
| 5e-R | GACTCCACTCACATTCTCCAG |
| 5f-F | GTCTCACATCCTGCACCAG |
| 5f-R | CTTCAAAGTCCACAAACACTC |
| 5g-F | TTAACCTCACTGCTGCTGATAC |
| 5g-R | CAGATTTGCTCTGTCCTATTC |
| M13-F | CCCAGTCACGACGTTGTAAAACG |
| M13-R | AGCGGATAACAATTTCACACAGG |

Marker designations correspond to Table1 and Fig.2. (F) denotes forward primers, (R) denotes reverse primers.
